# Deep learning based tumor–stroma ratio scoring in colon cancer correlates with microscopic assessment

**DOI:** 10.1016/j.jpi.2023.100191

**Published:** 2023-01-20

**Authors:** Marloes A. Smit, Francesco Ciompi, John-Melle Bokhorst, Gabi W. van Pelt, Oscar G.F. Geessink, Hein Putter, Rob A.E.M. Tollenaar, J. Han J.M. van Krieken, Wilma E. Mesker, Jeroen A.W.M. van der Laak

**Affiliations:** a Department of Surgery, Leiden University Medical Center, Leiden, The Netherlands; b Department of Pathology, Radboud University Medical Center, Nijmegen, the Netherlands; c Department of Medical Statistics, Leiden University Medical Center, Leiden, The Netherlands; d Center for Medical Image Science and Visualization, Linköping University, Linköping, Sweden

**Keywords:** Colon carcinoma, Tumor–stroma ratio, Computational pathology, Visual estimation, Artificial intelligence, Deep learning

## Abstract

**Background:**

The amount of stroma within the primary tumor is a prognostic parameter for colon cancer patients. This phenomenon can be assessed using the tumor–stroma ratio (TSR), which classifies tumors in stroma-low (≤50% stroma) and stroma-high (>50% stroma). Although the reproducibility for TSR determination is good, improvement might be expected from automation. The aim of this study was to investigate whether the scoring of the TSR in a semi- and fully automated method using deep learning algorithms is feasible.

**Methods:**

A series of 75 colon cancer slides were selected from a trial series of the UNITED study. For the standard determination of the TSR, 3 observers scored the histological slides. Next, the slides were digitized, color normalized, and the stroma percentages were scored using semi- and fully automated deep learning algorithms. Correlations were determined using intraclass correlation coefficients (ICCs) and Spearman rank correlations.

**Results:**

37 (49%) cases were classified as stroma-low and 38 (51%) as stroma-high by visual estimation. A high level of concordance between the 3 observers was reached, with ICCs of 0.91, 0.89, and 0.94 (all P < .001). Between visual and semi-automated assessment the ICC was 0.78 (95% CI 0.23–0.91, P-value 0.005), with a Spearman correlation of 0.88 (P < .001). Spearman correlation coefficients above 0.70 (N=3) were observed for visual estimation versus the fully automated scoring procedures.

**Conclusion:**

Good correlations were observed between standard visual TSR determination and semi- and fully automated TSR scores. At this point, visual examination has the highest observer agreement, but semi-automated scoring could be helpful to support pathologists.

## Introduction

The tumor–stroma ratio (TSR) has been shown to be an independent prognosticator in various forms of cancer and is scored by a pathologist using a conventional microscope, in relatively short time (<2 min).^1^^,^^2^ The parameter has been validated in multiple epithelial cancer types by research groups worldwide, including colon cancer.^2^, ^3^, ^4^ In general, patients with a large amount of stroma within the tumor (>50%; so-called “stroma-high” tumor) indicate an unfavorable phenotype, having an increased probability of disease recurrence or death by disease. Although the observer variation is good with Kappa values above 0.70,^1^ the visual estimation of the stroma percentage is subjective, and may impact the TSR classification, especially for tumors in which assignment to TSR status is relatively difficult (i.e., stroma percentage around the cut-off point of 50%).

For wide-scale introduction of the TSR in the decision-making process, reproducible and accurate assessment is mandatory. In this context, artificial intelligence has the potential to make assessment of the exact stroma percentage more objective and reproducible, especially in cases with a TSR around the 50% cut-off point. Since histopathology is becoming more and more digitalized, techniques as artificial intelligence now enter the field of cancer diagnostics. Artificial intelligence using deep learning approaches has been shown to perform at the level of experienced pathologists for tasks such as prostate cancer^5^ and breast cancer metastasis^6^ detection. Deep learning algorithms are also capable of distinguishing between tumor cells, tumor stroma, and other tissue components in scanned hematoxylin & eosin (H&E) stained sections of rectal cancer, as shown by Geessink et al.^7^ They used digital images to score the TSR and validated the prognostic value when scored with a semi-automated deep learning algorithm. A next step for automation and a difference from Geessink et al.^7^ would be a fully automated algorithm to score the TSR, which is explored in this study.

In the present study, the correlation between visual estimation of the TSR, semi- and fully automated TSR scoring using deep learning was investigated in colon cancer. The final goal is to add scoring TSR to current computational pathology procedures to support the pathologist in routinely scoring TSR.

## Materials and methods

### Case selection

The cases selected for this study were based on the E-learning module of the UNITED study.^8^ These cases were chosen for training purposes by limiting the number of stroma-low cases (e.g., 10% or 20% stroma cases), in order to increase the number of cases that are difficult to score for pathologists. All patients underwent surgical treatment at the Leiden University Medical Center (LUMC), had stage II or stage III colon cancer, and no patients received neo-adjuvant treatment. The slides were anonymized and scanned with the Panoramic 250 scanner (3DHistech, Hungary) (tissue level pixel size ∼0.33 μm/pixel), or with the IntelliSite Digital pathology slide scanner (Philips, the Netherlands) (tissue level pixel size ∼0.25–0.26 μm/pixel).

### Visual estimation of the tumor–stroma ratio

The TSR was scored on H&E stained sections, as described in detail by van Pelt et al.^1^ In brief, in the general procedure for visual TSR assessment, the region within the tumor with the highest relative amount of stroma is selected using a conventional microscope at low magnification. The region is subsequently zoomed to 100x magnification, and a field of view is selected in which tumor cells are visible on all opposite sides of the field. Areas with abundant necrosis, muscle tissue, and fatty tissue are not taken into account. Next, the percentage of stroma occupying the field of view is visually estimated using increments of 10%. In the present study, visual TSR scoring was performed using scanned slides, requiring a slight modification to the above described procedure.^9^ A visual annotation was placed in the scanned image, mimicking the surface area of a field of view of a 100x magnification of a microscope. The annotations were placed by 2 experienced TSR researchers (MS placed the annotations, GvP checked the position of the annotations) in the area with the highest amount of stroma by eye (TSR hot spot). Thereafter, the stroma percentage was scored by 3 observers (HvK, GvP, and MS) within this annotation.

### Semi-automated scoring of the tumor–stroma ratio

**Fig. 1:**
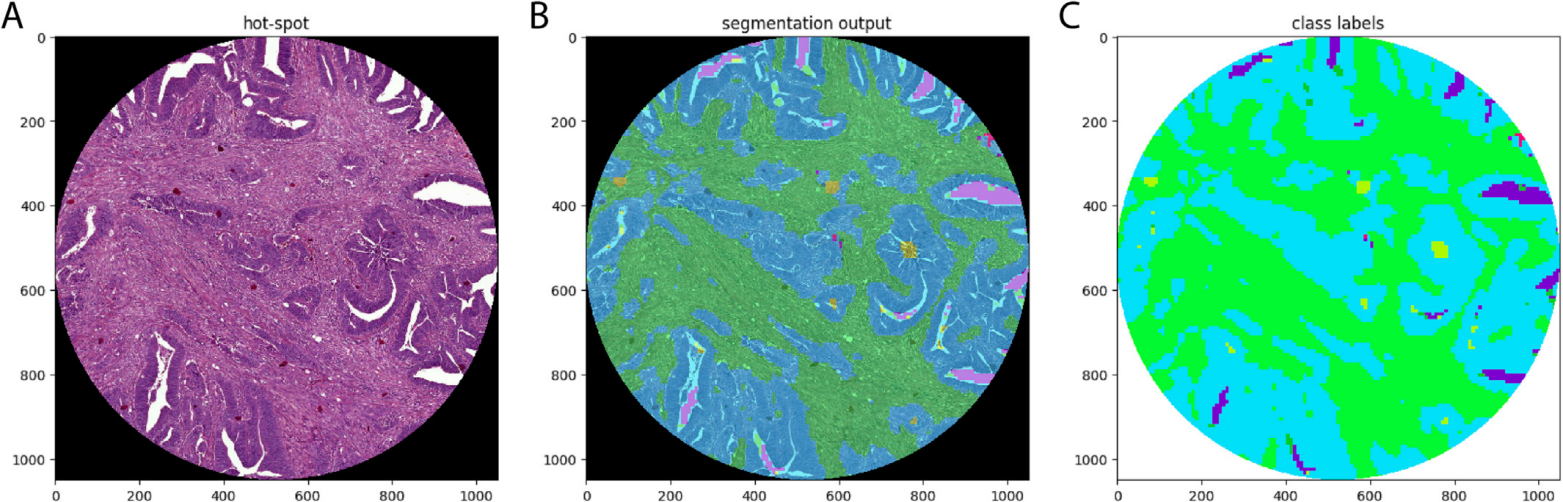
An example of the workflow output for semi-automated scoring algorithm. In (A) H&E stained sections in the spot chosen by microscopic assessment. (B) The first step was making a segmentation output, before in (C) the class labels can be displayed.

The semi-automated TSR was scored using a deep learning algorithm in the hot-spot regions selected for the visual TSR scoring. A deep learning algorithm based on fully convolutional neural networks (FCN)^10^ was used to process all whole-slide images (WSIs) at 20X magnification (0.5 μm/pixel), in order to segment multiple tissue types in each slide, i.e., predict the label of each pixel in the WSI. The FCN was built and trained as described by Geessink et al.^7^ A training dataset of manually annotated regions of colon tissue samples from Radboud University Medical Center, Nijmegen (Netherlands) was used for model development.^11^ In order to compensate for the difference in H&E staining between the single-center training set and the research population in this study, a recently developed stain normalization algorithm was adopted, based on cycleGAN model,^12^ capable of transferring the style (i.e., the H&E staining) from one image to another, based on a pre-defined template (i.e., the H&E staining of the training set). As a result, the FCN could label each pixel as one of the following classes: tumor glands, tumor-associated stroma, necrosis, lymphocytes, erythrocytes, muscle, healthy stroma, fatty tissue, mucus, nerve, stroma lamina propria, healthy glands, where “healthy stroma” indicates connective tissue not associated with the tumor. The FCN was used to classify all pixels in the manually selected regions of interest, and the semi-automated TSR was computed as the percentage of stromal pixels within the whole hot-spot area minus mucus, necrosis, and/or background. For determination of the TRS, all tumor-associated stroma, lymphocytes, erythrocytes, muscle, healthy stroma, nerve, and stroma lamina propria pixels were defined as stromal pixels. See Fig. 1 for the output of the semi-automated deep learning algorithm.

### Fully automated scoring of the tumor–stroma ratio

The fully automated TSR was computed by extending the application of the same FCN model used in the semi-automated approach to the entire WSI. For this purpose, 3 main components had to be defined, to cope with challenges that emerge when the TSR analysis is not restricted to human-provided hot-spots but rather to the entire WSI: (1) define the tumor-associated area of the WSI where TSR can be computed, (2) define the type and proportion of tissues involved in the computation, and (3) define the statistics to use to compute TSR. All the aspects are described in this section.

1. Tumor-associated area. Mimicking the visual estimation method, the fully automated TSR deep learning score should be determined on tissue likewise scored as the manual procedure. Based on a circular region of interest, equivalent to the one used for microscopic assessment, the TSR should then be computed by automated analysis. These points were addressed as follows. First, the analyses were limited to the “tumor bulk” region, defined as the area of the WSI that completely encloses all tumor glands predicted by the FCN (Fig. 2). The invasive edge and budding zone are considered part of the tumor bulk. In practice, the tumor map was extracted from the predicted segmentation and it was post-processed by running a “concave hull” algorithm, to get rid of small false positives and to identify the bulk of colon carcinoma. Second, the tumor bulk regions were considered valid when it was possible to completely fit the circular field of view of diameter d=2.0 mm, and where those fields of view did not contain any background regions (i.e., lack of tissue) or tissue tears. In practice, this resulted in an erosion of the tumor bulk area using the field of view as a structuring element.

**Fig. 2:**
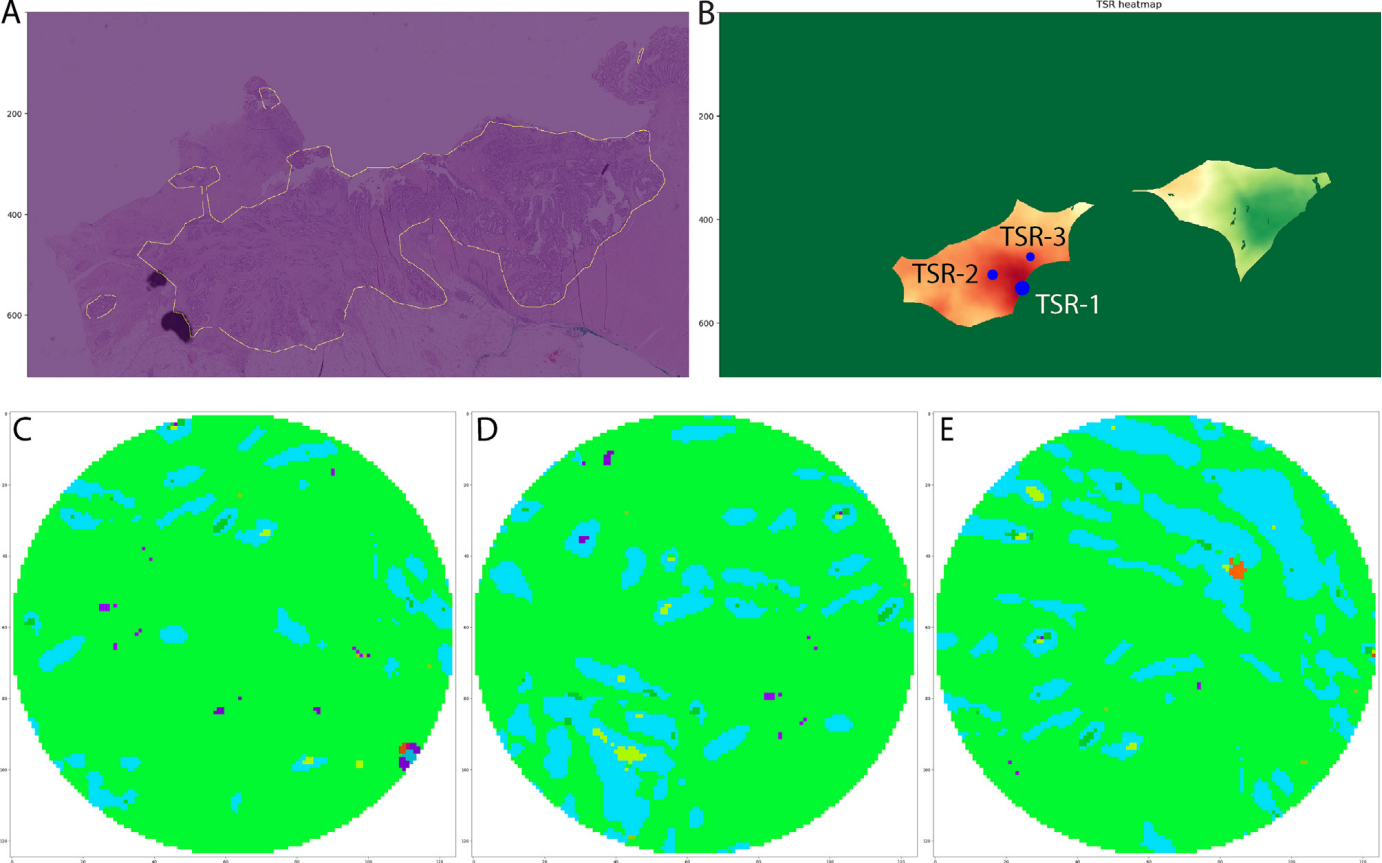
The output from the full automated workflow. In (A) the tumor bulk is annotated. In (B) the heatmap is created. The biggest dot corresponds with the highest stroma-percentage (TSR-1), the second biggest with the second highest (TSR-2), etcetera. In (C) the class output of the highest spot (TSR-1), in (D) the second highest spot (TSR-2) and in (E) the third highest spot (TSR-3).
2. Tissue types. Within the valid tumor bulk area, a set of tissue types was considered to compute the TSR score. Driven by good practices learned during visual assessment, rules were added to consider regions of interest as valid: area of fatty tissue should be <5% of the field of view; presence of erythrocytes should be <10% of the area of the field of view; the area of necrosis should be <30% of the field of view. Additionally, the “tumor” area was defined as the combination of tumor glands and healthy glands. Healthy glands within the tumor bulk as assigned by the algorithm appeared on visual inspection to be well differentiated tumor glands and thus were included as tumor in determination of the TSR. The “stroma” was defined as the combination of tumor-associated stroma, lymphocytes, nerves, and erythrocytes.
3. Computing TSR. Based on the semi-automated approach, for each circular input region of interest, a TSR score can be computed in the valid tumor bulk area considering the tissue types previously defined. Therefore, simply sliding this circular area over the entire WSI image would produce a TSR prediction at every location, eventually producing a “TSR heatmap” (Fig. 2). In practice, to make this operation efficient, the circular area was treated as a binary filter and computed the TSR map via convolution of the predicted classes of interest in the segmentation map and the circle itself in the Fourier domain. Driven by the definition of TSR hot-spot, the location was considered with the maximum value of stroma in the TSR heatmap as the best candidate for automated TSR, which was reported as TSR-1. However, due to misclassified regions within the tumor bulk, a rank of hot regions was considered. Starting from TSR-1, to include additional TSR values in the analysis. For this purpose, the top-3 TSR values were taken, namely TSR-1, TSR-2, and TSR-3 (Fig. 2), each computed as the maximum value of the TSR heatmap after zeroing the previous hot-spot using a region as large as the field of vision.

### Statistical analysis

The visual estimation percentage was based on the scores of the 3 observers. When all observers or 2 out of 3 scored the same percentage, this percentage was seen as the consensus percentage for visual estimation. For the other cases, a consensus meeting was set up to determine the percentage. These categorical percentages were used for intraclass correlation analysis. For interobserver analysis, the cases were classified as stroma-low (≤50% stroma) or stroma-high (>50% stroma)^1^^,^^2^ and Cohen’s kappa coefficient was used as method. The semi- and fully automated deep learning algorithm data were classified in the same categories as the visual estimation and were dichotomized in the same groups as described above (≤50% stroma or >50% stroma). The intraclass correlation coefficient (ICC) with corresponding 95% confidence interval (95% CI) was used to study the relationship between the 3 observers, and between the visual TSR assessment and semi-automated TSR deep learning algorithm. In addition, as the visual TSR assessment uses a slightly different definition of TSR compared to the automated assessments, the visual and deep learning TSR values (semi-automated as well as fully automated) were compared using Spearman rank correlations. Bubble plots were used for data visualization. In addition, Bland–Altman plots were created to study differences between measurement methods (data not shown). T-tests were used for hypothesis testing. P-values <.05 were considered significant. Statistical analyses were performed using IBM SPSS software version 25.0 (SPSS, Inc. an IBM Company Chicago, IL, USA).

## Results

### Visual estimation results

In total, 75 cases were analyzed in this study. In 32 cases (43%), all 3 observers scored the same percentage, and in 37 cases (49%), 2 out of 3 observers scored the same percentage. In 6 cases (8%), all observers scored a different percentage, impeding a consensus based on majority vote. These were discussed in a consensus meeting with the 3 observers, resulting in a consensus stroma percentage for all cases. Of the 75 cases, 37 (49%) were classified as stroma-low and 38 (51%) were classified as stroma-high by visual estimation. Twenty cases (27%) had a TSR score directly adjacent to the cut-off, being either 50% or 60% stroma. The observer agreements were good, with kappa scores of 0.68, 0.70, and 0.89 between pairs of observers. The associated ICCs confirm the high level of concordance, with values of 0.91, 0.89, and 0.94 (all P < .001; see Table 1).

**Table 1:** Overview of the interobserver agreements and correlations between the 3 observers.

Observers	Kappa	ICC	95% CI	P-value
1 vs 2	0.68	0.91	0.85–0.94	<.001^a^
1 vs 3	0.70	0.89	0.83–0.93	<.001^a^
2 vs 3	0.86	0.94	0.91–0.96	<.001^a^

ICC = intraclass correlation coefficient, 95% CI = 95% confidence interval.

a Significant results.

### Results of the semi-automated assessment of the TSR

**Fig. 3:**
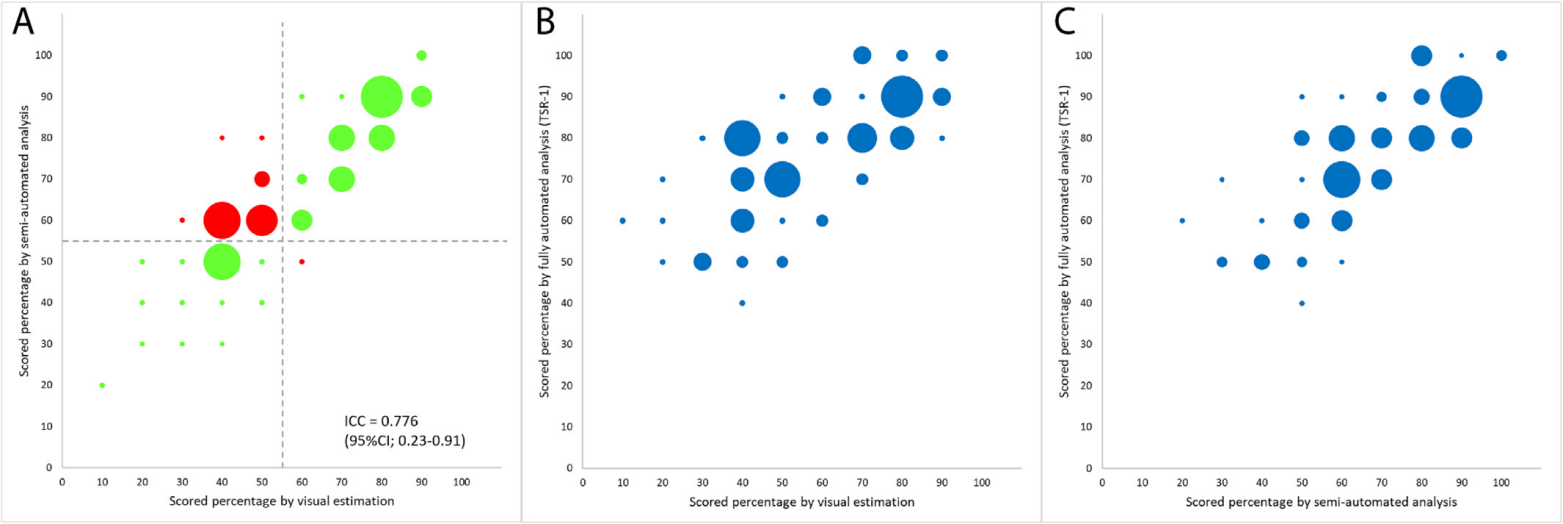
Bubble plot of assessed stroma percentage of the 75 cases. In (A) for the visual (consensus) scores plotted against the semi-automated scores. The lines indicate the cut-off between stroma-low and stroma-high. Green dots indicate cases where agreement was reached and dots in red indicate disagreement. In (B) the visual scores plotted against the highest fully automated scores (TSR-1), and in (C) the semi-automated scores plotted against the highest fully automated scores (TSR-1).

The semi-automated algorithm analyzed the identical tissue regions as used for visual assessment. Applying the 50% cut-off, 19 cases (25%) were classified as stroma-low and 56 (75%) cases as stroma-high. In total, 29 (39%) cases were adjacent to the 50% cut-off value (either 50% or 60%) for the semi-automated assessment. The ICC between visual and semi-automated assessment was 0.78 (95% CI 0.23–0.91, P-value .005), with Spearman rank correlation of 0.88 (P < .001). The relationship between visual and semi-automated assessment is shown in Fig. 3.

### Results of the fully automated assessment of the TSR

Results from fully automated assessment, applying the cut-off value of 50%, showed 9 (12%) stroma-low cases and 65 (88%) stroma-high cases. One case could not be assessed because the tumor area detected by the algorithm was too narrow to fit the circular measurement region with 2.0 mm diameter as used for fully automated deep learning assessment. Given the systematic difference between visual TSR assessment and fully automated TSR assessment, Spearman correlation coefficients were calculated to study concordance between the 2 methods. Spearman correlation coefficients of respectively 0.72, 0.77, and 0.75 between the 3 fully automated highest TSR spots (TSR-1, TRS-2, and TSR-3) and the consensus visual TSR were observed (all P < .001; see Table 2). Comparing semi- and the 3 fully automated assessments, a significant positive correlation was observed, with Spearman correlation coefficients of respectively 0.76, 0.83, and 0.80 (all P < .001; see Table 2). Fig. 3 shows plots comparing fully automated assessment with both visual and semi-automated assessment.

**Table 2:** Overview of the correlations between the different methods.

Methods	Spearman correlation	P-value
Visual estimation vs fully automated 1 (TSR-1)	0.72	<.001^a^
Visual estimation vs fully automated 2 (TSR-2)	0.77	<.001^a^
Visual estimation vs fully automated 3 (TSR-3)	0.75	<.001^a^
Semi-automated vs fully automated 1 (TSR-1)	0.76	<.001^a^
Semi-automated vs fully automated 2 (TSR-2)	0.83	<.001^a^
Semi-automated vs fully automated 3 (TSR-3)	0.80	<.001^a^

a Significant results.

**Fig. 4:**
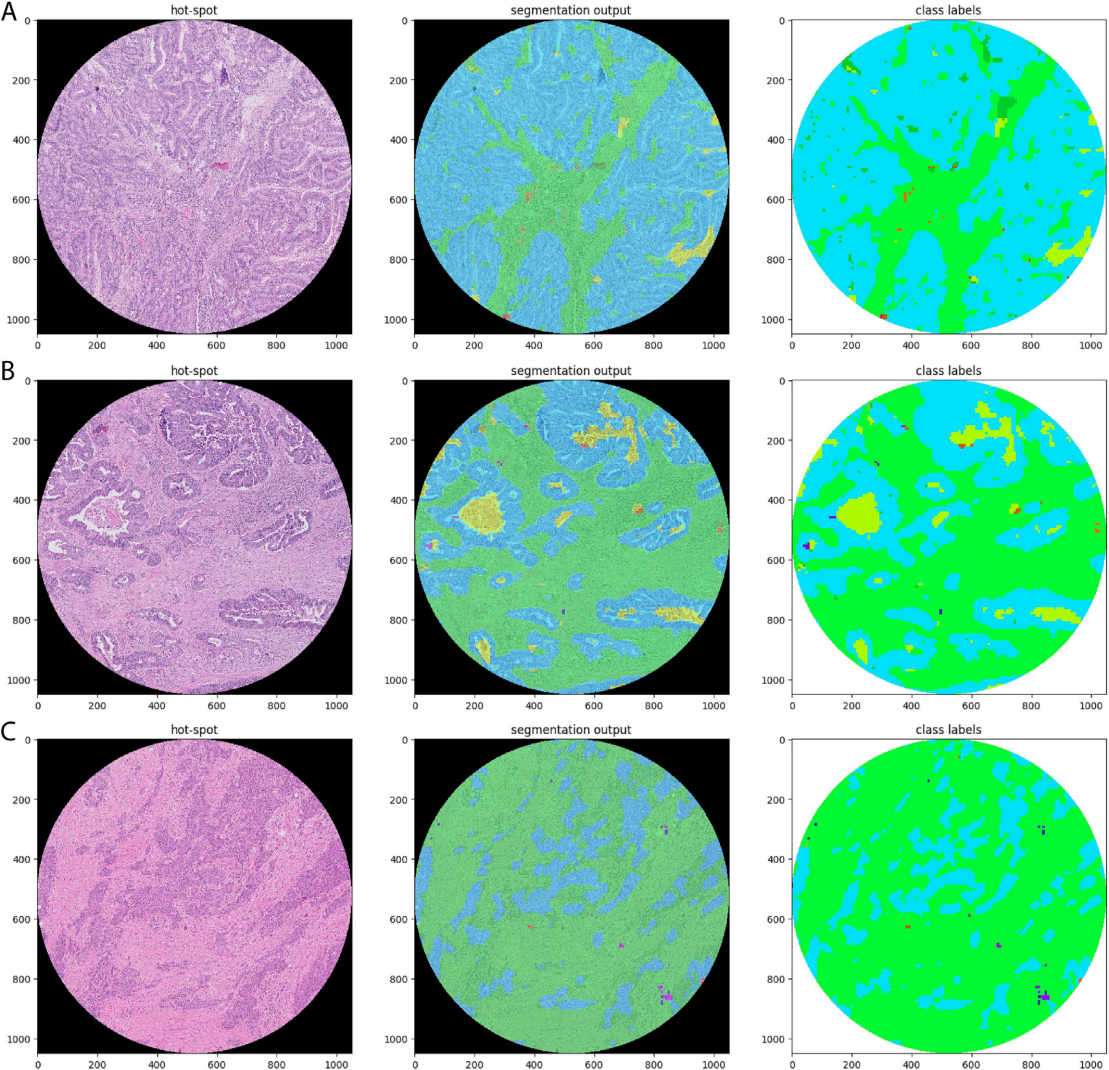
Three examples for the semi-automated output. (A) Agreement between visual estimation and semi-automated score (20% stroma), (B) agreement between visual estimation and semi-automated score (60% stroma), (C) by visual estimation 50% stroma was scored, 80% stroma was the semi-automated outcome due to not always recognized tumor cells.

The correlation between the visual assessment and semi- or fully automated assessment was generally good, but a number of large discrepancies were observed. In Fig. 4, examples are shown with agreement on stroma-low and on stroma-high status, as well as a discrepancy between visual TSR assessment and semi-automated deep learning assessment. Supplementary Fig. 1 shows 2 examples of a suboptimal selection of the measurement region by the fully automated deep learning algorithm.

## Discussion

This study focused on the use of computational pathology for scoring the TSR. The standard approach of scoring consisted of human visual estimation. In the current study, we investigated whether an automated analysis using artificial intelligence (more specifically: deep learning algorithms), applied in either a fully- or semi-automated manner could reach comparable results, especially for cases that are difficult to score, i.e., with a TSR score within 10 % of the commonly applied 50% cut-off point. The cases used in this study were chosen for training purposes,^8^ and therefore did not follow the normal TSR stroma-low/-high percentage distribution as would be expected in a cohort study. To the best of our knowledge, this is the first study in which these 3 methods are compared.

The TSR is easy to score by visual inspection of H&E stained slides during routine pathology, taking only 1–2 min. The interobserver agreement was shown to be substantial to good.^1^ Most common discrepancies between observers are the cases around the cut-off value of 50%. Scoring these, more difficult, cases with deep learning algorithms could be supportive to the pathologist.

With 3 experienced observers scoring the visual stroma percentage and using all scores together as the consensus agreement, this measurement can be seen as a solid visual estimation and a good basis for a comparison with the 2 computer-based methods. In this study, the annotations were placed upfront by one of the observers, to reach a consensus for the stroma percentage for the annotation.

The semi-automated deep learning algorithm can be seen as a fourth observer or as a new scorings method. When handling the semi-automated method as an observer the ICC can be used. The ICC of 0.77 between the visual estimation and the semi-automated deep learning algorithm output was good. If the semi-automated score is handled as a new scoring method, the Spearman correlation showed an even better correlation between the visual estimation and the semi-automated output (0.88). These results are comparable with earlier research in rectal cancer,^7^ where TSR was scored semi-automated by deep learning. Another semi-automated method recently used is point-counting. This is a comparable method, using a grid placed as layover and each point representing a tissue type which is visually assigned by a pathologist. After point classification, the stroma percentage is measured.^13^^,^^14^ Point-counting is useful to quantify the exact stroma percentage and showed good Kappa values within this method,^13^ but is time consuming and more subjective because all points have to be classified manually compared to a semi-automated deep learning algorithm. The results in the study from West et al. showed that semi-automated TSR assessment by point-counting is a good method to measure the prognostic value of the TSR. In this study, no comparison with visual TSR assessment has been made.^13^ Zhao et al.^15^ scored the TSR fully automated on whole-slide images, using the whole tumor area. No hotspot was chosen, therefore, their results are not comparable with microscopic results.

The second highest hot spot (TSR-2) determined by the fully automated deep learning algorithm corresponds best with the visual TSR assessment. Upon reviewing the cases, the suggestion is that in the highest spot more tissue types are classified, which are not taken into account when determining the stroma percentage visually. So, the highest spot has more “noise” than the second highest spot calculated by the fully automated deep learning algorithm.

One of the challenges in developing deep learning algorithms for histopathology is the large variability between slides, largely caused by variations in the H&E staining. Unless standardized protocols for H&E staining are strictly followed, there is a considerable variability in staining characteristics among and even within laboratories. While a pathologist will easily adapt to such differences, the performance of a deep learning algorithm may be very sensitive to variations not encountered in the slides used for training. Several techniques, such as stain normalization and augmentation, exist to make deep learning algorithms more robust for use in histopathology.^16^ The cases in the current study were stained in another laboratory than where the original deep learning algorithms were developed. Before the slides could be analyzed for TSR, stain normalization and augmentation took place (Supplementary Fig. 2 shows an example). The cycleGAN method was necessary for optimal functioning of the semi- and fully automated deep learning algorithms. Another challenge, was that the protocol for visual scoring TSR cannot be easily transferred to an algorithm. In stroma-high cases, it is of importance when scoring TSR by microscope that on 4 sides of the vision field tumor cells are present. Translating visual interaction into an algorithm is difficult. Therefore, for the fully automated algorithm all stroma within the tumor bulk was used for calculation of TSR (TSR-1, TSR-2, and TSR-3). We conclude that, before the algorithms can be used in daily practice, a validation study should be performed using the in house H&E staining procedure, and including the adjusted stain normalization steps. In this case, it would also be of interest to (re-)investigate the cut-off value of the TSR, especially when using the (semi-)automated method (both binary and numerically). Also because of the fact that in earlier research about quantifying the percentage of malignant nuclei, it appeared that humans score systematically higher compared to a computer algorithm.^17^^,^^18^

However, with current digitization of the pathology workflow, more and more routine histology and new biomarkers become applicable using artificial intelligence methods offering standardization and robustness, both most valuable standards in strategies for personalized treatment for the patient with colon cancer. To easily improve artificial intelligence workflows, pathology images and patient data should be connected at the source.^19^ When all laboratories are able to connect this way, data (including pathology images) can be easily shared and used.

To conclude, this study showed good correlations between the TSR scored by microscope and using deep learning algorithms. No conclusions however can be drawn for the method of preference, because no survival data were available. The UNITED study^8^ will provide answers to these remaining questions, as within this European validation study, including 1500 colon cancer patients, the TSR will be scored microscopically and by deep-learning algorithms.

The following are the supplementary data related to this article.

Supplementary material 1 Examples of cases where the fully automated spot is not ideal chosen because in A) not on all sides are tumor cells so by eye one would say it is not possible to tell whether it is normal stroma or tumor stroma, the spot in B) is not optimal because of the amount of necrosis. Supplementary material 1

Supplementary material 2 In A) an example of the output of the stain normalization procedure, in B) the stain augmentation process is visualized. Supplementary material 2

Supplementary material 3 Output for all cases for the semi-automated algorithm and the fully-automated algorithm. *In part 1 case 1-24, part 2 case 25-49 and part 3 50-75*. Supplementary material 3

## Funding

Marloes Smit and Gabi van Pelt received partly financial support from a grant from the 10.13039/501100004622Dutch Cancer Society (KWF) / Alpe d’HuZes fund (project 2016-10174). Francesco Ciompi received partly support for this research by a grant from the KWF / Alpe d’HuZes fund (KUN 2014-7032), and by the 10.13039/100010661European Union's Horizon 2020 Examode project; No 825292 (ExaMode, htttp://www.examode.eu/).

Furthermore, this research did not receive any specific grant from funding agencies in the public, commercial, or non-profit sectors.

## Ethical considerations

The UNITED study protocol has been approved by the Medical Research Ethics Committee (MREC) of the LUMC, study number p17.302. All patient material handled in the current study was in accordance with the 1964 Helsinki declaration and its later amendments, and the Code of conduct.

## Conflict of interest

Jeroen van der Laak is member of the scientific advisory boards of Philips, the Netherlands and ContextVision, Sweden and receives research funding from Philips, the Netherlands and Sectra, Sweden. Francesco Ciompi is member of the scientific advisory board of TRIBVN, France. All other authors declare they have no conflicts of interest.

## Data Availability

The dataset analyzed during the current study is available from the corresponding author on reasonable request. All output from the semi-automated and fully-automated algorithm are attached in Supplementary Fig. 3.
