# Supplementary material for: Deep learning based tumor–stroma ratio scoring in colon cancer correlates with microscopic assessment
Source: J Pathol Inform. 2023 Jan 20;14:100191. doi: 10.1016/j.jpi.2023.100191 (PMC9922811; doi:10.1016/j.jpi.2023.100191)
Supplement: Supplementary material 1 — Examples of cases where the fully automated spot is not ideal chosen because in A) not on all sides are tumor cells so by eye one would say it is not possible to tell whether it is normal stroma or tumor stroma, the spot in B) is not optimal because of the amount of necrosis. [file mmc1.pdf]

Deep learning based tumor-stroma ratio scoring in colon cancer correlates with microscopic assessment. *Marloes A Smit, et al.*

Supplemantary figure 1: Examples of cases where the fully automated spot is not ideal chosen because in A) not on all sides are tumor cells so by eye one would say it is not possible to tell whether it is normal stroma or tumor stroma, the spot in B) is not optimal because of the amount of necrosis.

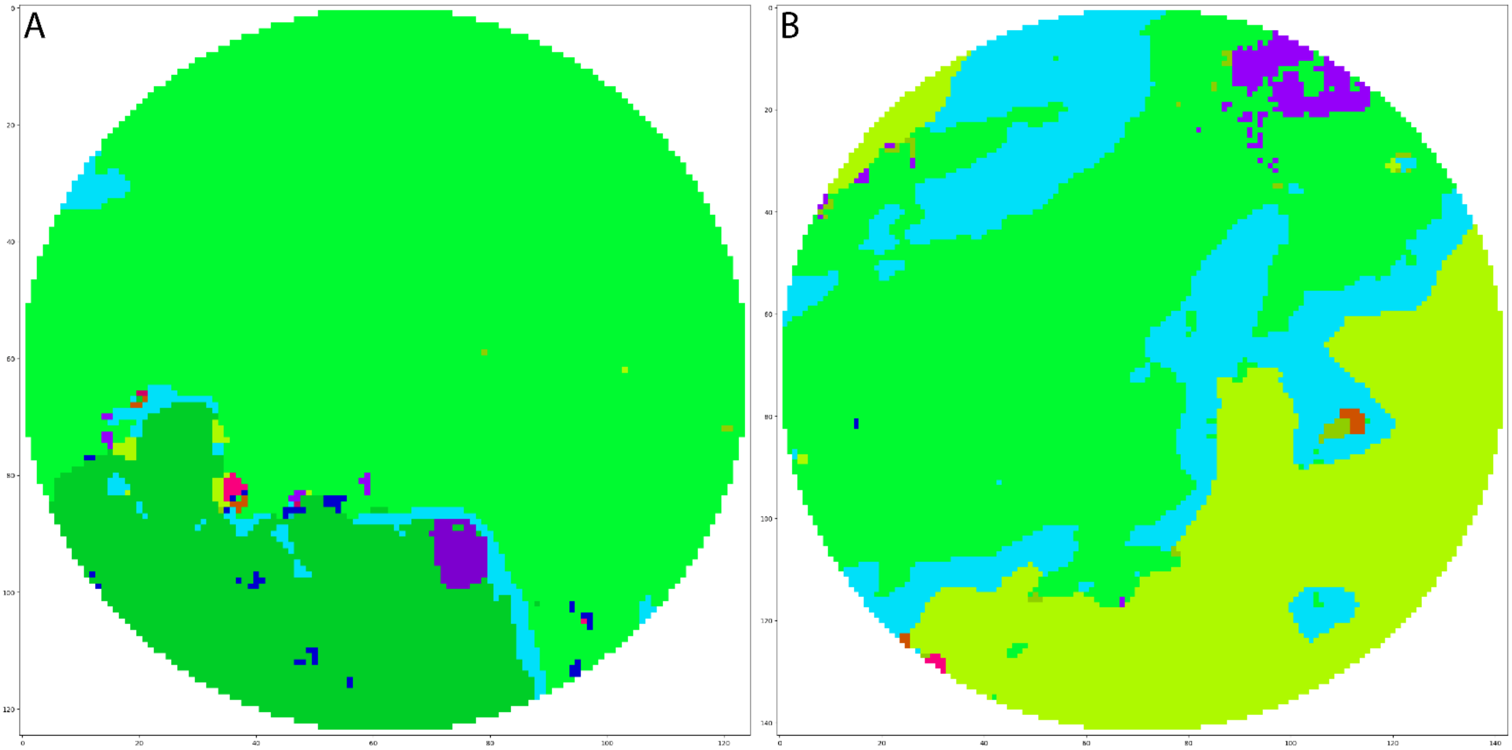

Light blue is tumor, light green is tumor-stroma, dark green is mucus, yellow is necrosis, purple is muscle.
