# Supplementary material for: Deep learning based tumor–stroma ratio scoring in colon cancer correlates with microscopic assessment
Source: J Pathol Inform. 2023 Jan 20;14:100191. doi: 10.1016/j.jpi.2023.100191 (PMC9922811; doi:10.1016/j.jpi.2023.100191)

Deep learning based tumor-stroma ratio scoring in colon cancer correlates with microscopic assessment. *Marloes A Smit, et al.*

Supplemantary figure 2 In A) an example of the output of the stain normalization procedure, in B) the stain augmentation process is visualized.

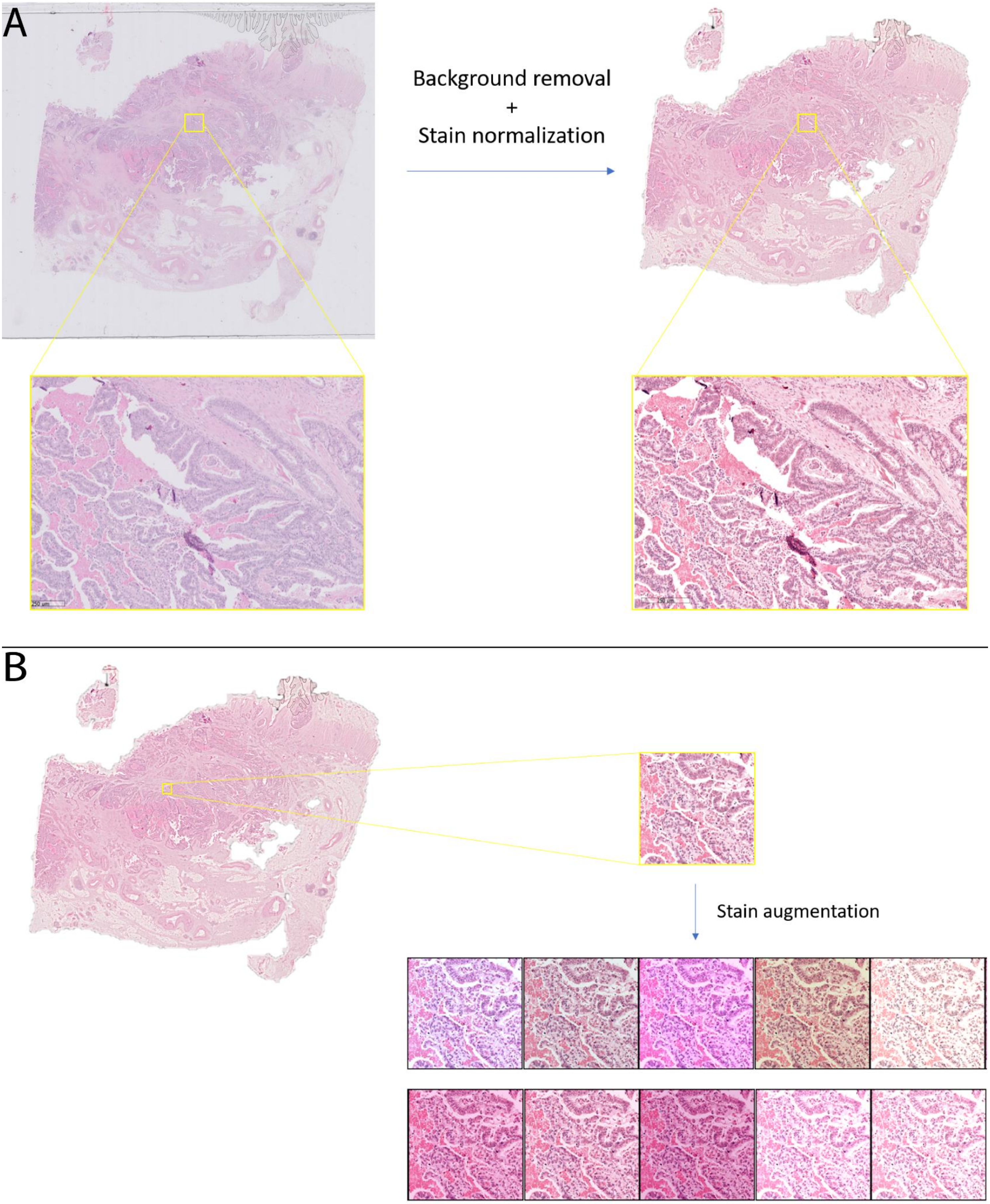

Supplement: Supplementary material 2 — In A) an example of the output of the stain normalization procedure, in B) the stain augmentation process is visualized. [file mmc2.pdf]
