# Supplementary material for: Deep learning based tumor–stroma ratio scoring in colon cancer correlates with microscopic assessment
Source: J Pathol Inform. 2023 Jan 20;14:100191. doi: 10.1016/j.jpi.2023.100191 (PMC9922811; doi:10.1016/j.jpi.2023.100191)

Supplementary file

Original data figures

(Part 1)

Legend

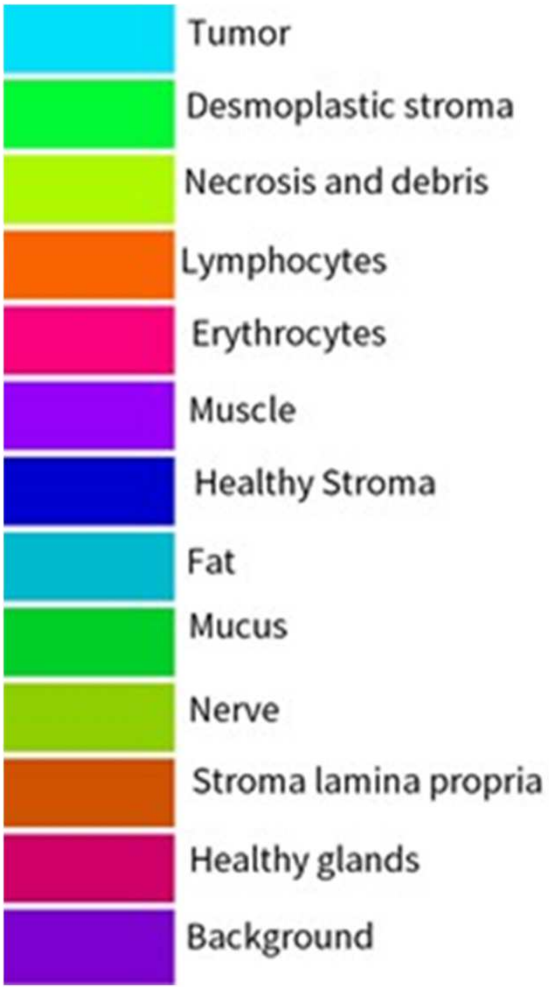
Tumor
Desmoplastic stroma
Necrosis and debris
Lymphocytes
Erythrocytes
Muscle
Healthy Stroma
Fat
Mucus
Nerve
Stroma lamina propria
Healthy glands
Background

<u>Case 1</u>

Semi-automated output

Left: H&E stained section in the spot chosen by microscopic assessment. Middle: the first step was making an segmentation output. Right the class labels can be displayed

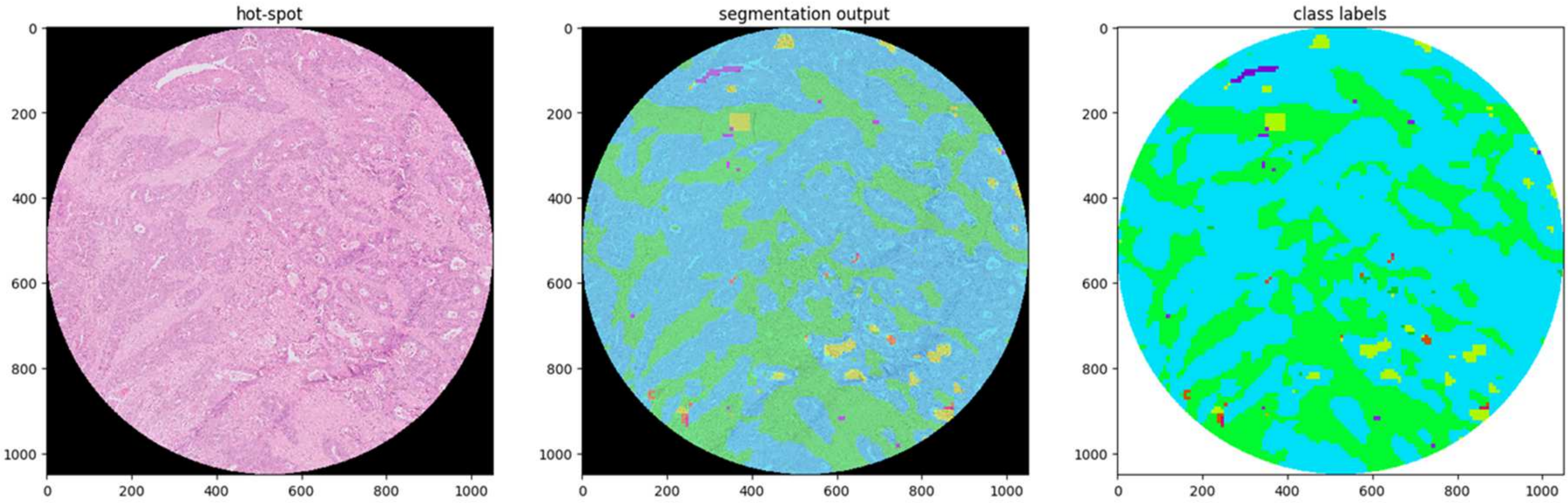

Fully-automated output

Top row; left: the tumor bulk is annotated. Right: heatmap is created. The biggest dot corresponds with the highest stroma-percentage (TSR-1), the second biggest with the second highest (TSR-2), etcetera.

Bottom row; left: the class output of the highest spot (TSR-1), middle: the second highest spot (TSR-2) and right the third highest spot (TSR-3)

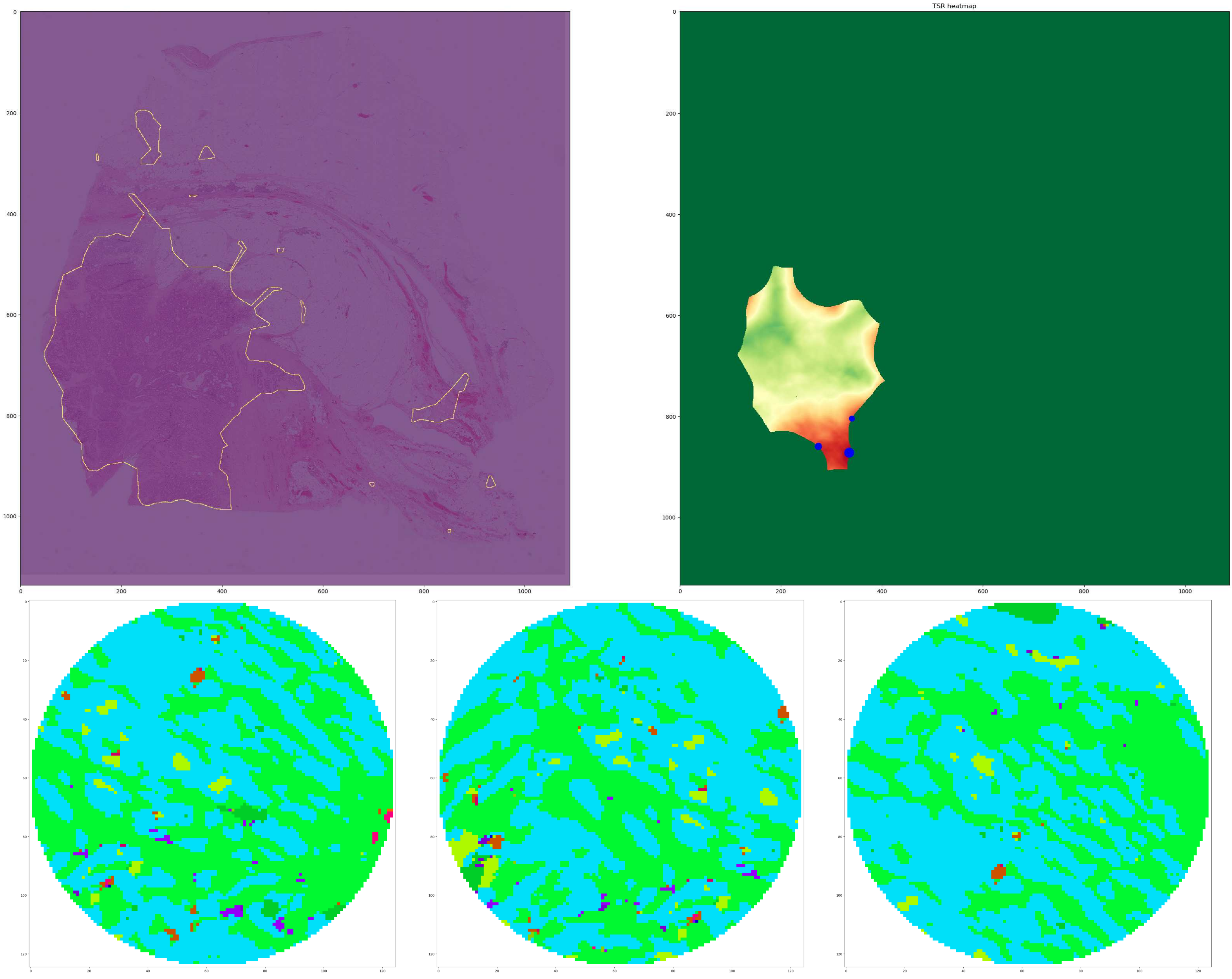

<u>Case 2</u>

Semi-automated output

Left: H&E stained section in the spot chosen by microscopic assessment. Middle: the first step was making an segmentation output. Right the class labels can be displayed

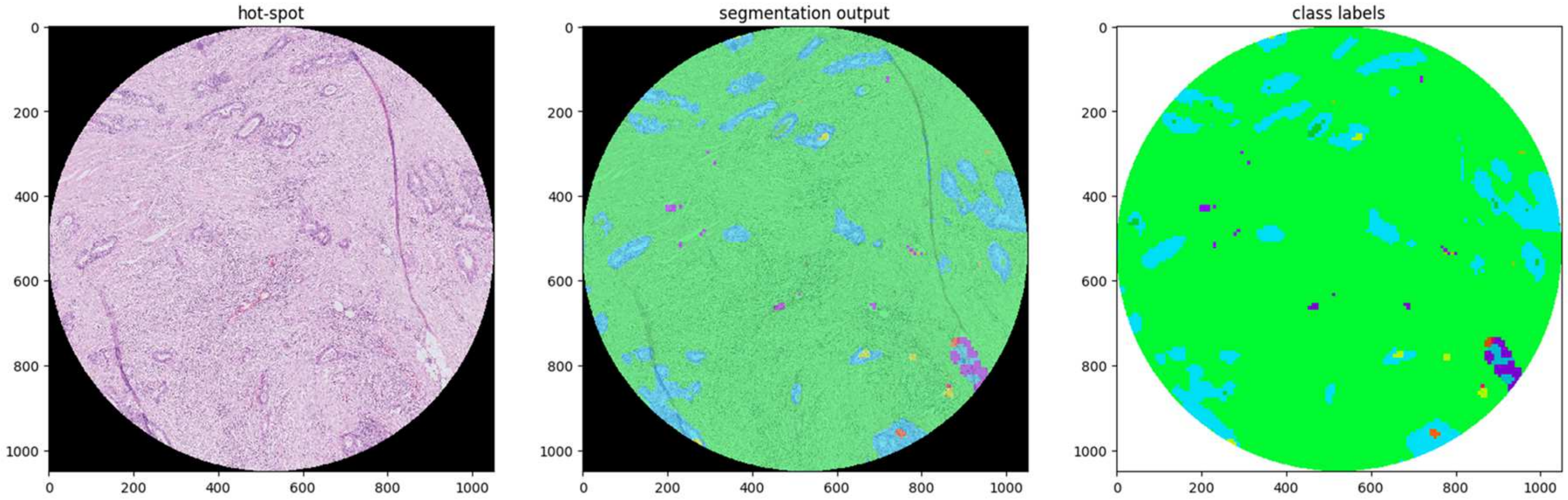

Fully-automated output

Top row; left: the tumor bulk is annotated. Right: heatmap is created. The biggest dot corresponds with the highest stroma-percentage (TSR-1), the second biggest with the second highest (TSR-2), etcetera.

Bottom row; left: the class output of the highest spot (TSR-1), middle: the second highest spot (TSR-2) and right the third highest spot (TSR-3)

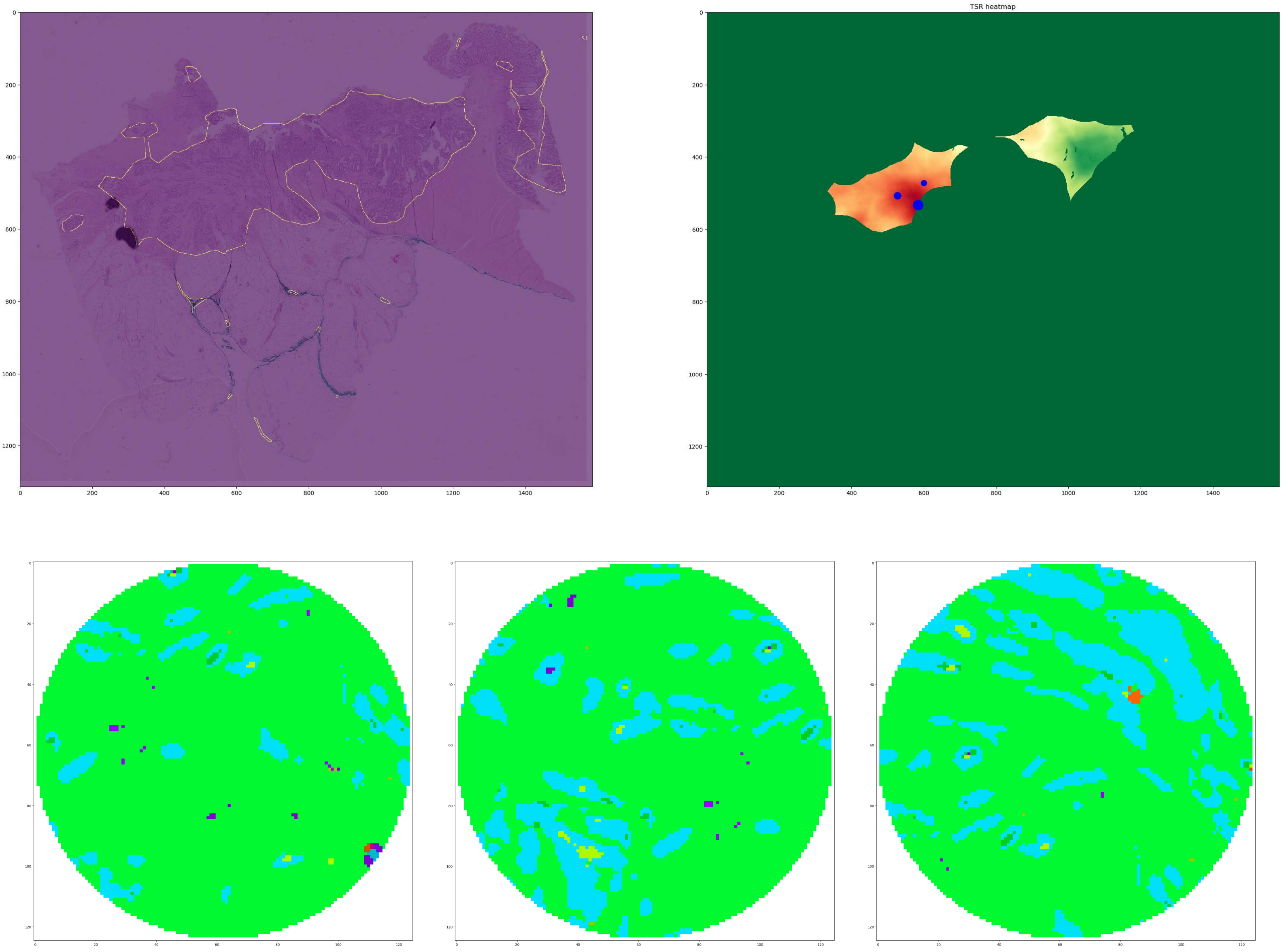

<u>Case 3</u>

Semi-automated output

Left: H&E stained section in the spot chosen by microscopic assessment. Middle: the first step was making an segmentation output. Right the class labels can be displayed

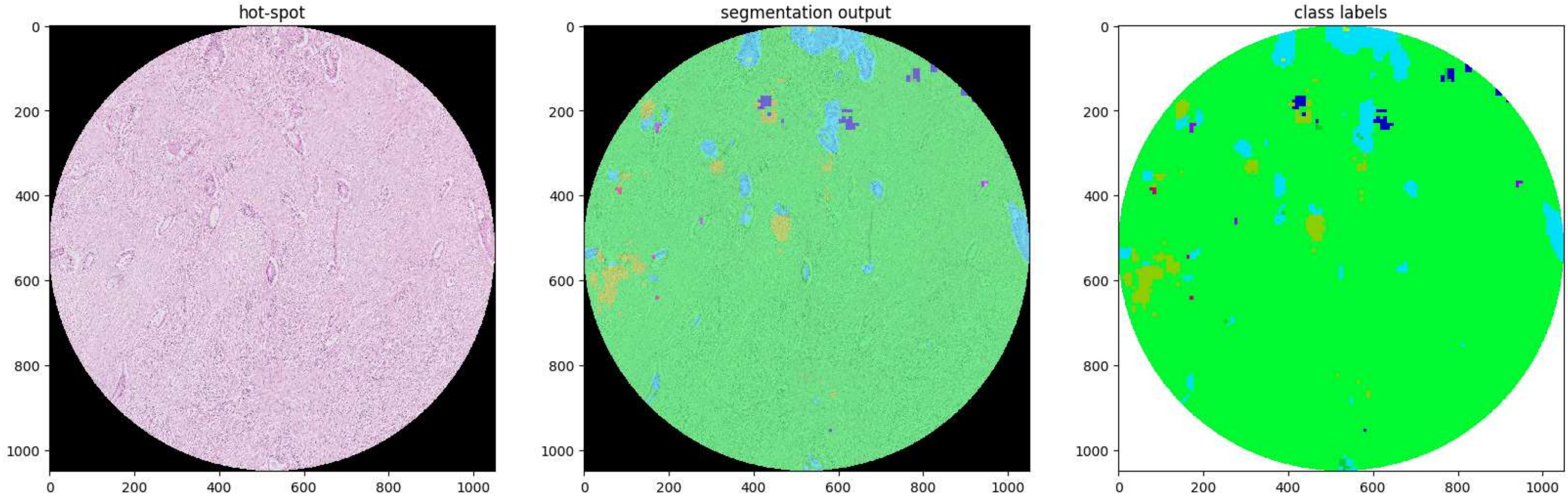

Fully-automated output

Top row; left: the tumor bulk is annotated. Right: heatmap is created. The biggest dot corresponds with the highest stroma-percentage (TSR-1), the second biggest with the second highest (TSR-2), etcetera.

Bottom row; left: the class output of the highest spot (TSR-1), middle: the second highest spot (TSR-2) and right the third highest spot (TSR-3)

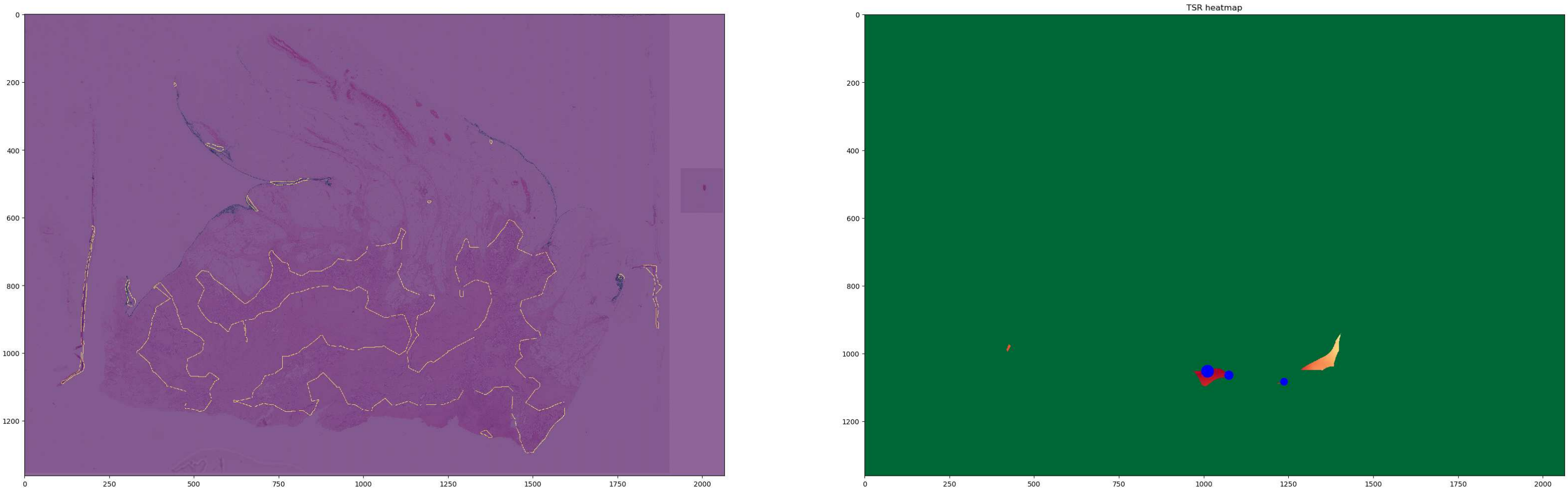

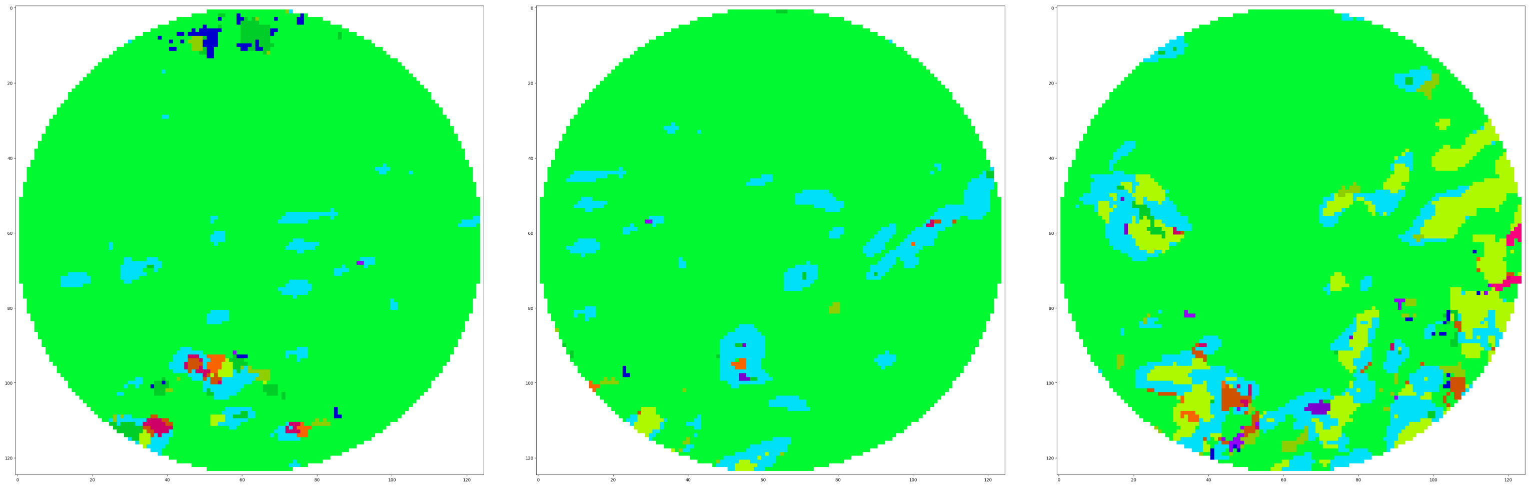

Case 4

Semi-automated output

Left: H&E stained section in the spot chosen by microscopic assessment. Middle: the first step was making an segmentation output. Right the class labels can be displayed

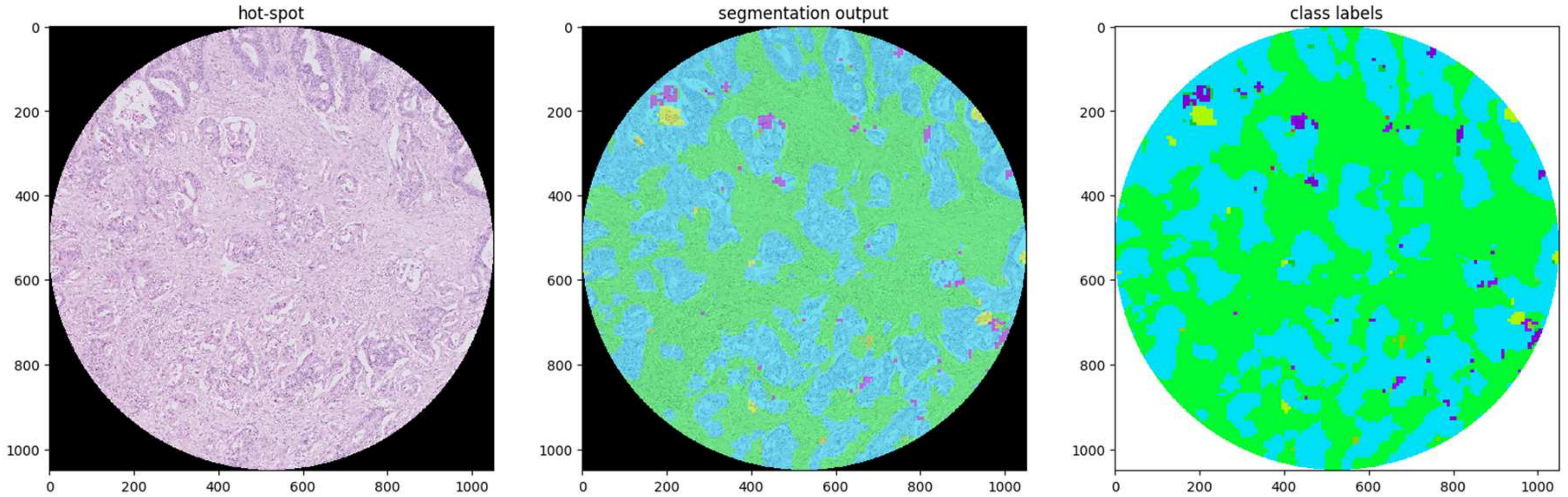

Fully-automated output

Top row; left: the tumor bulk is annotated. Right: heatmap is created. The biggest dot corresponds with the highest stroma-percentage (TSR-1), the second biggest with the second highest (TSR-2), etcetera.

Bottom row; left: the class output of the highest spot (TSR-1), middle: the second highest spot (TSR-2) and right the third highest spot (TSR-3)

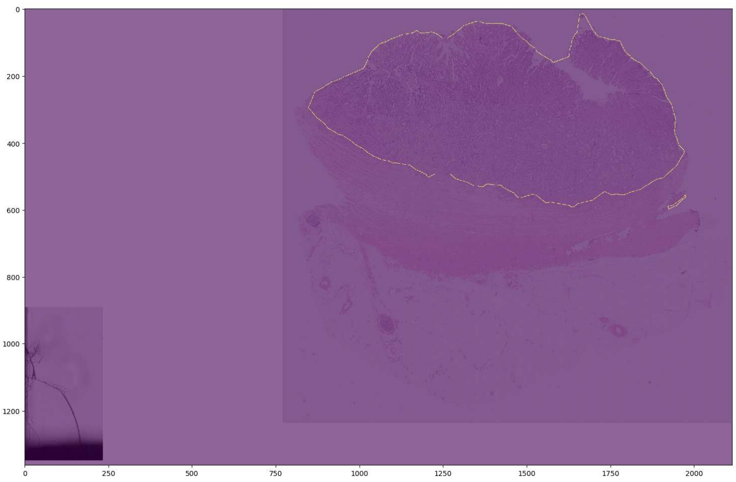

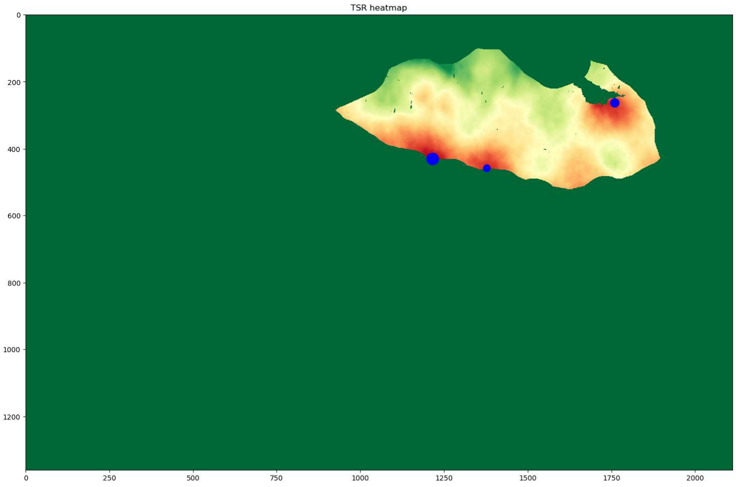

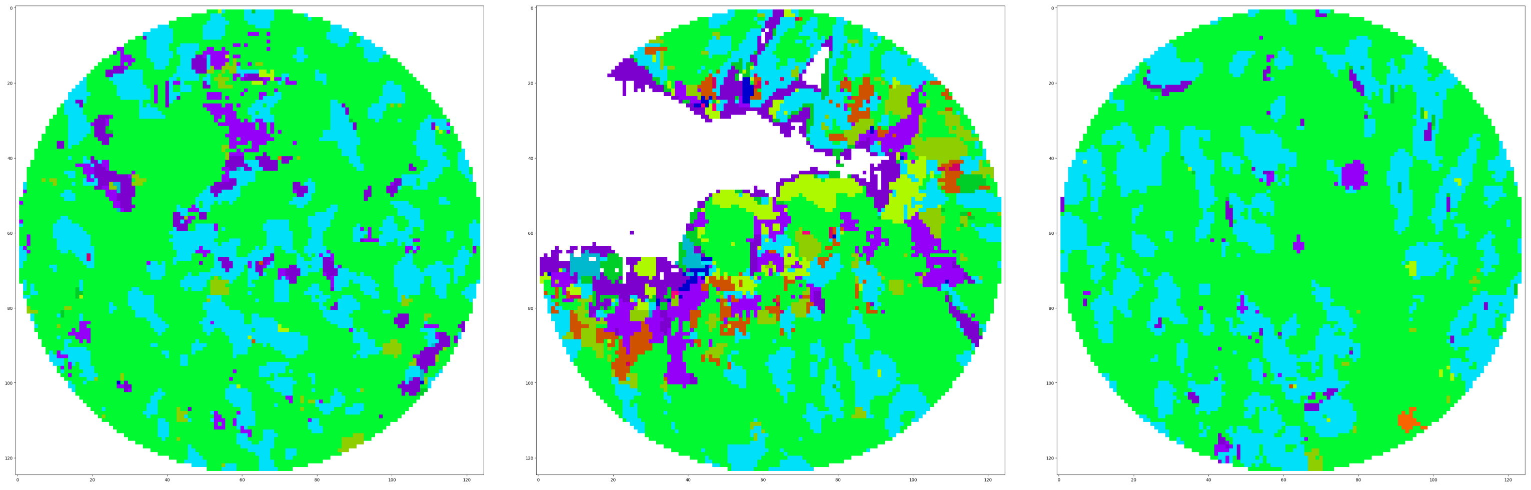

<u>Case 5</u>

Semi-automated output

Left: H&E stained section in the spot chosen by microscopic assessment. Middle: the first step was making an segmentation output. Right the class labels can be displayed

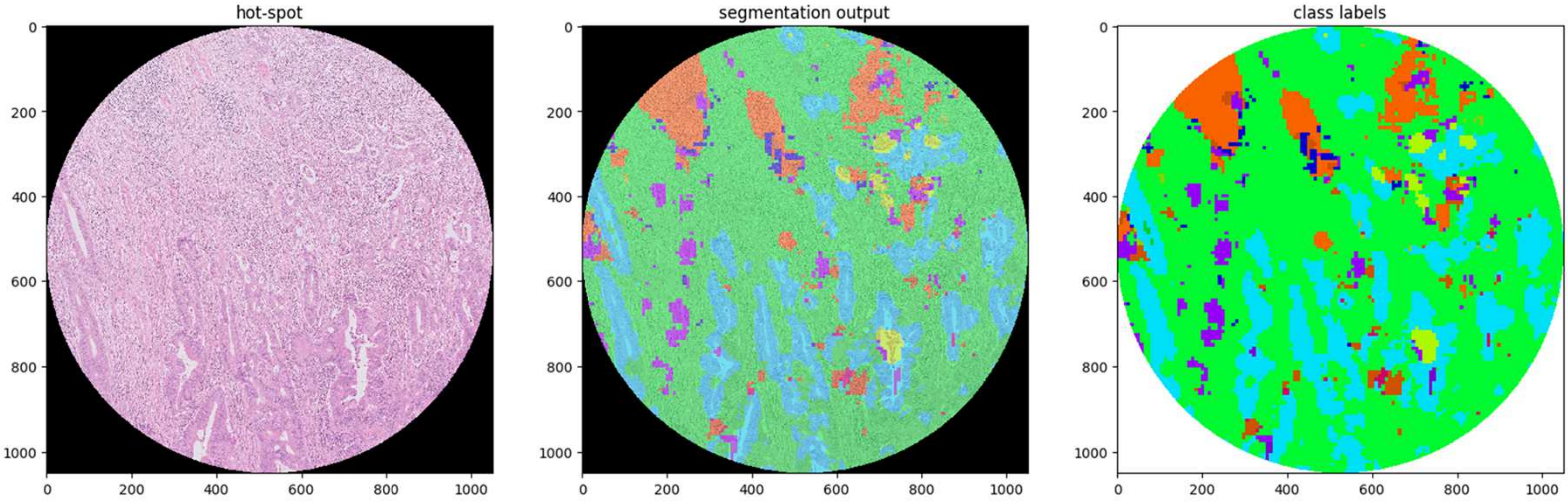

Fully-automated output

Top row; left: the tumor bulk is annotated. Right: heatmap is created. The biggest dot corresponds with the highest stroma-percentage (TSR-1), the second biggest with the second highest (TSR-2), etcetera.

Bottom row; left: the class output of the highest spot (TSR-1), middle: the second highest spot (TSR-2) and right the third highest spot (TSR-3)

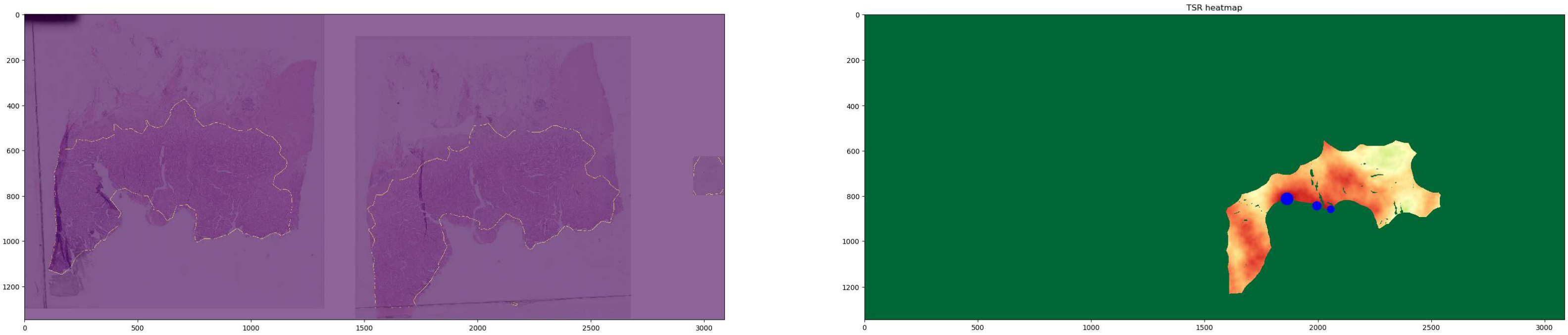

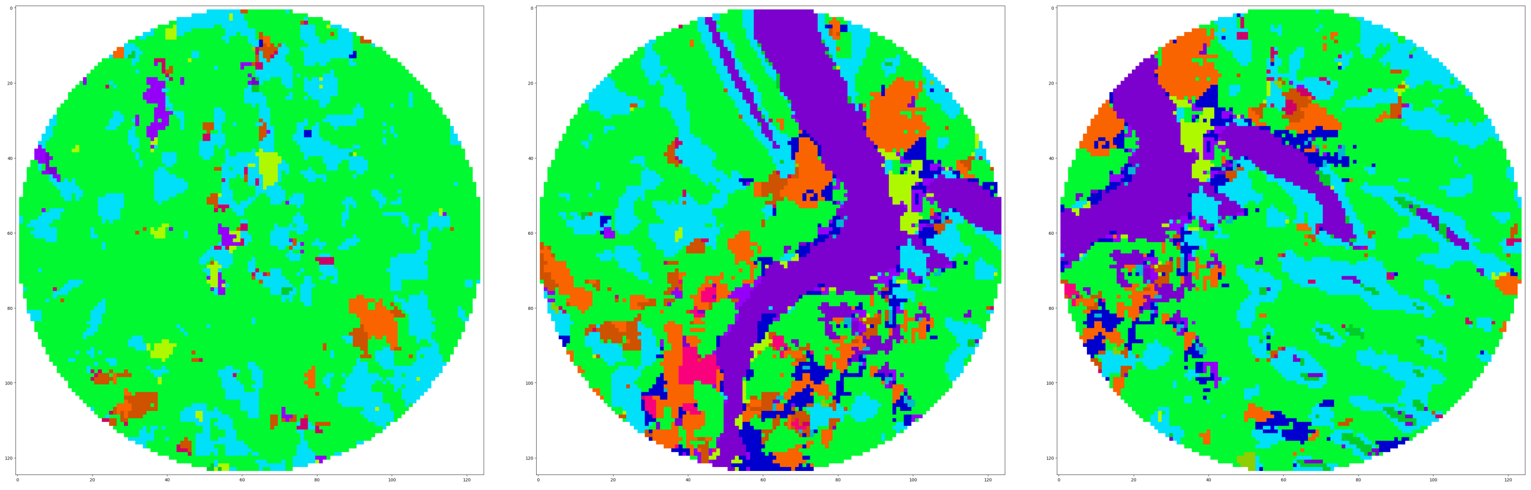

<u>Case 6</u>

Semi-automated output

Left: H&E stained section in the spot chosen by microscopic assessment. Middle: the first step was making an segmentation output. Right the class labels can be displayed

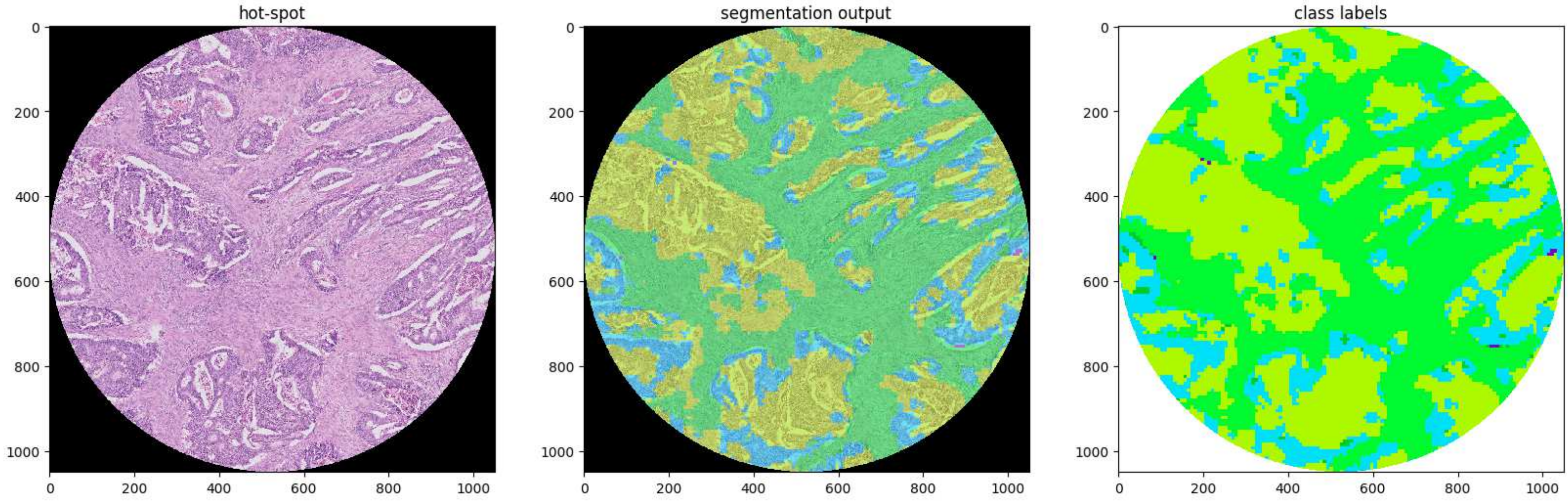

Fully-automated output

Top row; left: the tumor bulk is annotated. Right: heatmap is created. The biggest dot corresponds with the highest stroma-percentage (TSR-1), the second biggest with the second highest (TSR-2), etcetera.

Bottom row; left: the class output of the highest spot (TSR-1), middle: the second highest spot (TSR-2) and right the third highest spot (TSR-3)

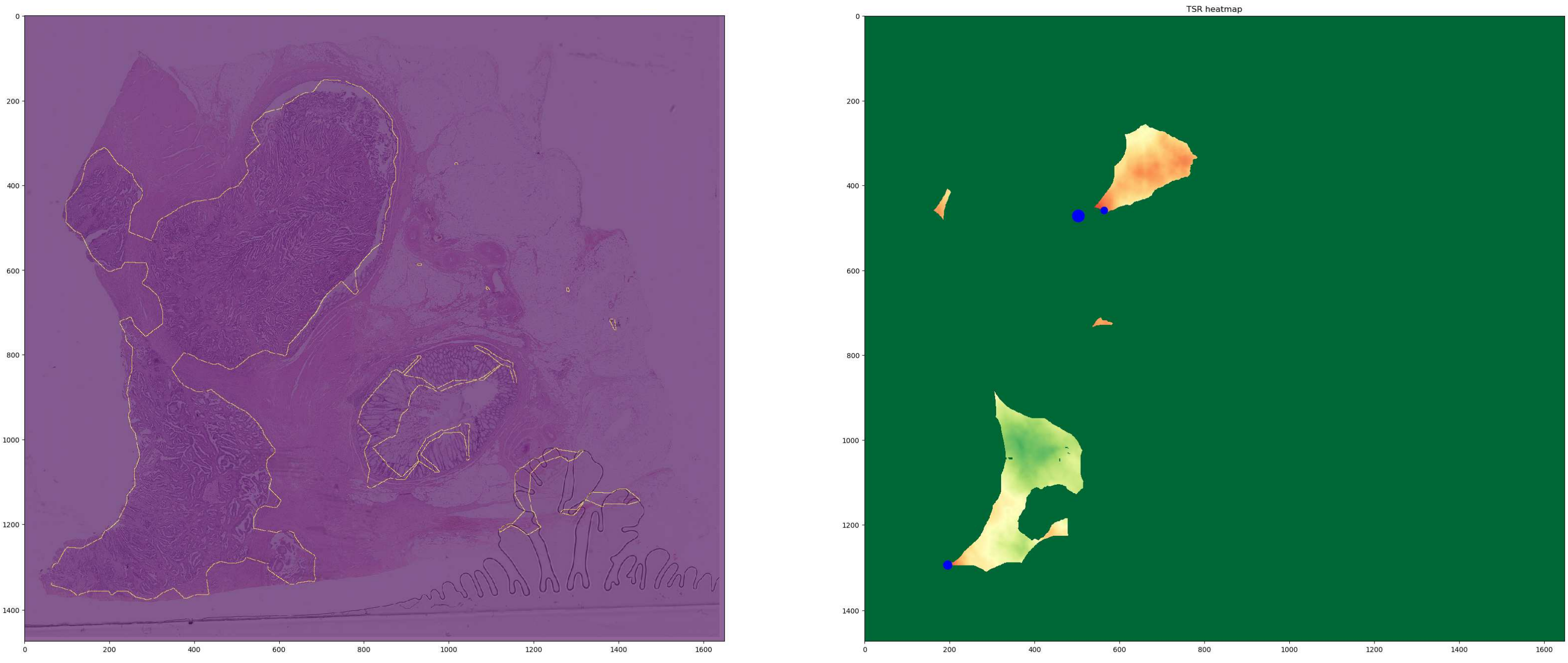

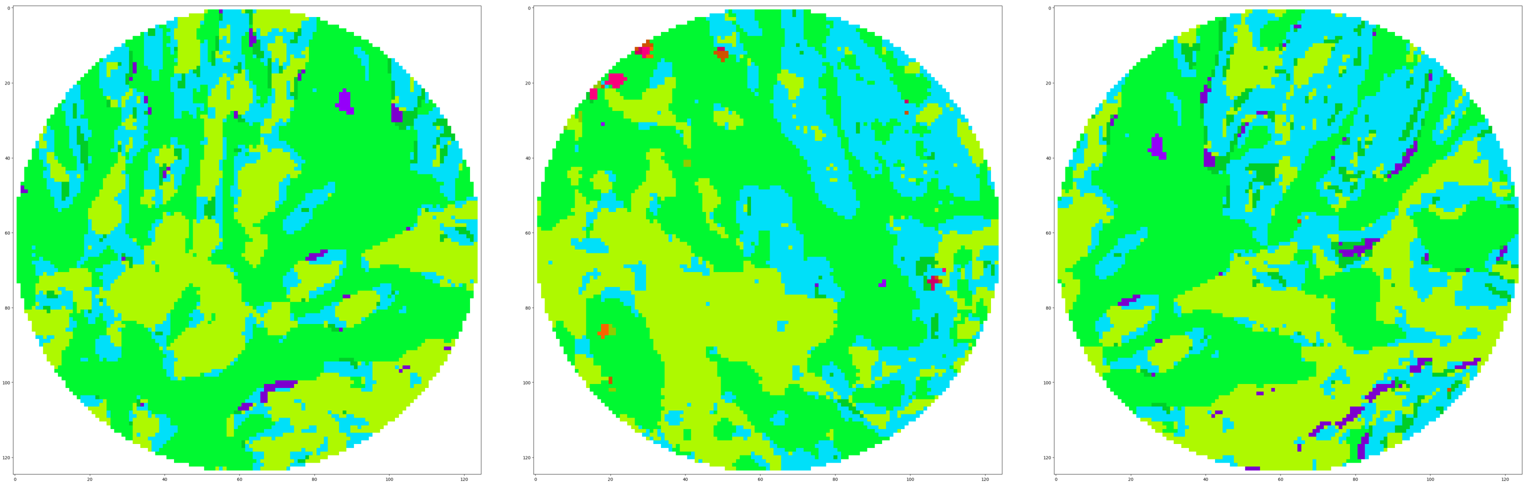

<u>Case 7</u>

Semi-automated output

Left: H&E stained section in the spot chosen by microscopic assessment. Middle: the first step was making an segmentation output. Right the class labels can be displayed

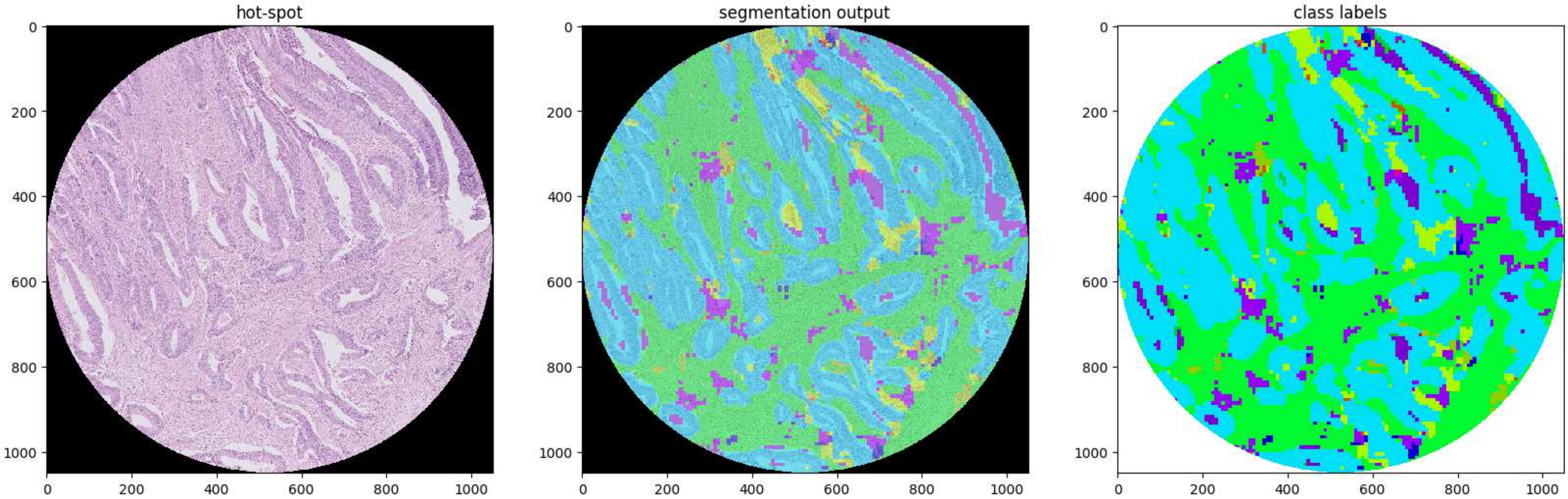

Fully-automated output

Top row; left: the tumor bulk is annotated. Right: heatmap is created. The biggest dot corresponds with the highest stroma-percentage (TSR-1), the second biggest with the second highest (TSR-2), etcetera.

Bottom row; left: the class output of the highest spot (TSR-1), middle: the second highest spot (TSR-2) and right the third highest spot (TSR-3)

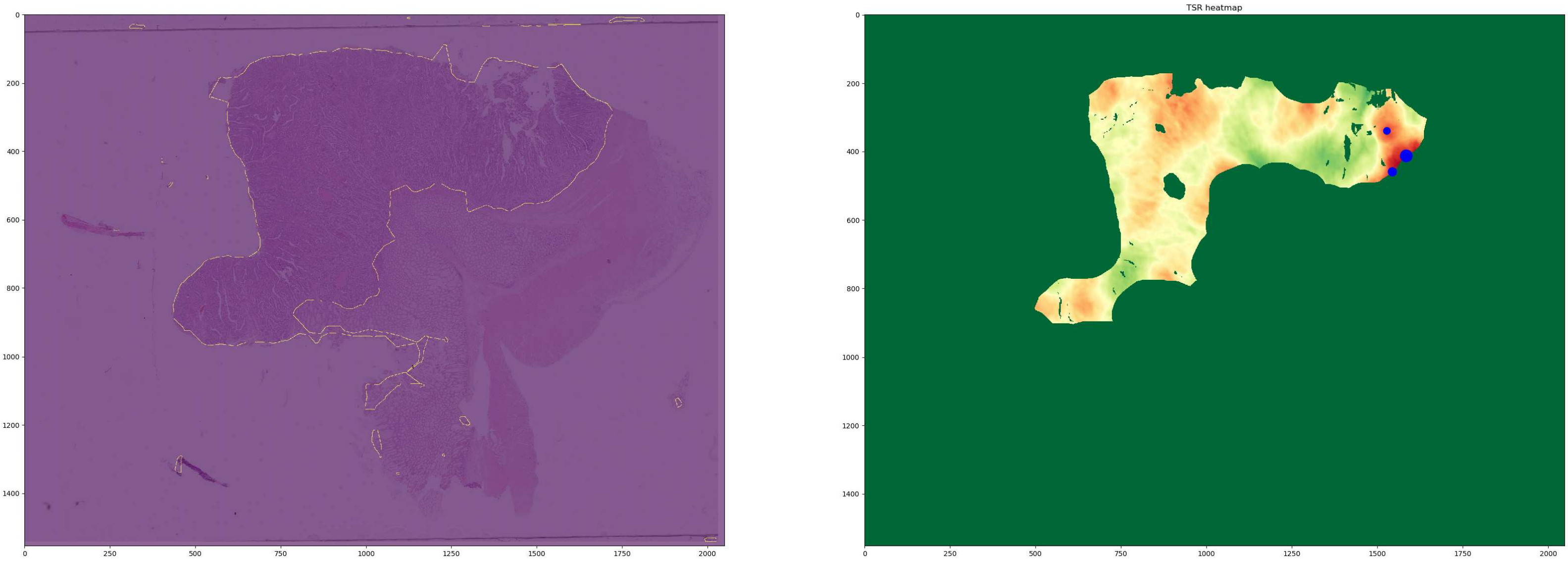

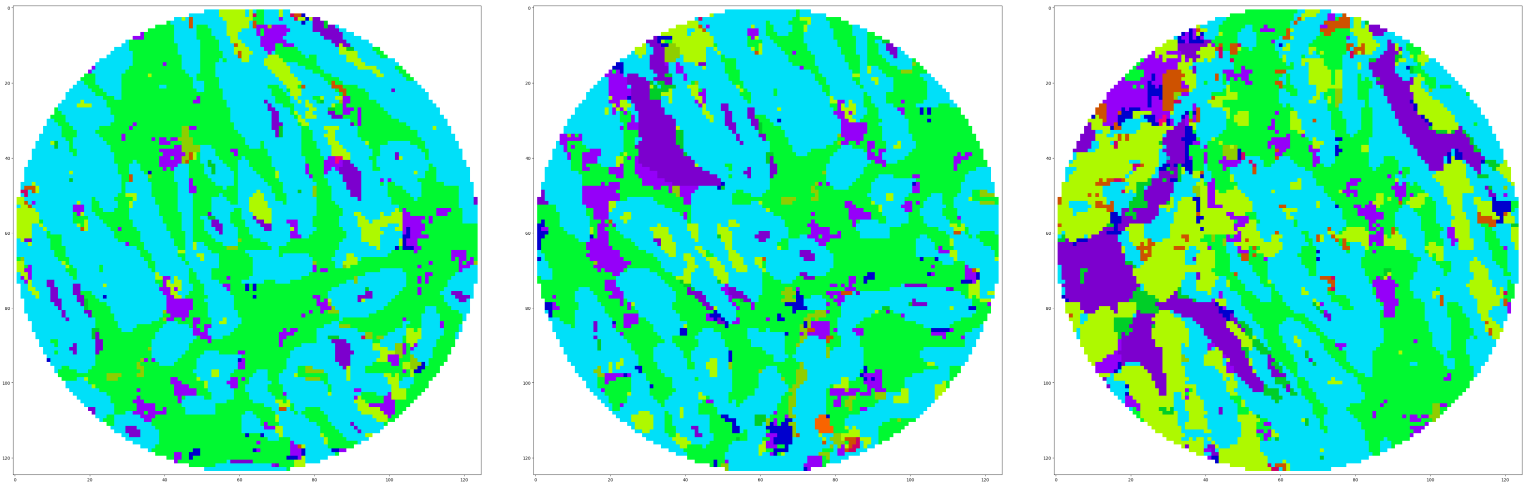

<u>Case 8</u>

Semi-automated output

Left: H&E stained section in the spot chosen by microscopic assessment. Middle: the first step was making an segmentation output. Right the class labels can be displayed

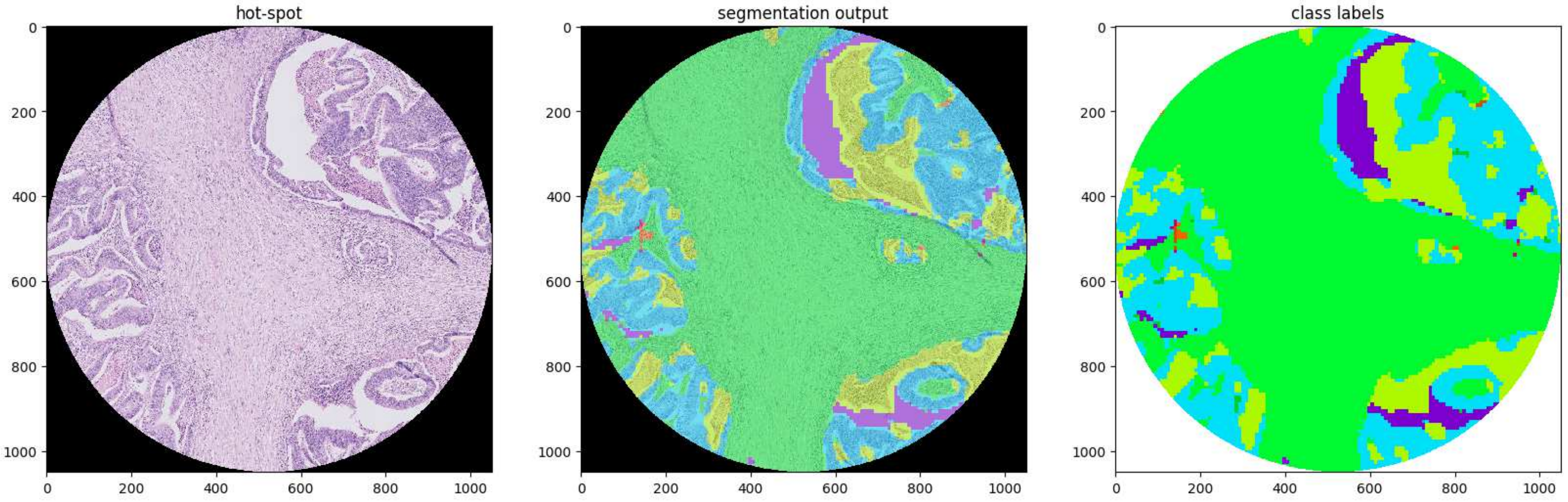

Fully-automated output

Top row; left: the tumor bulk is annotated. Right: heatmap is created. The biggest dot corresponds with the highest stroma-percentage (TSR-1), the second biggest with the second highest (TSR-2), etcetera.

Bottom row; left: the class output of the highest spot (TSR-1), middle: the second highest spot (TSR-2) and right the third highest spot (TSR-3)

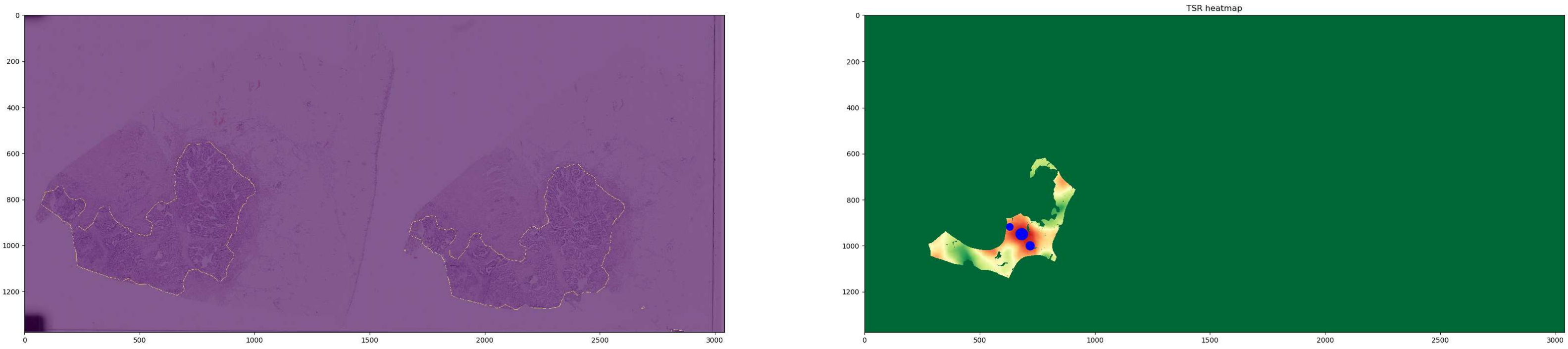

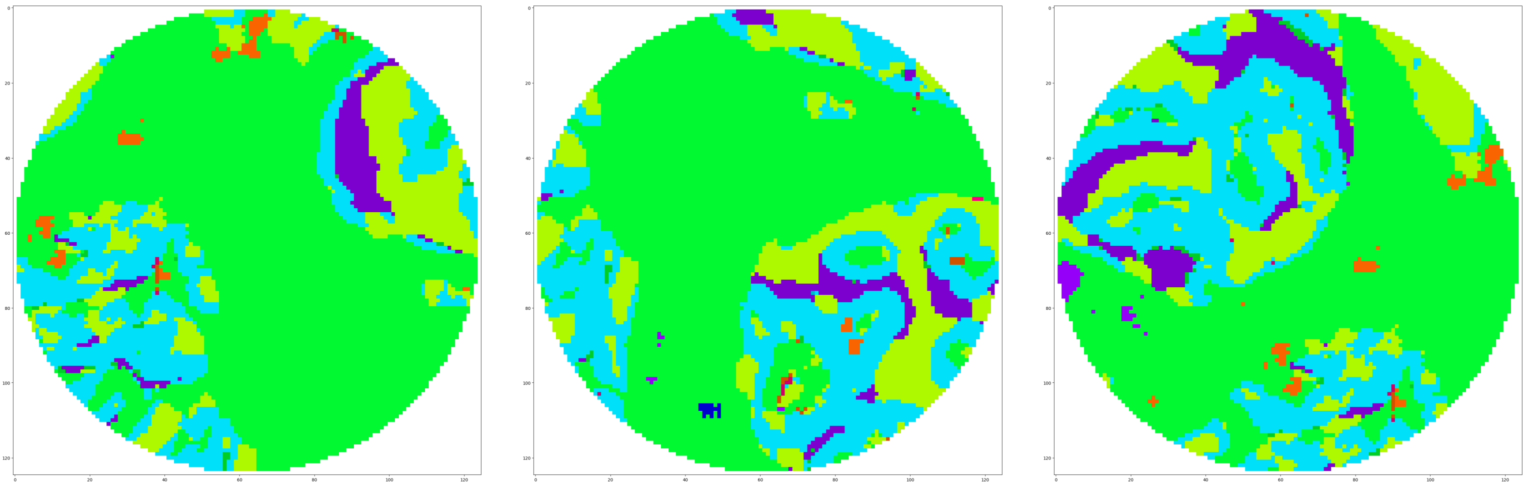

<u>Case 9</u>

Semi-automated output

Left: H&E stained section in the spot chosen by microscopic assessment. Middle: the first step was making an segmentation output. Right the class labels can be displayed

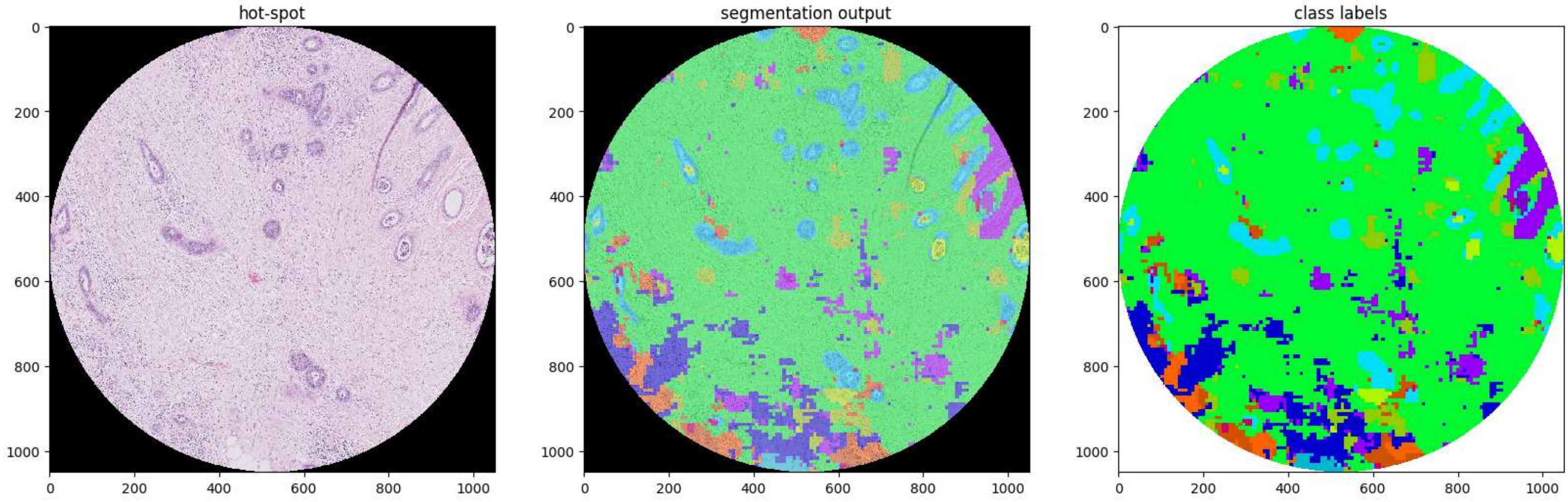

Fully-automated output

Top row; left: the tumor bulk is annotated. Right: heatmap is created. The biggest dot corresponds with the highest stroma-percentage (TSR-1), the second biggest with the second highest (TSR-2), etcetera.

Bottom row; left: the class output of the highest spot (TSR-1), middle: the second highest spot (TSR-2) and right the third highest spot (TSR-3)

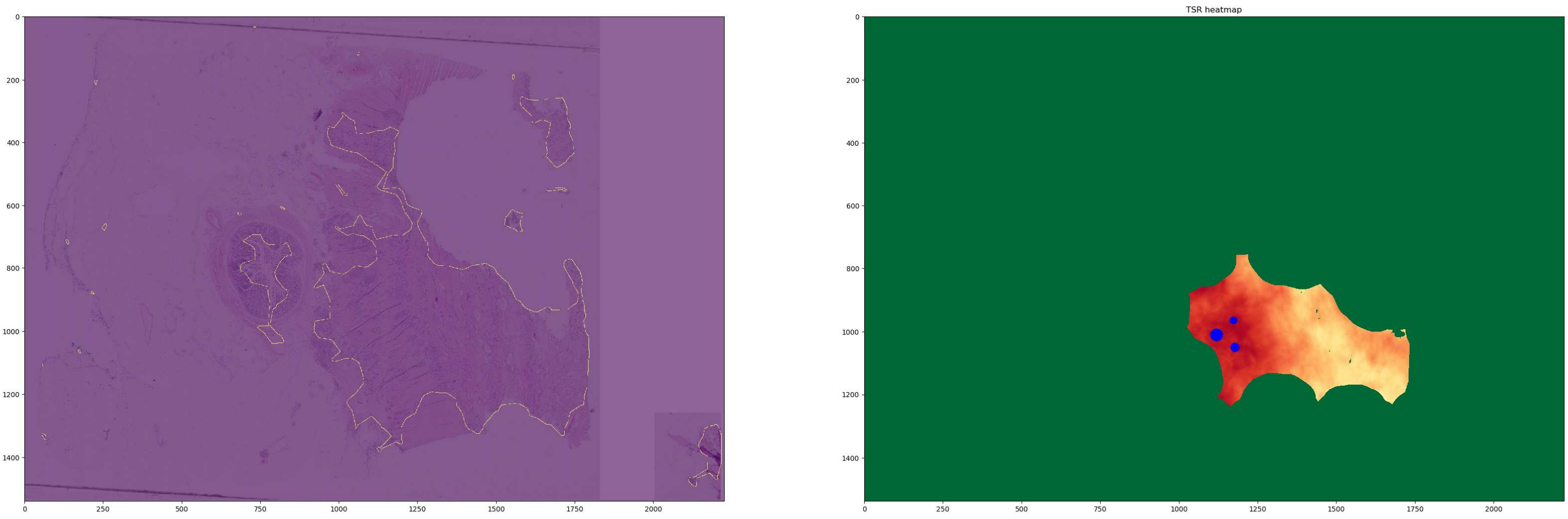

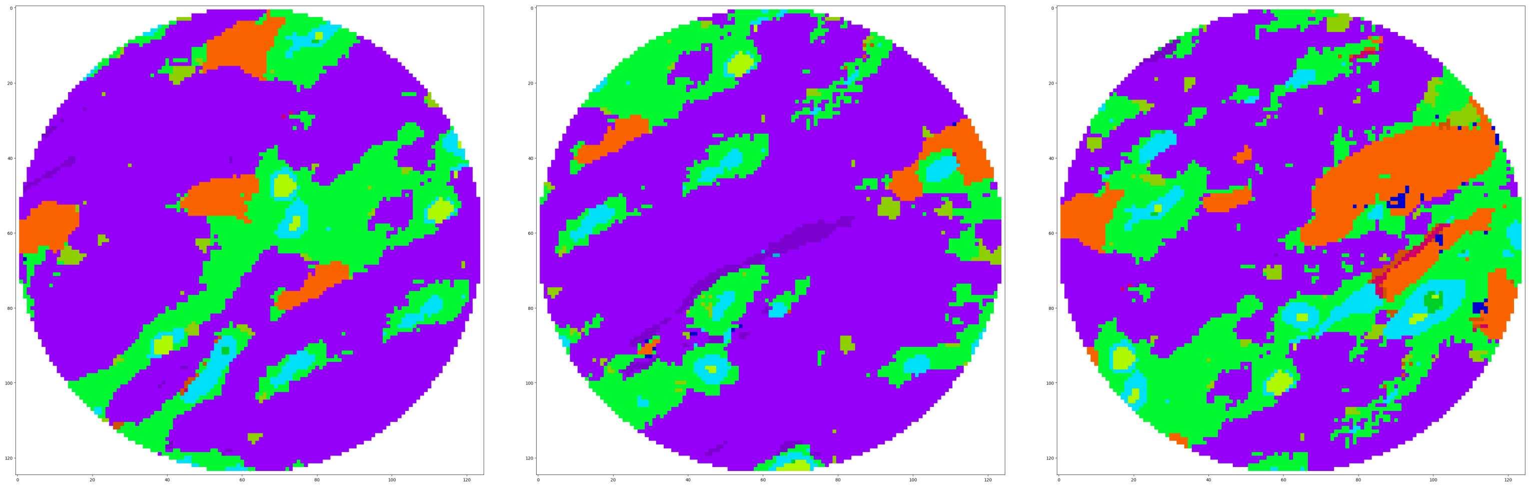

Case 10

Semi-automated output

Left: H&E stained section in the spot chosen by microscopic assessment. Middle: the first step was making an segmentation output. Right the class labels can be displayed

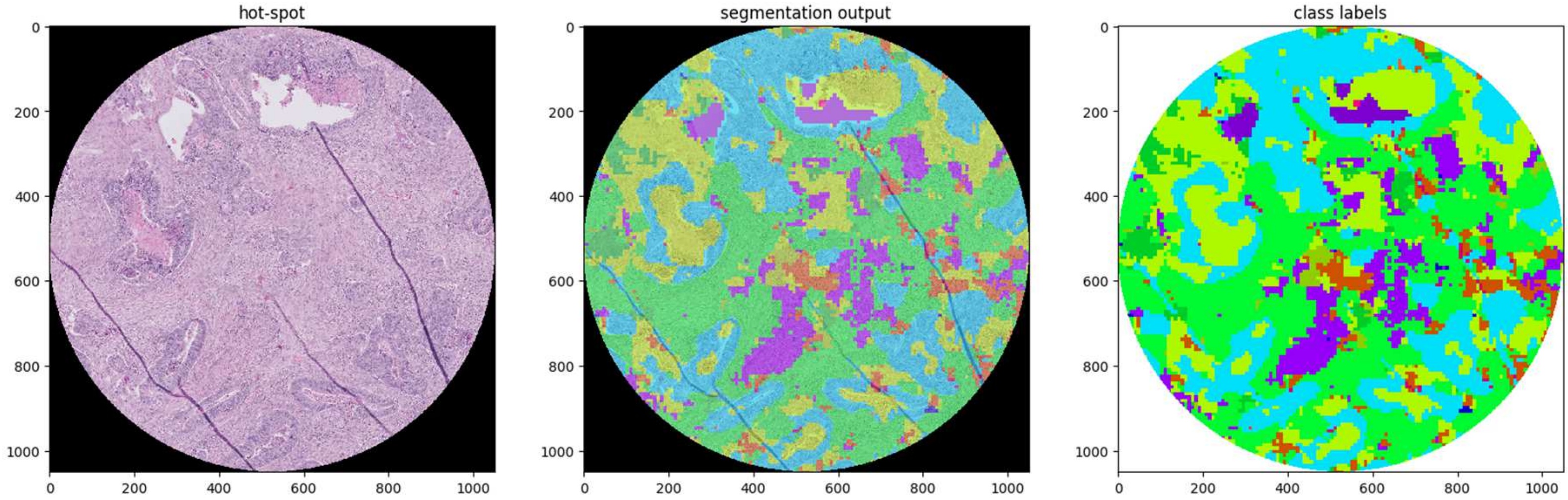

Fully-automated output

Top row; left: the tumor bulk is annotated. Right: heatmap is created. The biggest dot corresponds with the highest stroma-percentage (TSR-1), the second biggest with the second highest (TSR-2), etcetera.

Bottom row; left: the class output of the highest spot (TSR-1), middle: the second highest spot (TSR-2) and right the third highest spot (TSR-3)

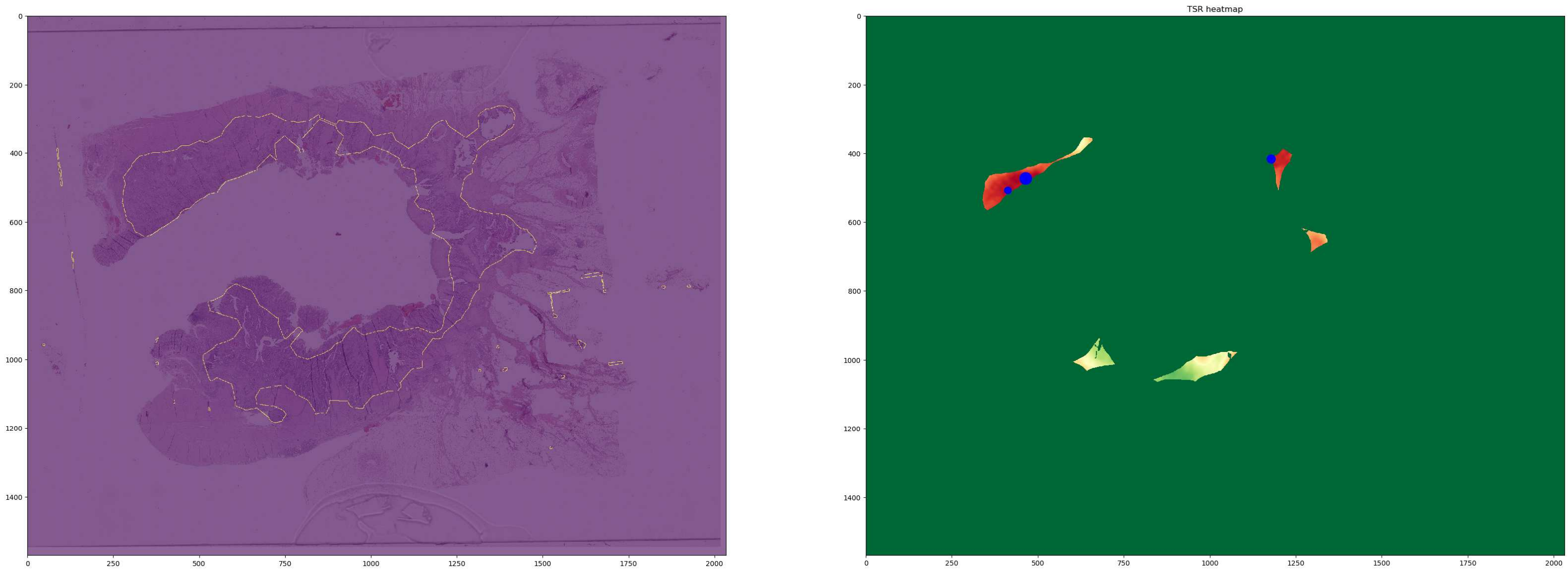

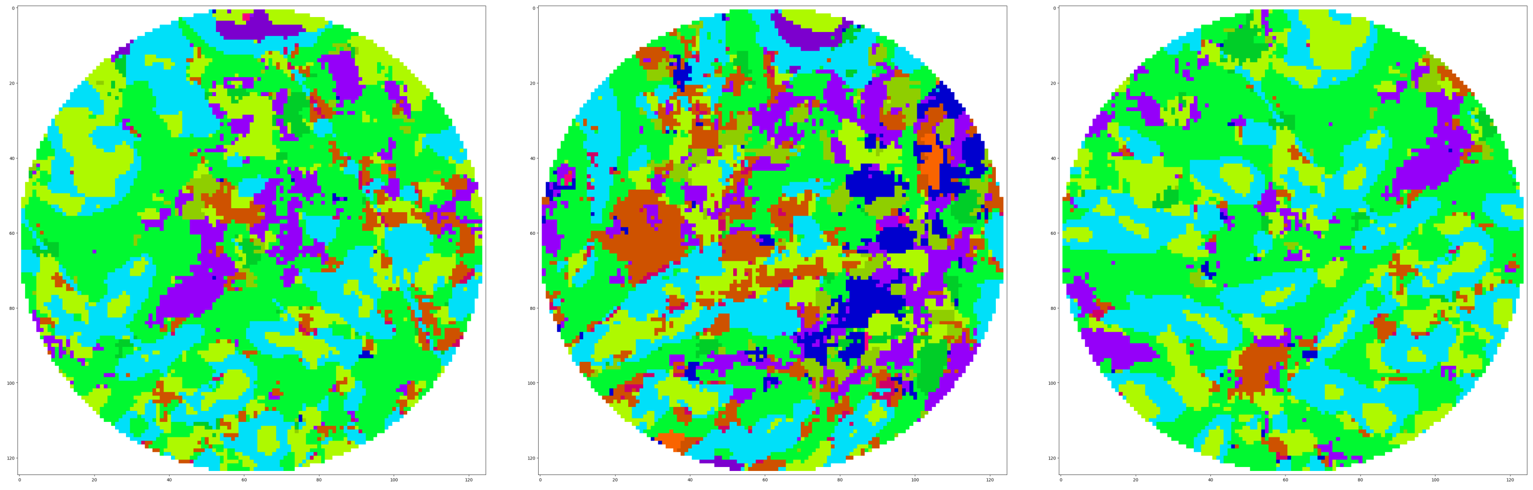

Case 11

Semi-automated output

Left: H&E stained section in the spot chosen by microscopic assessment. Middle: the first step was making an segmentation output. Right the class labels can be displayed

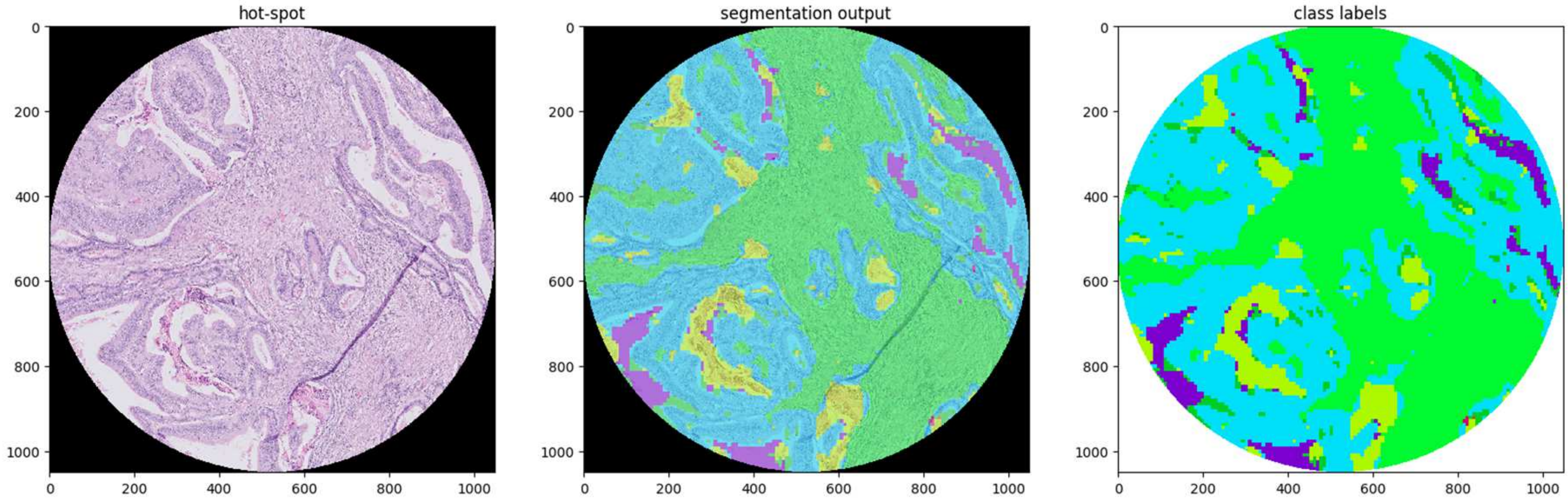

Fully-automated output

Top row; left: the tumor bulk is annotated. Right: heatmap is created. The biggest dot corresponds with the highest stroma-percentage (TSR-1), the second biggest with the second highest (TSR-2), etcetera.

Bottom row; left: the class output of the highest spot (TSR-1), middle: the second highest spot (TSR-2) and right the third highest spot (TSR-3)

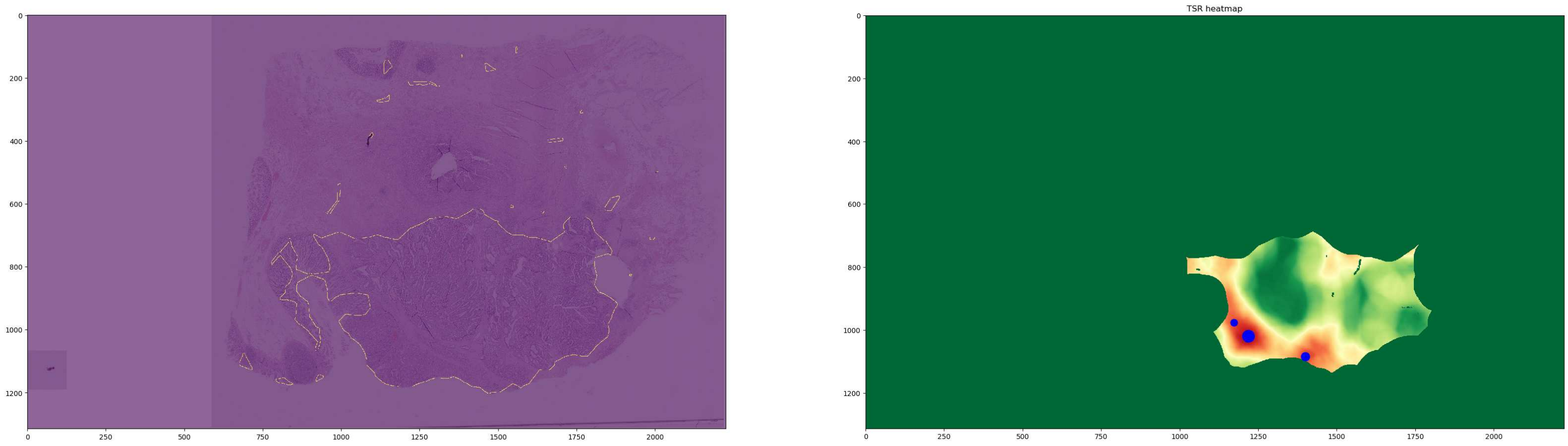

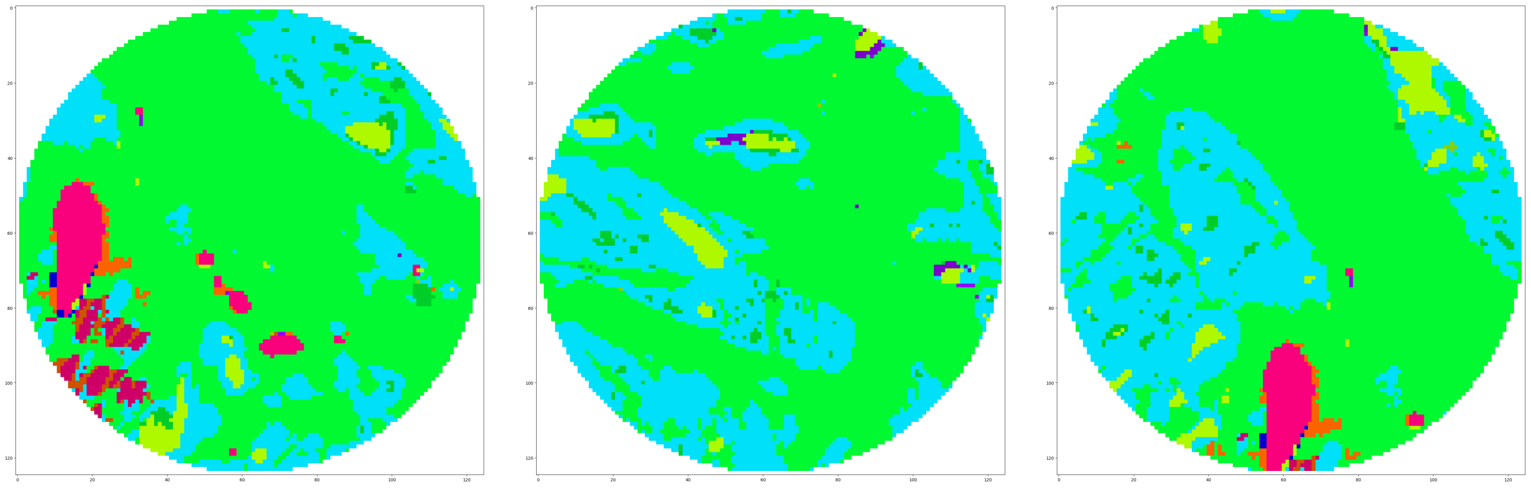

Case 12

Semi-automated output

Left: H&E stained section in the spot chosen by microscopic assessment. Middle: the first step was making an segmentation output. Right the class labels can be displayed

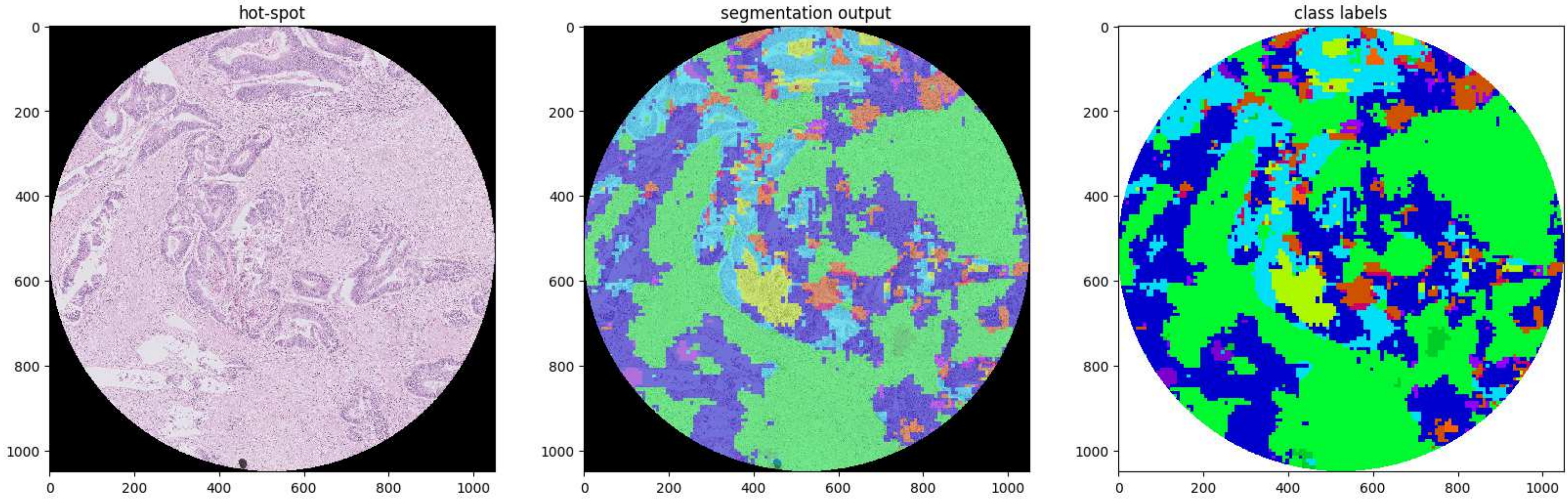

Fully-automated output

Top row; left: the tumor bulk is annotated. Right: heatmap is created. The biggest dot corresponds with the highest stroma-percentage (TSR-1), the second biggest with the second highest (TSR-2), etcetera.
Bottom row; left: the class output of the highest spot (TSR-1), middle: the second highest spot (TSR-2) and right the third highest spot (TSR-3)

- According to the rules used for fully-automated TSR scoring, the tumor bulk was not big enough and so no output was generated

Case 13

Semi-automated output

Left: H&E stained section in the spot chosen by microscopic assessment. Middle: the first step was making an segmentation output. Right the class labels can be displayed

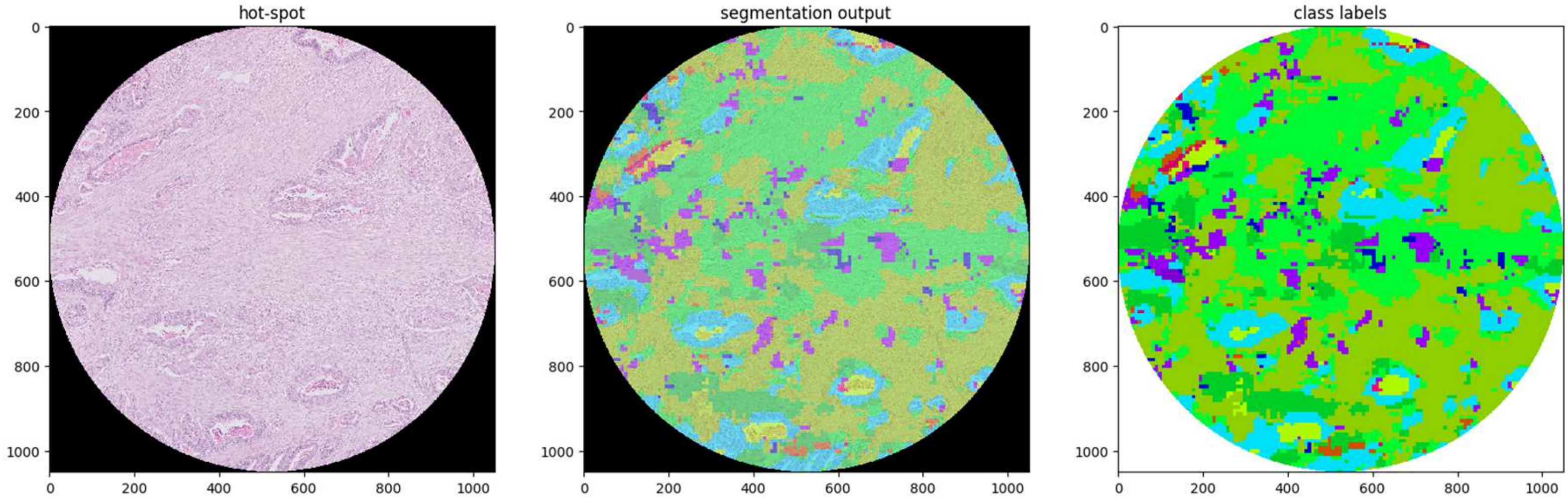

Fully-automated output

Top row; left: the tumor bulk is annotated. Right: heatmap is created. The biggest dot corresponds with the highest stroma-percentage (TSR-1), the second biggest with the second highest (TSR-2), etcetera.

Bottom row; left: the class output of the highest spot (TSR-1), middle: the second highest spot (TSR-2) and right the third highest spot (TSR-3)

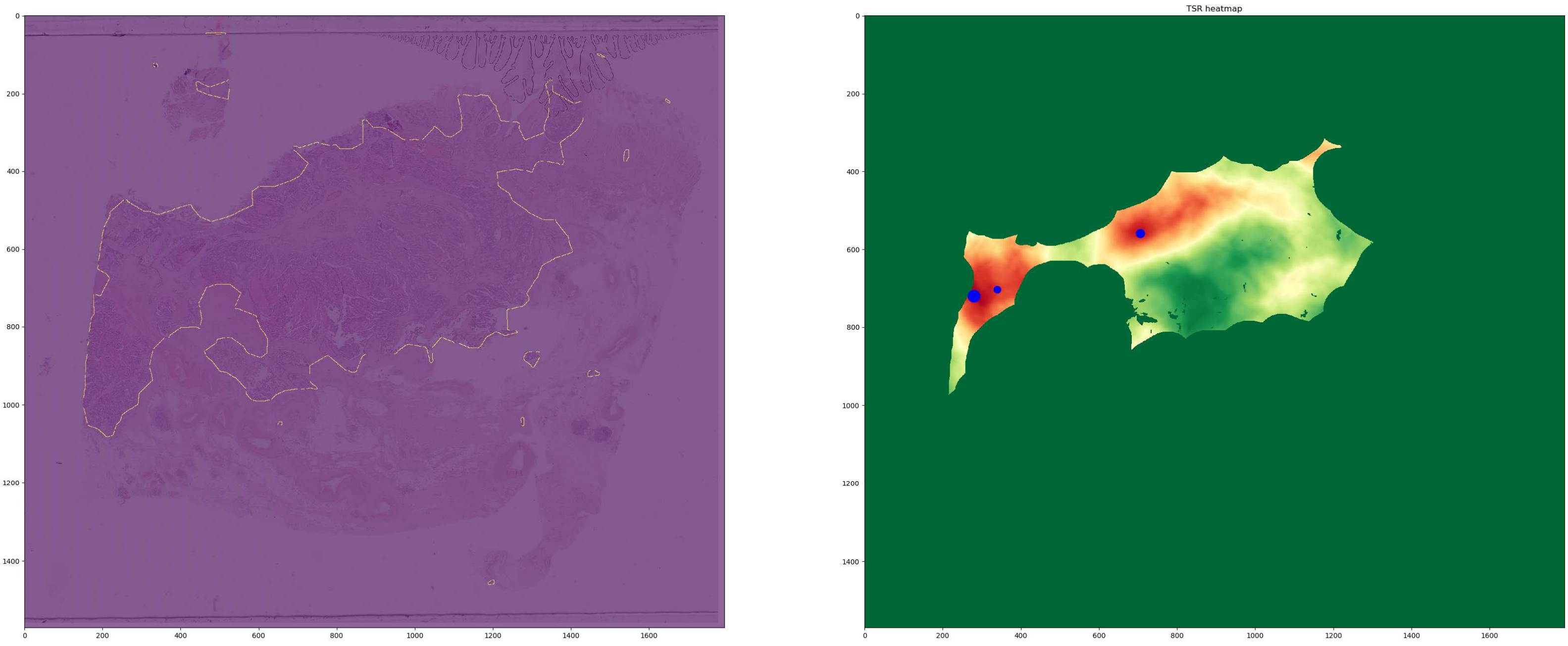

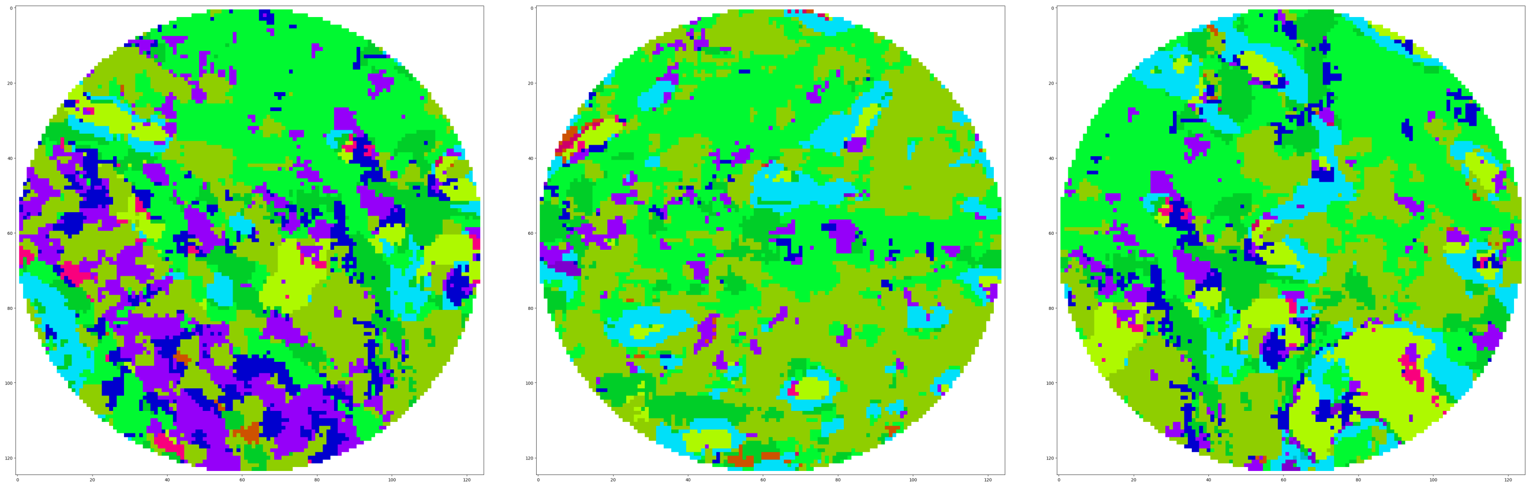

Case 14

Semi-automated output

Left: H&E stained section in the spot chosen by microscopic assessment. Middle: the first step was making an segmentation output. Right the class labels can be displayed

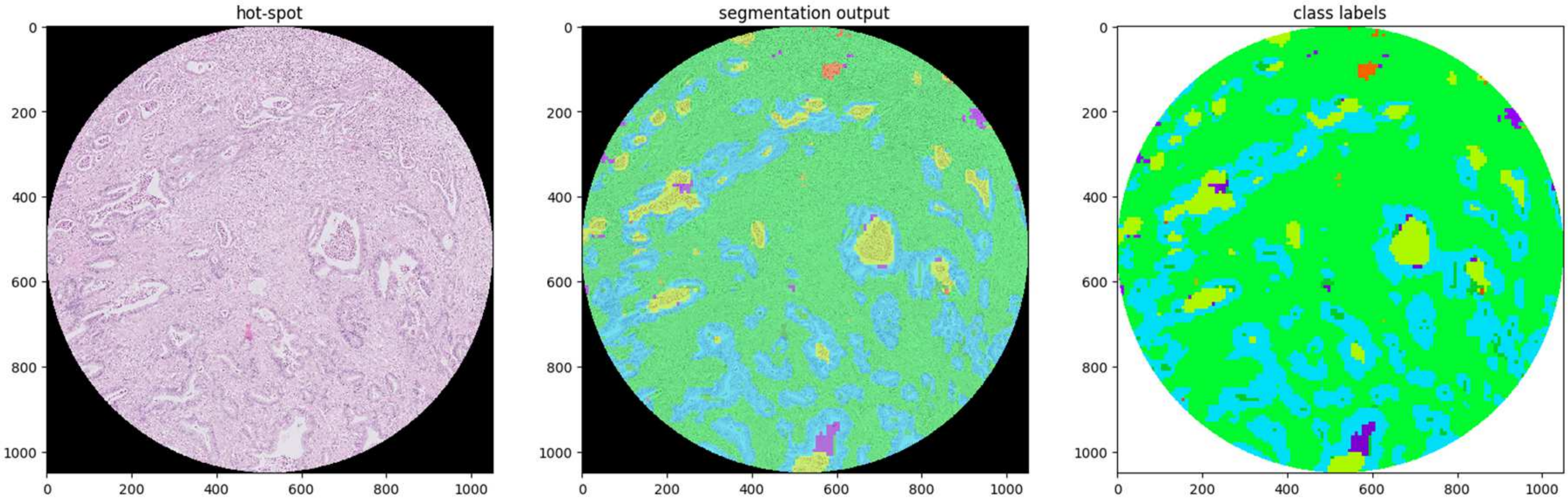

Fully-automated output

Top row; left: the tumor bulk is annotated. Right: heatmap is created. The biggest dot corresponds with the highest stroma-percentage (TSR-1), the second biggest with the second highest (TSR-2), etcetera.

Bottom row; left: the class output of the highest spot (TSR-1), middle: the second highest spot (TSR-2) and right the third highest spot (TSR-3)

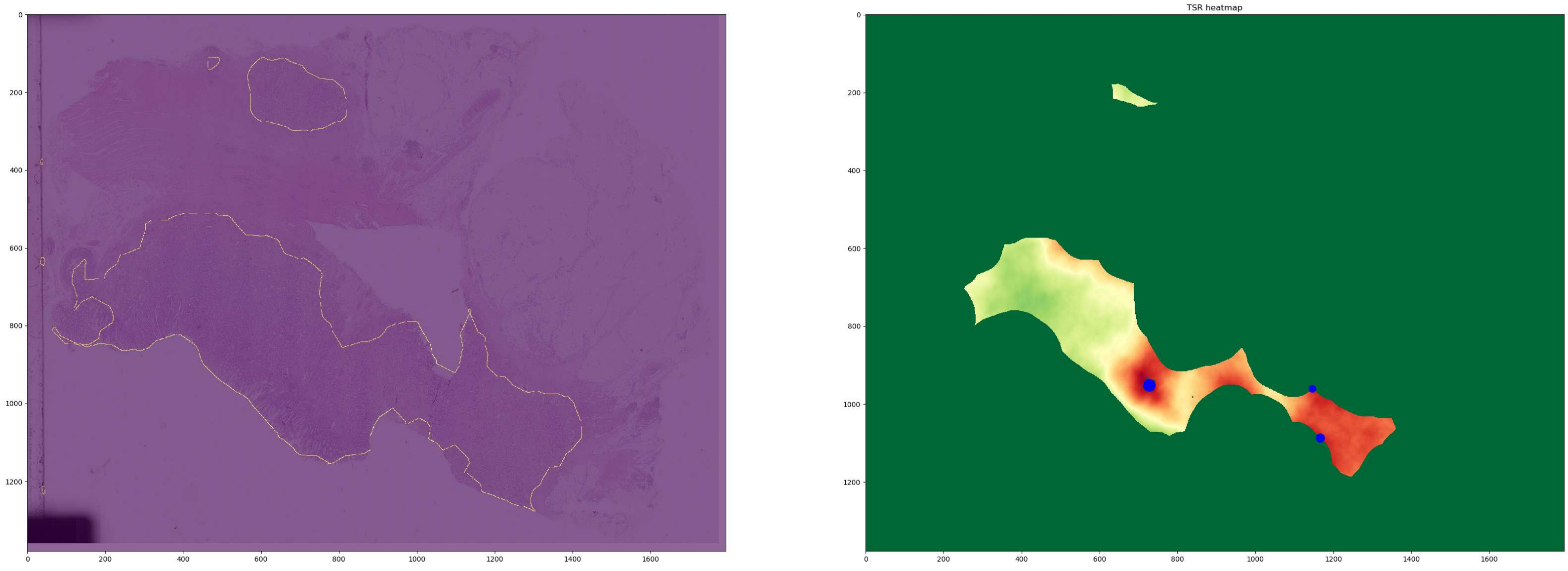

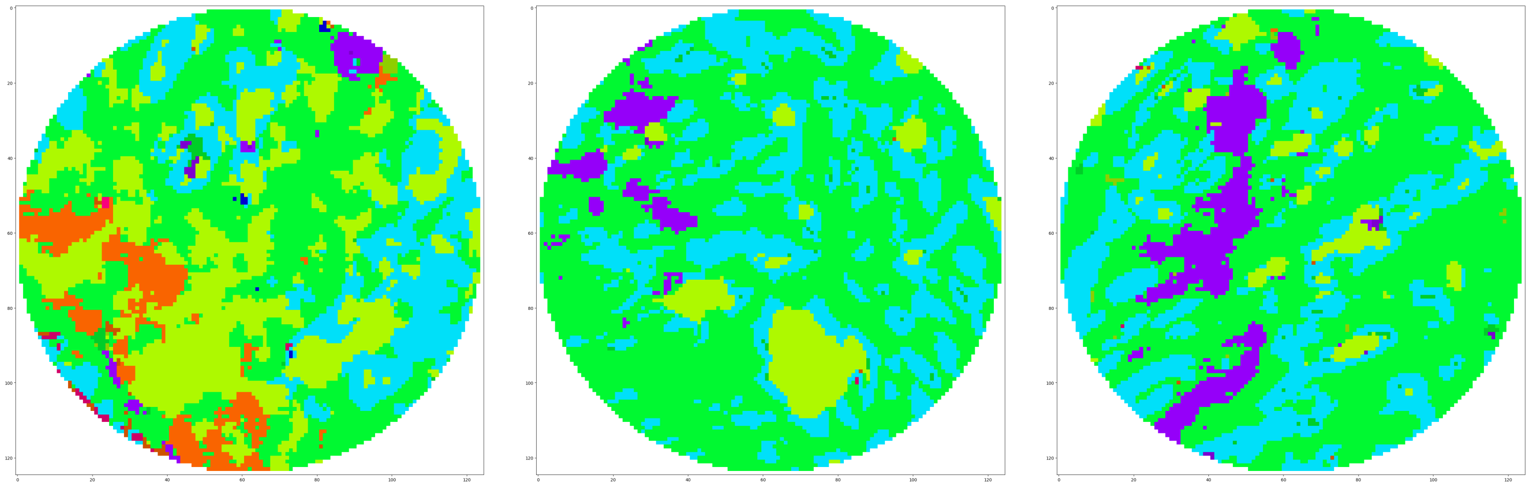

Case 15

Semi-automated output

Left: H&E stained section in the spot chosen by microscopic assessment. Middle: the first step was making an segmentation output. Right the class labels can be displayed

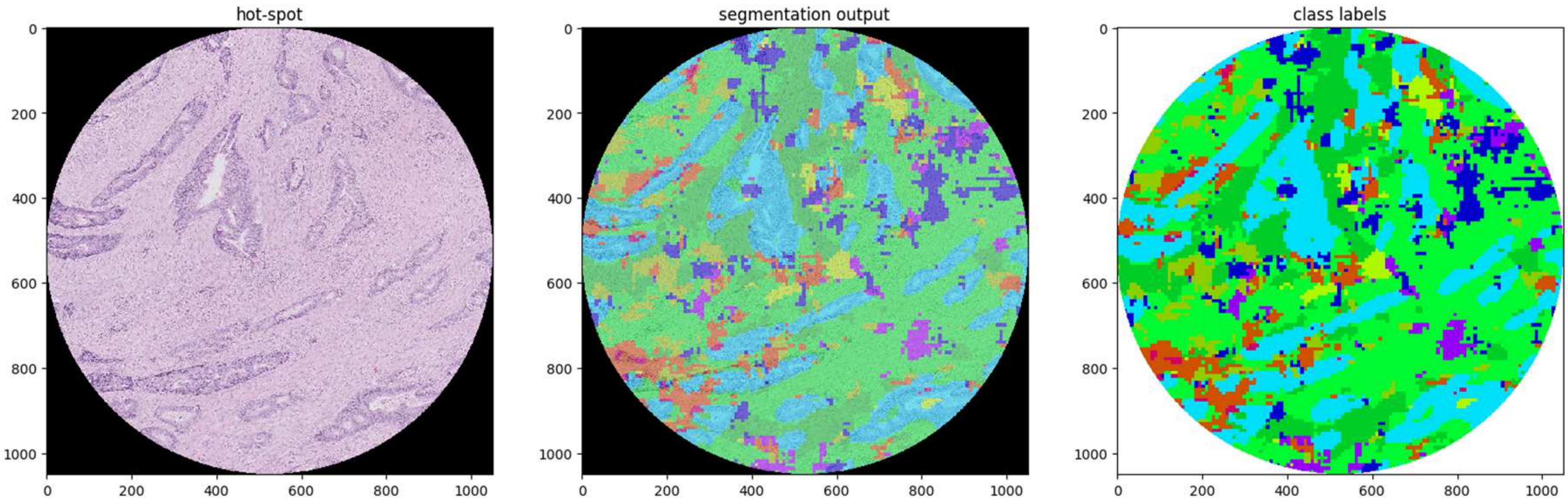

Fully-automated output

Top row; left: the tumor bulk is annotated. Right: heatmap is created. The biggest dot corresponds with the highest stroma-percentage (TSR-1), the second biggest with the second highest (TSR-2), etcetera.
Bottom row; left: the class output of the highest spot (TSR-1), middle: the second highest spot (TSR-2) and right the third highest spot (TSR-3)

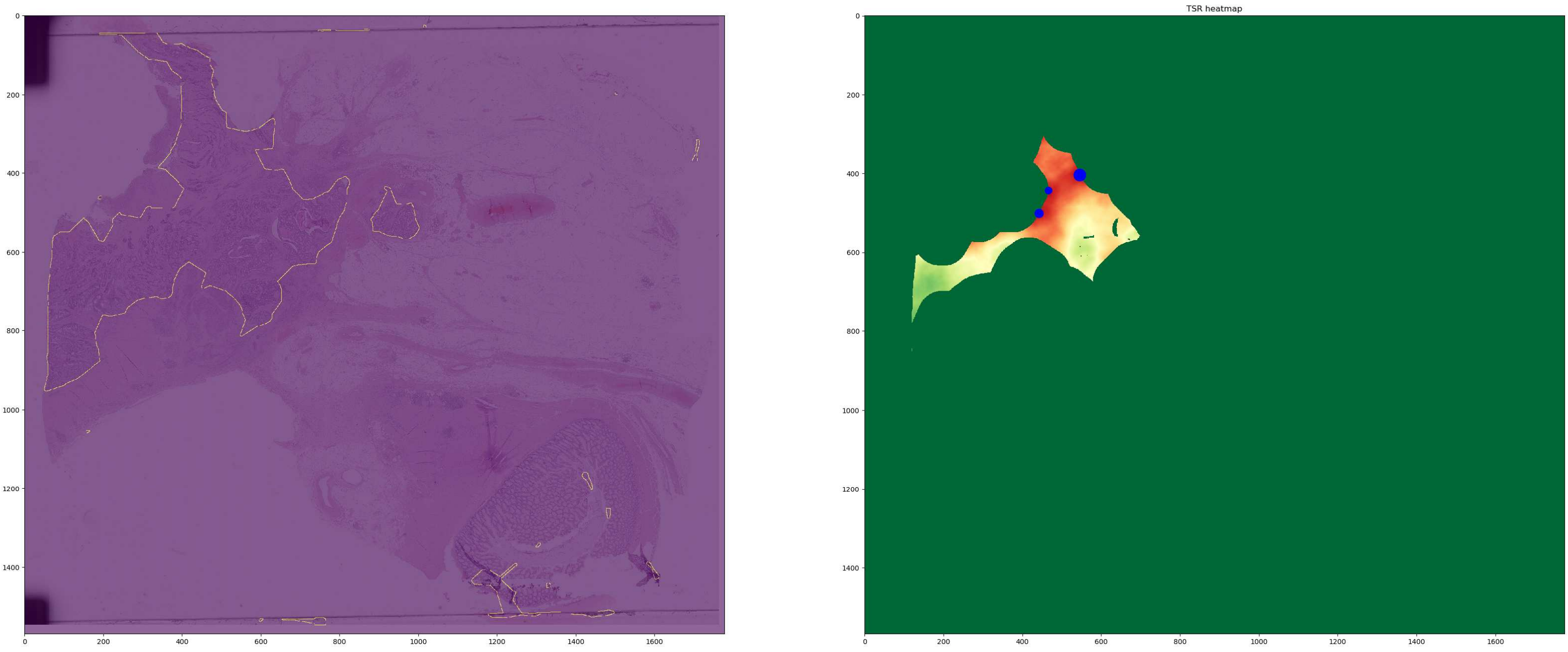

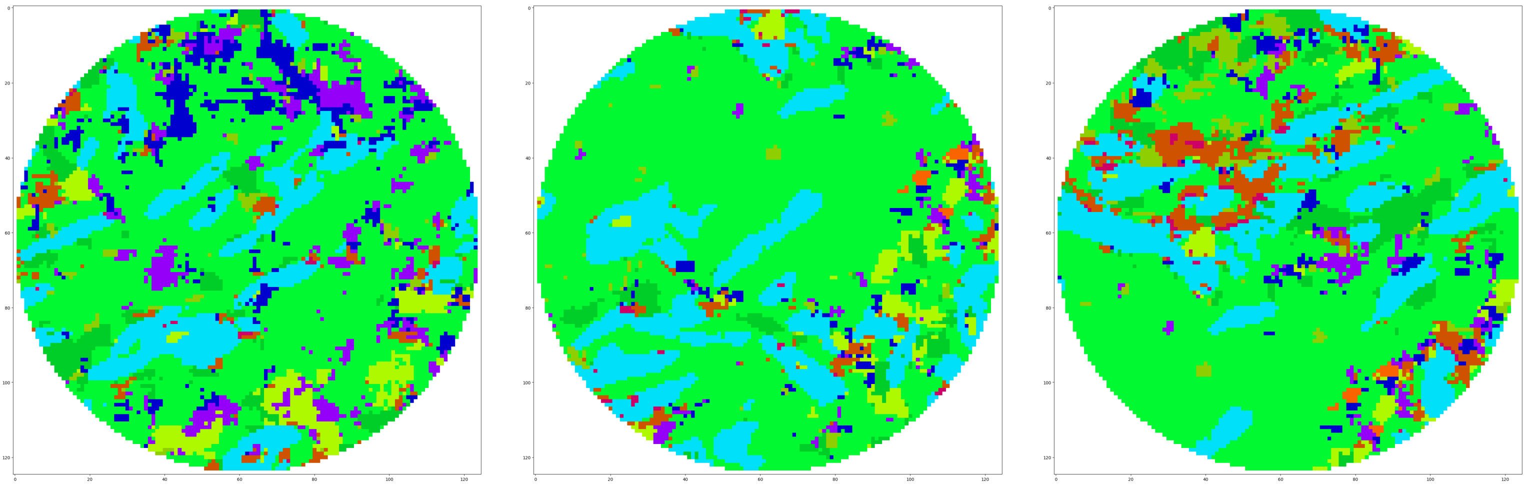

<u>Case 16</u>

Semi-automated output

Left: H&E stained section in the spot chosen by microscopic assessment. Middle: the first step was making an segmentation output. Right the class labels can be displayed

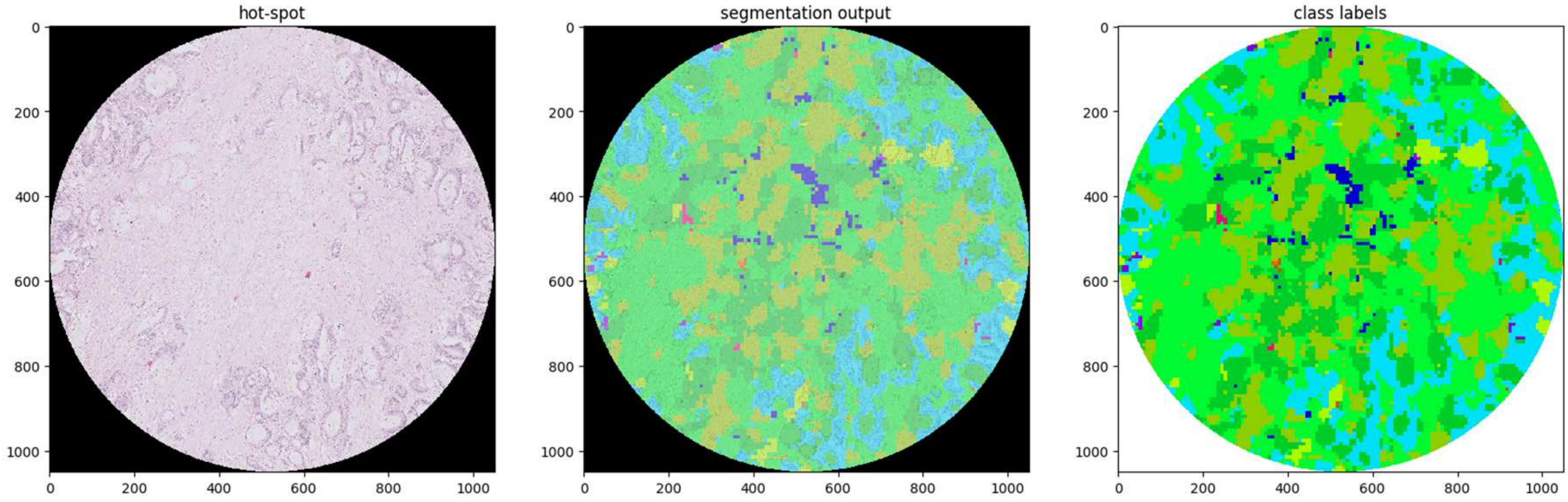

Fully-automated output

Top row; left: the tumor bulk is annotated. Right: heatmap is created. The biggest dot corresponds with the highest stroma-percentage (TSR-1), the second biggest with the second highest (TSR-2), etcetera.

Bottom row; left: the class output of the highest spot (TSR-1), middle: the second highest spot (TSR-2) and right the third highest spot (TSR-3)

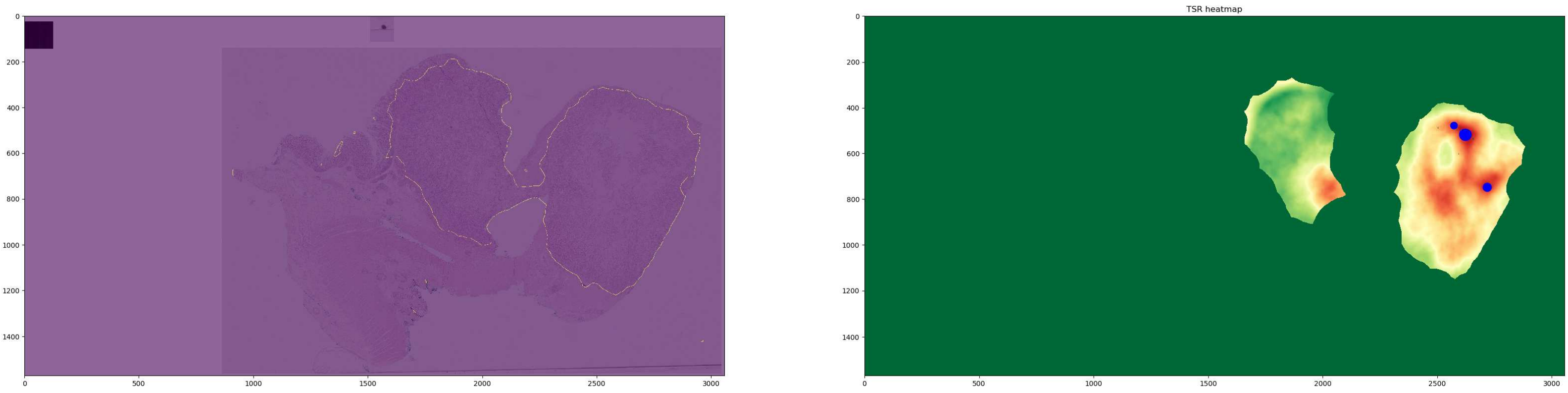

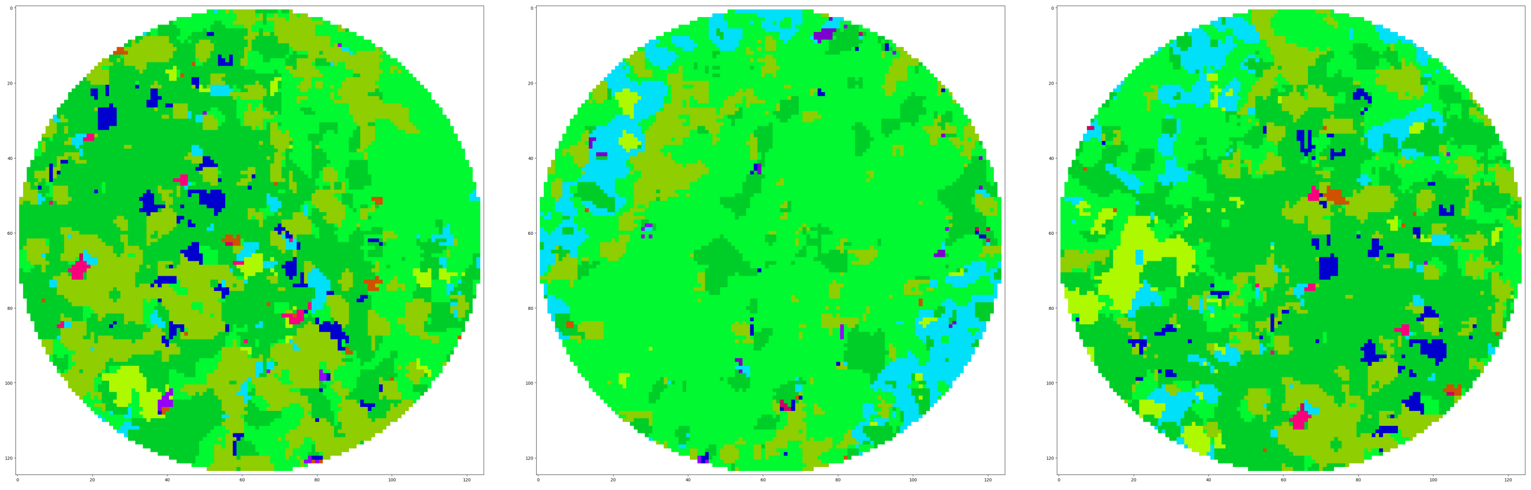

Case 17

Semi-automated output

Left: H&E stained section in the spot chosen by microscopic assessment. Middle: the first step was making an segmentation output. Right the class labels can be displayed

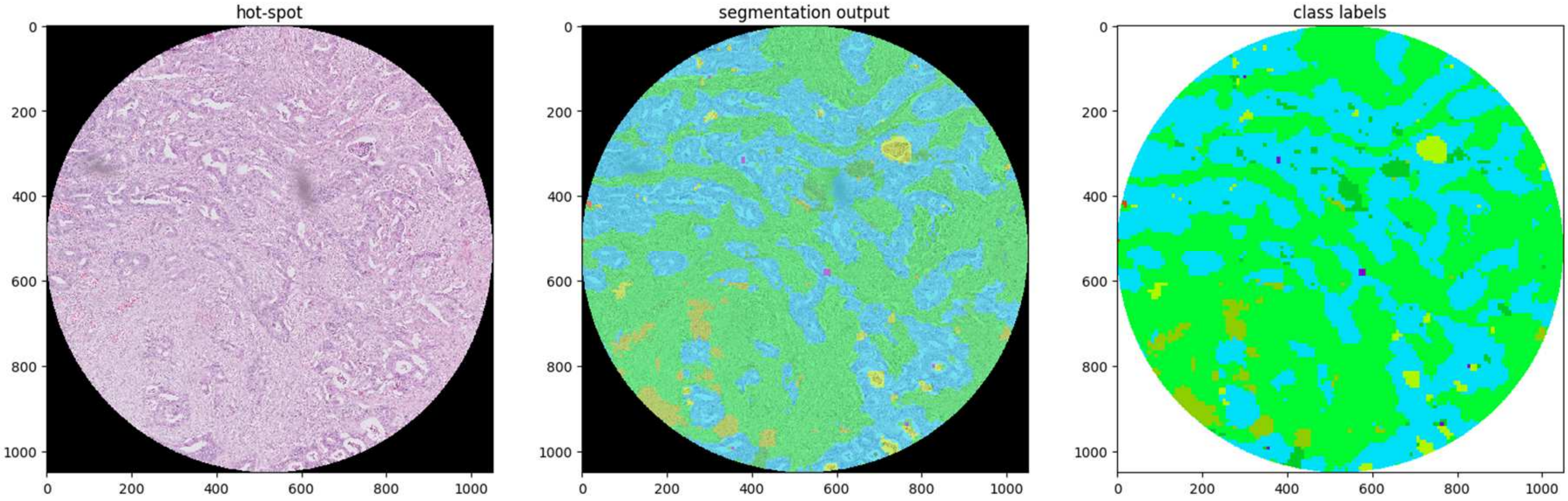

Fully-automated output

Top row; left: the tumor bulk is annotated. Right: heatmap is created. The biggest dot corresponds with the highest stroma-percentage (TSR-1), the second biggest with the second highest (TSR-2), etcetera.

Bottom row; left: the class output of the highest spot (TSR-1), middle: the second highest spot (TSR-2) and right the third highest spot (TSR-3)

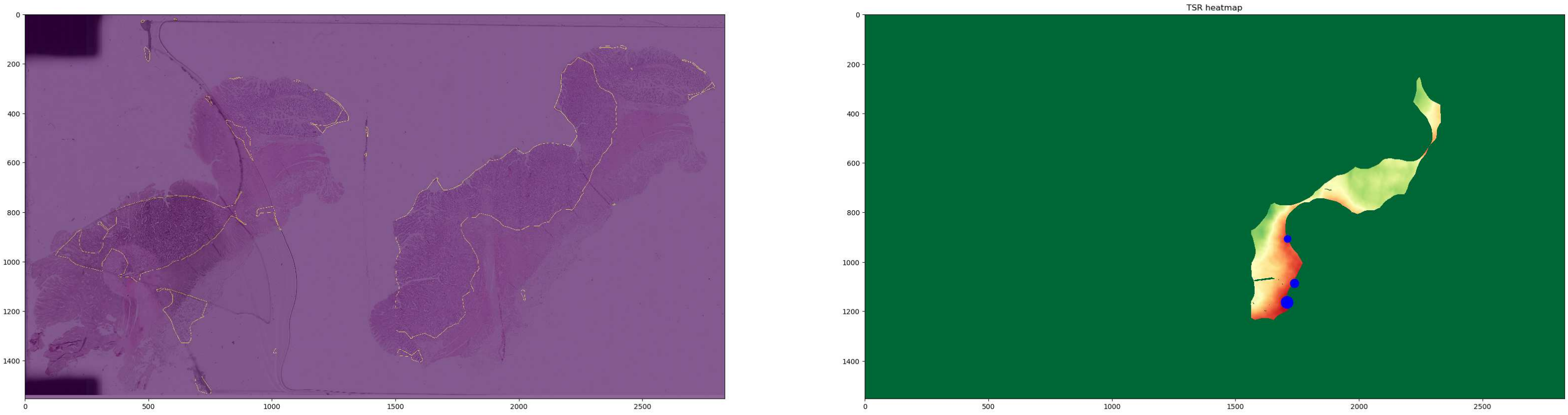

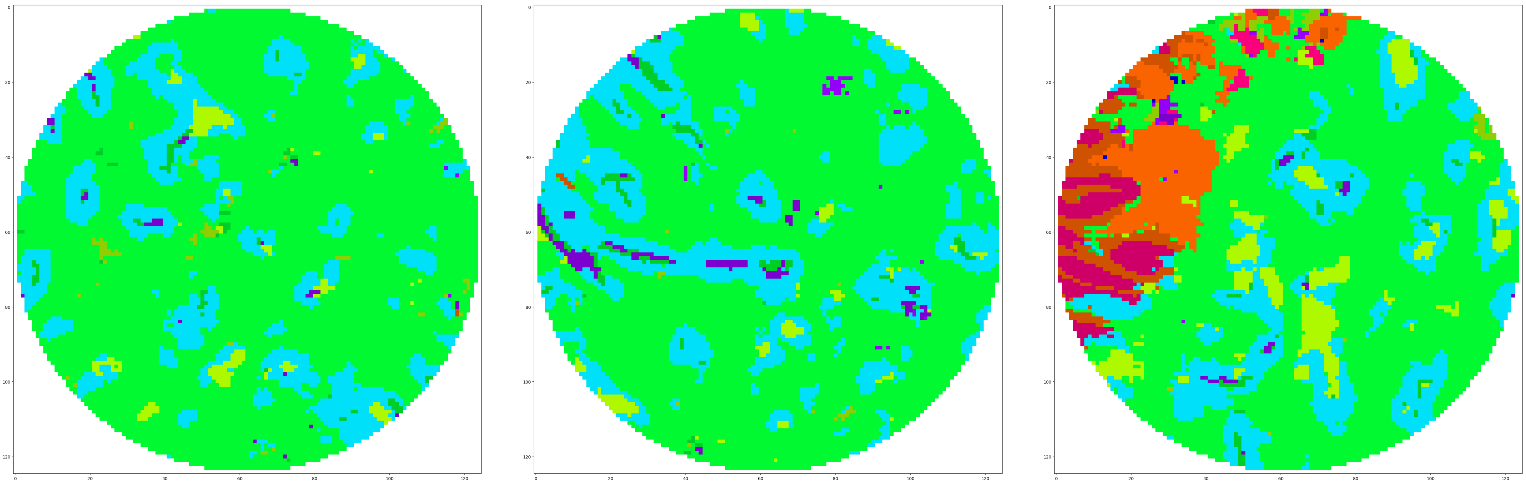

<u>Case 18</u>

Semi-automated output

Left: H&E stained section in the spot chosen by microscopic assessment. Middle: the first step was making an segmentation output. Right the class labels can be displayed

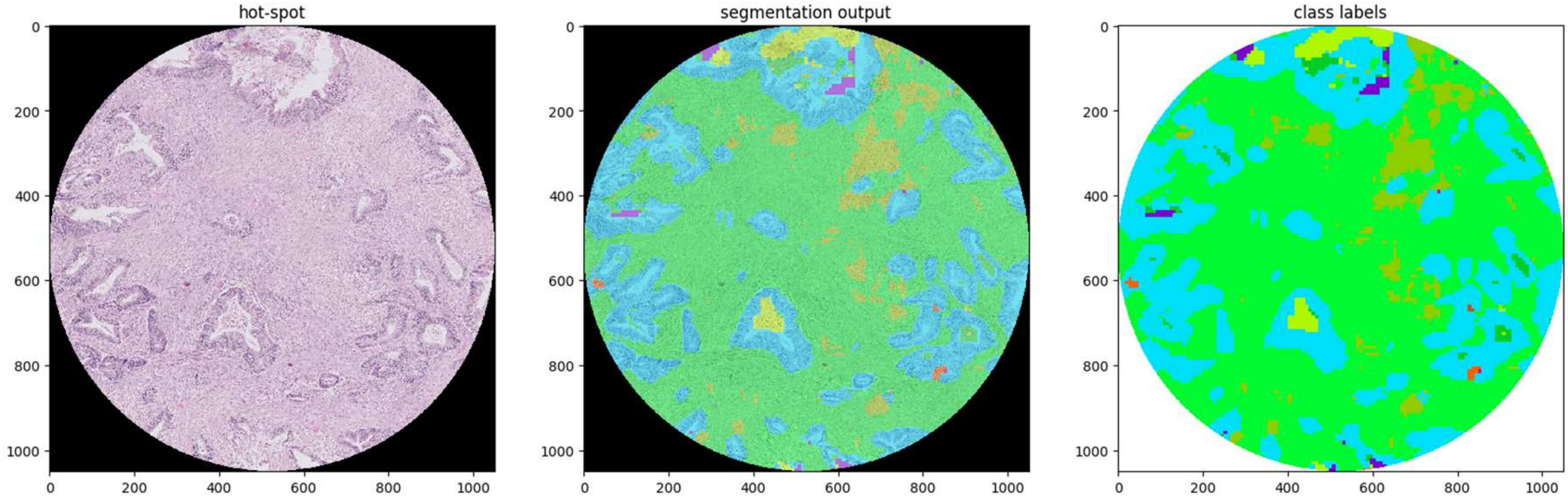

Fully-automated output

Top row; left: the tumor bulk is annotated. Right: heatmap is created. The biggest dot corresponds with the highest stroma-percentage (TSR-1), the second biggest with the second highest (TSR-2), etcetera.

Bottom row; left: the class output of the highest spot (TSR-1), middle: the second highest spot (TSR-2) and right the third highest spot (TSR-3)

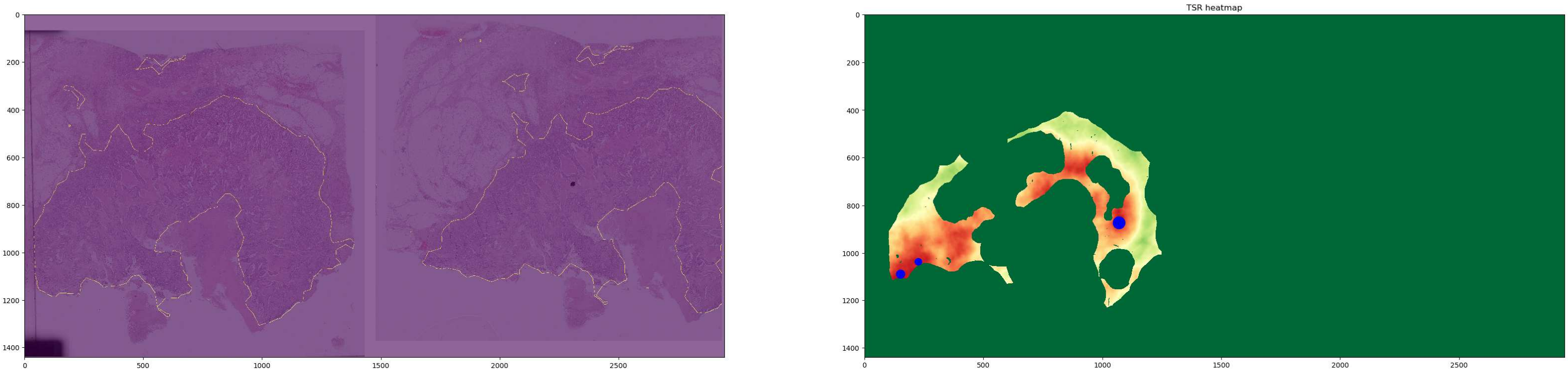

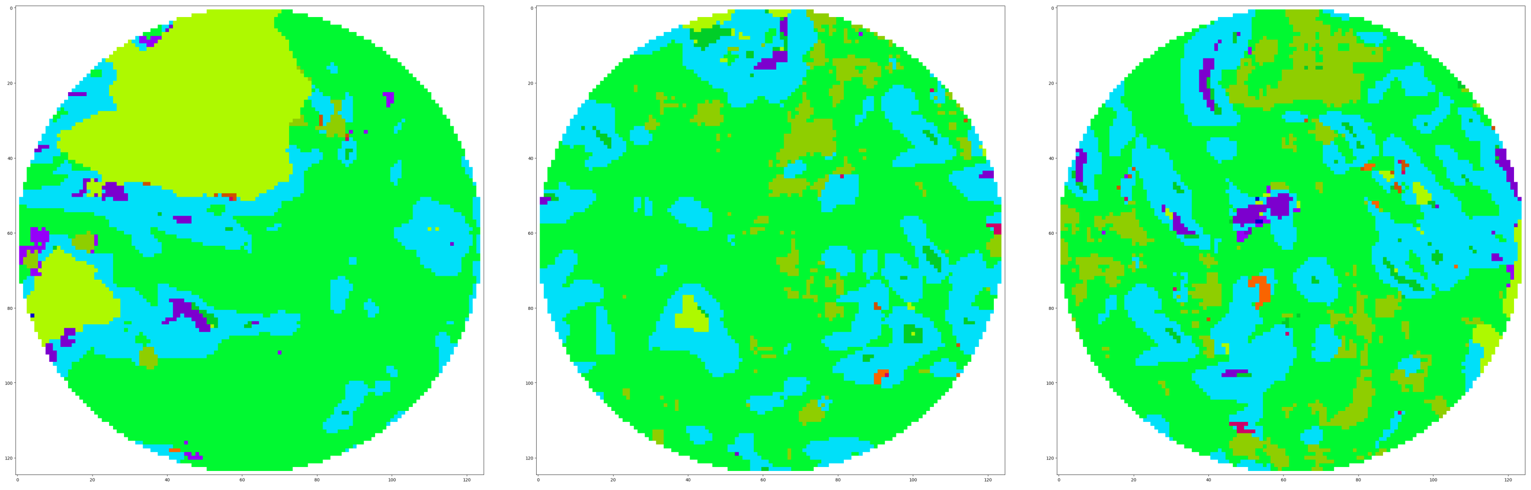

<u>Case 19</u>

Semi-automated output

Left: H&E stained section in the spot chosen by microscopic assessment. Middle: the first step was making an segmentation output. Right the class labels can be displayed

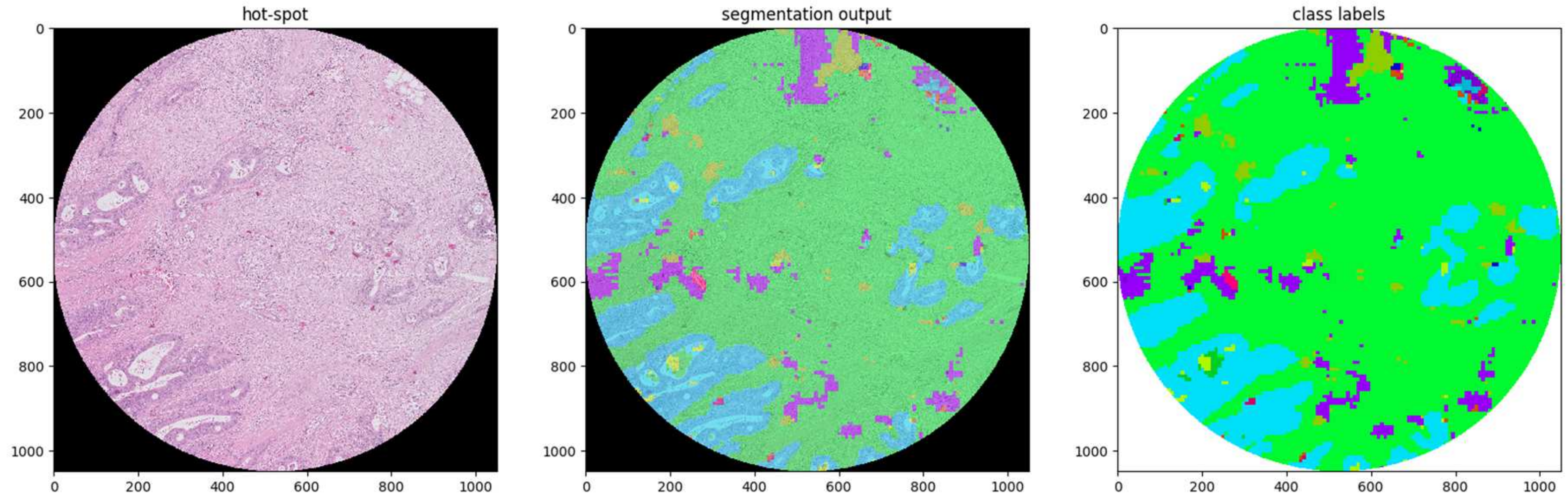

Fully-automated output

Top row; left: the tumor bulk is annotated. Right: heatmap is created. The biggest dot corresponds with the highest stroma-percentage (TSR-1), the second biggest with the second highest (TSR-2), etcetera.

Bottom row; left: the class output of the highest spot (TSR-1), middle: the second highest spot (TSR-2) and right the third highest spot (TSR-3)

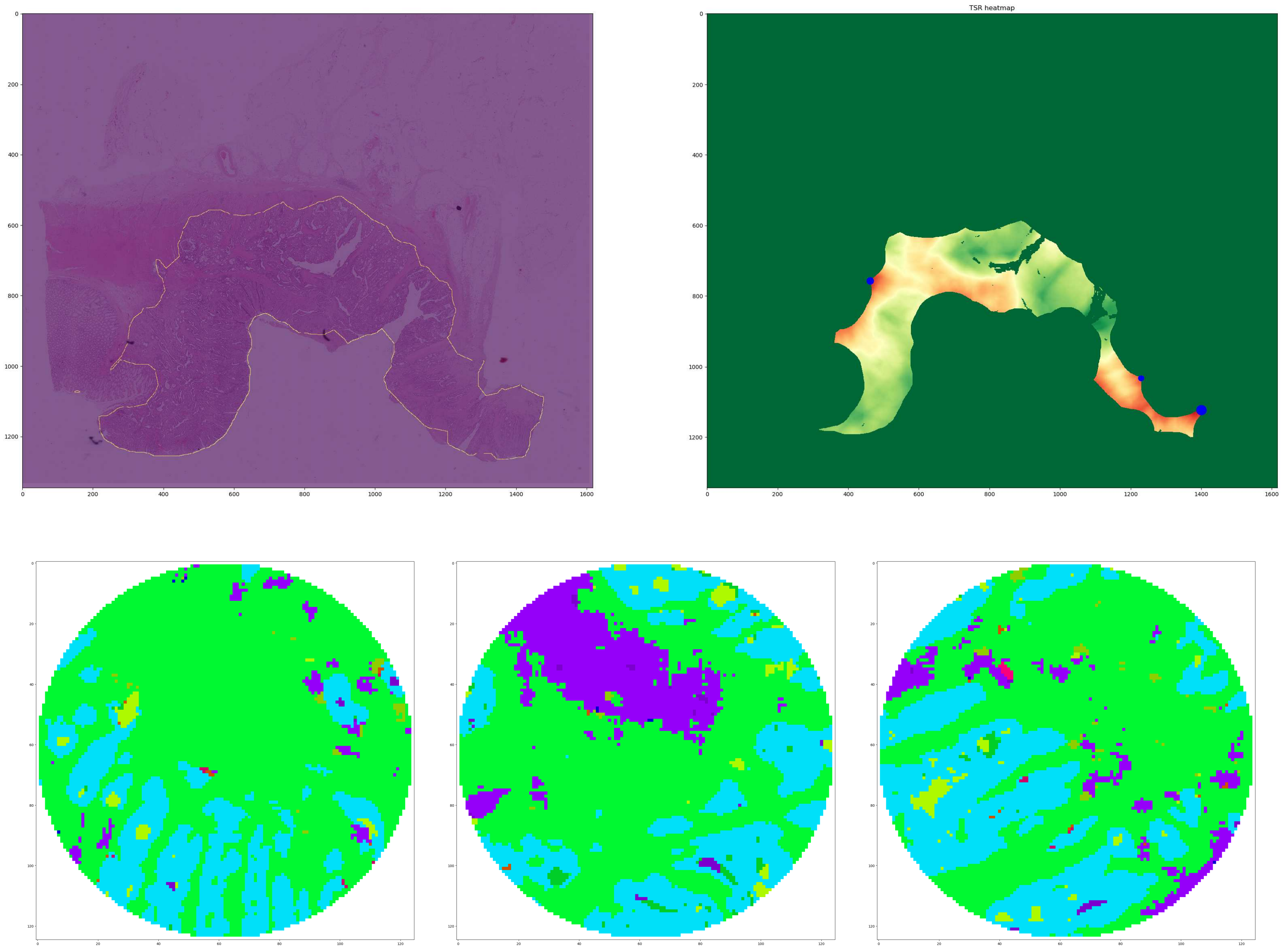

Case 20

Semi-automated output

Left: H&E stained section in the spot chosen by microscopic assessment. Middle: the first step was making an segmentation output. Right the class labels can be displayed

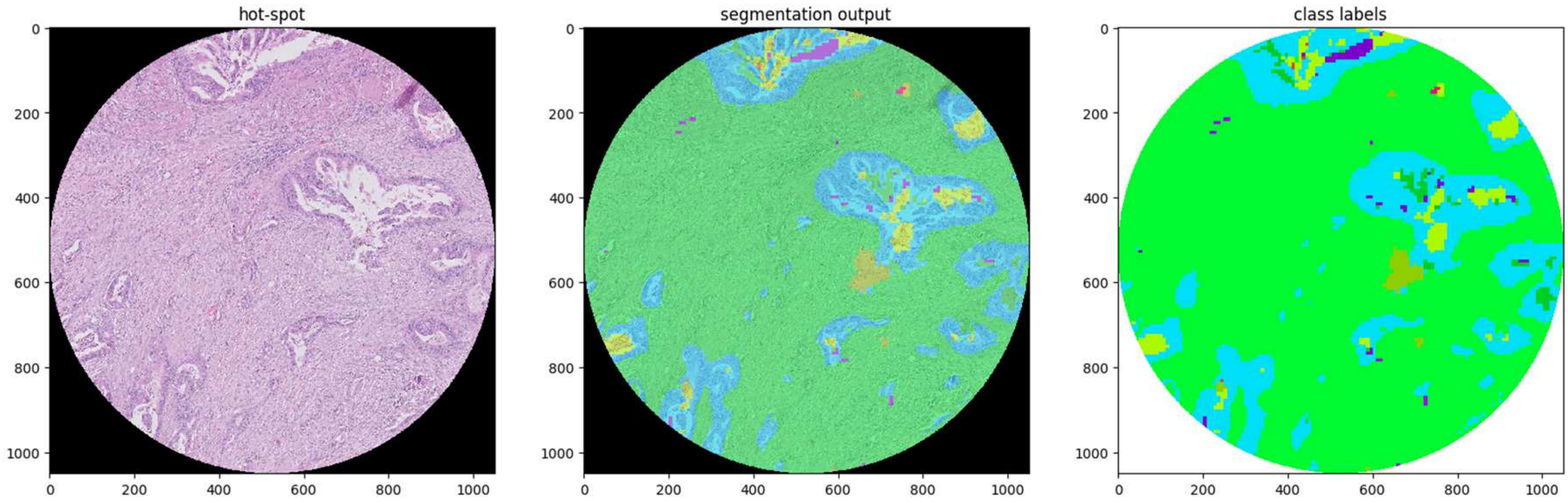

Fully-automated output

Top row; left: the tumor bulk is annotated. Right: heatmap is created. The biggest dot corresponds with the highest stroma-percentage (TSR-1), the second biggest with the second highest (TSR-2), etcetera.

Bottom row; left: the class output of the highest spot (TSR-1), middle: the second highest spot (TSR-2) and right the third highest spot (TSR-3)

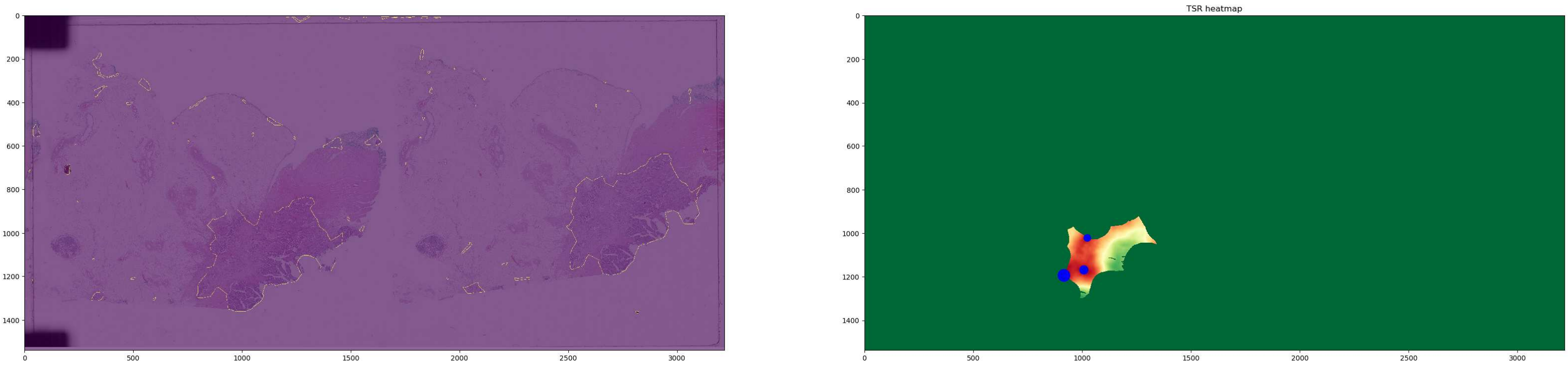

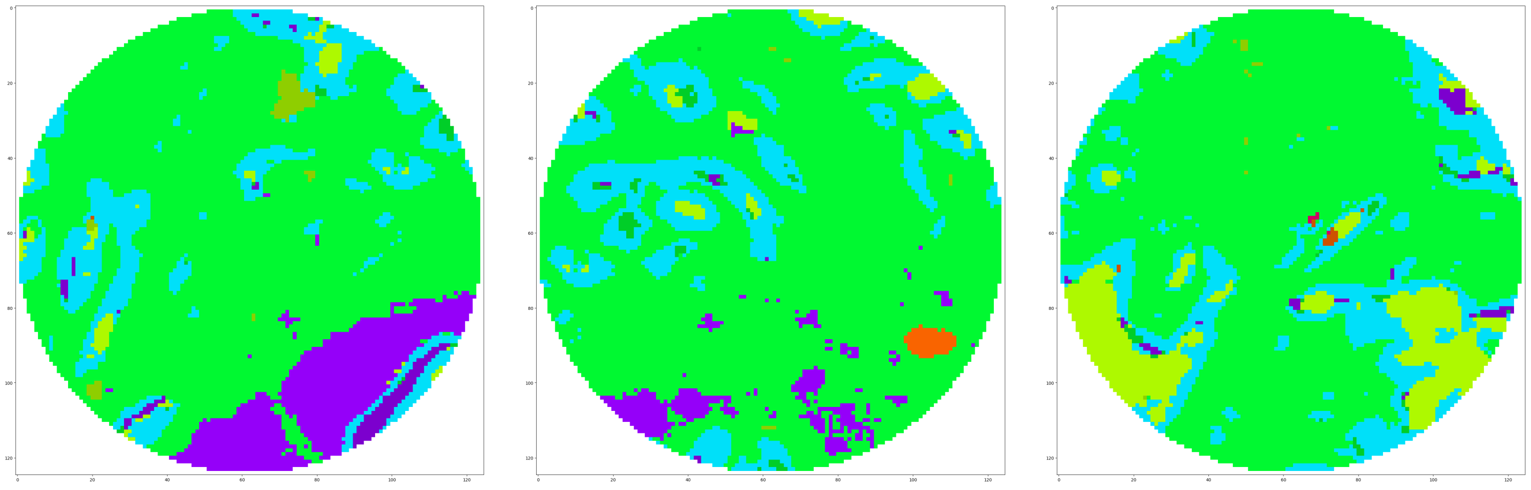

Case 21

Semi-automated output

Left: H&E stained section in the spot chosen by microscopic assessment. Middle: the first step was making an segmentation output. Right the class labels can be displayed

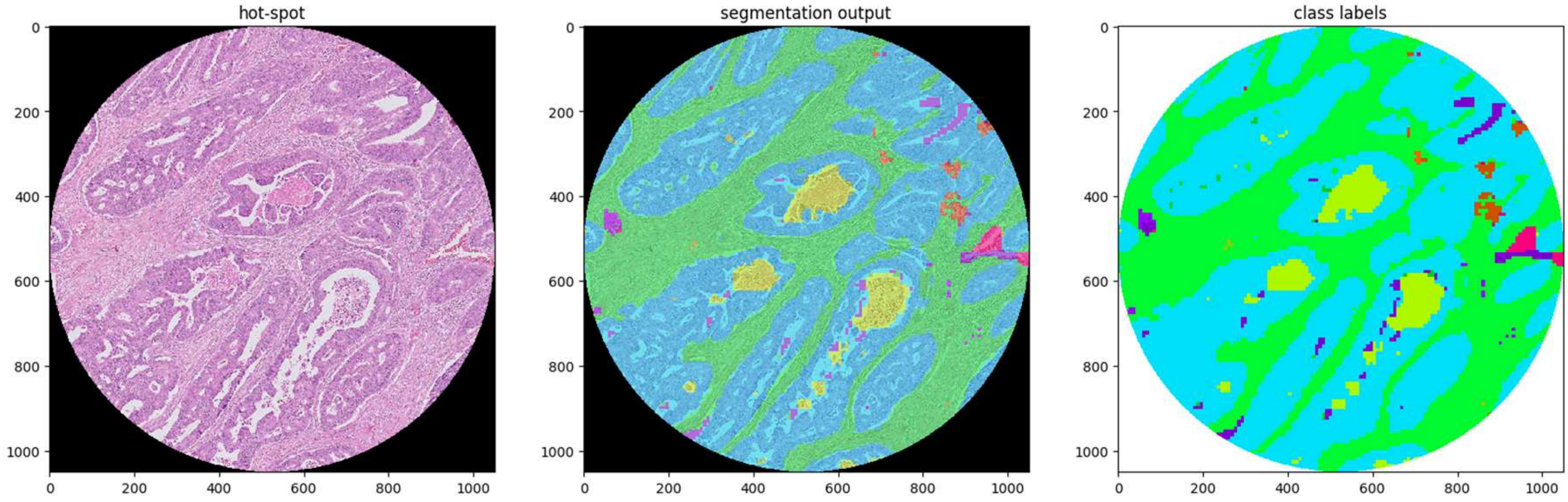

Fully-automated output

Top row; left: the tumor bulk is annotated. Right: heatmap is created. The biggest dot corresponds with the highest stroma-percentage (TSR-1), the second biggest with the second highest (TSR-2), etcetera.

Bottom row; left: the class output of the highest spot (TSR-1), middle: the second highest spot (TSR-2) and right the third highest spot (TSR-3)

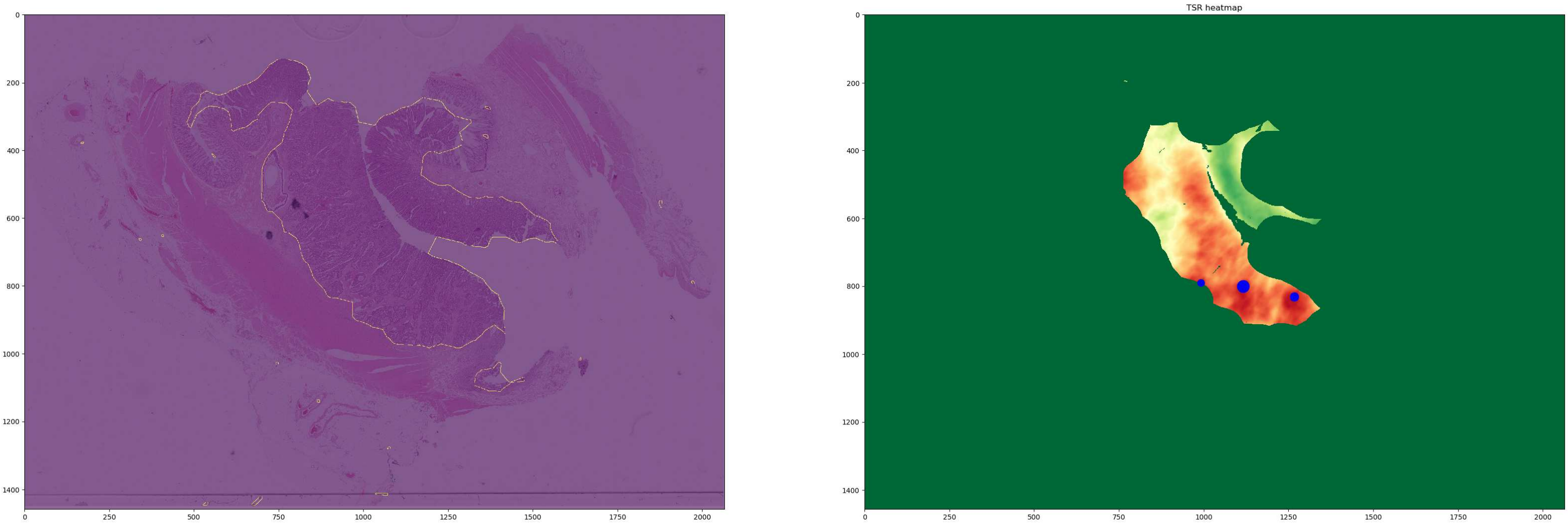

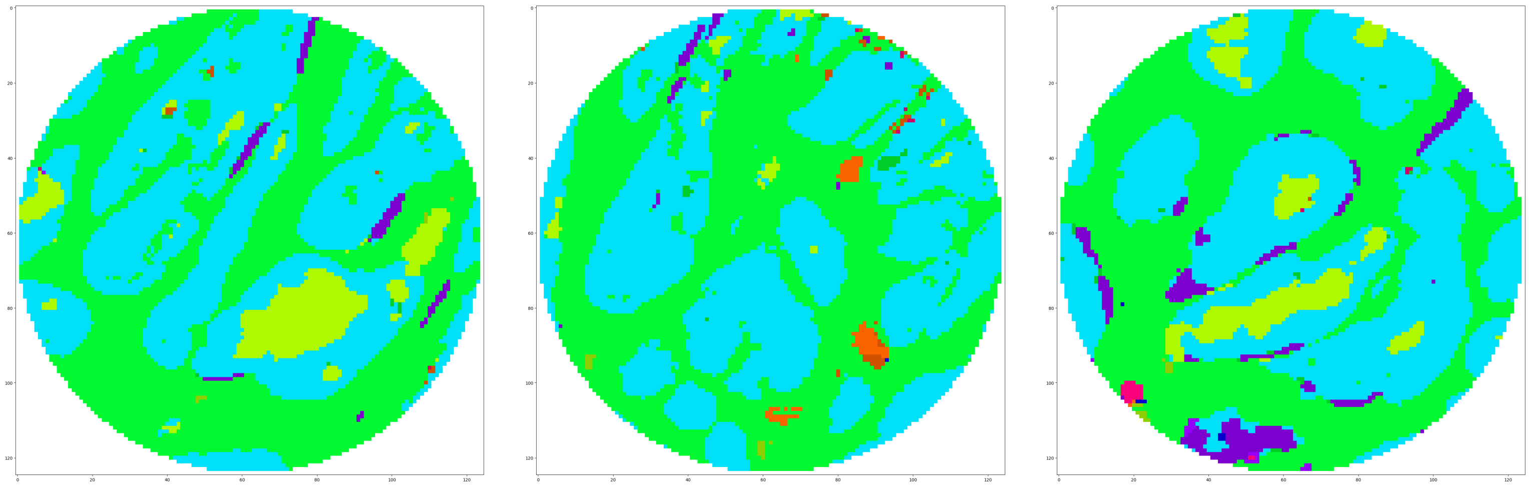

<u>Case 22</u>

Semi-automated output

Left: H&E stained section in the spot chosen by microscopic assessment. Middle: the first step was making an segmentation output. Right the class labels can be displayed

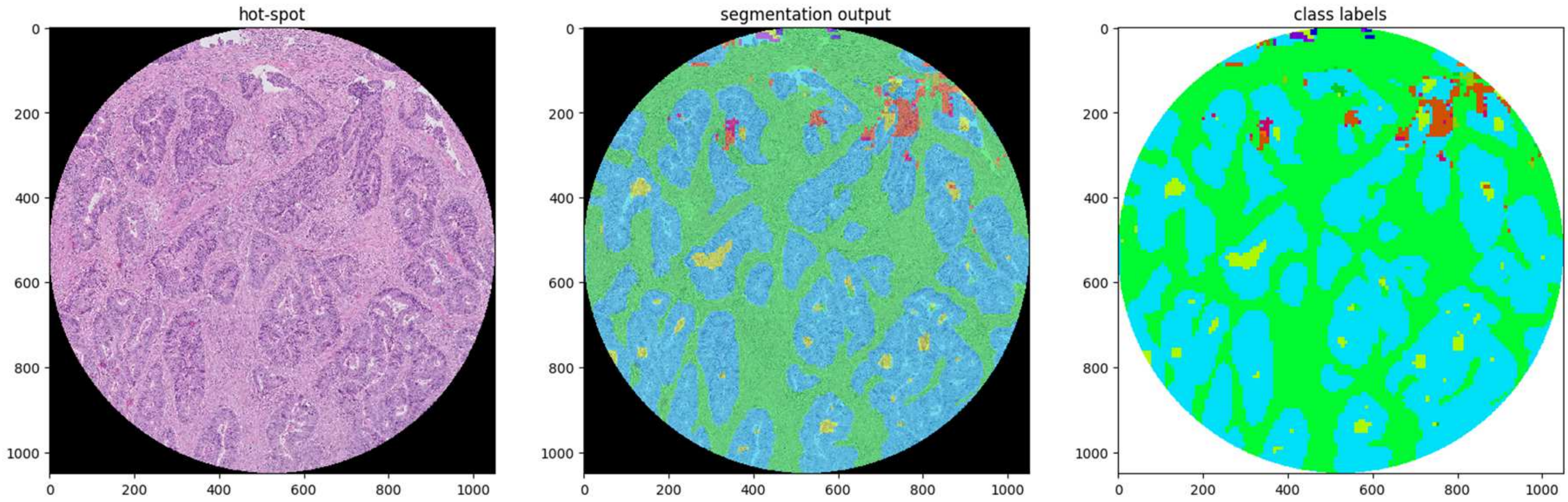

Fully-automated output

Top row; left: the tumor bulk is annotated. Right: heatmap is created. The biggest dot corresponds with the highest stroma-percentage (TSR-1), the second biggest with the second highest (TSR-2), etcetera.

Bottom row; left: the class output of the highest spot (TSR-1), middle: the second highest spot (TSR-2) and right the third highest spot (TSR-3)

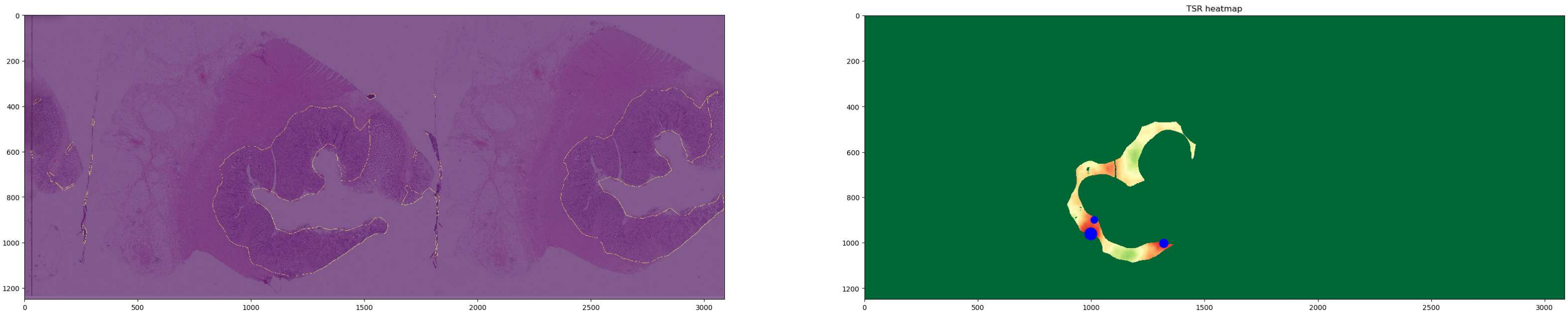

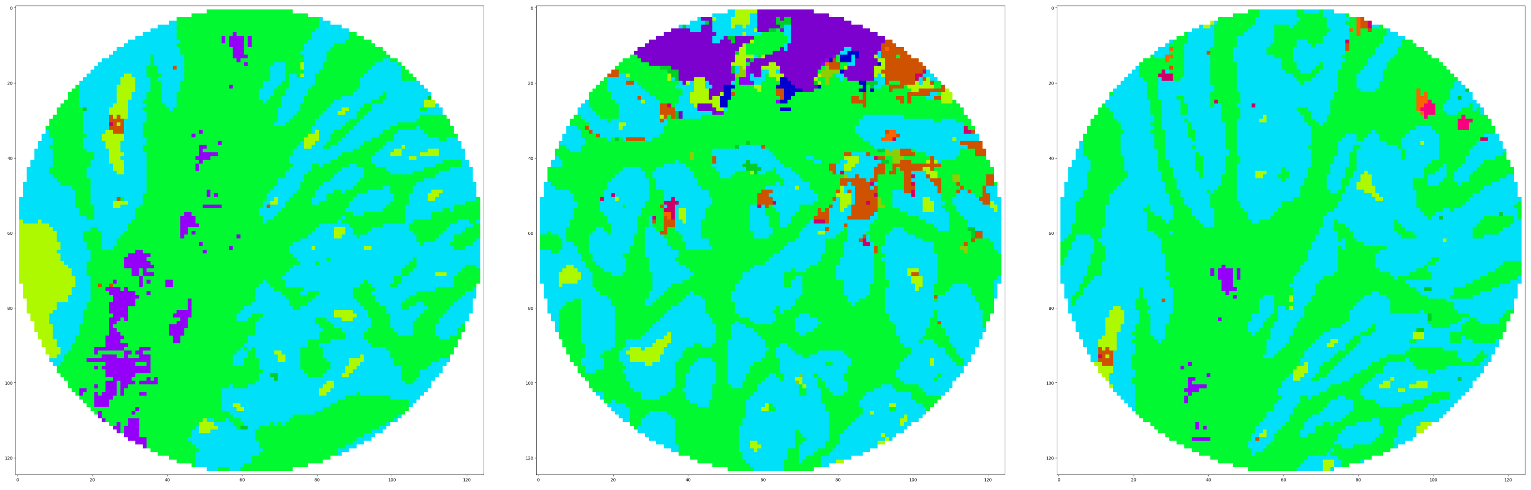

Case 23

Semi-automated output

Left: H&E stained section in the spot chosen by microscopic assessment. Middle: the first step was making an segmentation output. Right the class labels can be displayed

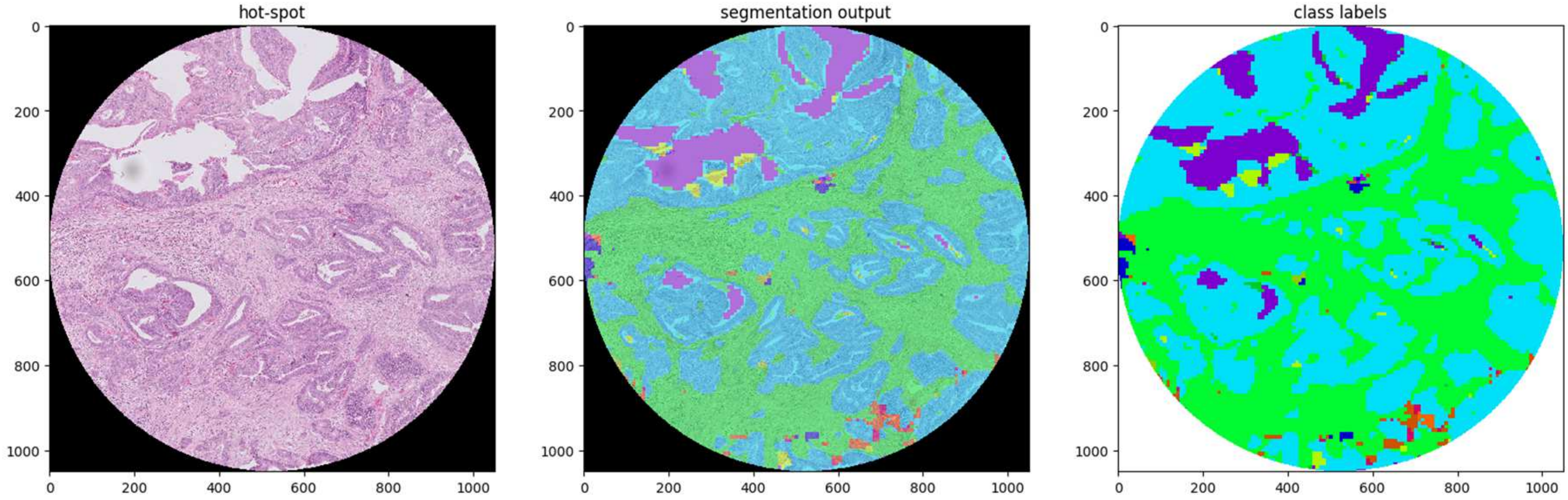

Fully-automated output

Top row; left: the tumor bulk is annotated. Right: heatmap is created. The biggest dot corresponds with the highest stroma-percentage (TSR-1), the second biggest with the second highest (TSR-2), etcetera.

Bottom row; left: the class output of the highest spot (TSR-1), middle: the second highest spot (TSR-2) and right the third highest spot (TSR-3)

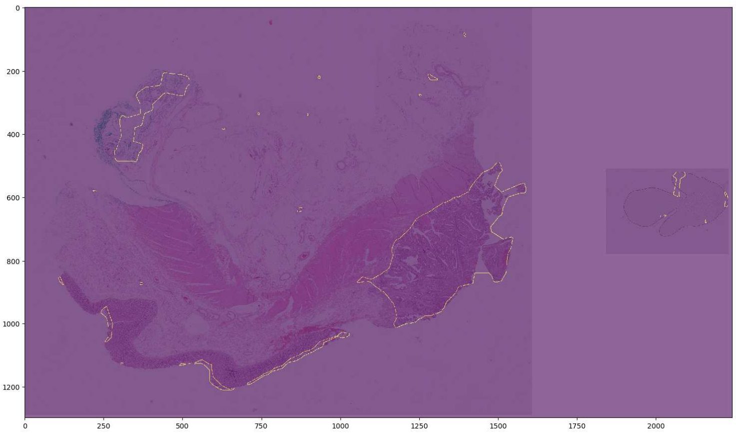

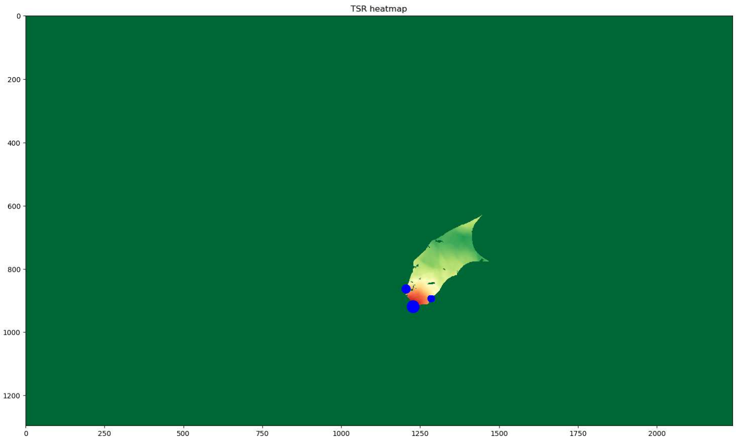

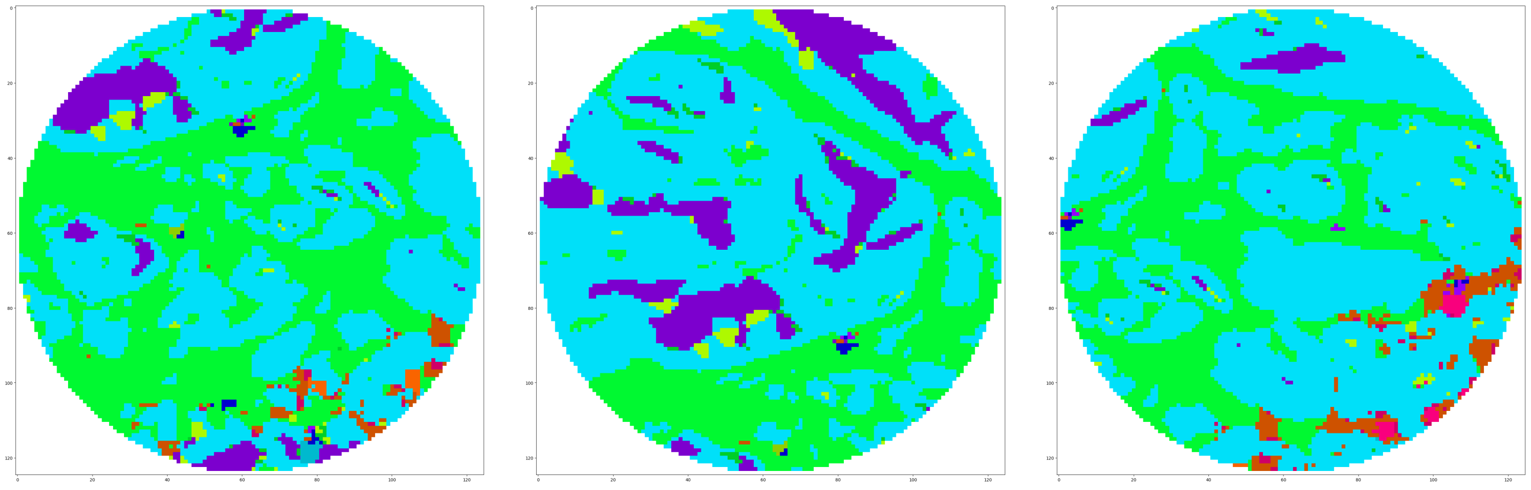

Case 24

Semi-automated output

Left: H&E stained section in the spot chosen by microscopic assessment. Middle: the first step was making an segmentation output. Right the class labels can be displayed

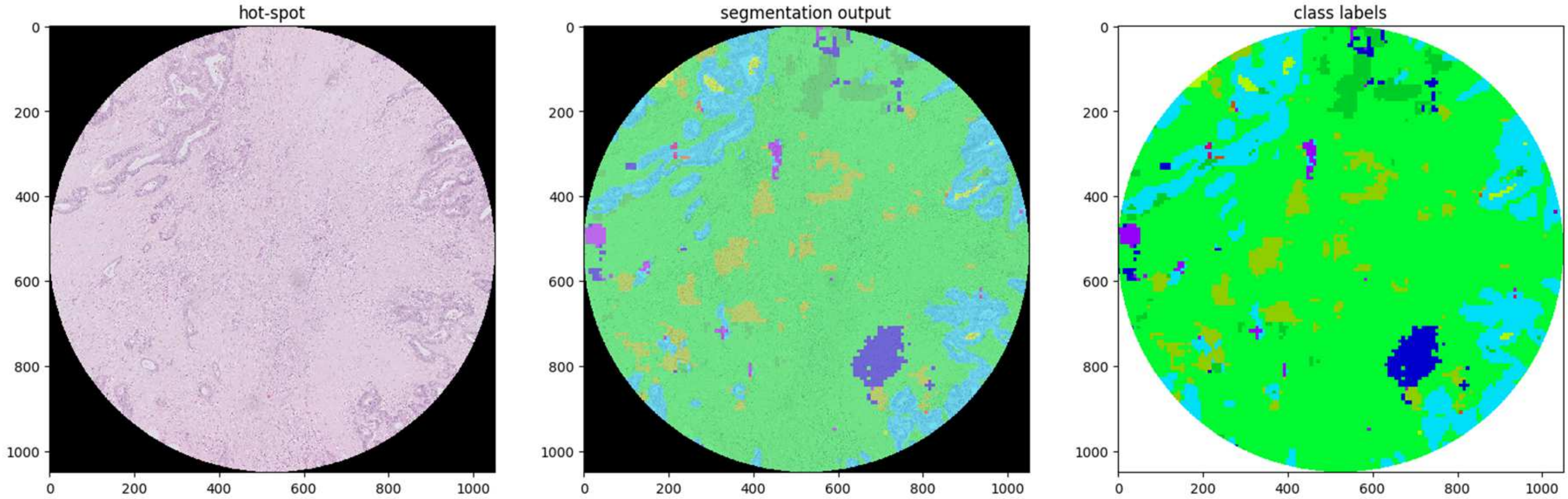

Fully-automated output

Top row; left: the tumor bulk is annotated. Right: heatmap is created. The biggest dot corresponds with the highest stroma-percentage (TSR-1), the second biggest with the second highest (TSR-2), etcetera.

Bottom row; left: the class output of the highest spot (TSR-1), middle: the second highest spot (TSR-2) and right the third highest spot (TSR-3)

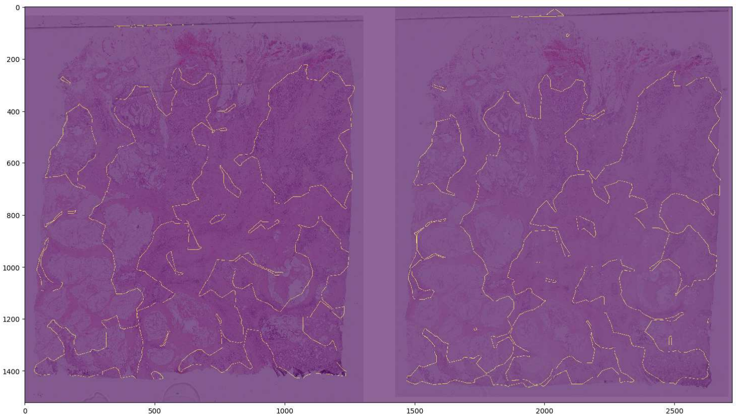

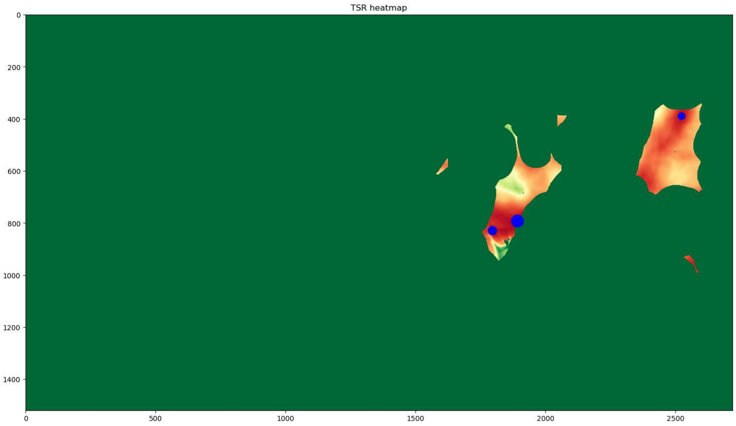

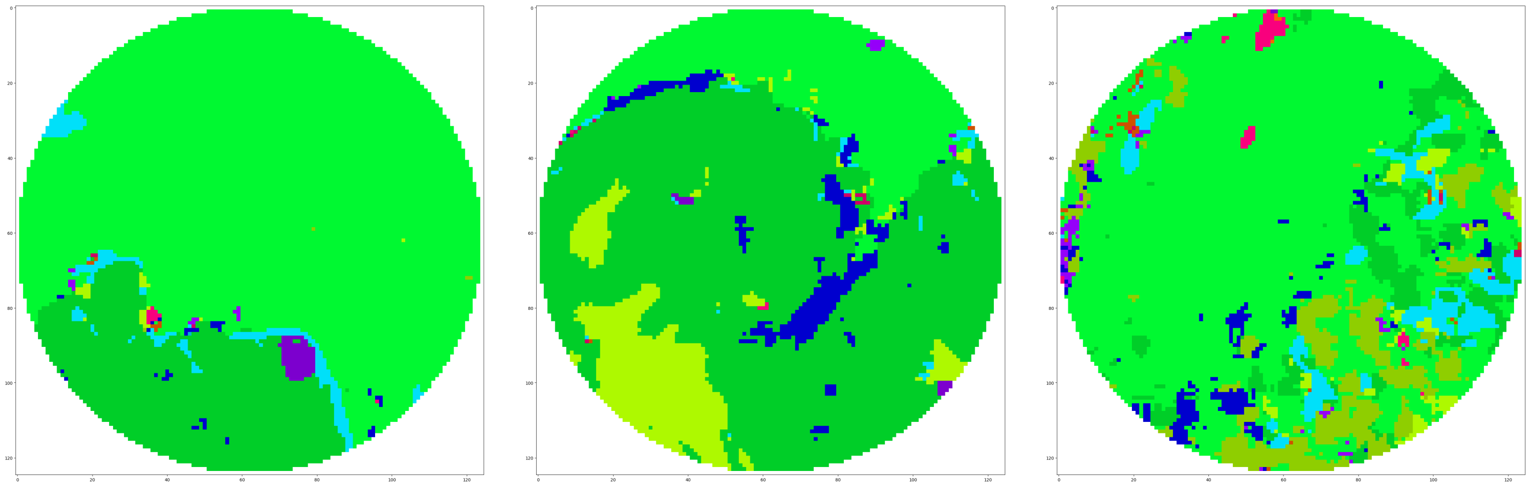

Supplement: Supplementary material 3 — Output for all cases for the semi-automated algorithm and the fully-automated algorithm. In part 1 case 1-24, part 2 case 25-49 and part 3 50-75. [file mmc3.zip › mmc3/Supplementary data - output figures (part 1).pdf]
