# Supplementary material for: Deep learning based tumor–stroma ratio scoring in colon cancer correlates with microscopic assessment
Source: J Pathol Inform. 2023 Jan 20;14:100191. doi: 10.1016/j.jpi.2023.100191 (PMC9922811; doi:10.1016/j.jpi.2023.100191)

Supplementary file

Original data figures

(Part 2)

Legend

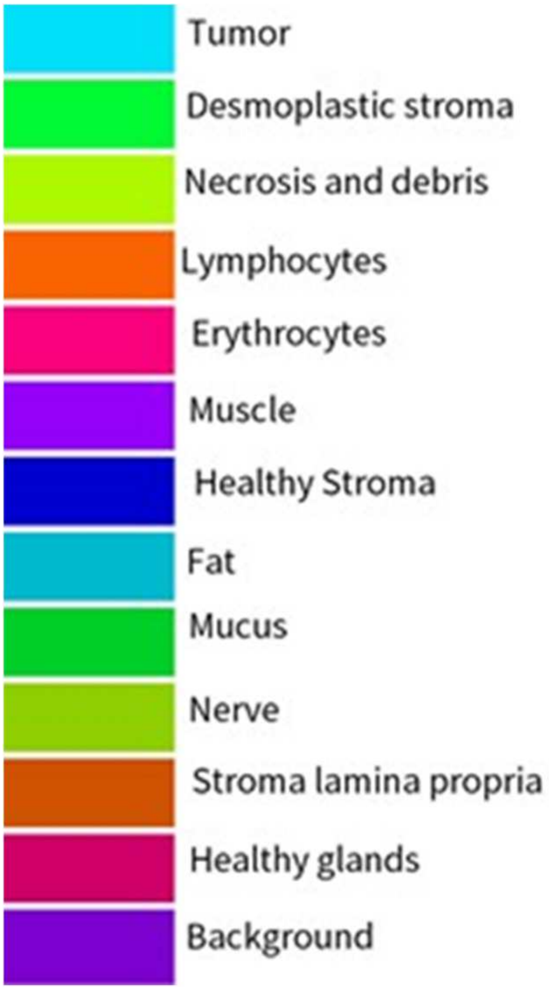
Tumor
Desmoplastic stroma
Necrosis and debris
Lymphocytes
Erythrocytes
Muscle
Healthy Stroma
Fat
Mucus
Nerve
Stroma lamina propria
Healthy glands
Background

Case 25

Semi-automated output

Left: H&E stained section in the spot chosen by microscopic assessment. Middle: the first step was making an segmentation output. Right the class labels can be displayed

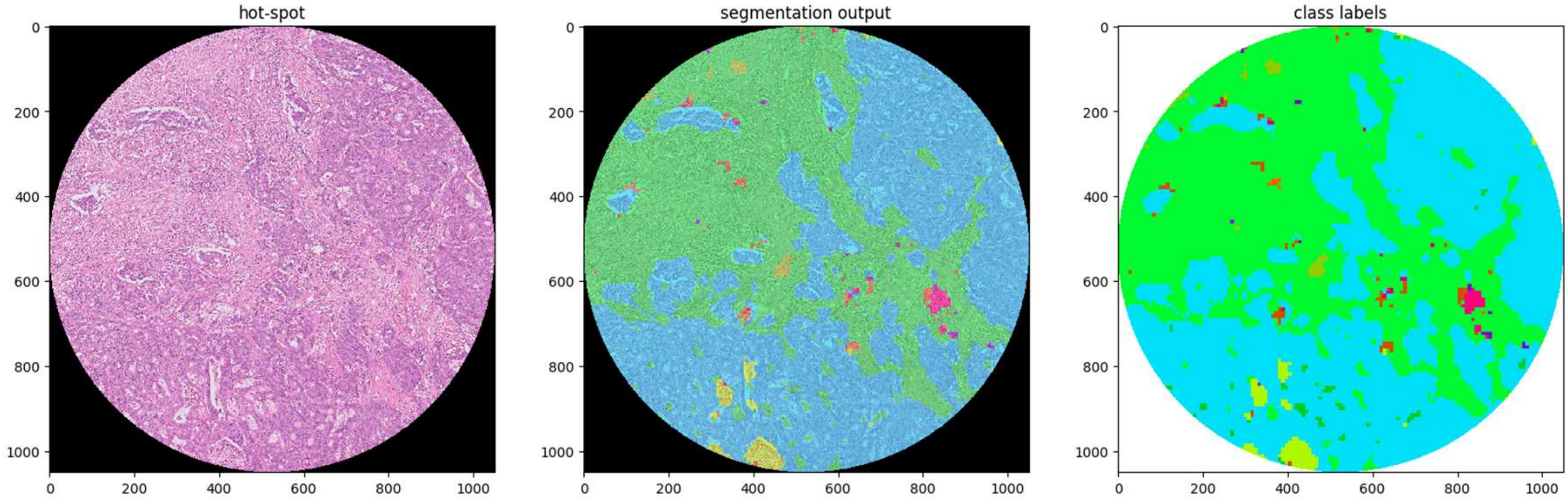

Fully-automated output

Top row; left: the tumor bulk is annotated. Right: heatmap is created. The biggest dot corresponds with the highest stroma-percentage (TSR-1), the second biggest with the second highest (TSR-2), etcetera.

Bottom row; left: the class output of the highest spot (TSR-1), middle: the second highest spot (TSR-2) and right the third highest spot (TSR-3)

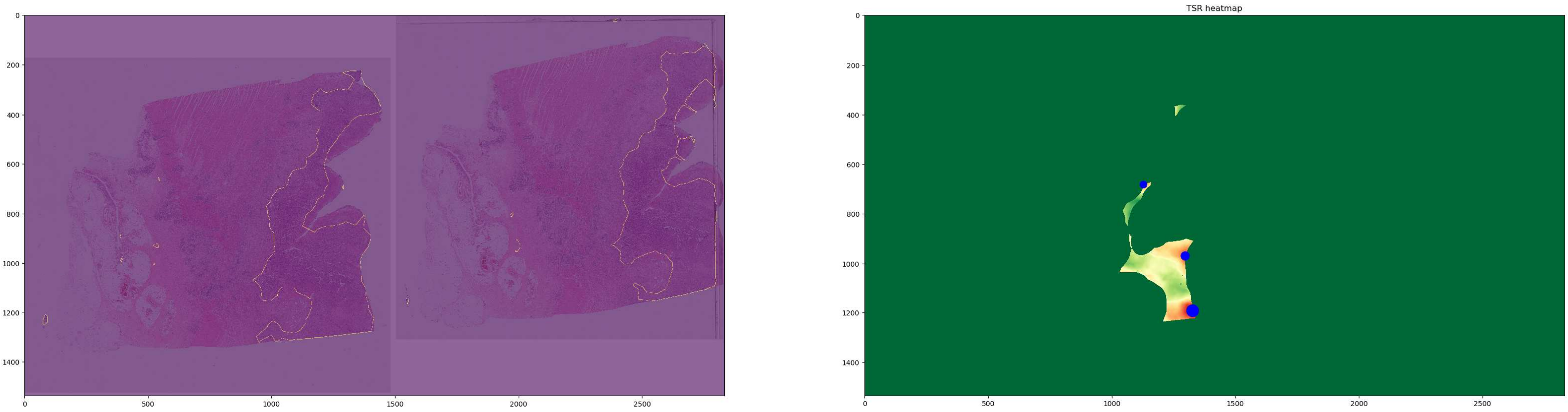

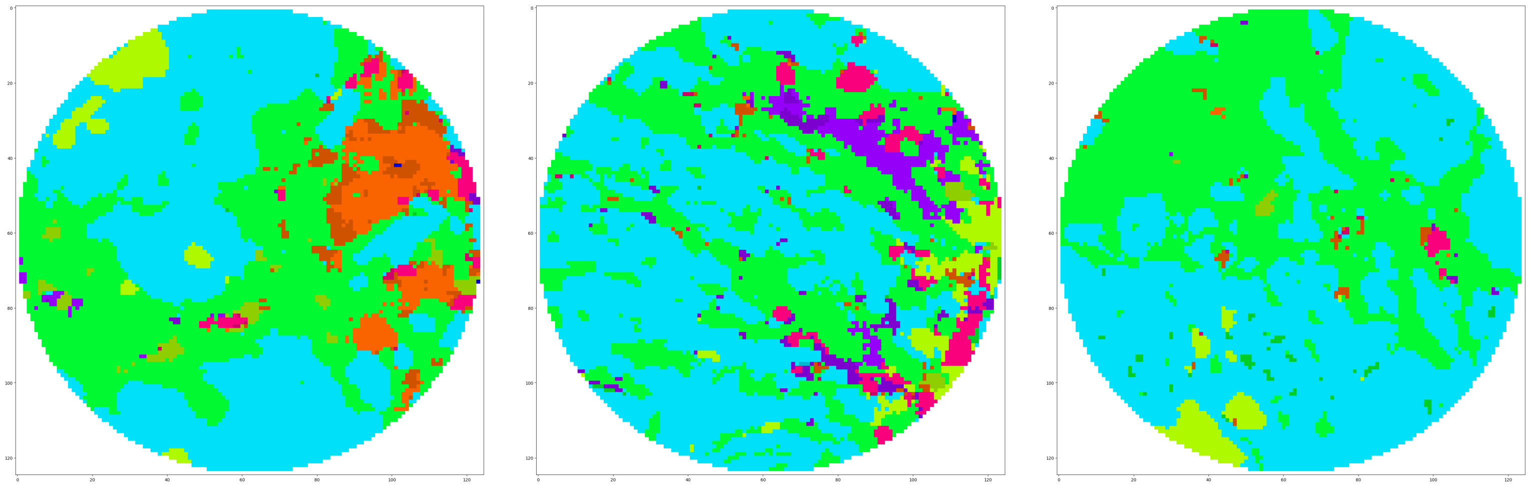

<u>Case 26</u>

Semi-automated output

Left: H&E stained section in the spot chosen by microscopic assessment. Middle: the first step was making an segmentation output. Right the class labels can be displayed

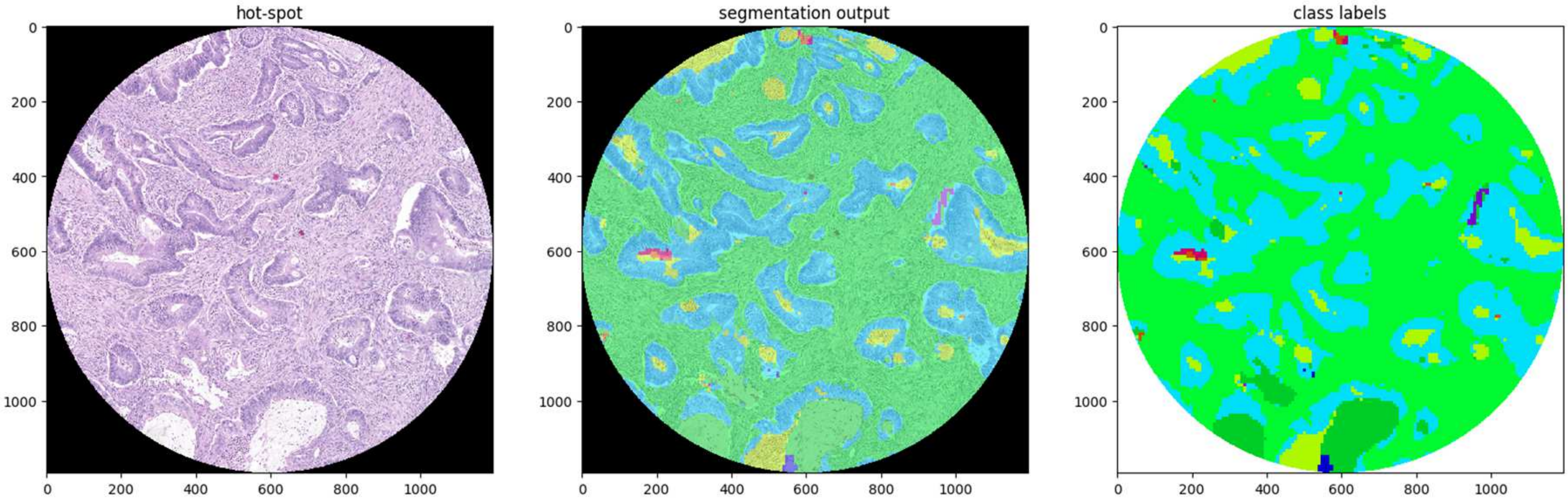

Fully-automated output

Top row; left: the tumor bulk is annotated. Right: heatmap is created. The biggest dot corresponds with the highest stroma-percentage (TSR-1), the second biggest with the second highest (TSR-2), etcetera.

Bottom row; left: the class output of the highest spot (TSR-1), middle: the second highest spot (TSR-2) and right the third highest spot (TSR-3)

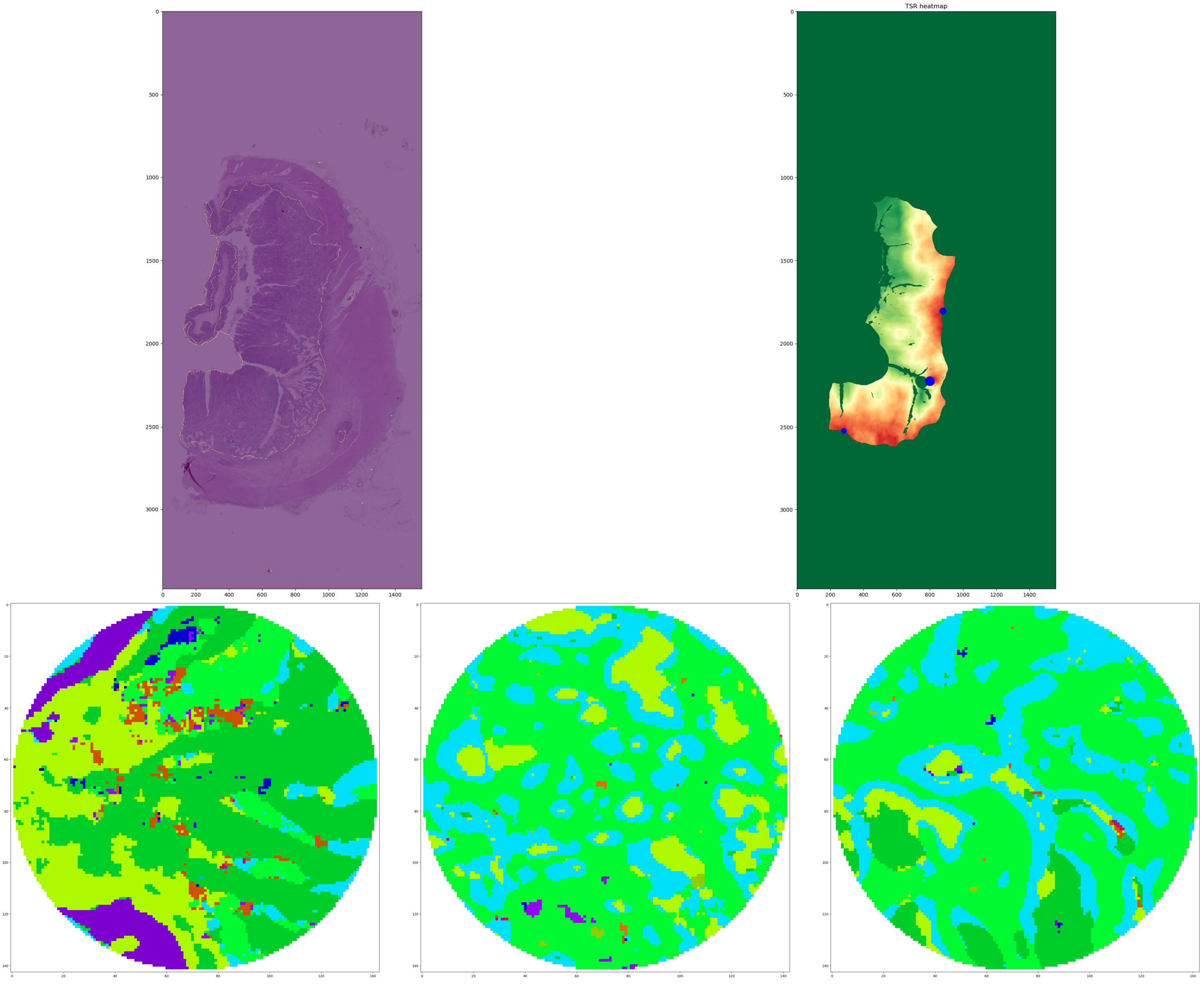

<u>Case 27</u>

Semi-automated output

Left: H&E stained section in the spot chosen by microscopic assessment. Middle: the first step was making an segmentation output. Right the class labels can be displayed

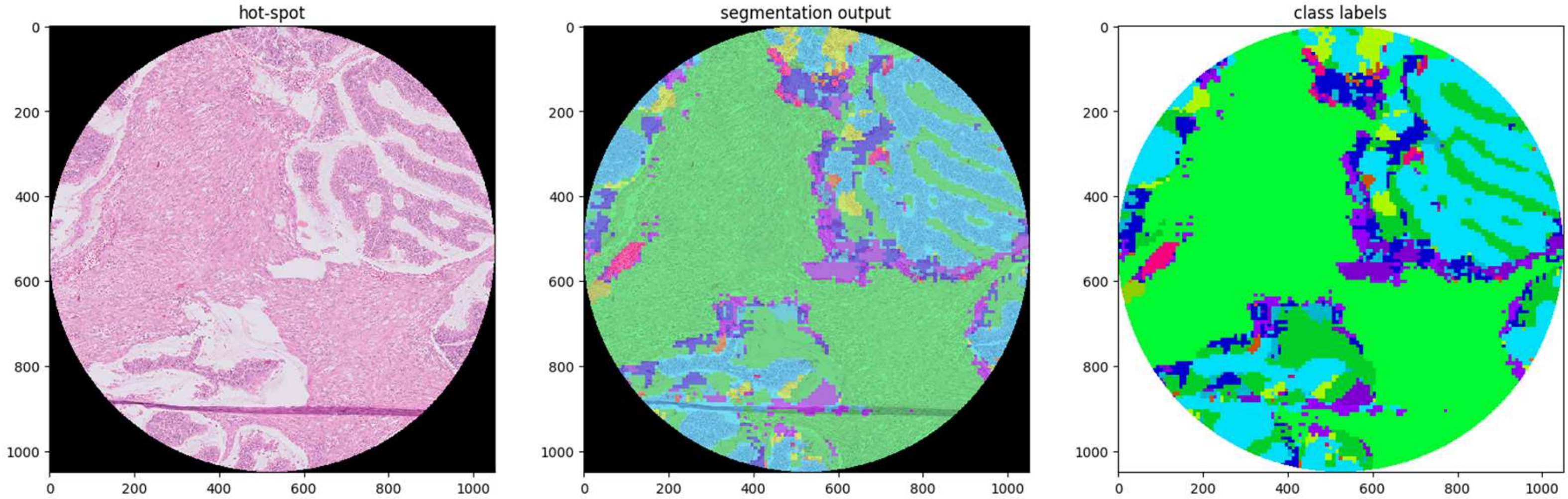

Fully-automated output

Top row; left: the tumor bulk is annotated. Right: heatmap is created. The biggest dot corresponds with the highest stroma-percentage (TSR-1), the second biggest with the second highest (TSR-2), etcetera.
Bottom row; left: the class output of the highest spot (TSR-1), middle: the second highest spot (TSR-2) and right the third highest spot (TSR-3)

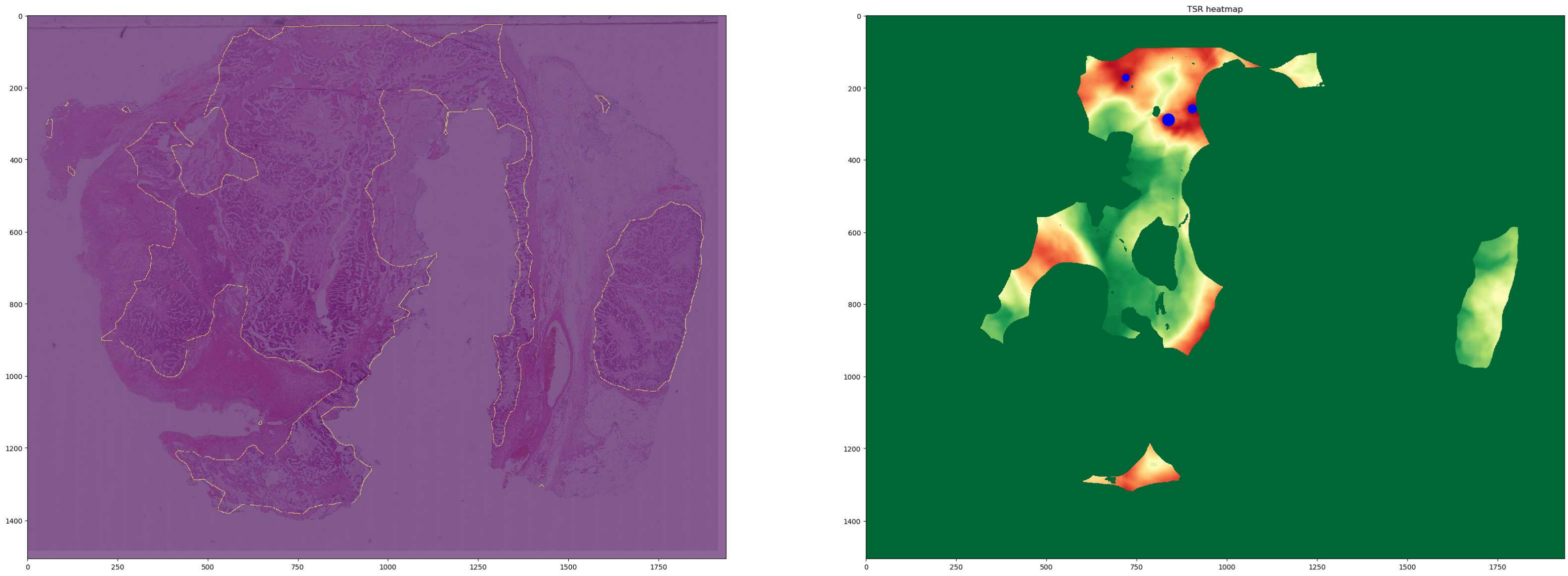

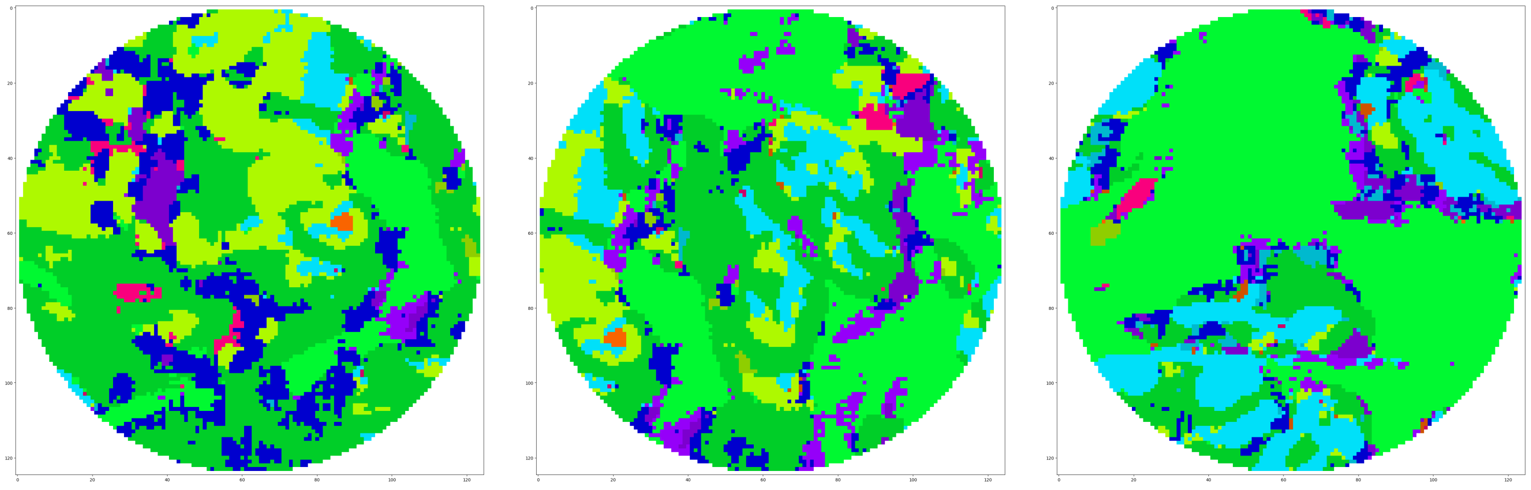

<u>Case 28</u>

Semi-automated output

Left: H&E stained section in the spot chosen by microscopic assessment. Middle: the first step was making an segmentation output. Right the class labels can be displayed

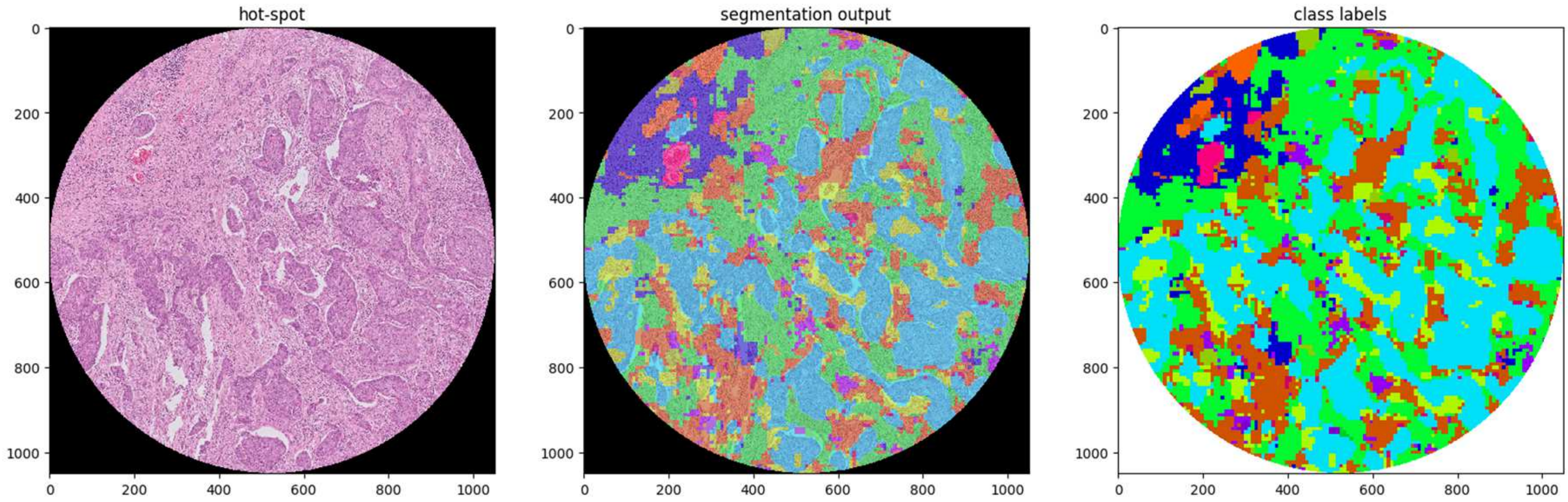

Fully-automated output

Top row; left: the tumor bulk is annotated. Right: heatmap is created. The biggest dot corresponds with the highest stroma-percentage (TSR-1), the second biggest with the second highest (TSR-2), etcetera.

Bottom row; left: the class output of the highest spot (TSR-1), middle: the second highest spot (TSR-2) and right the third highest spot (TSR-3)

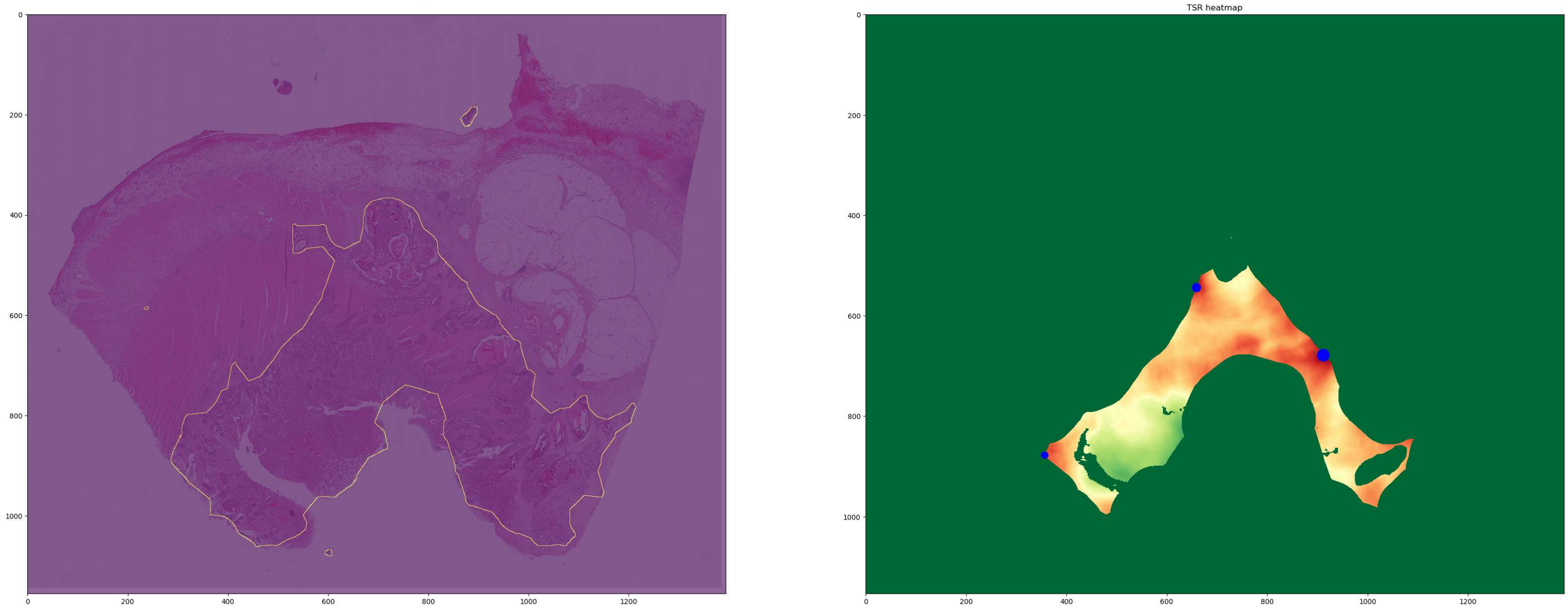

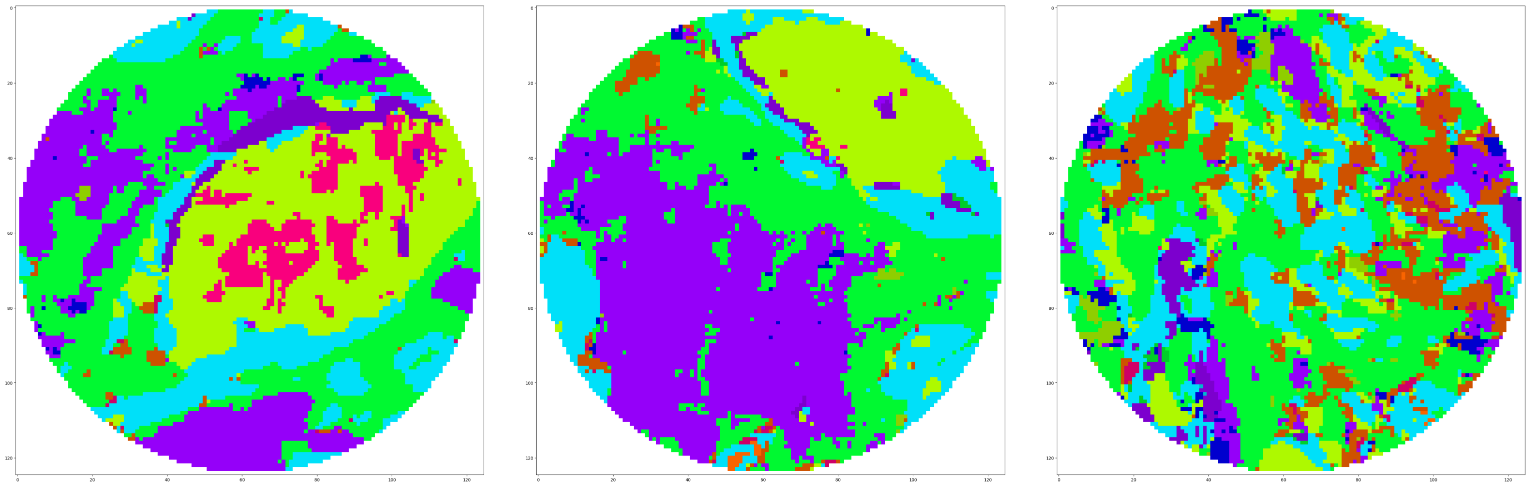

<u>Case 29</u>

Semi-automated output

Left: H&E stained section in the spot chosen by microscopic assessment. Middle: the first step was making an segmentation output. Right the class labels can be displayed

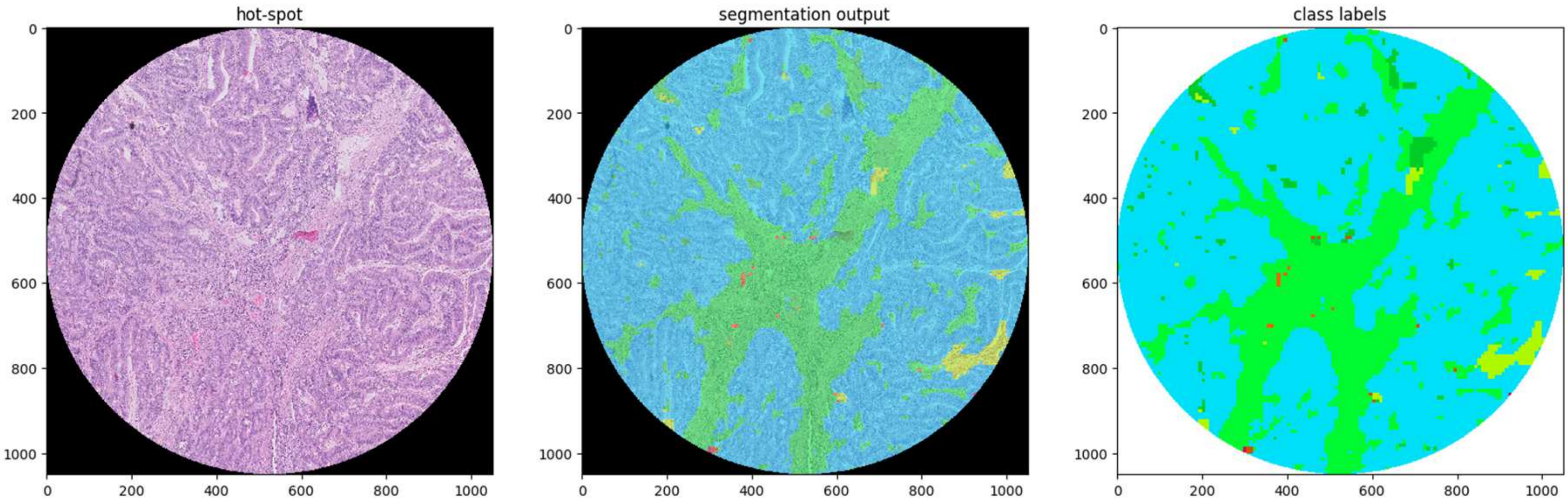

Fully-automated output

Top row; left: the tumor bulk is annotated. Right: heatmap is created. The biggest dot corresponds with the highest stroma-percentage (TSR-1), the second biggest with the second highest (TSR-2), etcetera.

Bottom row; left: the class output of the highest spot (TSR-1), middle: the second highest spot (TSR-2) and right the third highest spot (TSR-3)

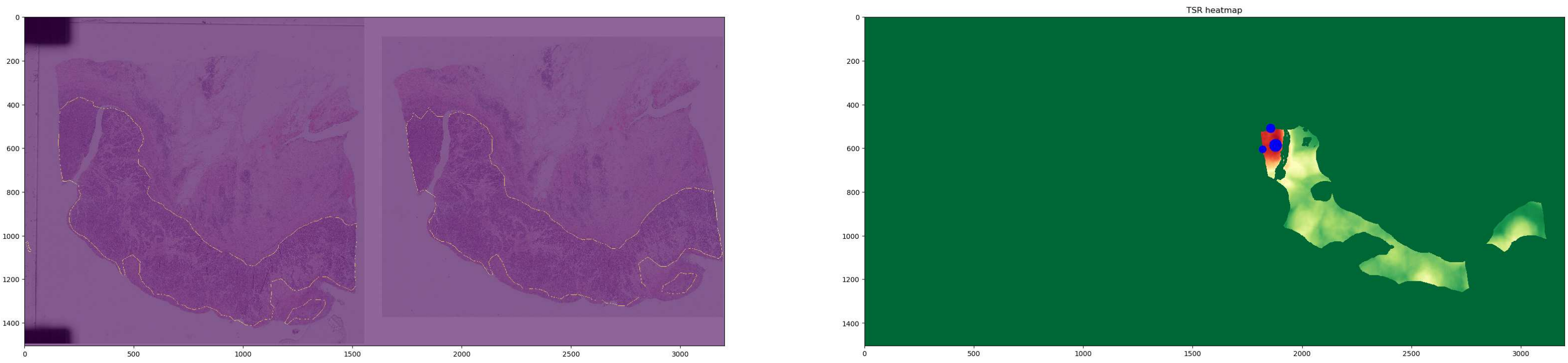

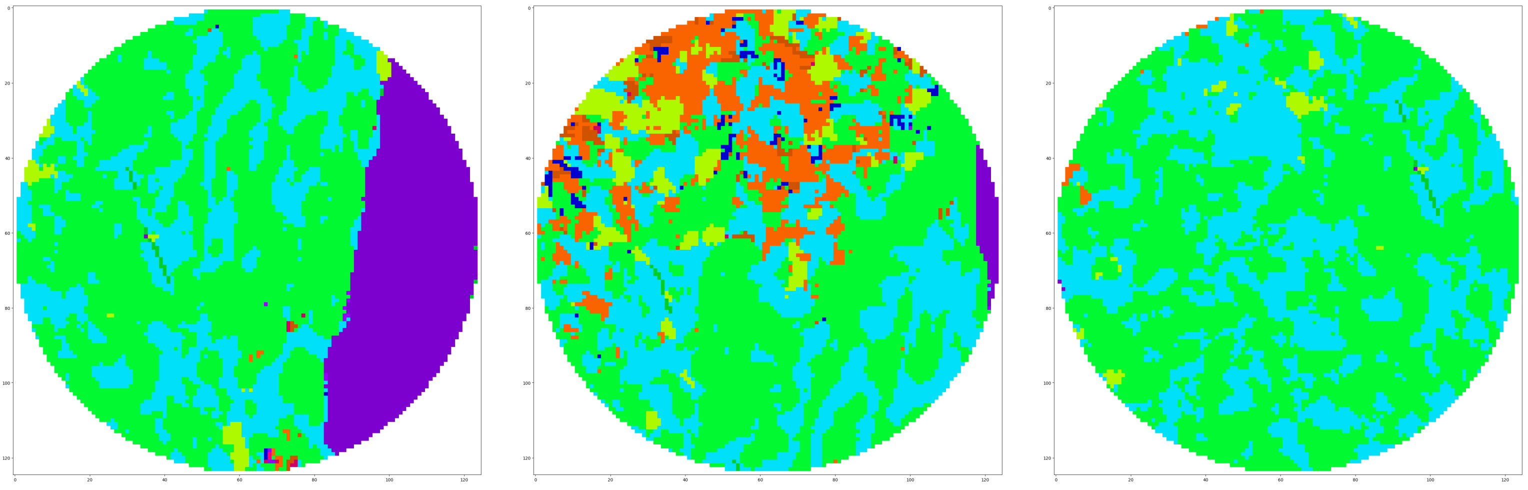

<u>Case 30</u>

Semi-automated output

Left: H&E stained section in the spot chosen by microscopic assessment. Middle: the first step was making an segmentation output. Right the class labels can be displayed

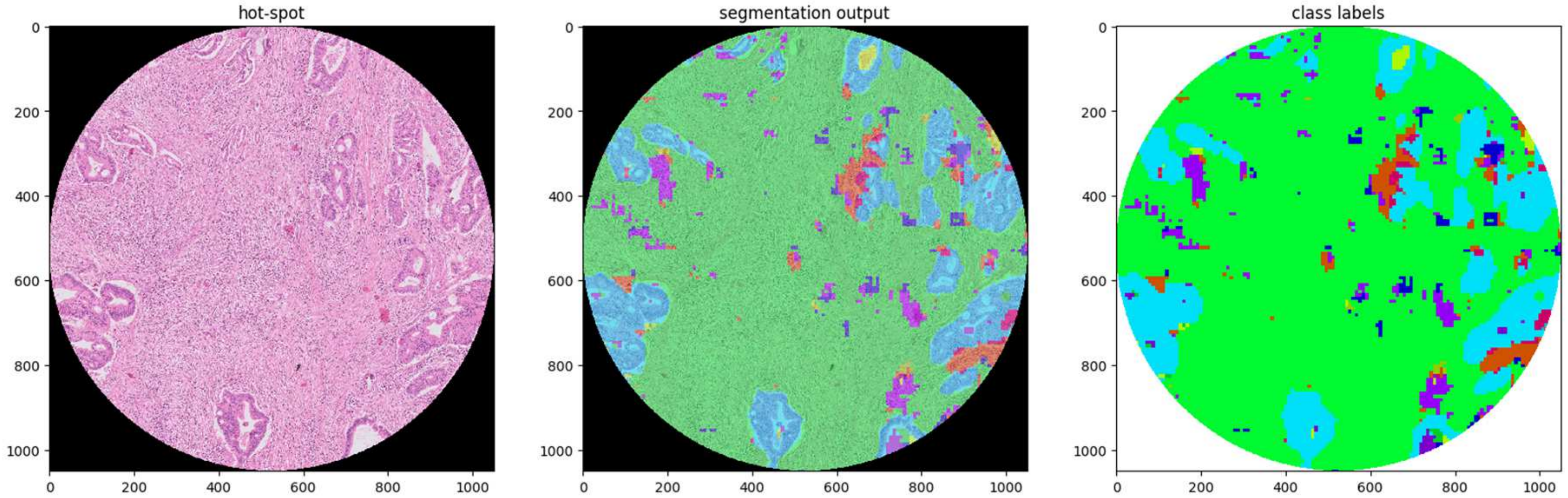

Fully-automated output

Top row; left: the tumor bulk is annotated. Right: heatmap is created. The biggest dot corresponds with the highest stroma-percentage (TSR-1), the second biggest with the second highest (TSR-2), etcetera.

Bottom row; left: the class output of the highest spot (TSR-1), middle: the second highest spot (TSR-2) and right the third highest spot (TSR-3)

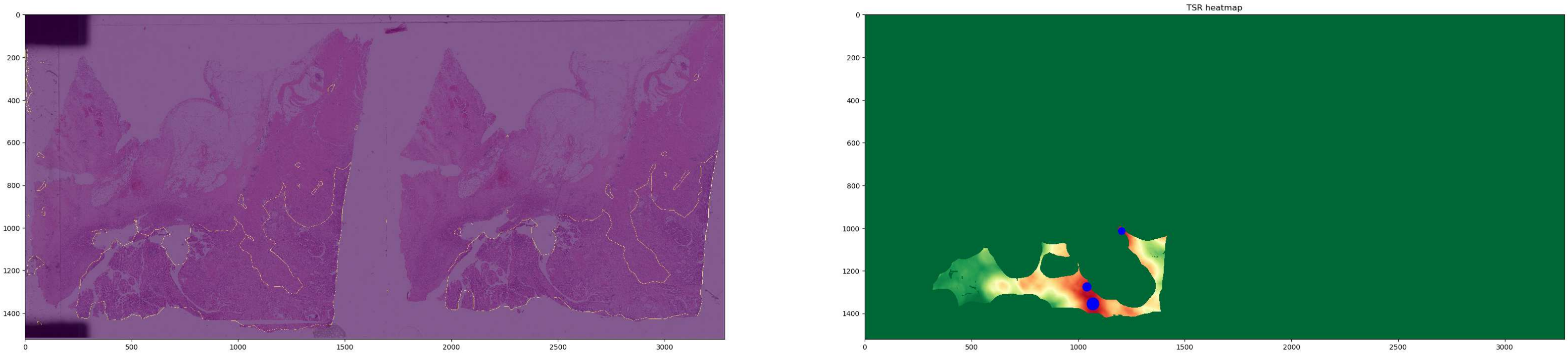

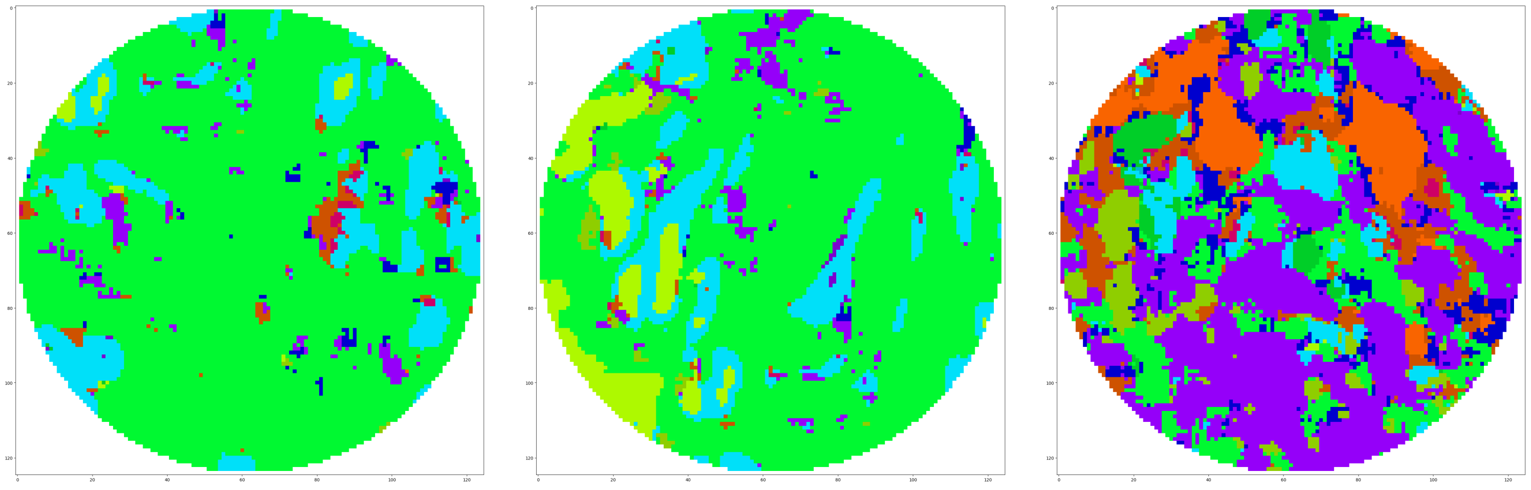

<u>Case 31</u>

Semi-automated output

Left: H&E stained section in the spot chosen by microscopic assessment. Middle: the first step was making an segmentation output. Right the class labels can be displayed

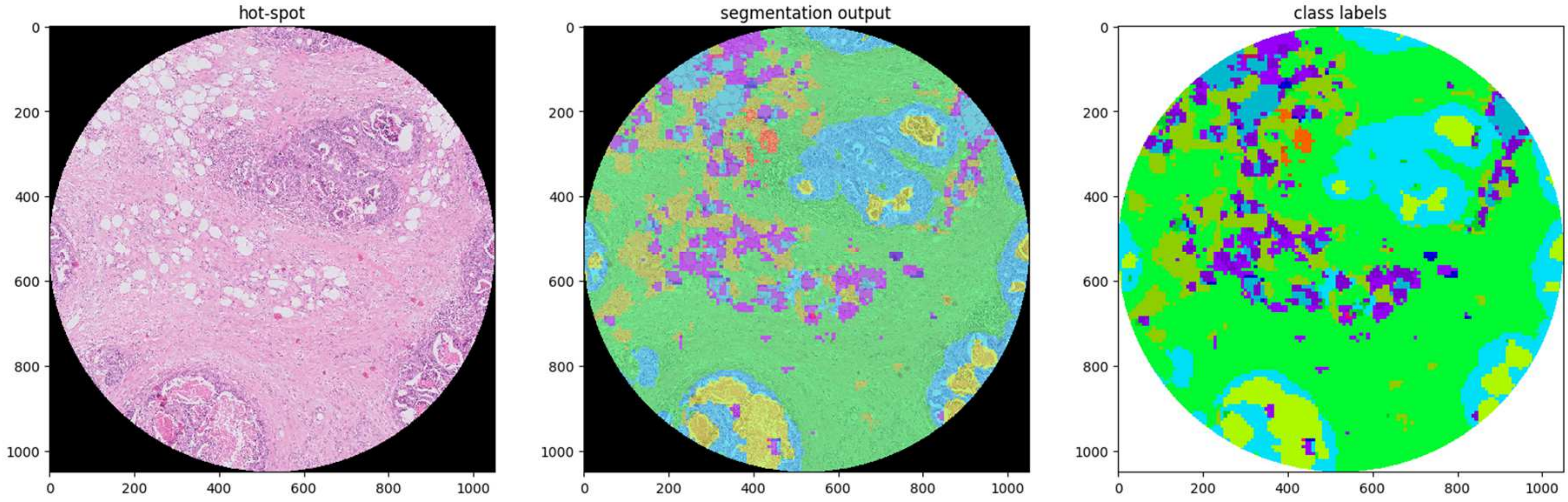

Fully-automated output

Top row; left: the tumor bulk is annotated. Right: heatmap is created. The biggest dot corresponds with the highest stroma-percentage (TSR-1), the second biggest with the second highest (TSR-2), etcetera.

Bottom row; left: the class output of the highest spot (TSR-1), middle: the second highest spot (TSR-2) and right the third highest spot (TSR-3)

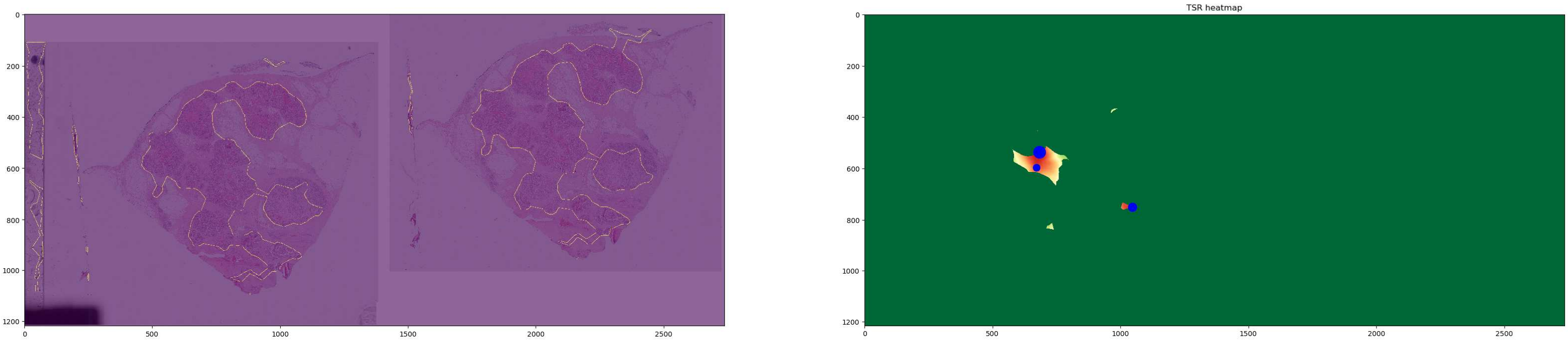

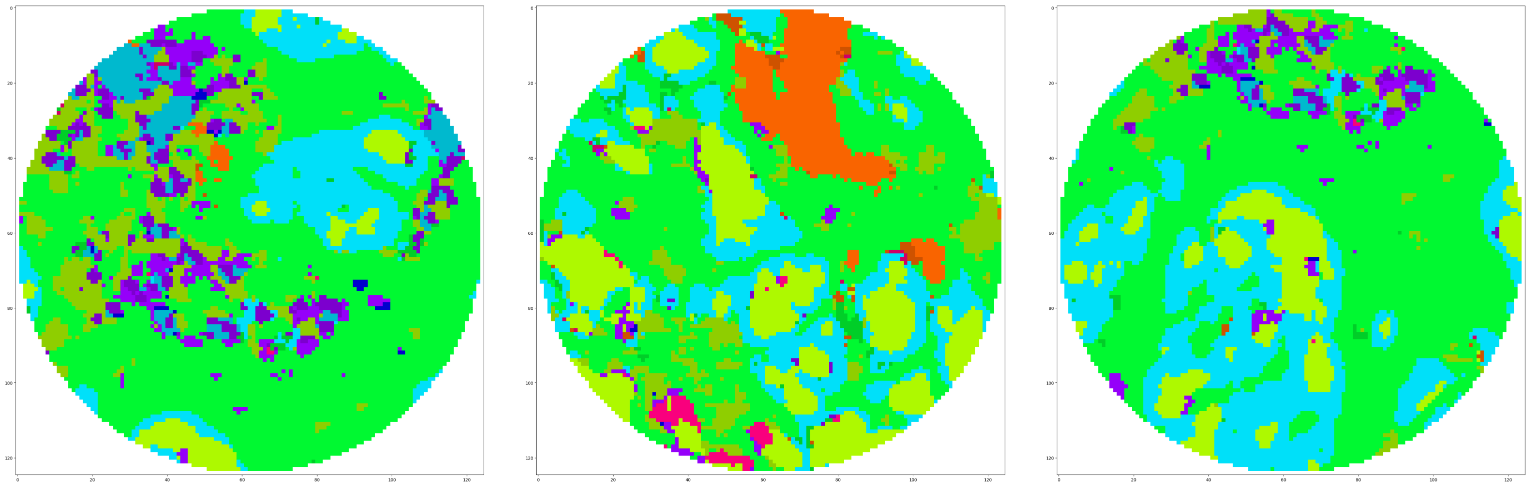

Case 32

Semi-automated output

Left: H&E stained section in the spot chosen by microscopic assessment. Middle: the first step was making an segmentation output. Right the class labels can be displayed

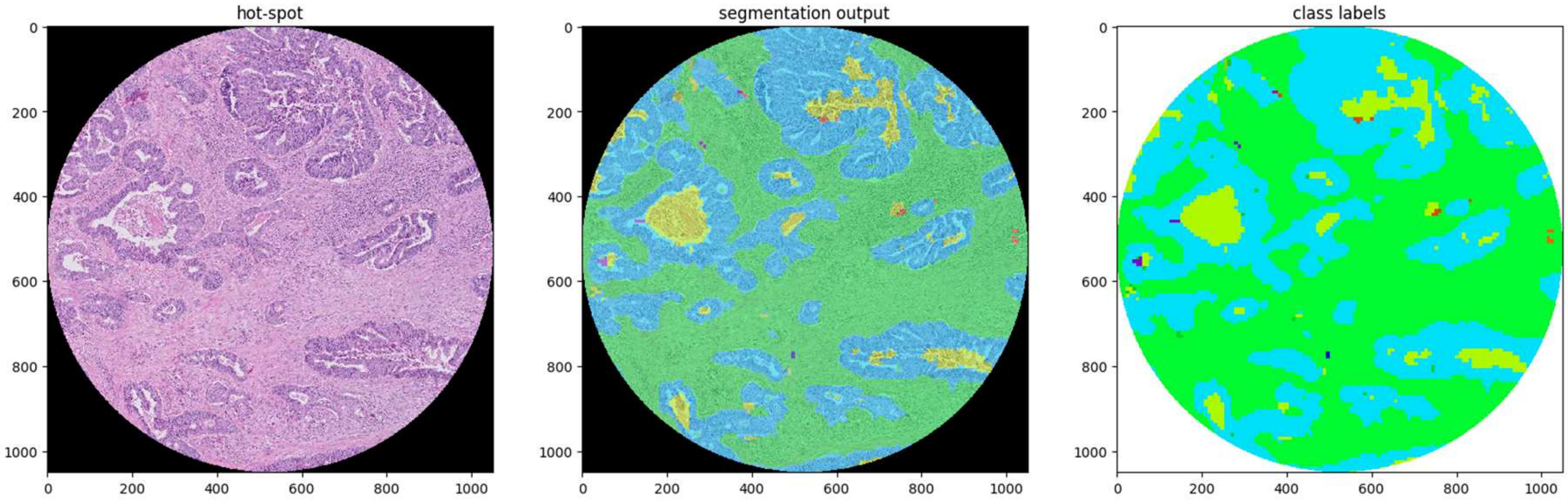

Fully-automated output

Top row; left: the tumor bulk is annotated. Right: heatmap is created. The biggest dot corresponds with the highest stroma-percentage (TSR-1), the second biggest with the second highest (TSR-2), etcetera.

Bottom row; left: the class output of the highest spot (TSR-1), middle: the second highest spot (TSR-2) and right the third highest spot (TSR-3)

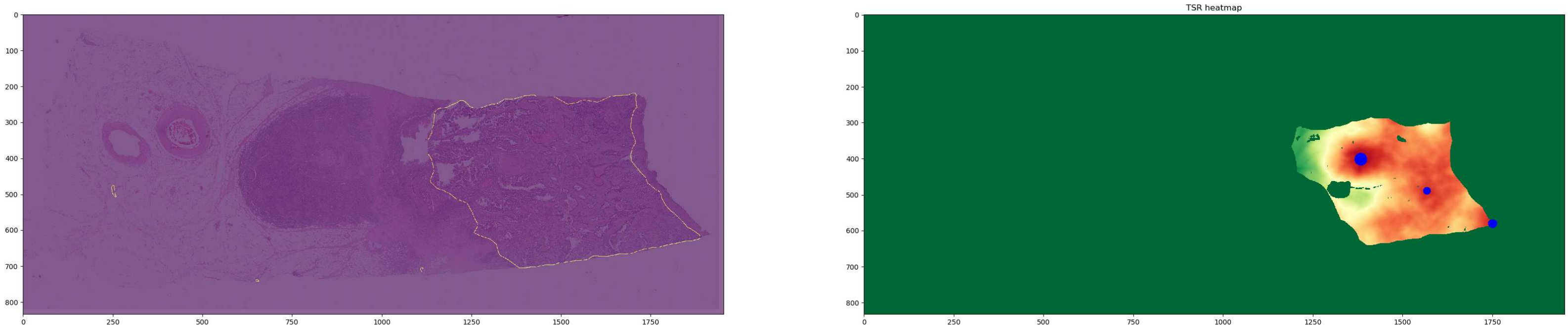

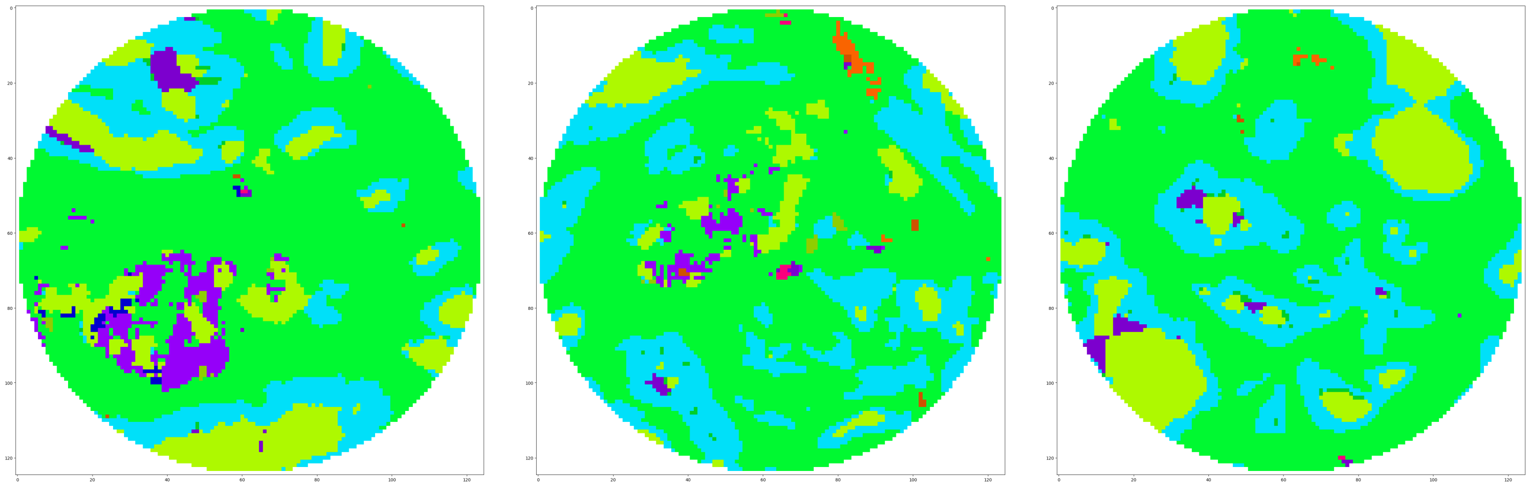

<u>Case 33</u>

Semi-automated output

Left: H&E stained section in the spot chosen by microscopic assessment. Middle: the first step was making an segmentation output. Right the class labels can be displayed

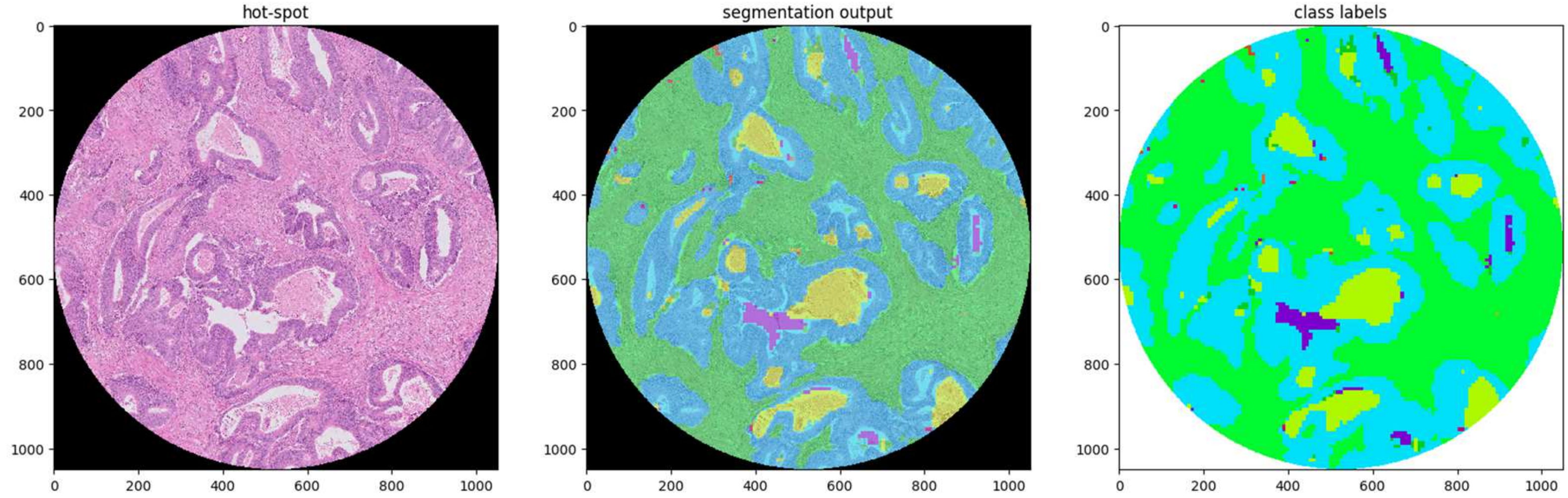

Fully-automated output

Top row; left: the tumor bulk is annotated. Right: heatmap is created. The biggest dot corresponds with the highest stroma-percentage (TSR-1), the second biggest with the second highest (TSR-2), etcetera.

Bottom row; left: the class output of the highest spot (TSR-1), middle: the second highest spot (TSR-2) and right the third highest spot (TSR-3)

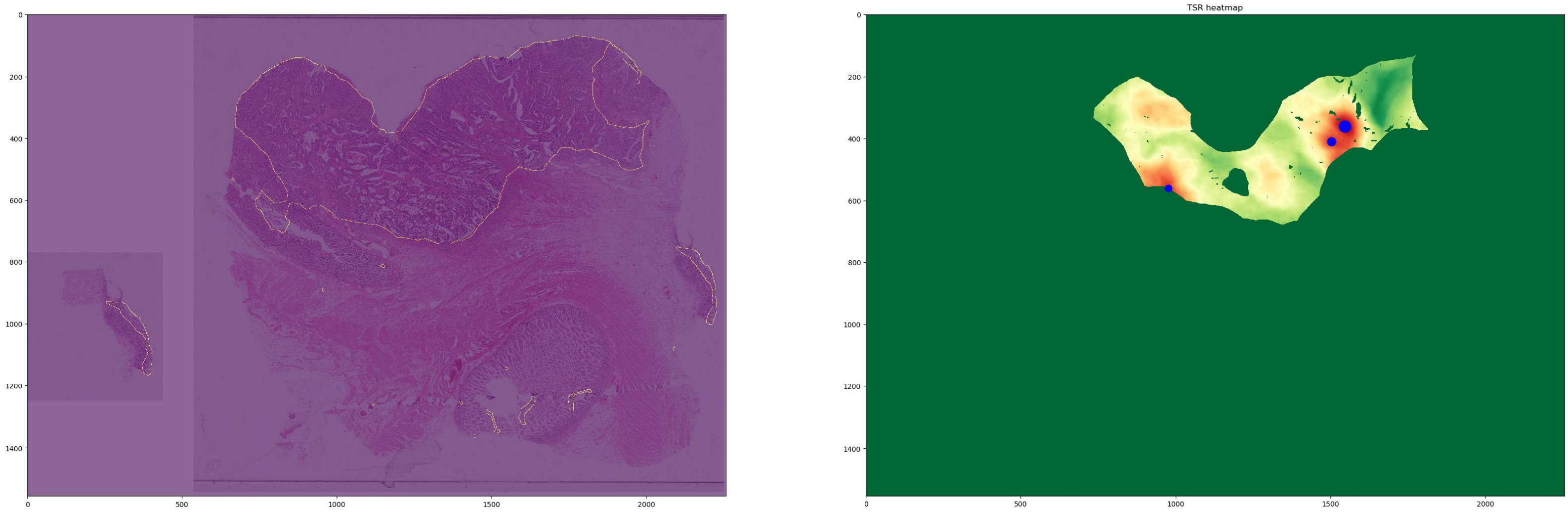

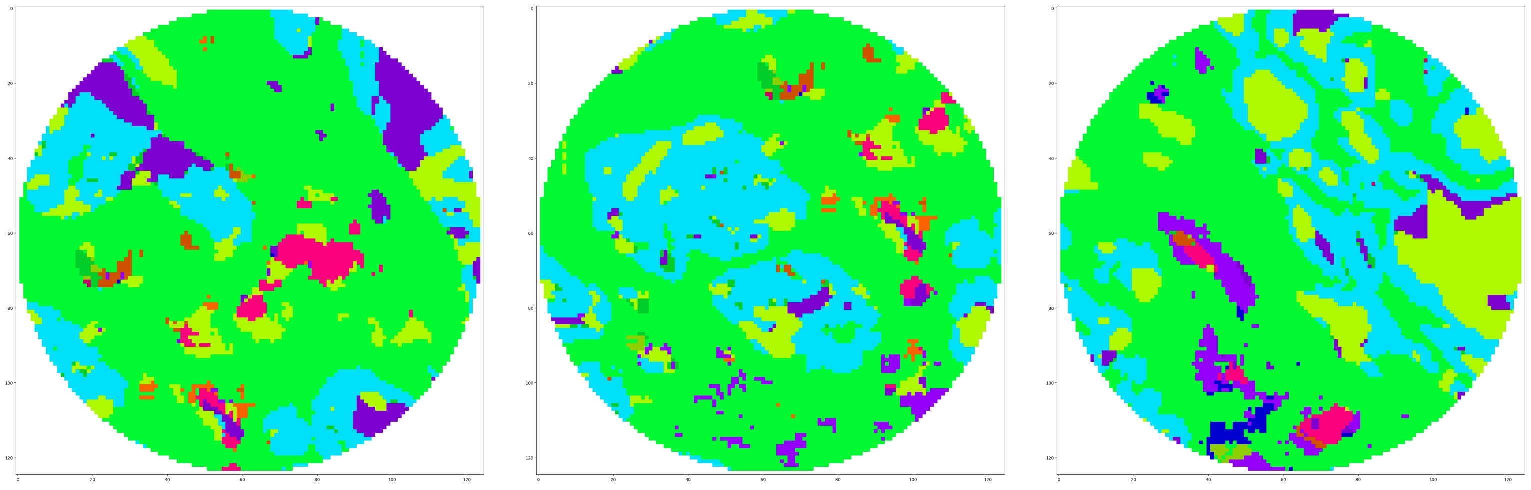

Case 34

Semi-automated output

Left: H&E stained section in the spot chosen by microscopic assessment. Middle: the first step was making an segmentation output. Right the class labels can be displayed

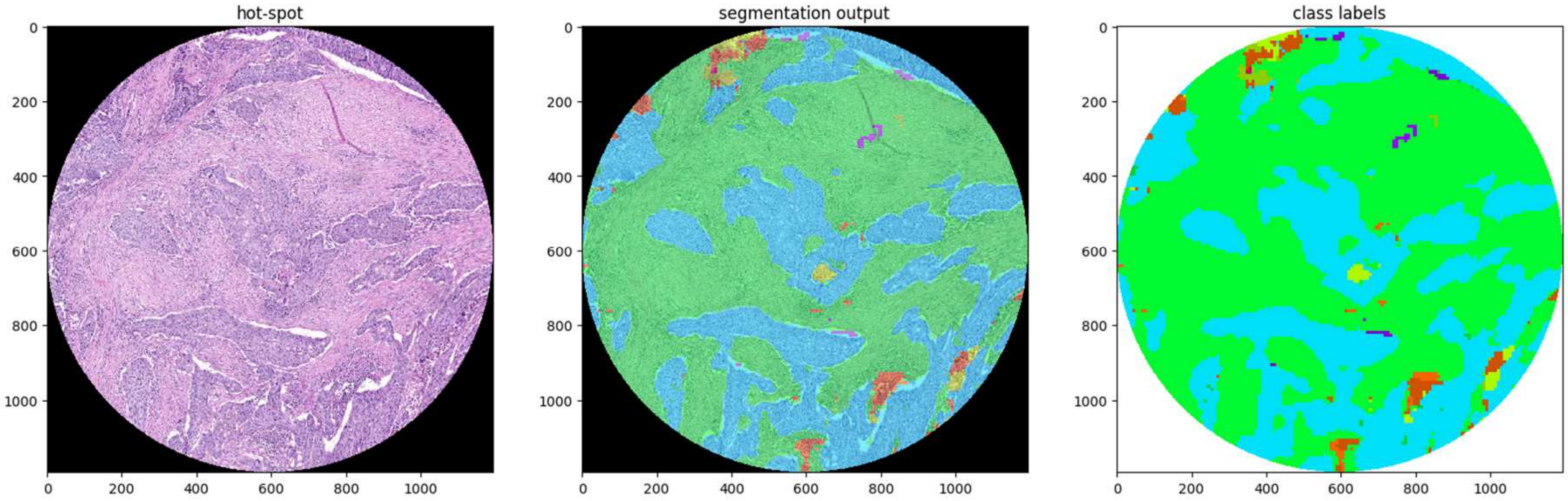

Fully-automated output

Top row; left: the tumor bulk is annotated. Right: heatmap is created. The biggest dot corresponds with the highest stroma-percentage (TSR-1), the second biggest with the second highest (TSR-2), etcetera.

Bottom row; left: the class output of the highest spot (TSR-1), middle: the second highest spot (TSR-2) and right the third highest spot (TSR-3)

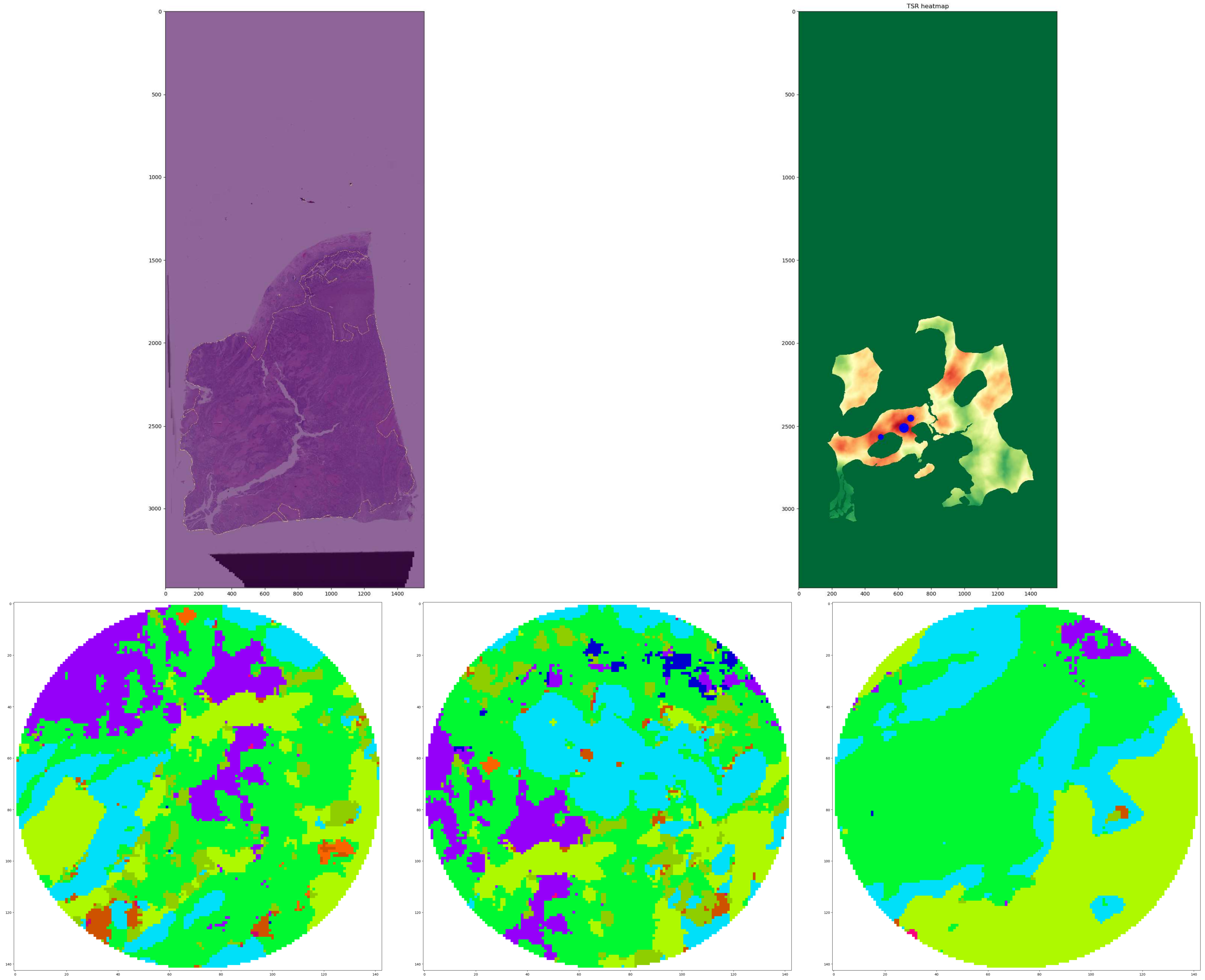

Case 35

Semi-automated output

Left: H&E stained section in the spot chosen by microscopic assessment. Middle: the first step was making an segmentation output. Right the class labels can be displayed

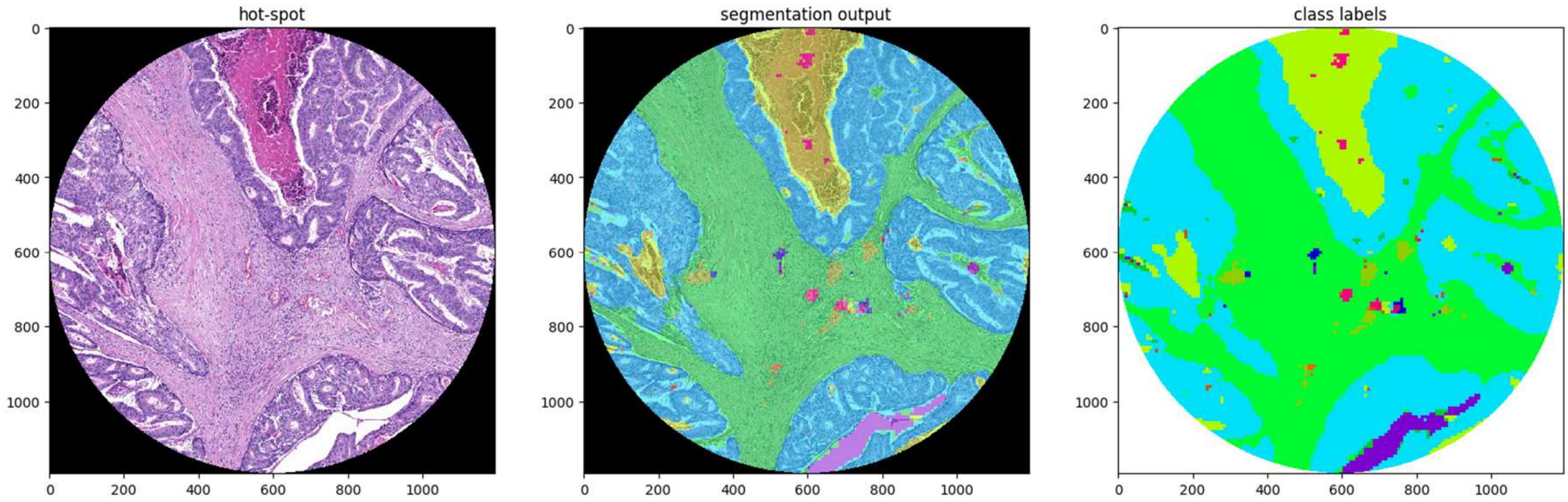

Fully-automated output

Top row; left: the tumor bulk is annotated. Right: heatmap is created. The biggest dot corresponds with the highest stroma-percentage (TSR-1), the second biggest with the second highest (TSR-2), etcetera.

Bottom row; left: the class output of the highest spot (TSR-1), middle: the second highest spot (TSR-2) and right the third highest spot (TSR-3)

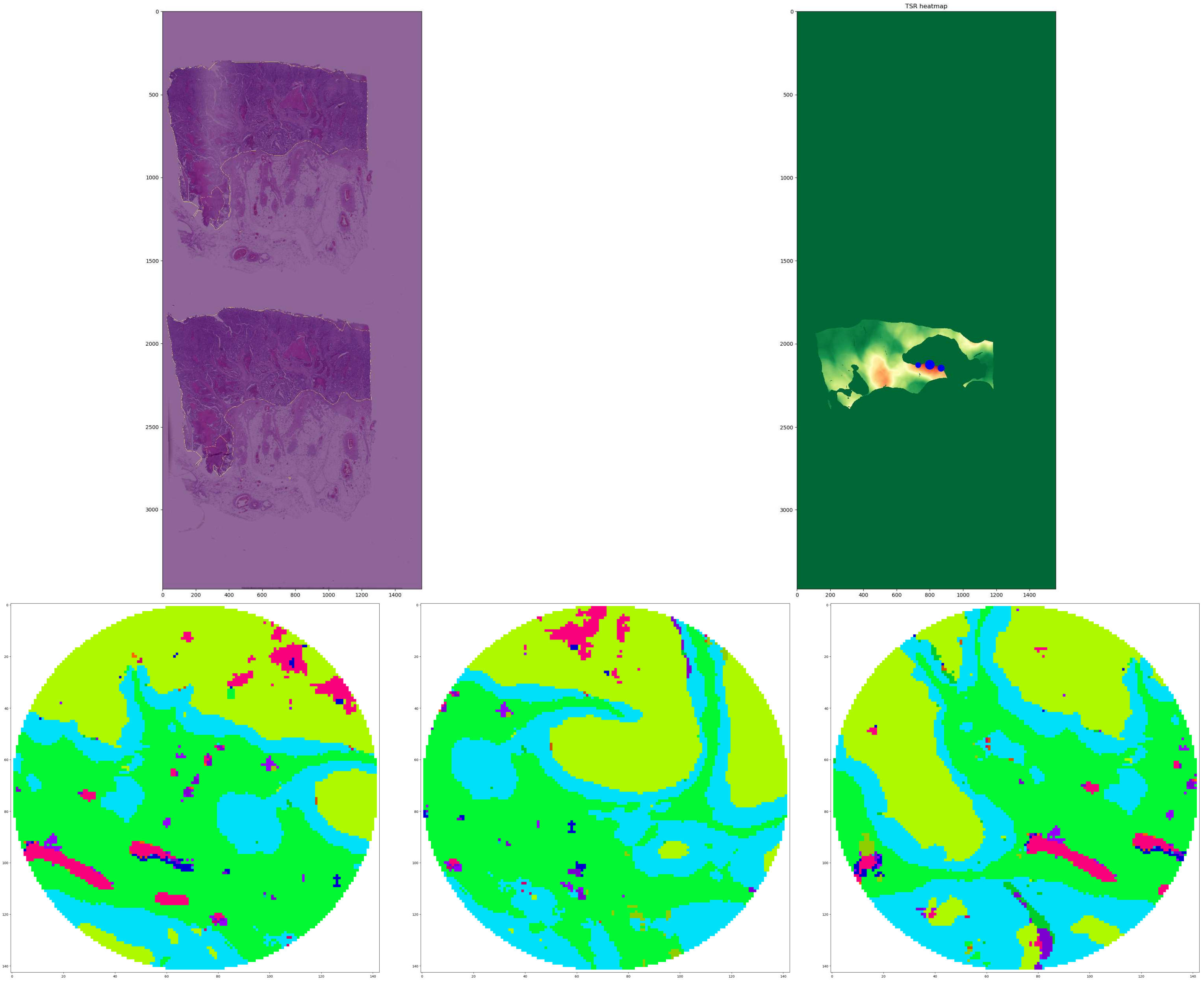

<u>Case 36</u>

Semi-automated output

Left: H&E stained section in the spot chosen by microscopic assessment. Middle: the first step was making an segmentation output. Right the class labels can be displayed

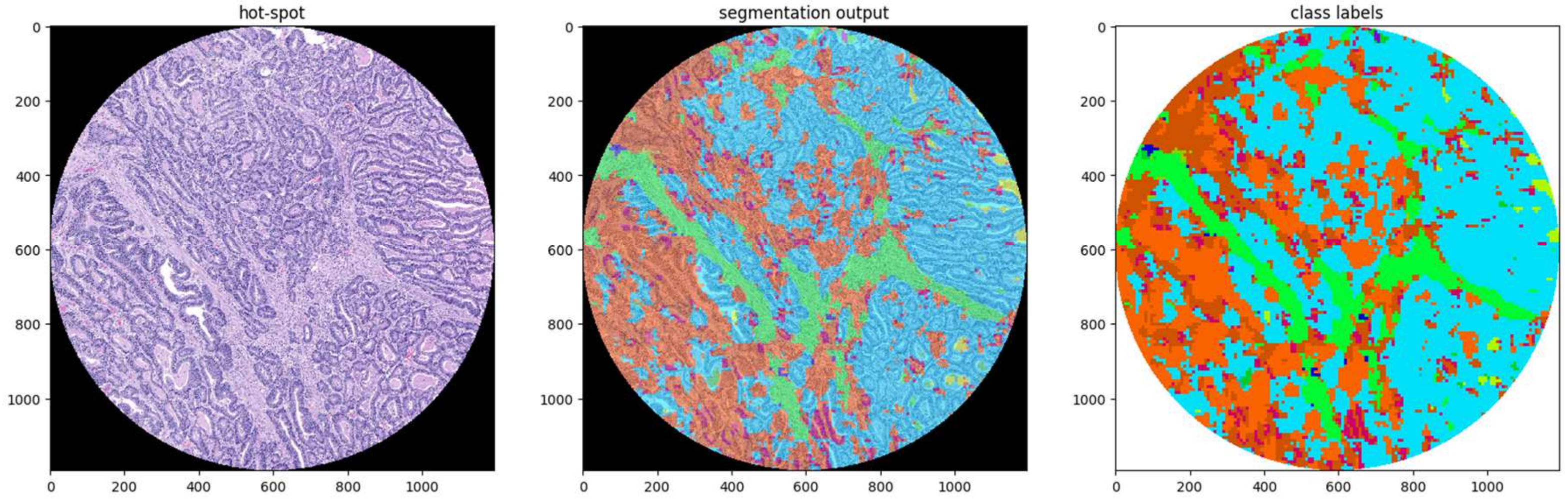

Fully-automated output

Top row; left: the tumor bulk is annotated. Right: heatmap is created. The biggest dot corresponds with the highest stroma-percentage (TSR-1), the second biggest with the second highest (TSR-2), etcetera.

Bottom row; left: the class output of the highest spot (TSR-1), middle: the second highest spot (TSR-2) and right the third highest spot (TSR-3)

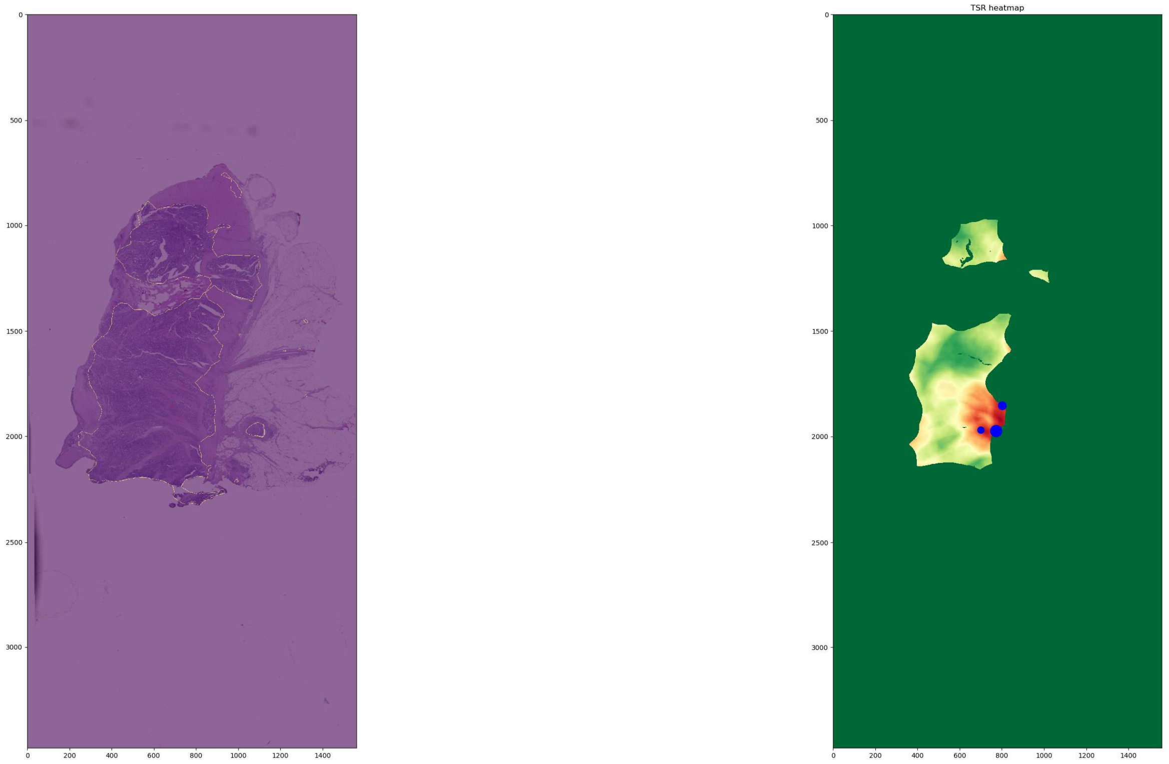

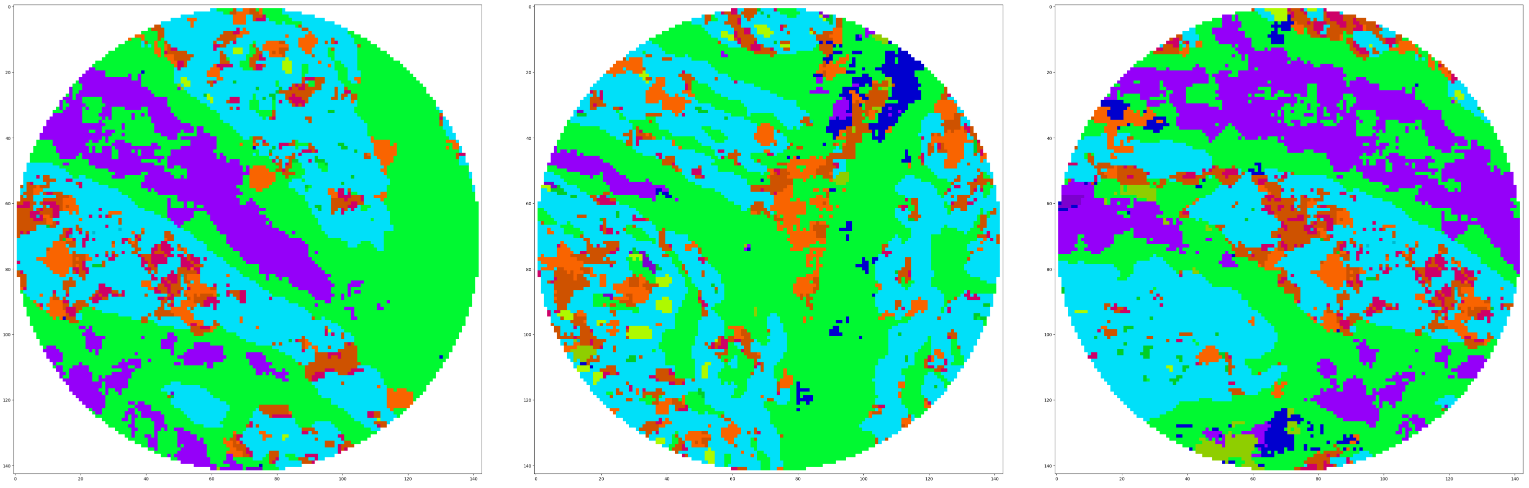

Case 37

Semi-automated output

Left: H&E stained section in the spot chosen by microscopic assessment. Middle: the first step was making an segmentation output. Right the class labels can be displayed

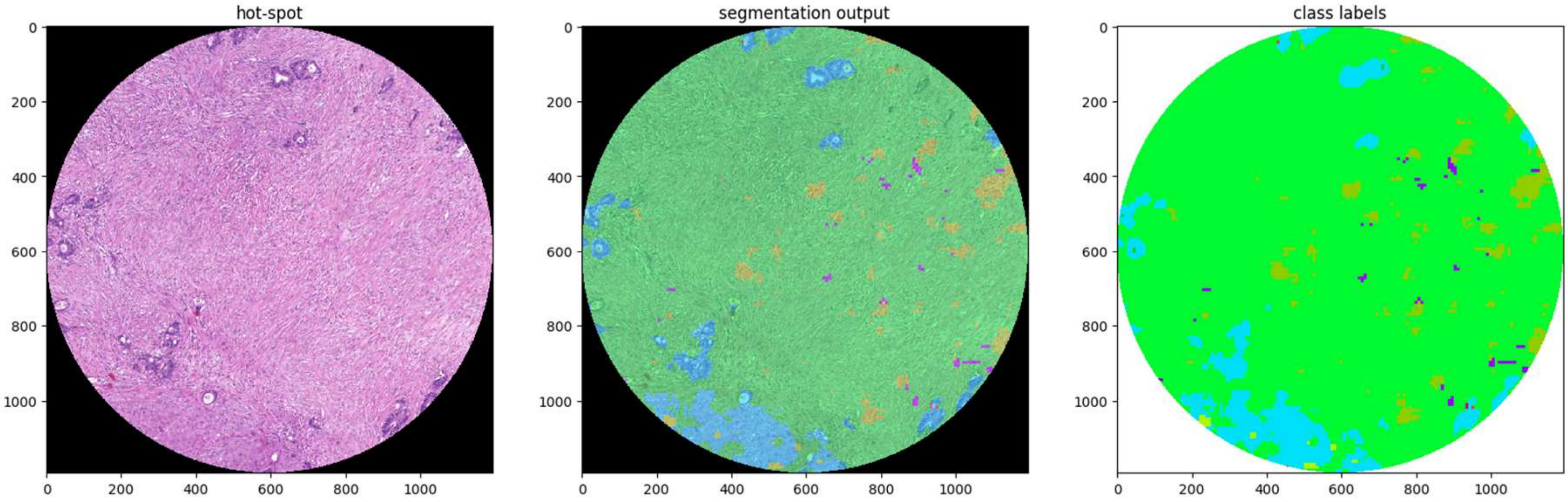

Fully-automated output

Top row; left: the tumor bulk is annotated. Right: heatmap is created. The biggest dot corresponds with the highest stroma-percentage (TSR-1), the second biggest with the second highest (TSR-2), etcetera.

Bottom row; left: the class output of the highest spot (TSR-1), middle: the second highest spot (TSR-2) and right the third highest spot (TSR-3)

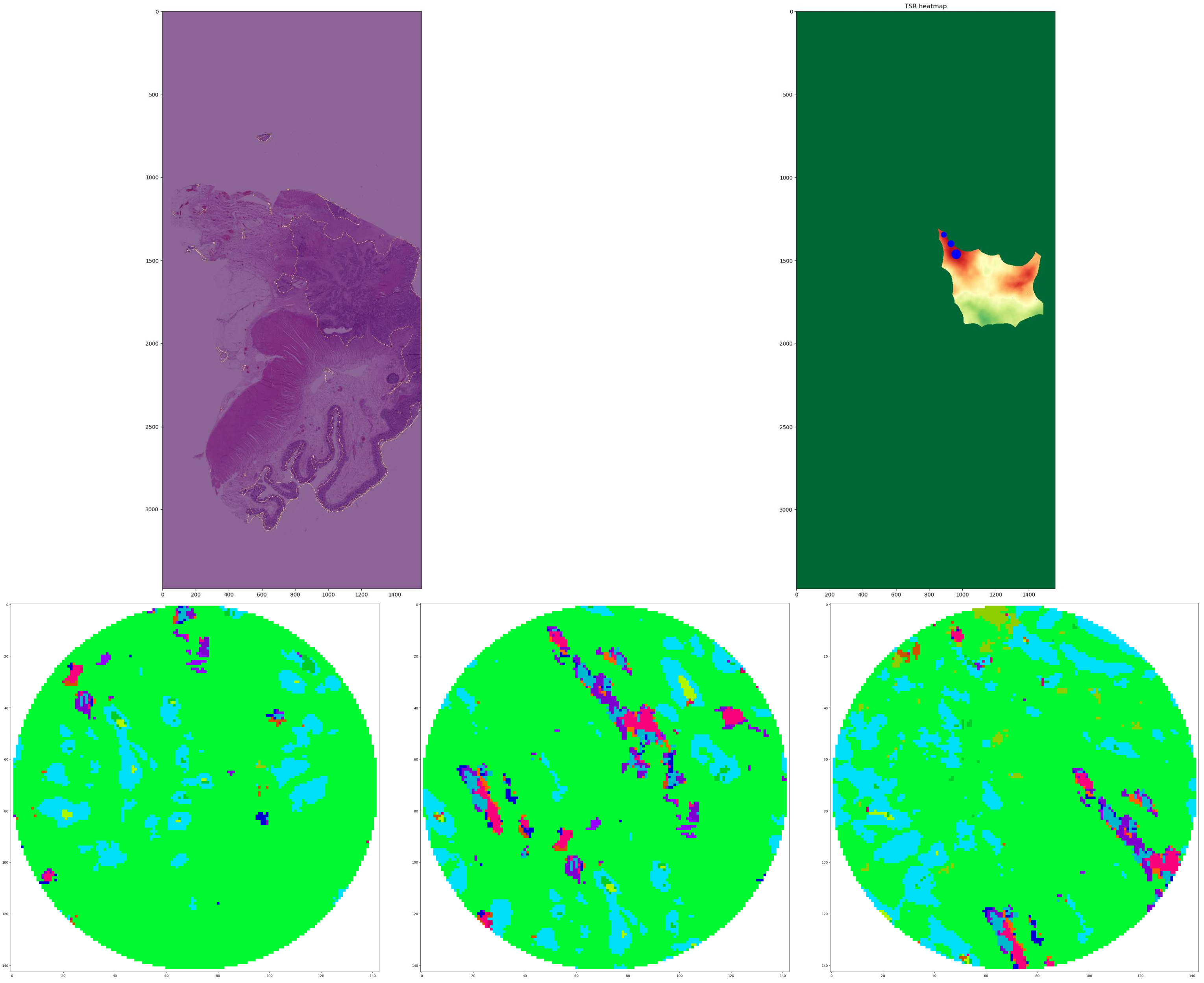

Case 38

Semi-automated output

Left: H&E stained section in the spot chosen by microscopic assessment. Middle: the first step was making an segmentation output. Right the class labels can be displayed

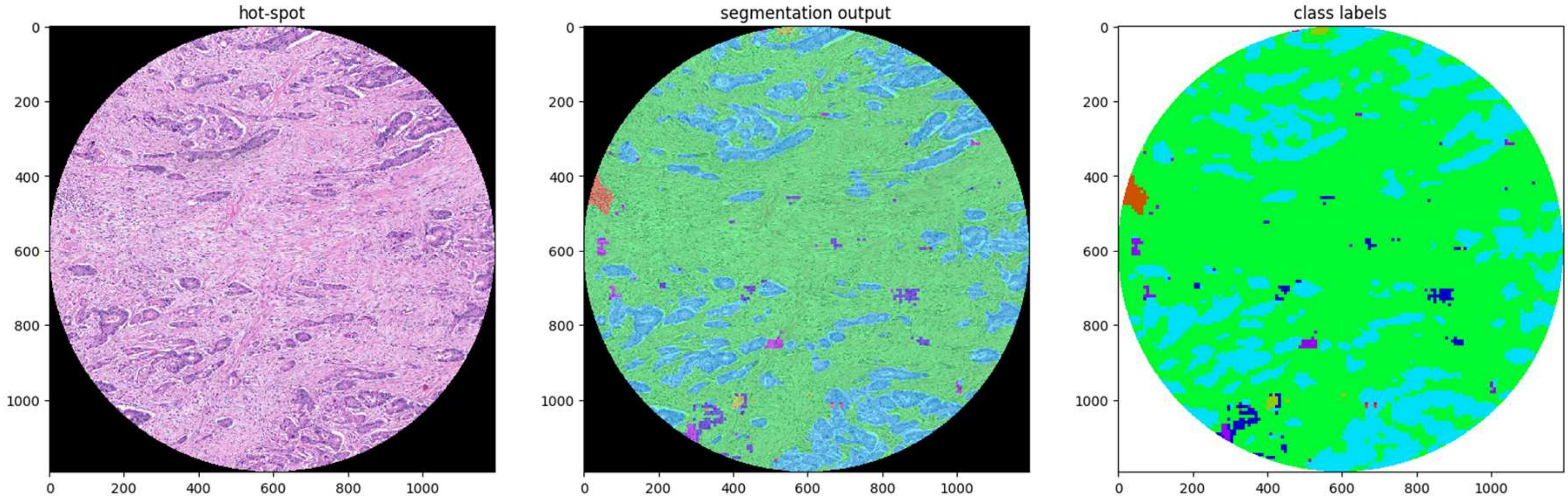

Fully-automated output

Top row; left: the tumor bulk is annotated. Right: heatmap is created. The biggest dot corresponds with the highest stroma-percentage (TSR-1), the second biggest with the second highest (TSR-2), etcetera.

Bottom row; left: the class output of the highest spot (TSR-1), middle: the second highest spot (TSR-2) and right the third highest spot (TSR-3)

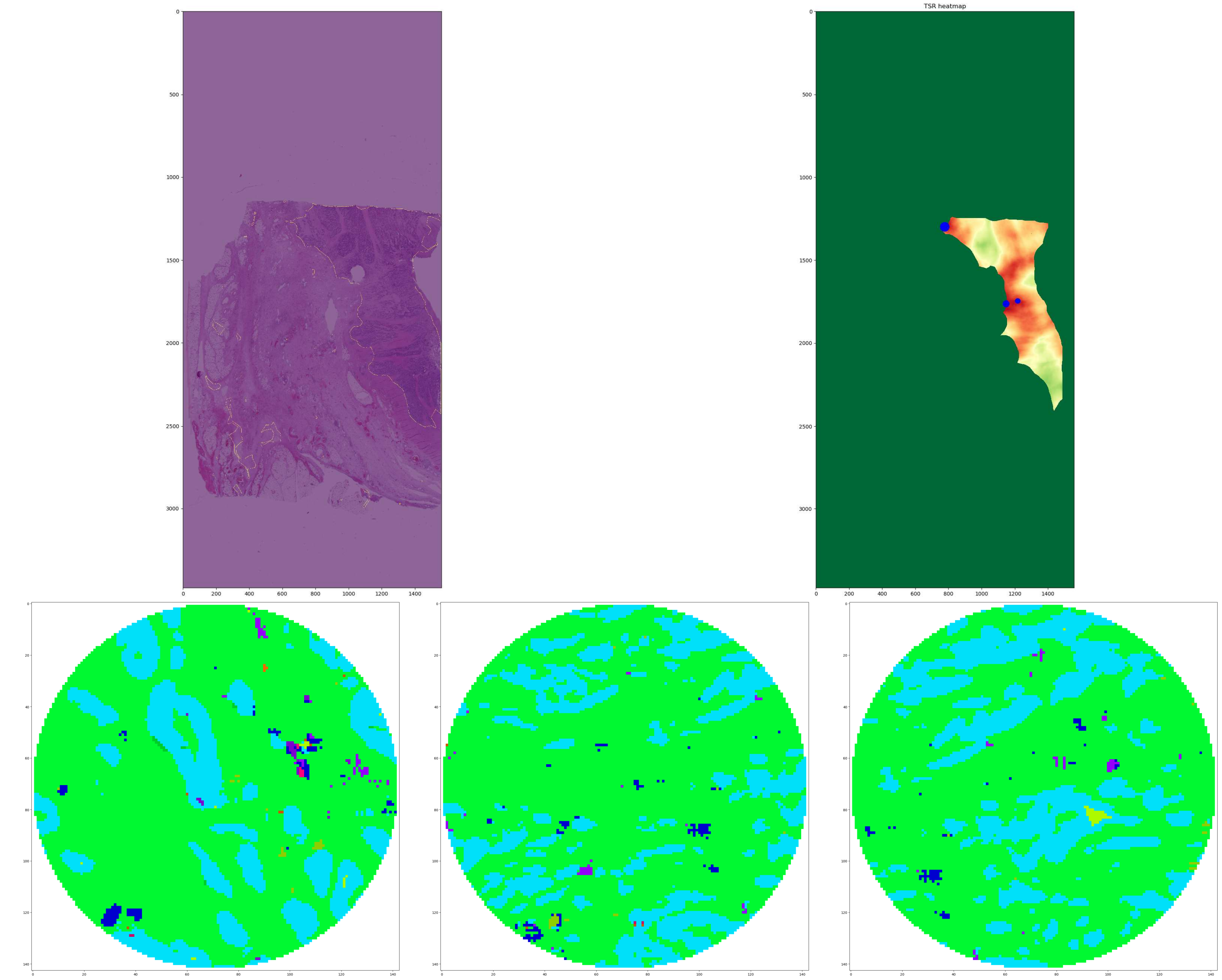

<u>Case 39</u>

Semi-automated output

Left: H&E stained section in the spot chosen by microscopic assessment. Middle: the first step was making an segmentation output. Right the class labels can be displayed

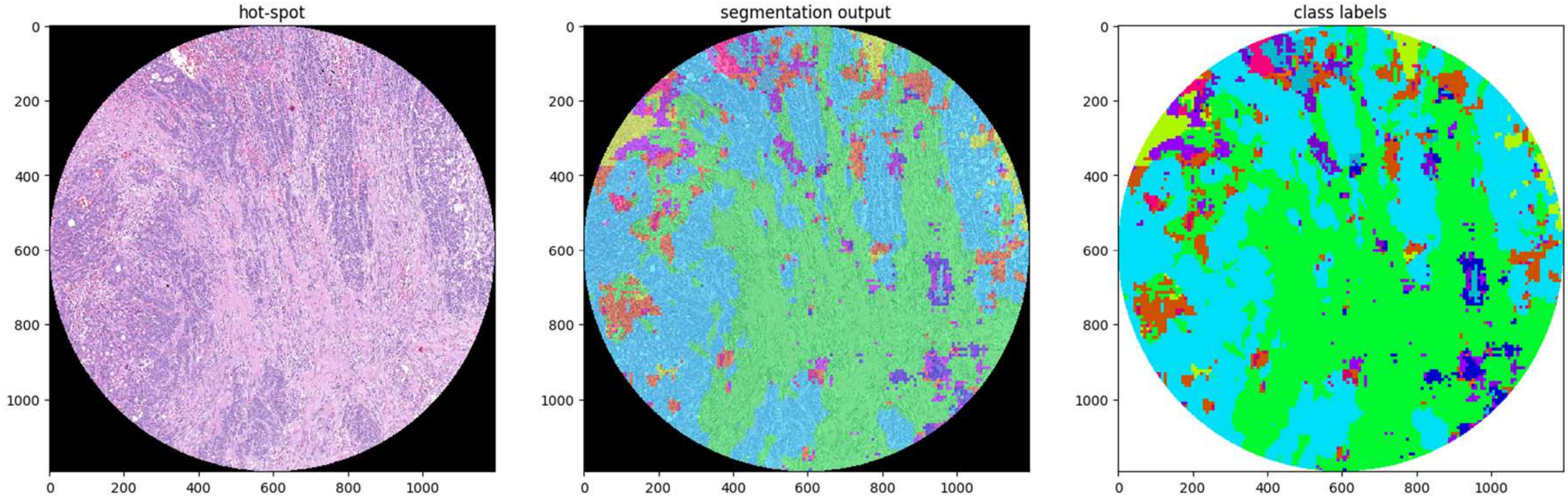

Fully-automated output

Top row; left: the tumor bulk is annotated. Right: heatmap is created. The biggest dot corresponds with the highest stroma-percentage (TSR-1), the second biggest with the second highest (TSR-2), etcetera.

Bottom row; left: the class output of the highest spot (TSR-1), middle: the second highest spot (TSR-2) and right the third highest spot (TSR-3)

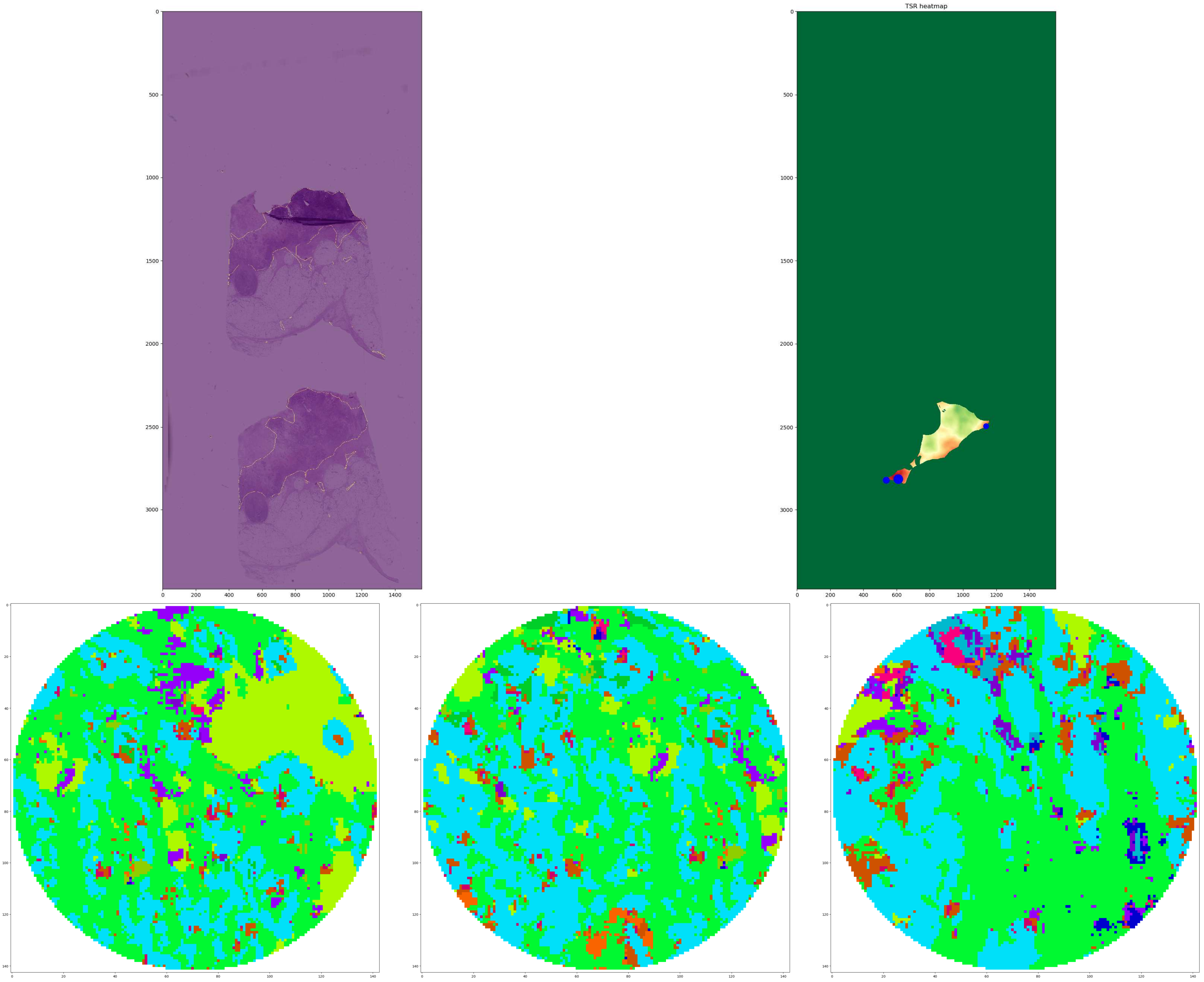

Case 40

Semi-automated output

Left: H&E stained section in the spot chosen by microscopic assessment. Middle: the first step was making an segmentation output. Right the class labels can be displayed

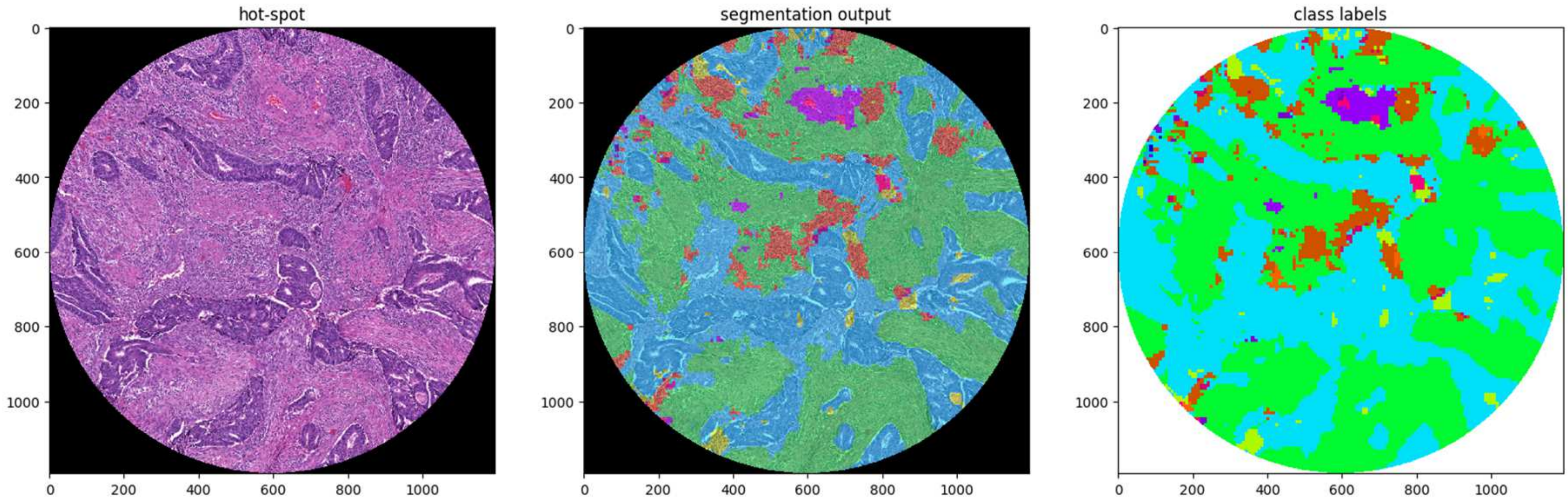

Fully-automated output

Top row; left: the tumor bulk is annotated. Right: heatmap is created. The biggest dot corresponds with the highest stroma-percentage (TSR-1), the second biggest with the second highest (TSR-2), etcetera.

Bottom row; left: the class output of the highest spot (TSR-1), middle: the second highest spot (TSR-2) and right the third highest spot (TSR-3)

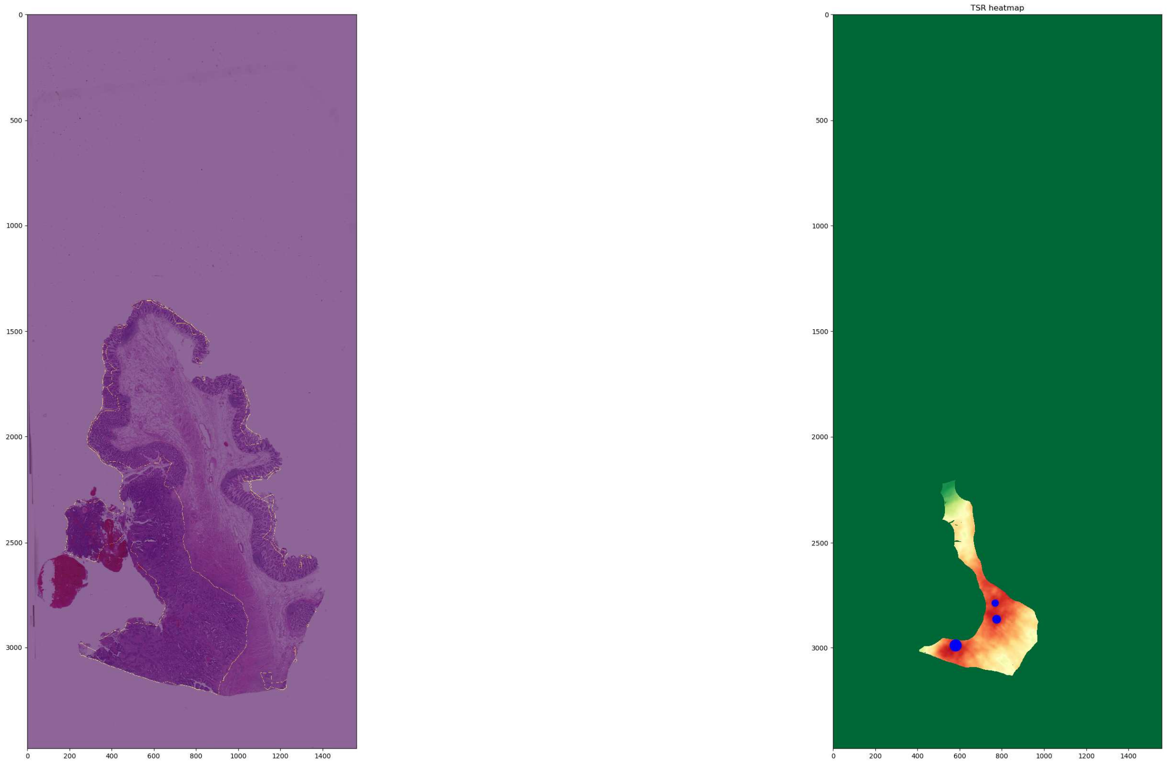

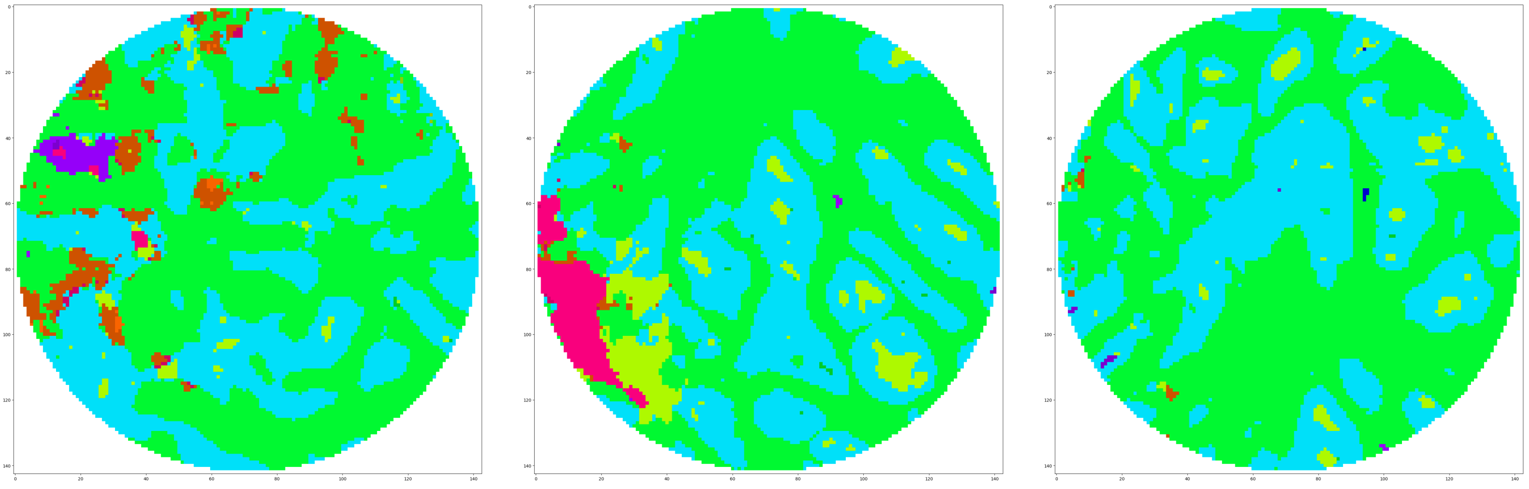

Case 41

Semi-automated output

Left: H&E stained section in the spot chosen by microscopic assessment. Middle: the first step was making an segmentation output. Right the class labels can be displayed

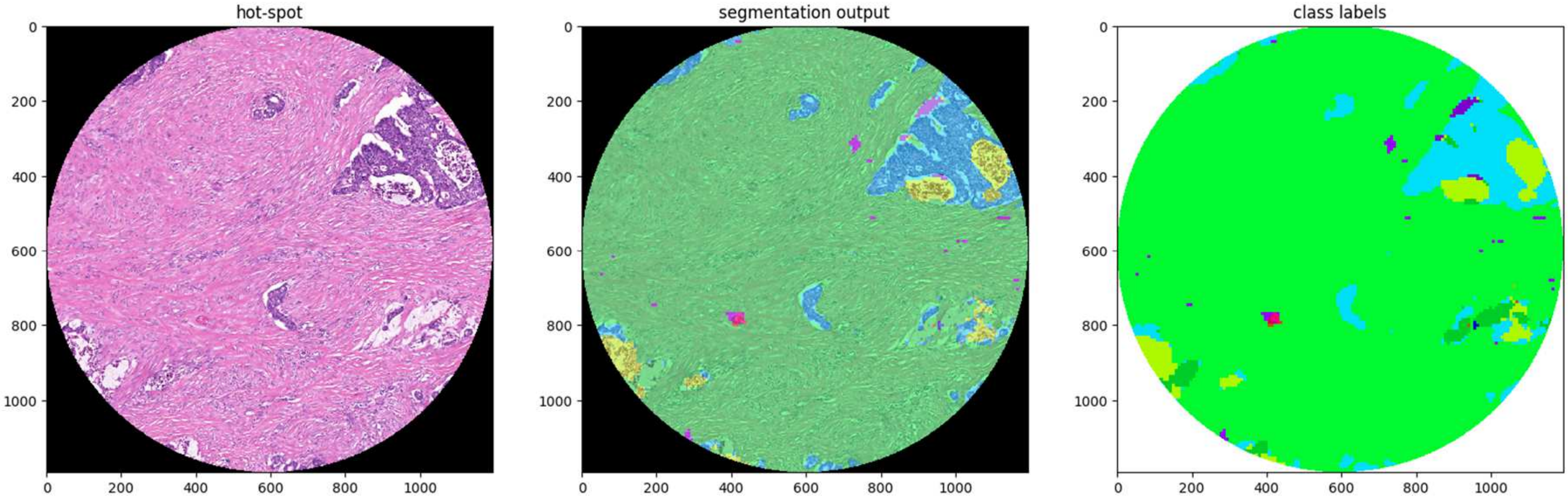

Fully-automated output

Top row; left: the tumor bulk is annotated. Right: heatmap is created. The biggest dot corresponds with the highest stroma-percentage (TSR-1), the second biggest with the second highest (TSR-2), etcetera.

Bottom row; left: the class output of the highest spot (TSR-1), middle: the second highest spot (TSR-2) and right the third highest spot (TSR-3)

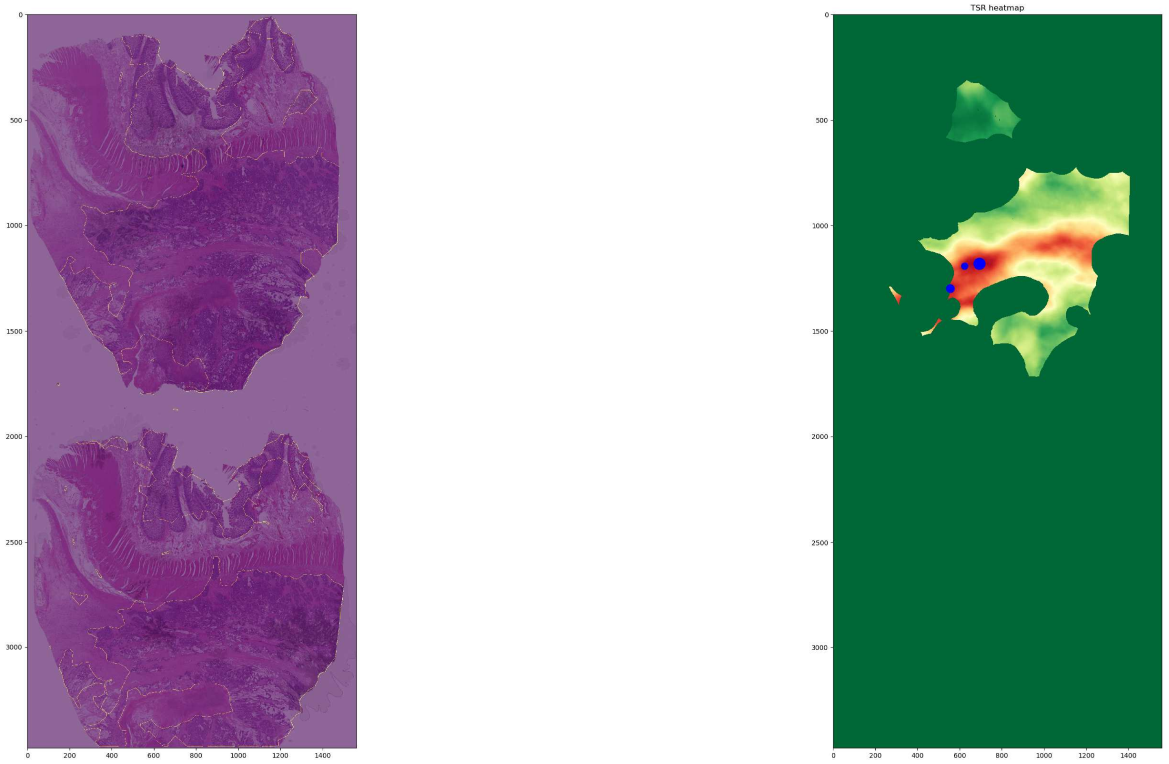

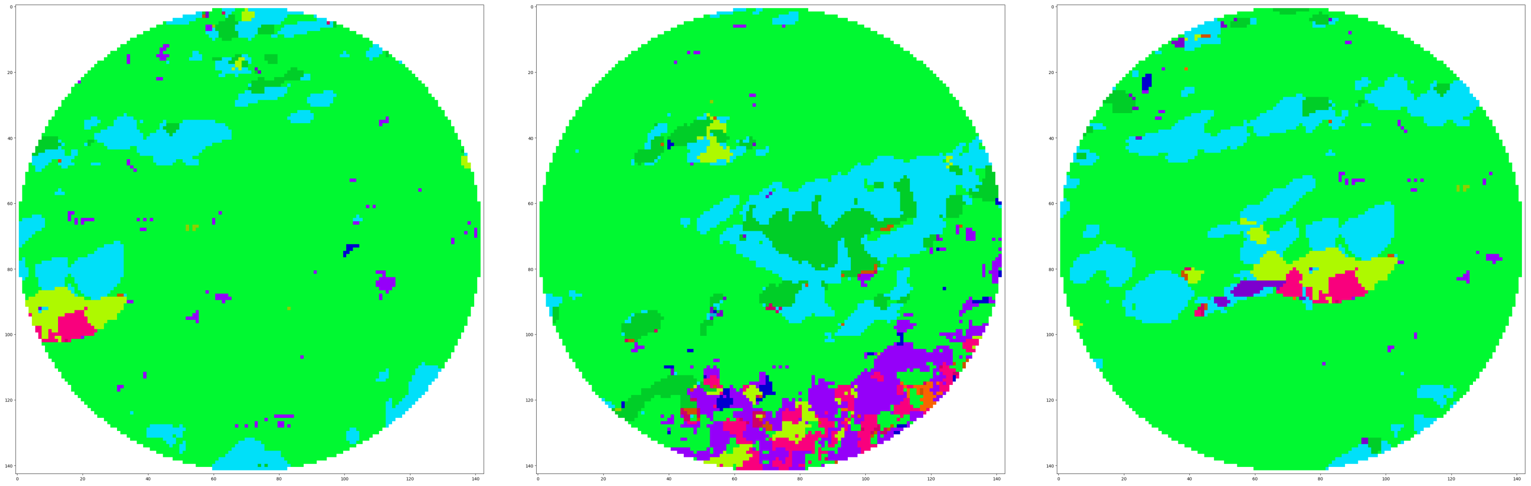

Case 42

Semi-automated output

Left: H&E stained section in the spot chosen by microscopic assessment. Middle: the first step was making an segmentation output. Right the class labels can be displayed

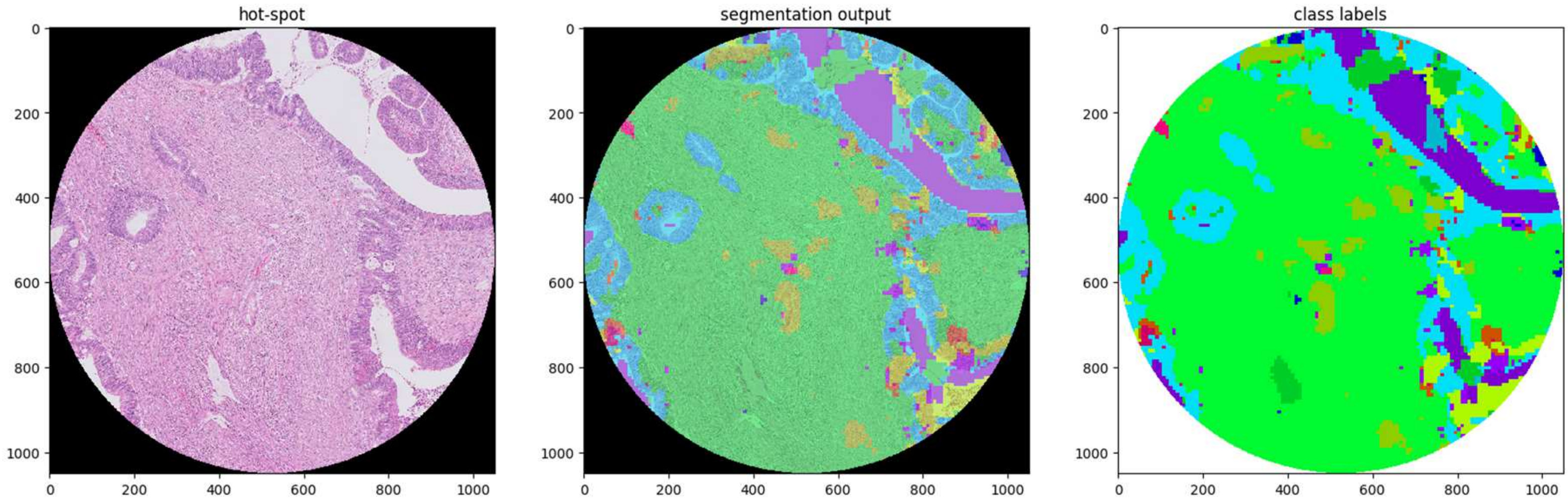

Fully-automated output

Top row; left: the tumor bulk is annotated. Right: heatmap is created. The biggest dot corresponds with the highest stroma-percentage (TSR-1), the second biggest with the second highest (TSR-2), etcetera.

Bottom row; left: the class output of the highest spot (TSR-1), middle: the second highest spot (TSR-2) and right the third highest spot (TSR-3)

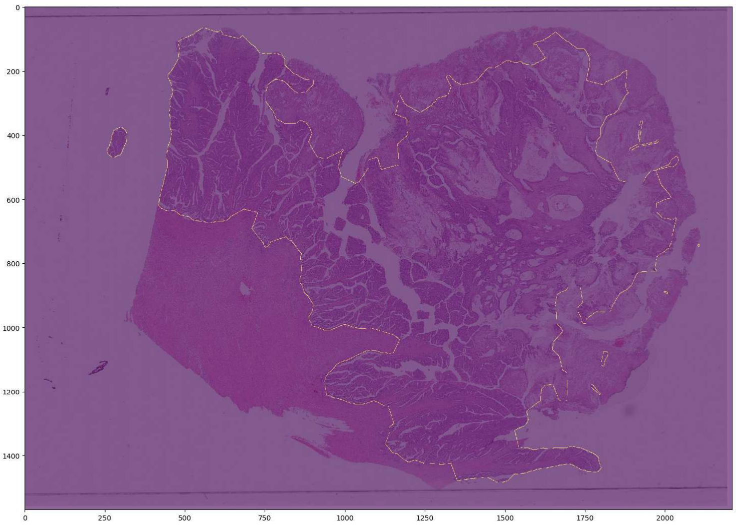

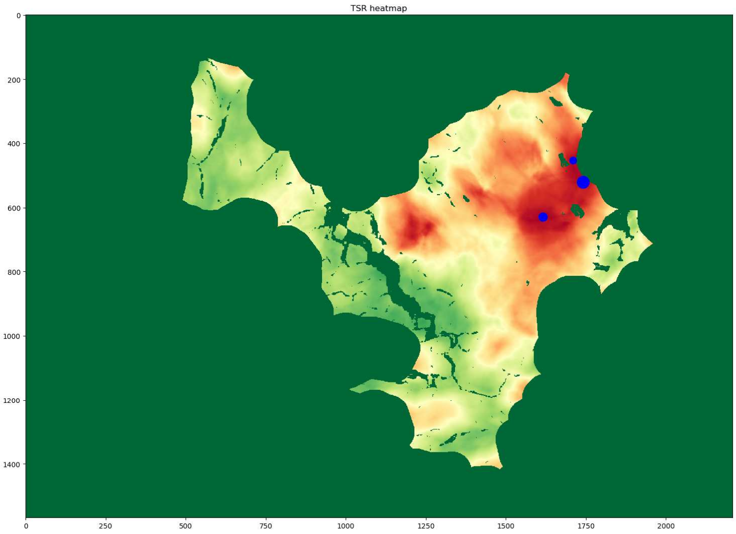

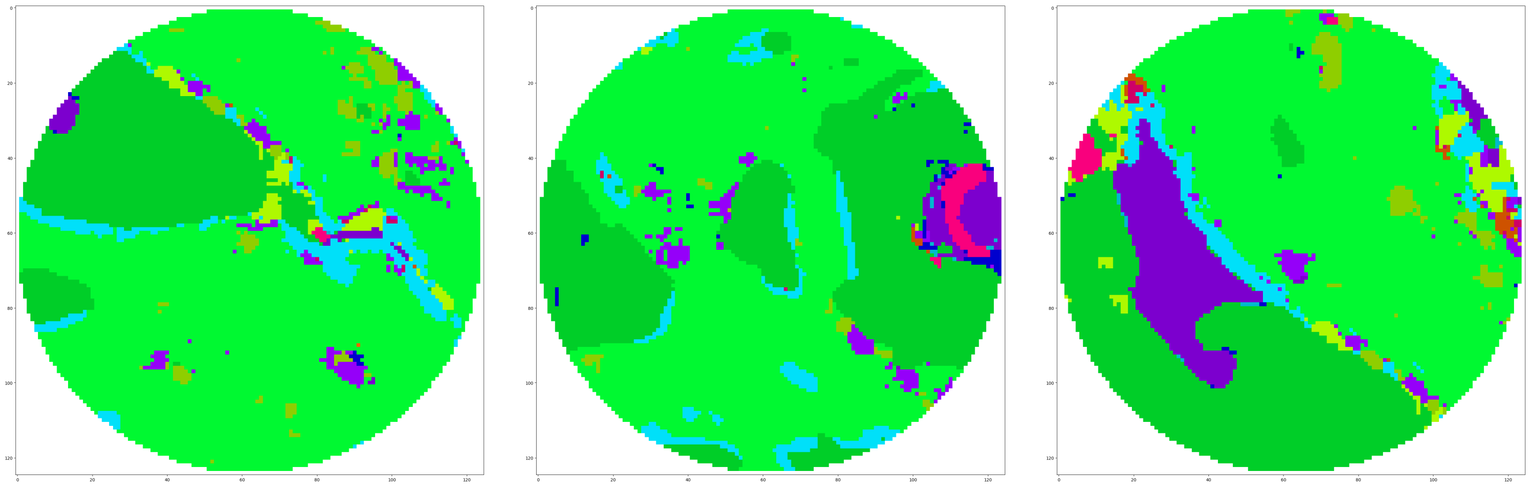

Case 43

Semi-automated output

Left: H&E stained section in the spot chosen by microscopic assessment. Middle: the first step was making an segmentation output. Right the class labels can be displayed

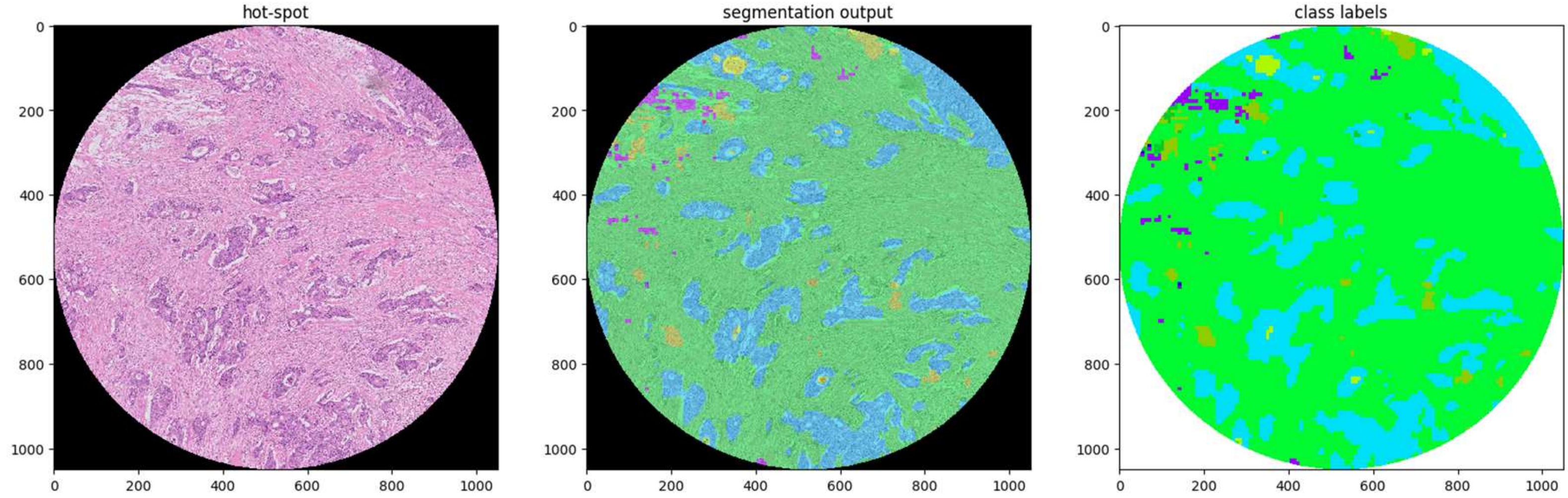

Fully-automated output

Top row; left: the tumor bulk is annotated. Right: heatmap is created. The biggest dot corresponds with the highest stroma-percentage (TSR-1), the second biggest with the second highest (TSR-2), etcetera.

Bottom row; left: the class output of the highest spot (TSR-1), middle: the second highest spot (TSR-2) and right the third highest spot (TSR-3)

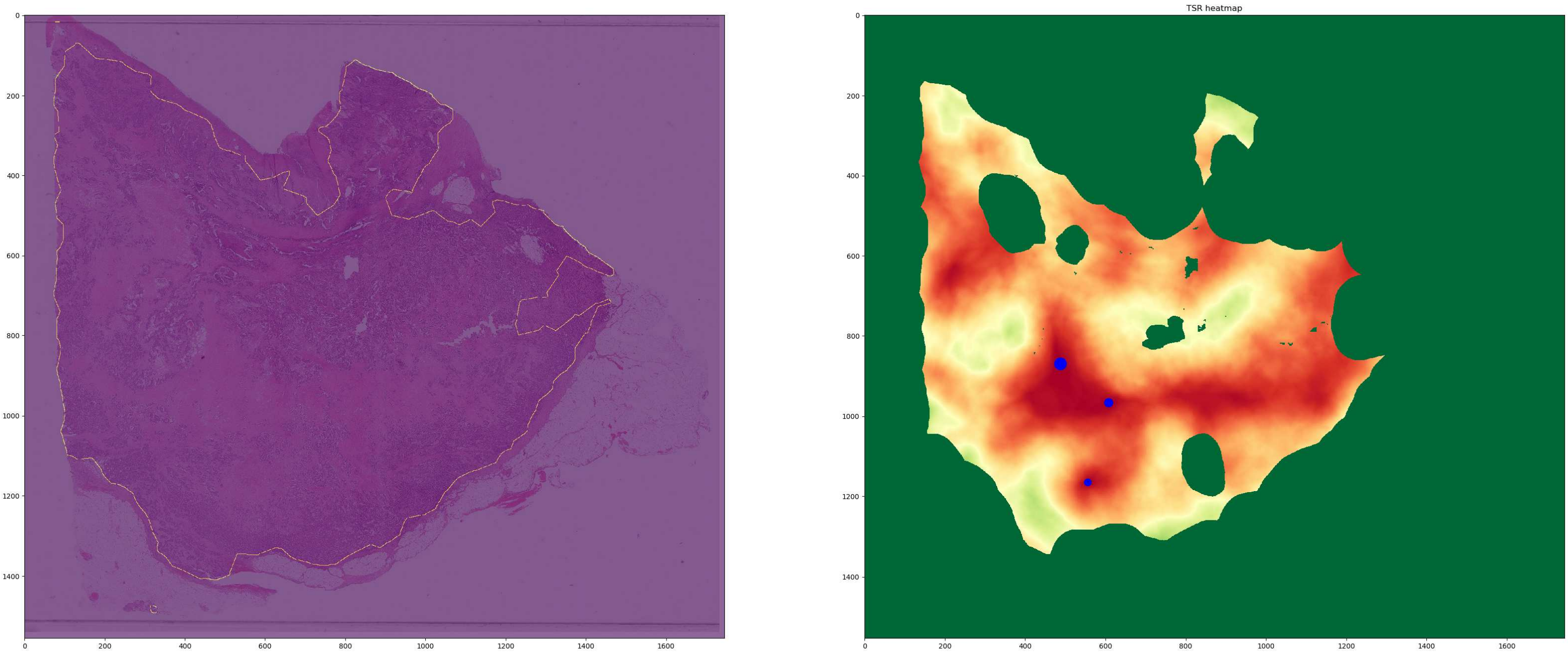

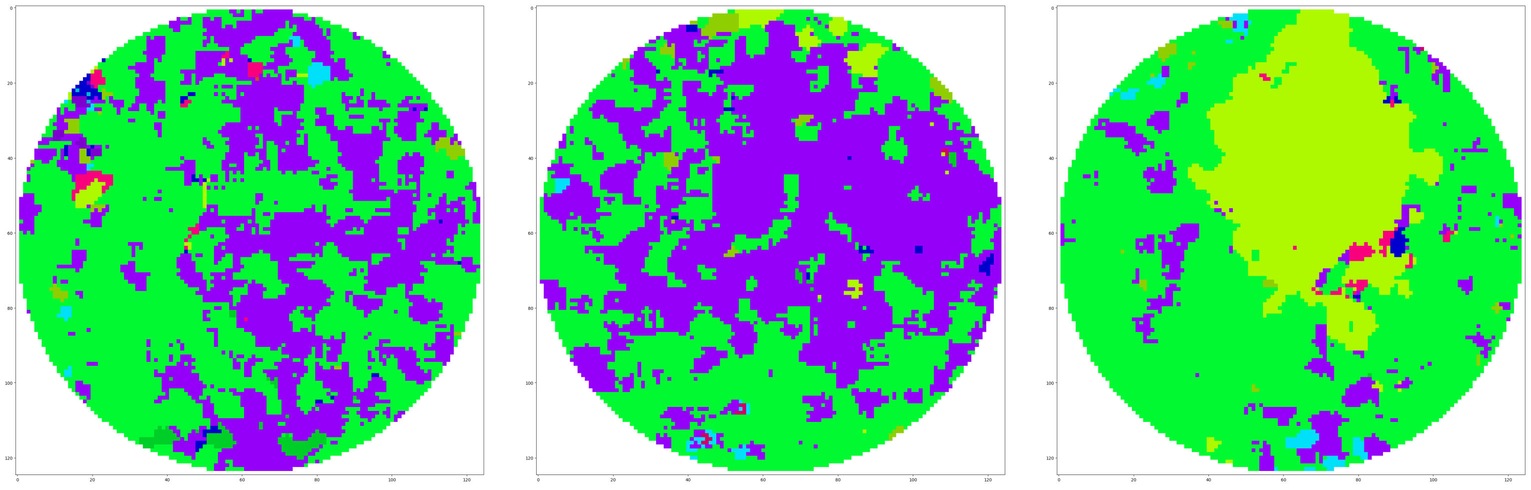

Case 44

Semi-automated output

Left: H&E stained section in the spot chosen by microscopic assessment. Middle: the first step was making an segmentation output. Right the class labels can be displayed

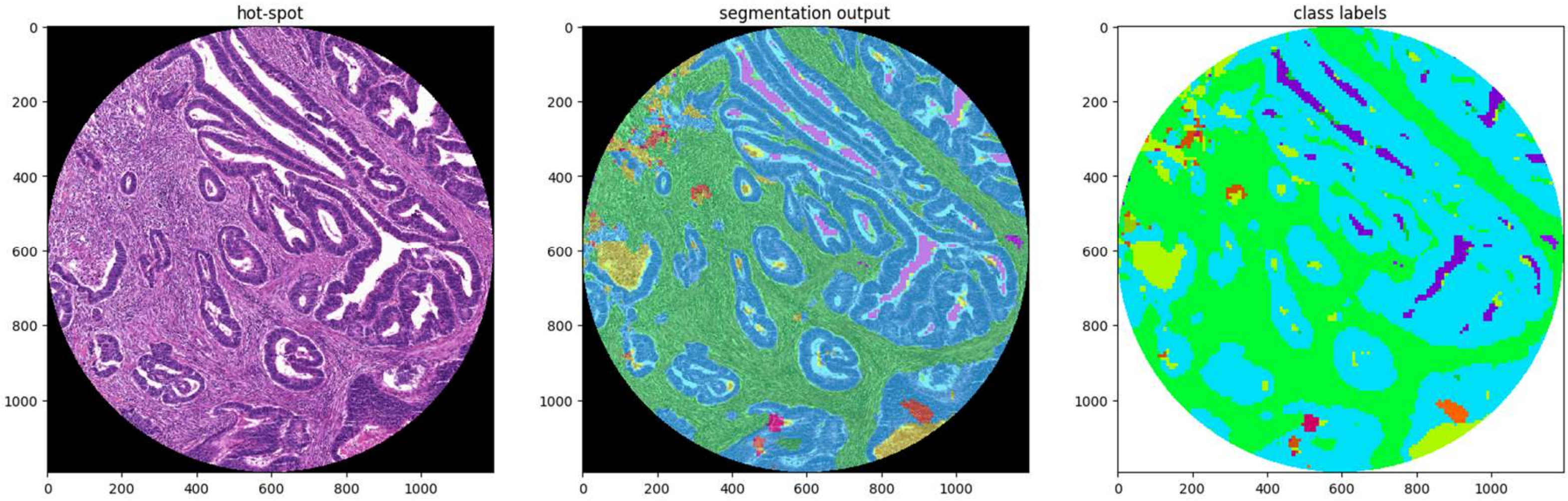

Fully-automated output

Top row; left: the tumor bulk is annotated. Right: heatmap is created. The biggest dot corresponds with the highest stroma-percentage (TSR-1), the second biggest with the second highest (TSR-2), etcetera.

Bottom row; left: the class output of the highest spot (TSR-1), middle: the second highest spot (TSR-2) and right the third highest spot (TSR-3)

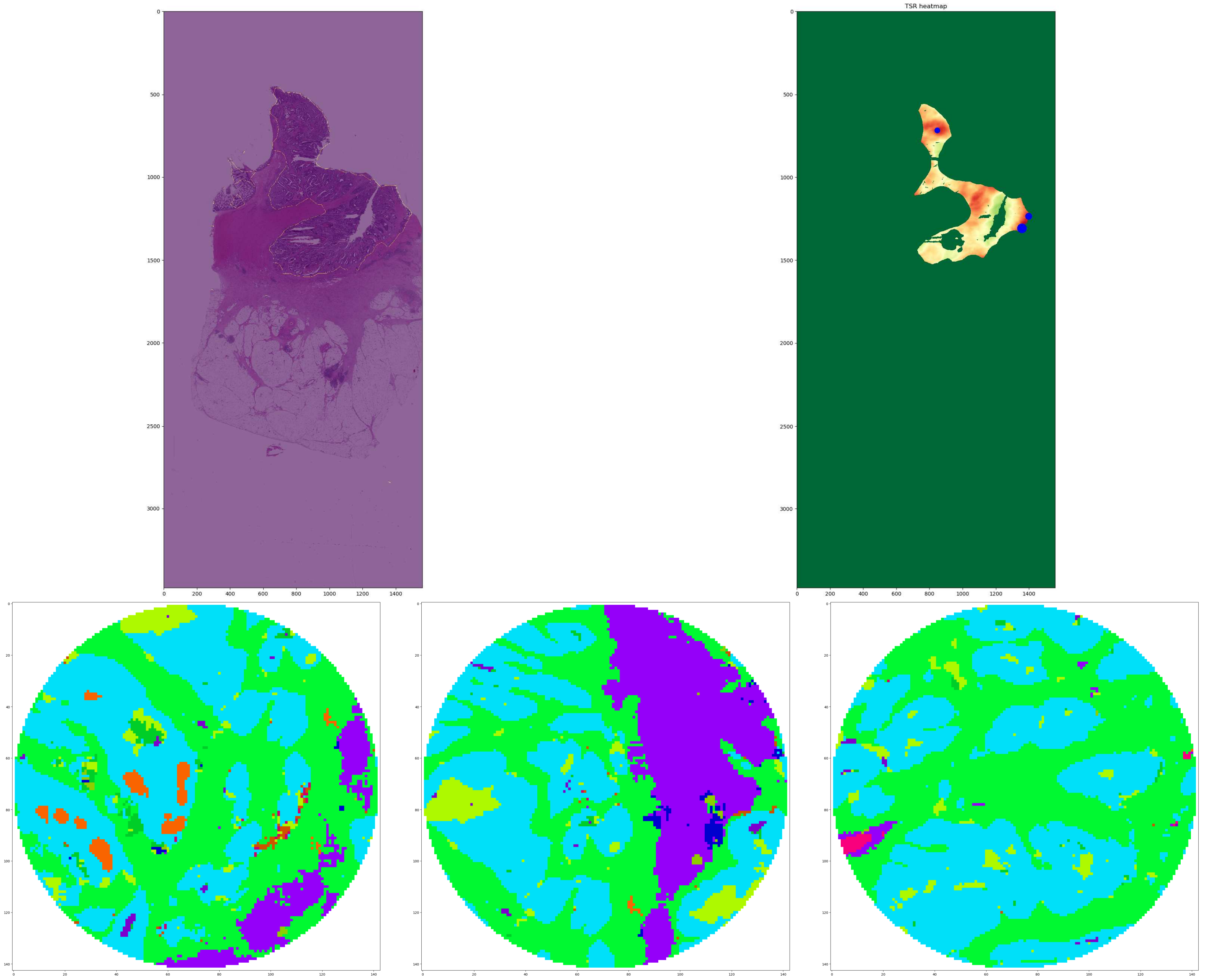

<u>Case 45</u>

Semi-automated output

Left: H&E stained section in the spot chosen by microscopic assessment. Middle: the first step was making an segmentation output. Right the class labels can be displayed

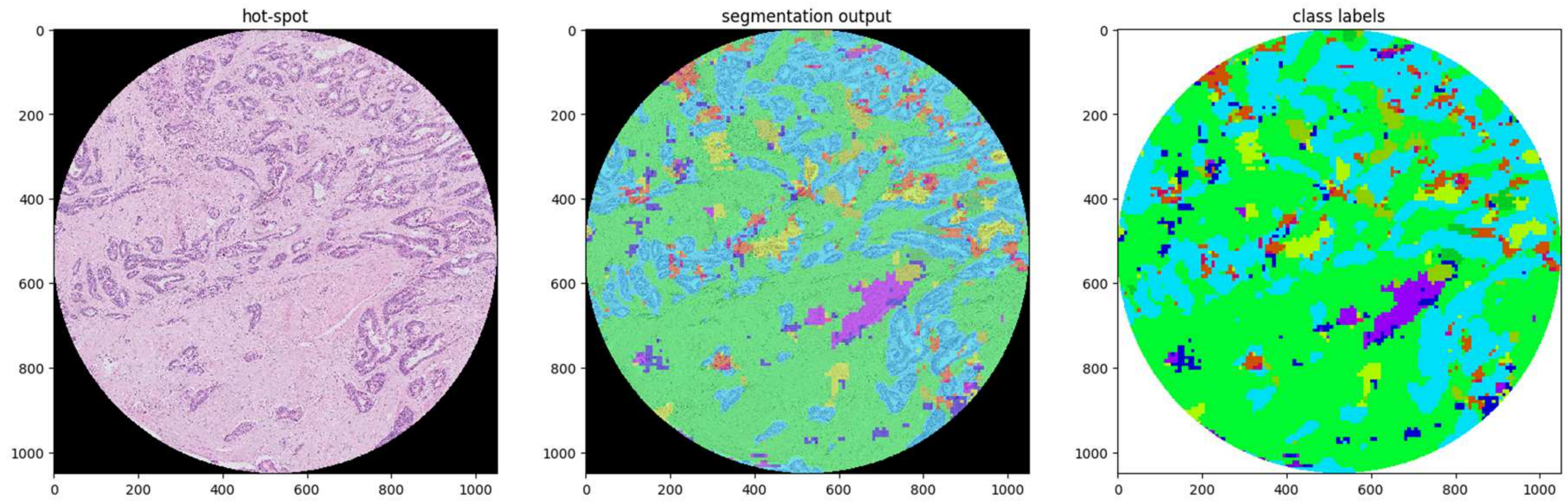

Fully-automated output

Top row; left: the tumor bulk is annotated. Right: heatmap is created. The biggest dot corresponds with the highest stroma-percentage (TSR-1), the second biggest with the second highest (TSR-2), etcetera.

Bottom row; left: the class output of the highest spot (TSR-1), middle: the second highest spot (TSR-2) and right the third highest spot (TSR-3)

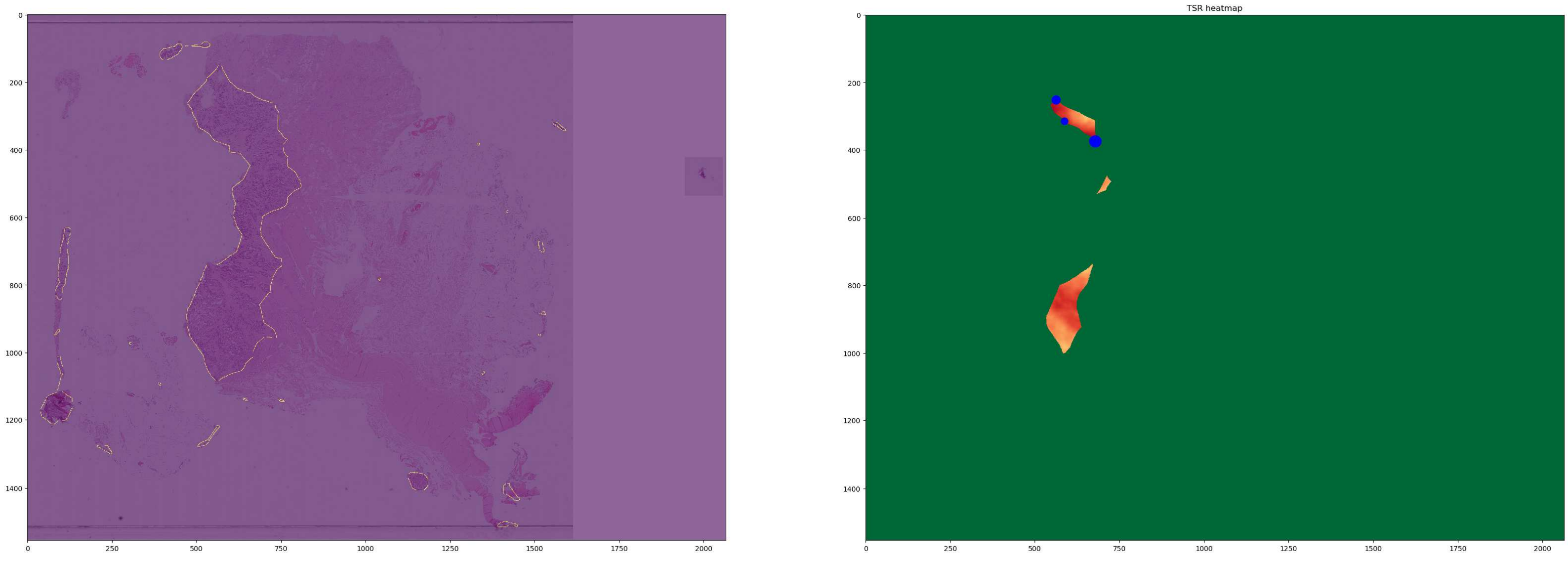

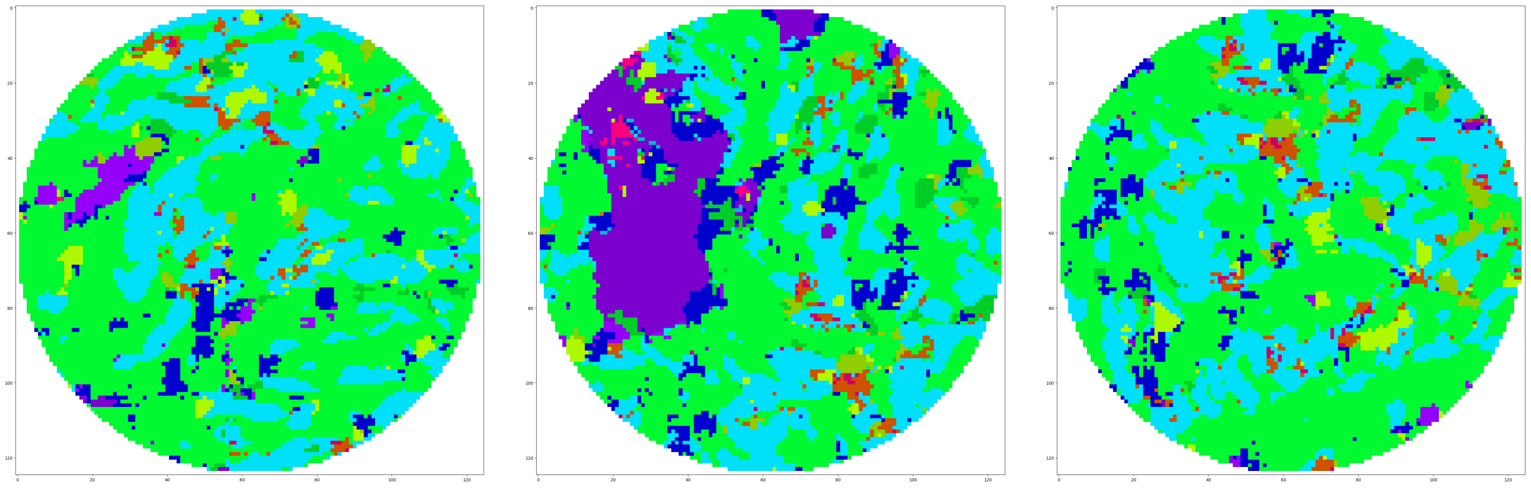

<u>Case 46</u>

Semi-automated output

Left: H&E stained section in the spot chosen by microscopic assessment. Middle: the first step was making an segmentation output. Right the class labels can be displayed

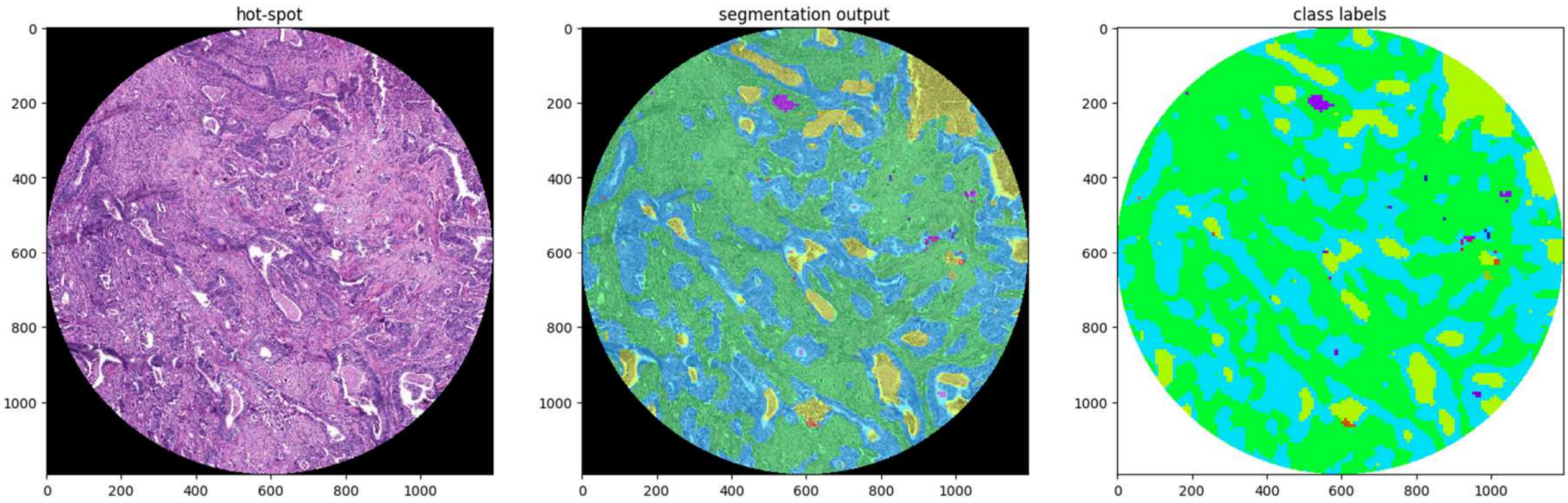

Fully-automated output

Top row; left: the tumor bulk is annotated. Right: heatmap is created. The biggest dot corresponds with the highest stroma-percentage (TSR-1), the second biggest with the second highest (TSR-2), etcetera.

Bottom row; left: the class output of the highest spot (TSR-1), middle: the second highest spot (TSR-2) and right the third highest spot (TSR-3)

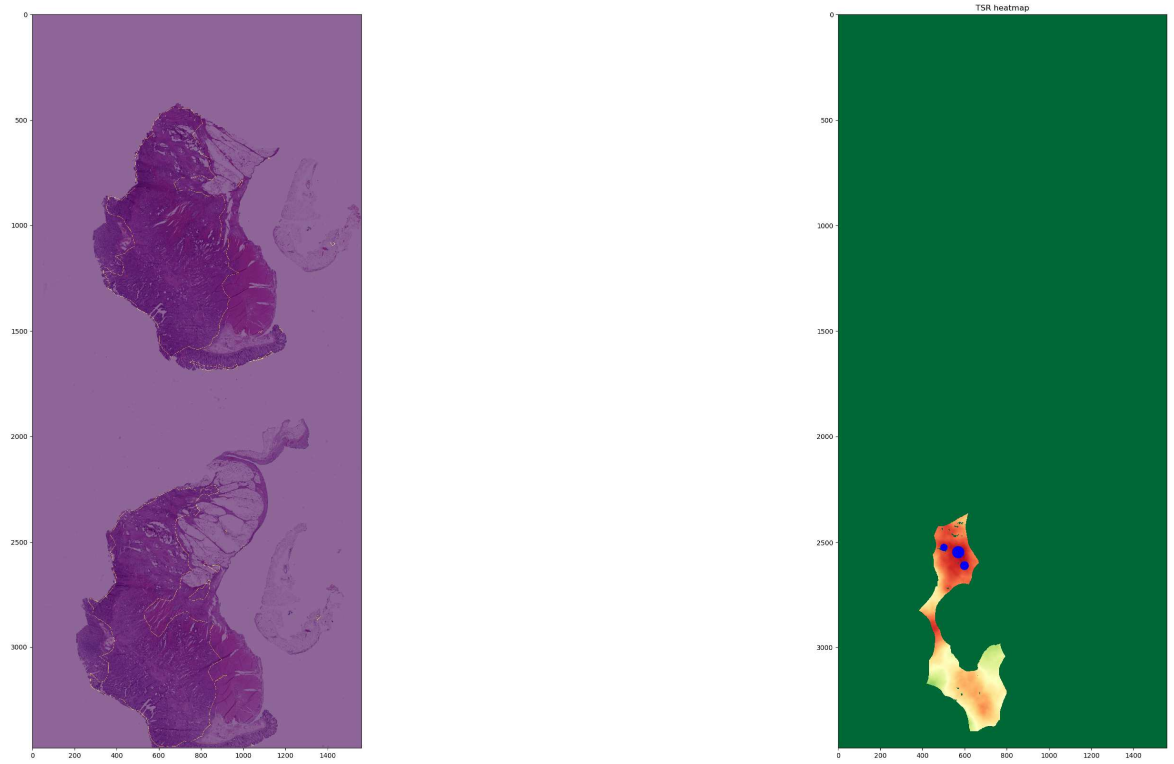

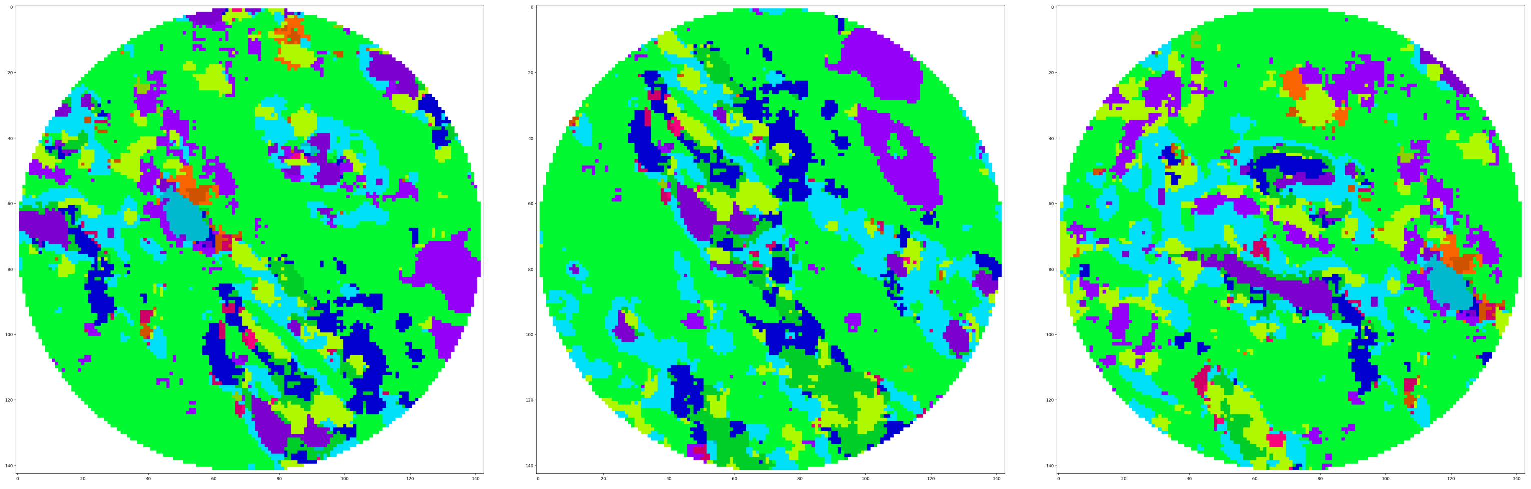

<u>Case 47</u>

Semi-automated output

Left: H&E stained section in the spot chosen by microscopic assessment. Middle: the first step was making an segmentation output. Right the class labels can be displayed

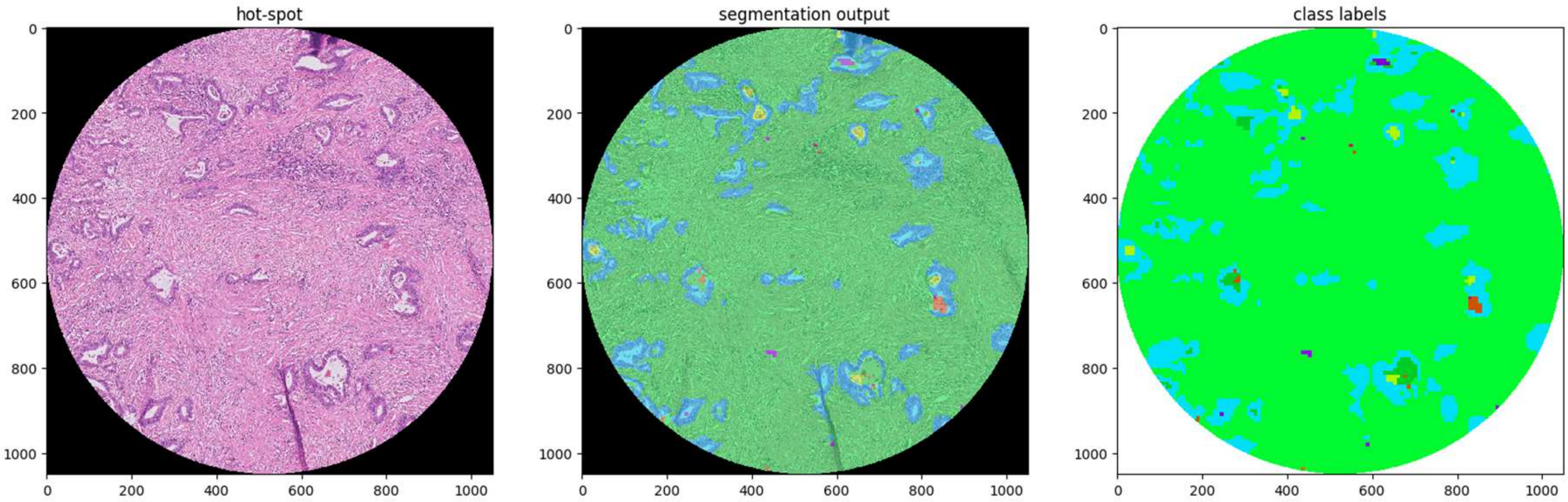

Fully-automated output

Top row; left: the tumor bulk is annotated. Right: heatmap is created. The biggest dot corresponds with the highest stroma-percentage (TSR-1), the second biggest with the second highest (TSR-2), etcetera.

Bottom row; left: the class output of the highest spot (TSR-1), middle: the second highest spot (TSR-2) and right the third highest spot (TSR-3)

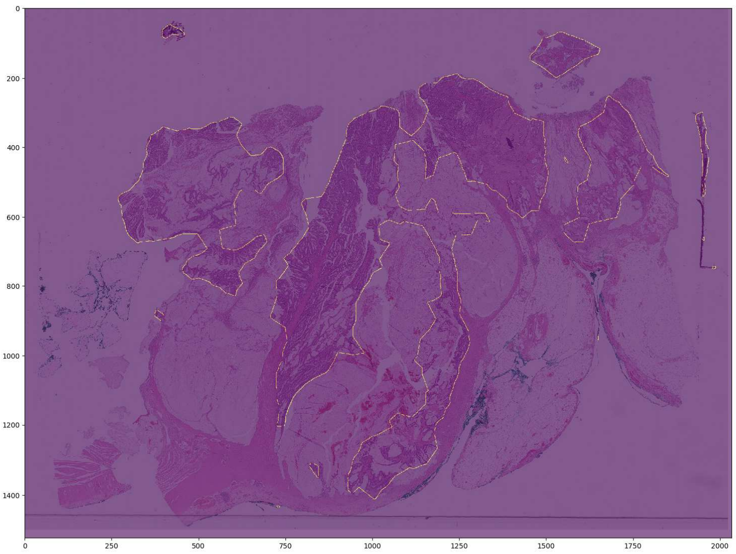

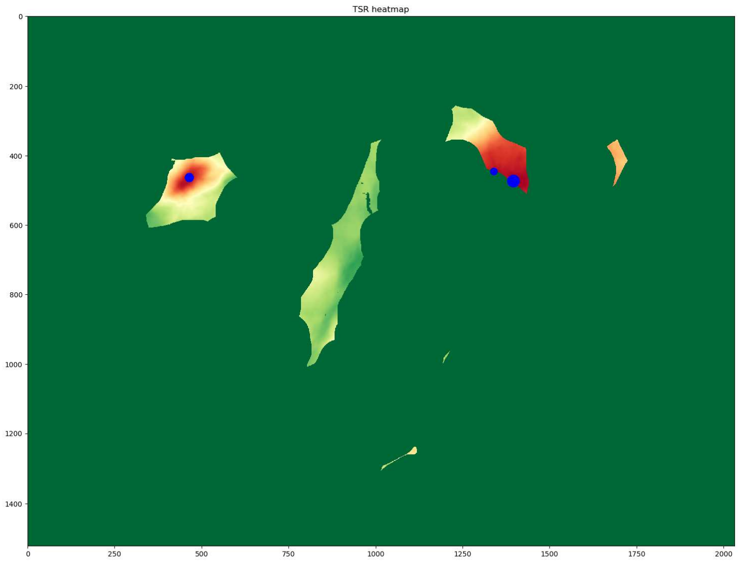

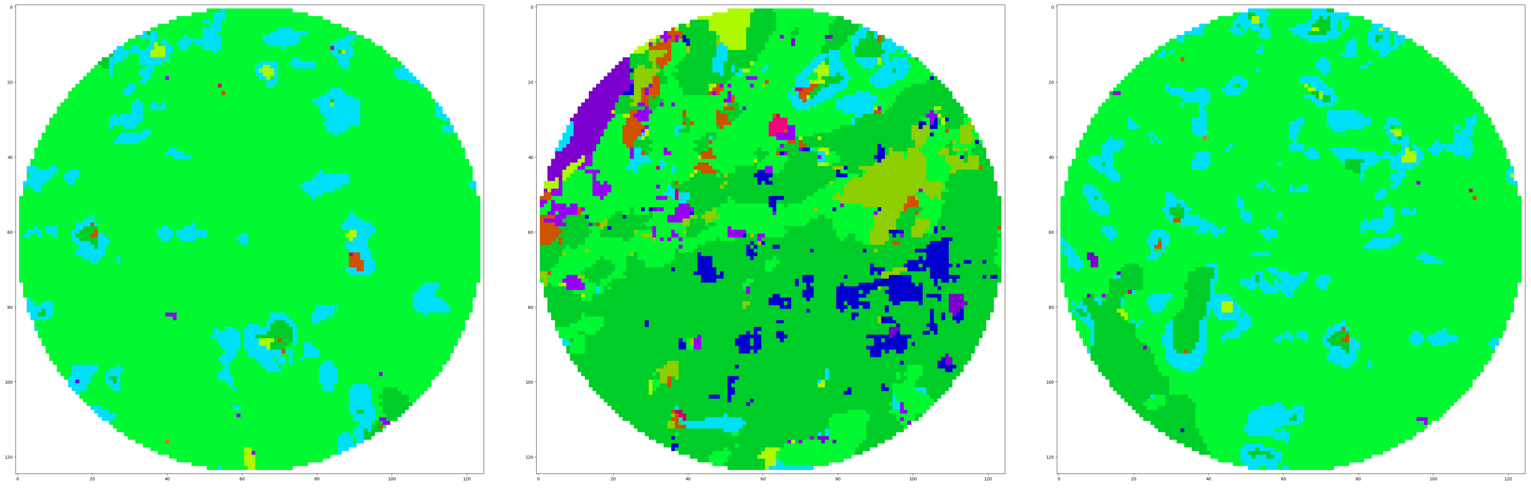

Case 48

Semi-automated output

Left: H&E stained section in the spot chosen by microscopic assessment. Middle: the first step was making an segmentation output. Right the class labels can be displayed

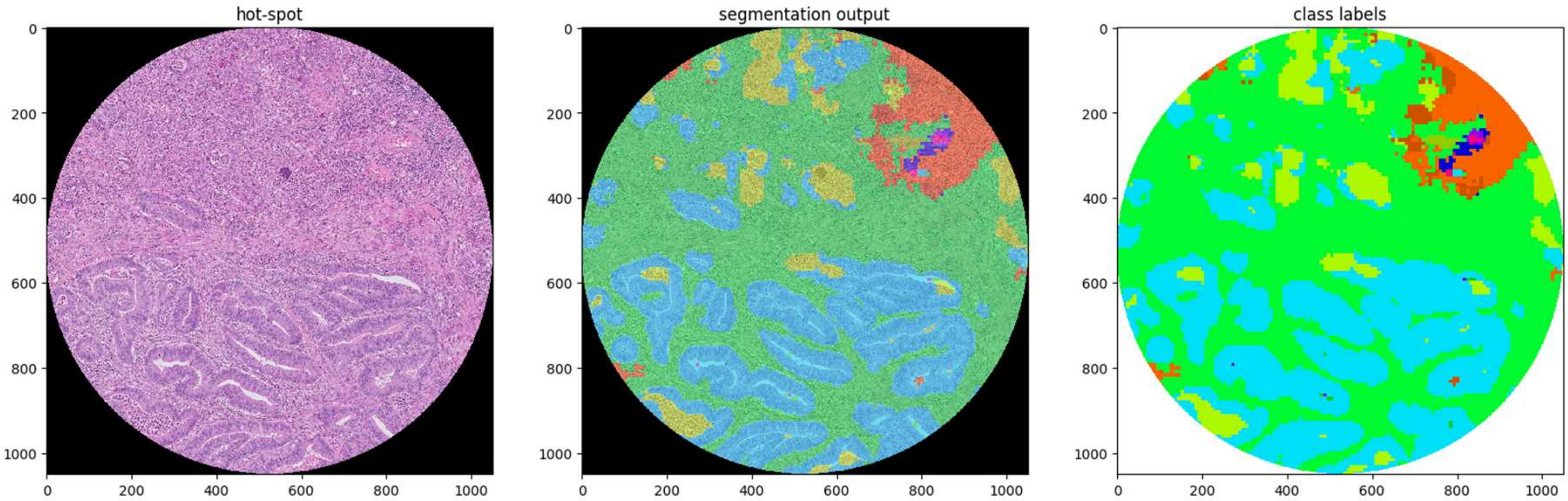

Fully-automated output

Top row; left: the tumor bulk is annotated. Right: heatmap is created. The biggest dot corresponds with the highest stroma-percentage (TSR-1), the second biggest with the second highest (TSR-2), etcetera.

Bottom row; left: the class output of the highest spot (TSR-1), middle: the second highest spot (TSR-2) and right the third highest spot (TSR-3)

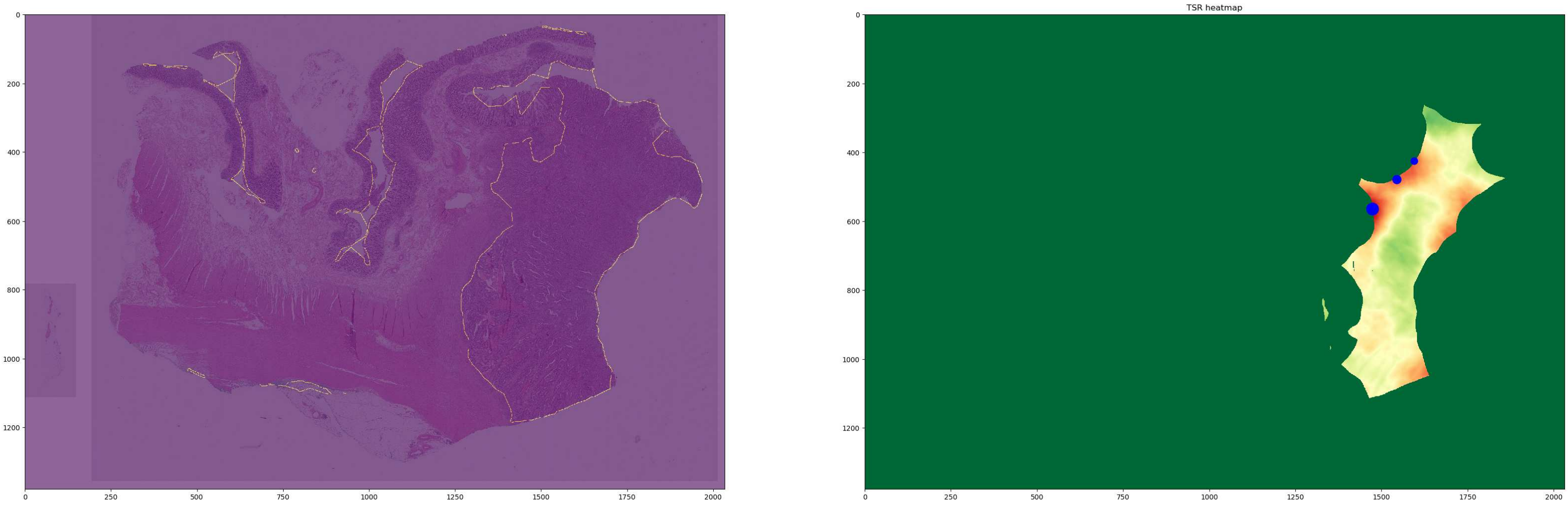

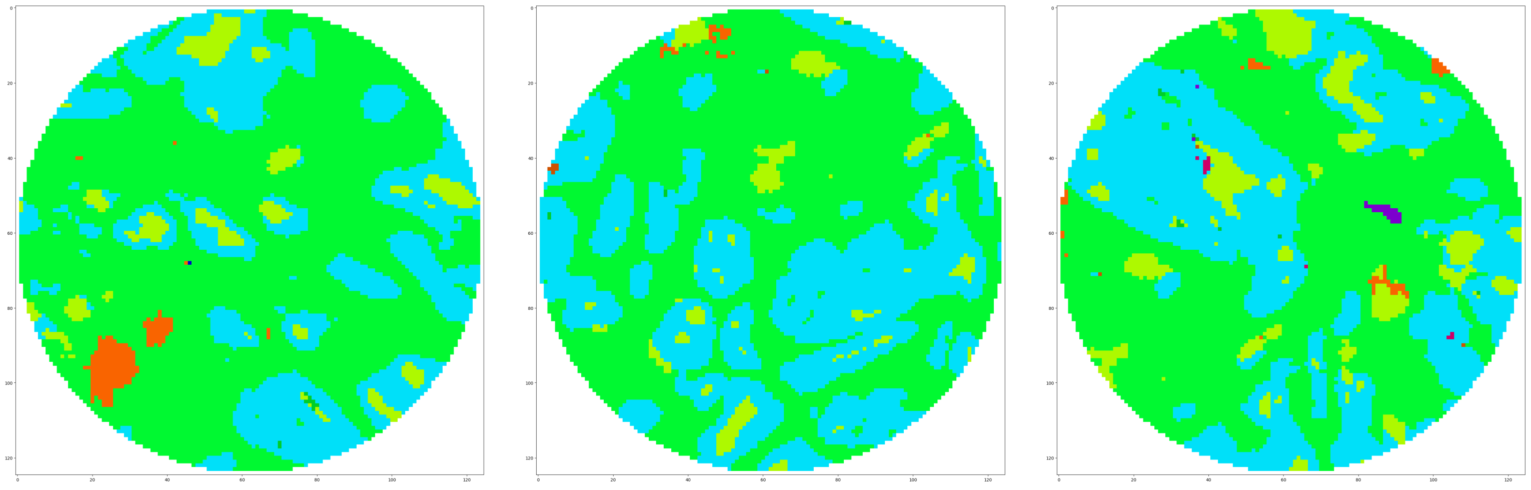

<u>Case 49</u>

Semi-automated output

Left: H&E stained section in the spot chosen by microscopic assessment. Middle: the first step was making an segmentation output. Right the class labels can be displayed

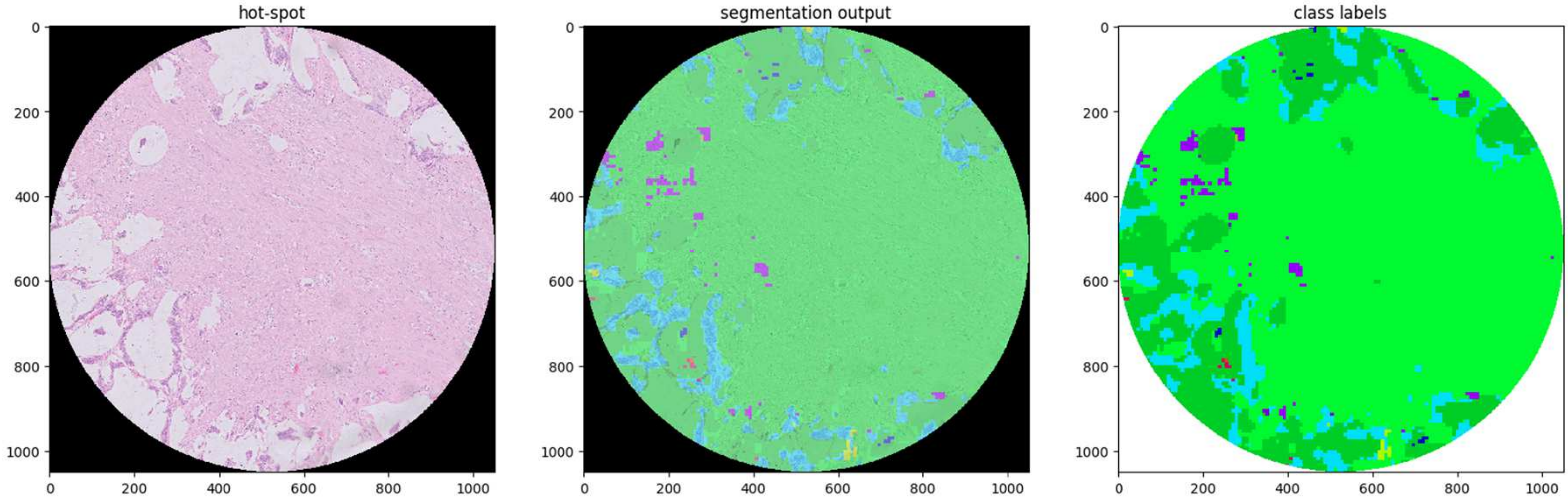

Fully-automated output

Top row; left: the tumor bulk is annotated. Right: heatmap is created. The biggest dot corresponds with the highest stroma-percentage (TSR-1), the second biggest with the second highest (TSR-2), etcetera.

Bottom row; left: the class output of the highest spot (TSR-1), middle: the second highest spot (TSR-2) and right the third highest spot (TSR-3)

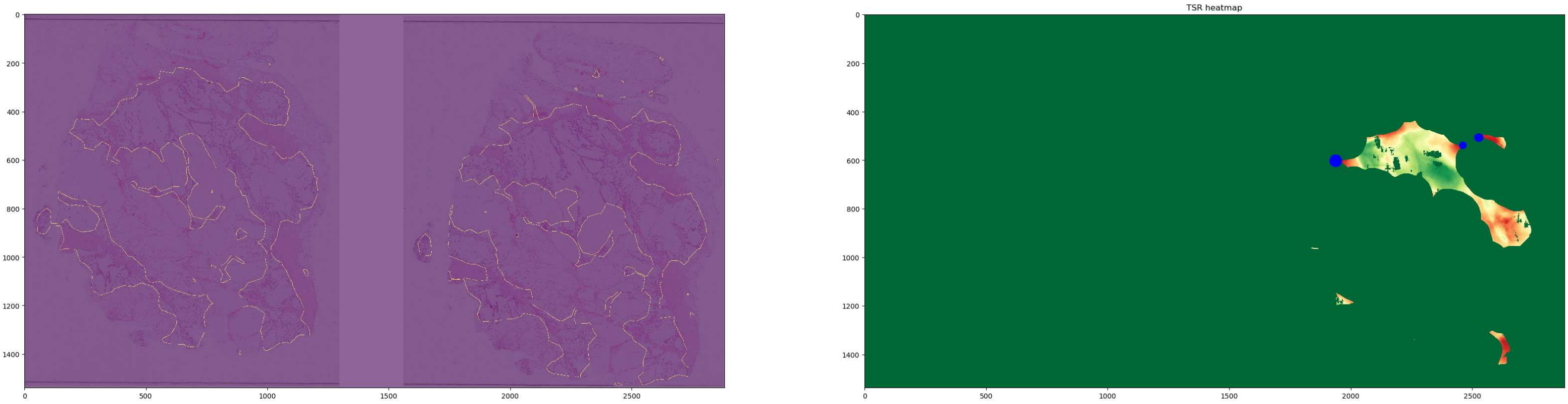

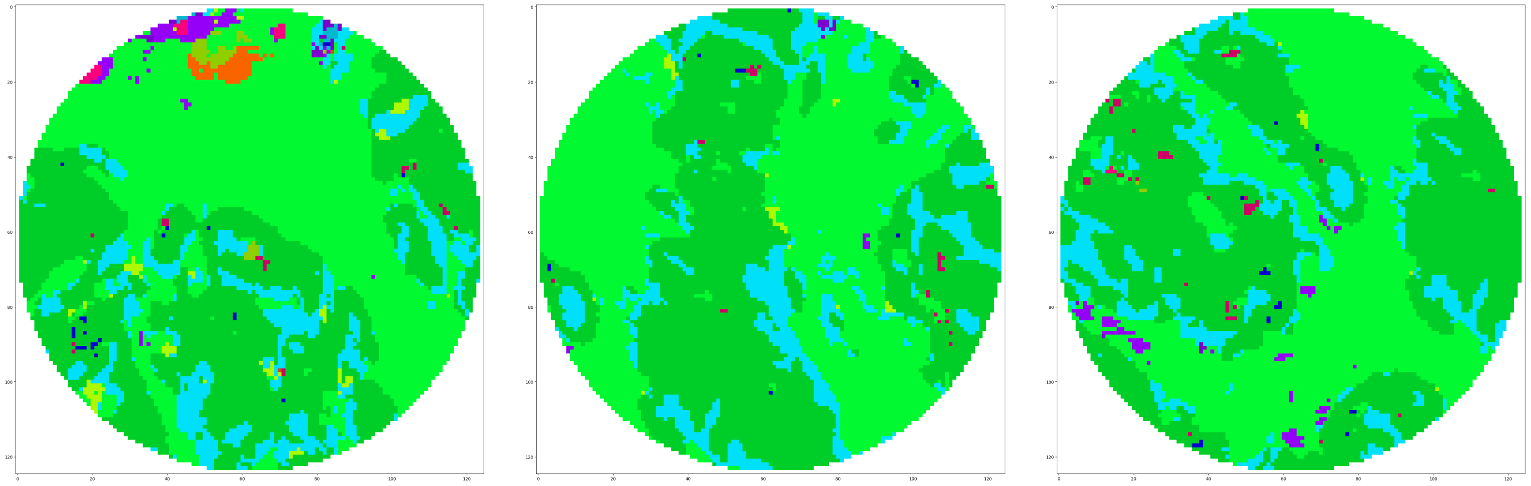

Supplement: Supplementary material 3 — Output for all cases for the semi-automated algorithm and the fully-automated algorithm. In part 1 case 1-24, part 2 case 25-49 and part 3 50-75. [file mmc3.zip › mmc3/Supplementary data - output figures (part 2).pdf]
