# Supplementary material for: Deep learning based tumor–stroma ratio scoring in colon cancer correlates with microscopic assessment
Source: J Pathol Inform. 2023 Jan 20;14:100191. doi: 10.1016/j.jpi.2023.100191 (PMC9922811; doi:10.1016/j.jpi.2023.100191)

Supplementary file

Original data figures

(Part 3)

Legend

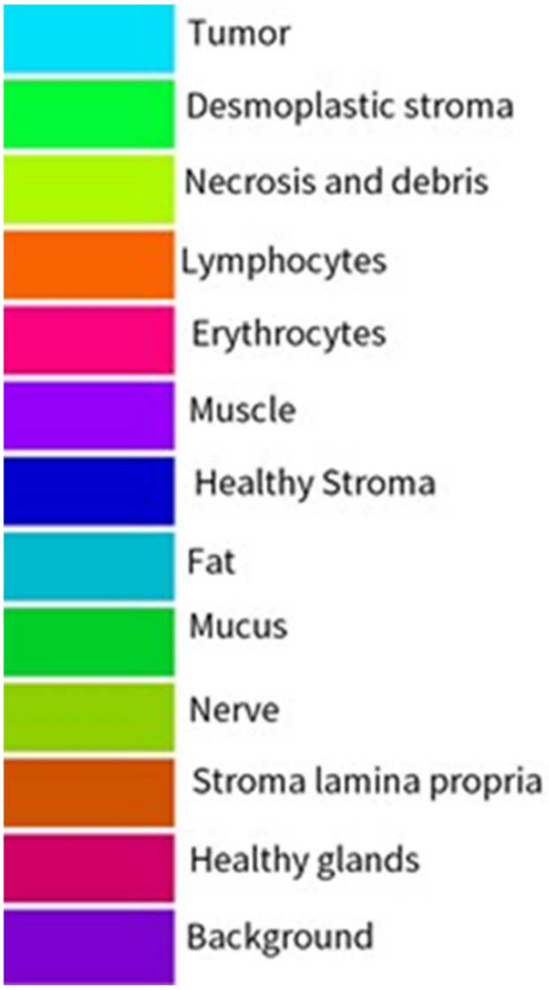
Tumor
Desmoplastic stroma
Necrosis and debris
Lymphocytes
Erythrocytes
Muscle
Healthy Stroma
Fat
Mucus
Nerve
Stroma lamina propria
Healthy glands
Background

Case 50

Semi-automated output

Left: H&E stained section in the spot chosen by microscopic assessment. Middle: the first step was making an segmentation output. Right the class labels can be displayed

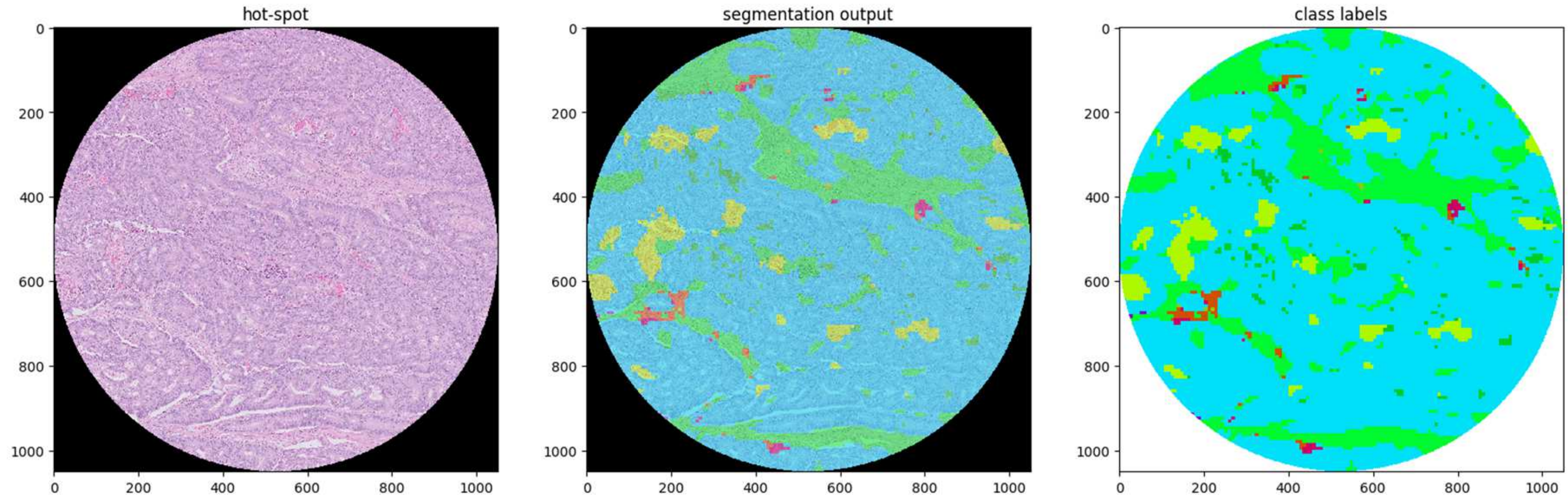

Fully-automated output

Top row; left: the tumor bulk is annotated. Right: heatmap is created. The biggest dot corresponds with the highest stroma-percentage (TSR-1), the second biggest with the second highest (TSR-2), etcetera.

Bottom row; left: the class output of the highest spot (TSR-1), middle: the second highest spot (TSR-2) and right the third highest spot (TSR-3)

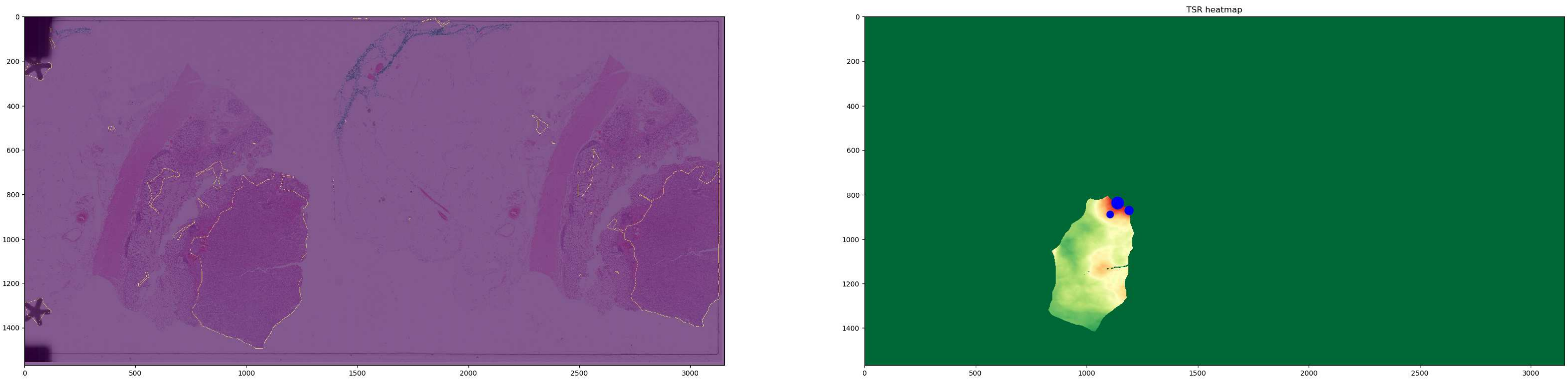

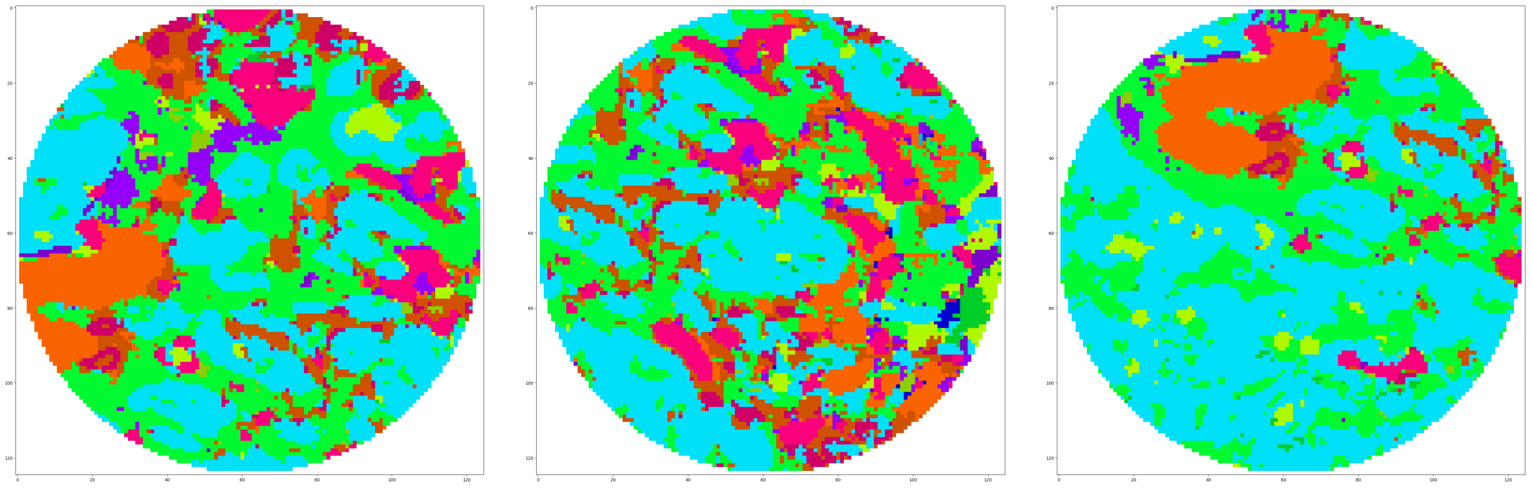

Case 51

Semi-automated output

Left: H&E stained section in the spot chosen by microscopic assessment. Middle: the first step was making an segmentation output. Right the class labels can be displayed

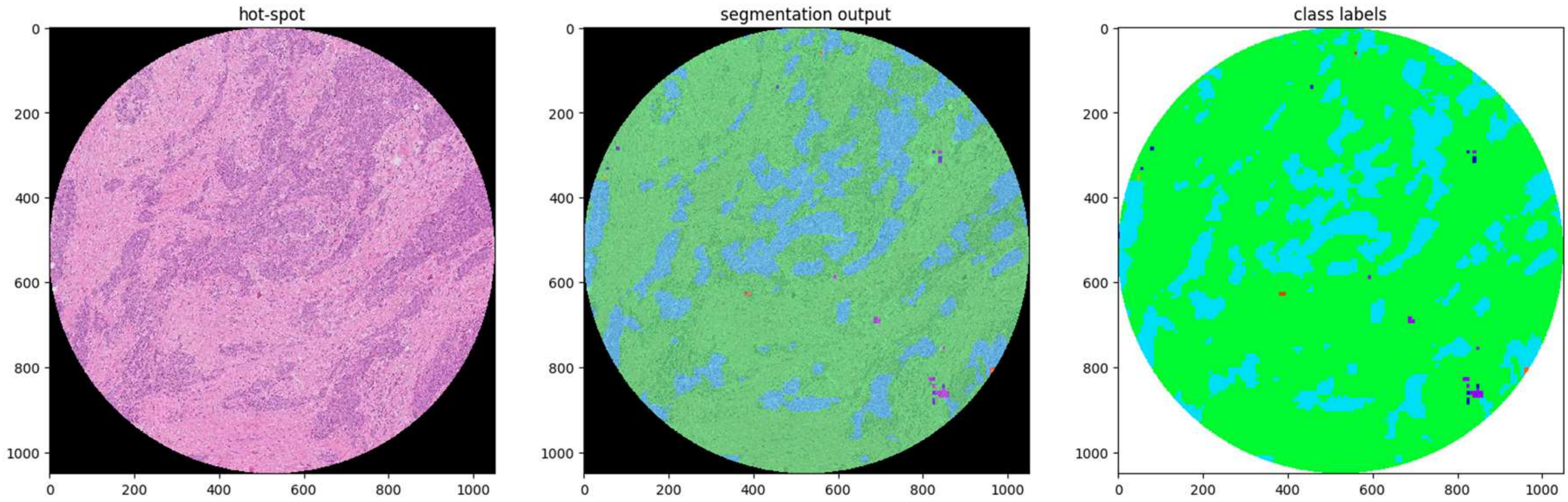

Fully-automated output

Top row; left: the tumor bulk is annotated. Right: heatmap is created. The biggest dot corresponds with the highest stroma-percentage (TSR-1), the second biggest with the second highest (TSR-2), etcetera.

Bottom row; left: the class output of the highest spot (TSR-1), middle: the second highest spot (TSR-2) and right the third highest spot (TSR-3)

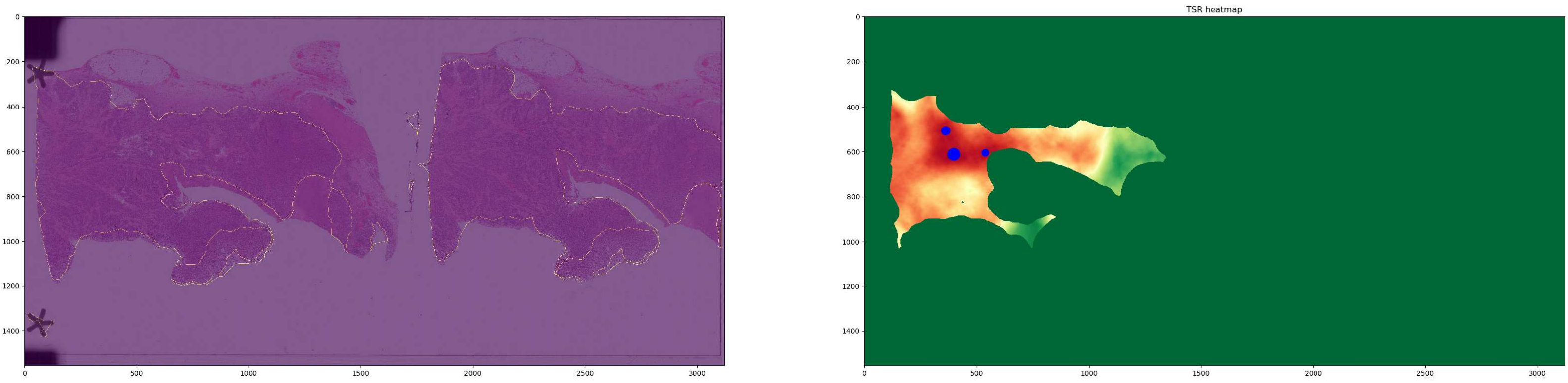

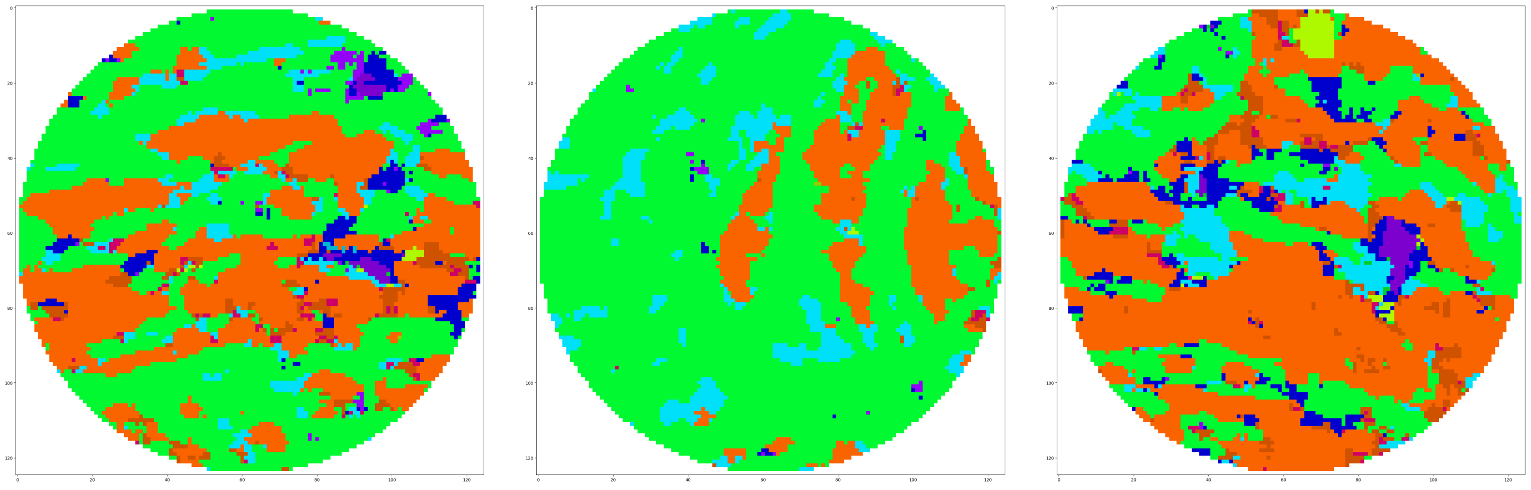

Case 52

Semi-automated output

Left: H&E stained section in the spot chosen by microscopic assessment. Middle: the first step was making an segmentation output. Right the class labels can be displayed

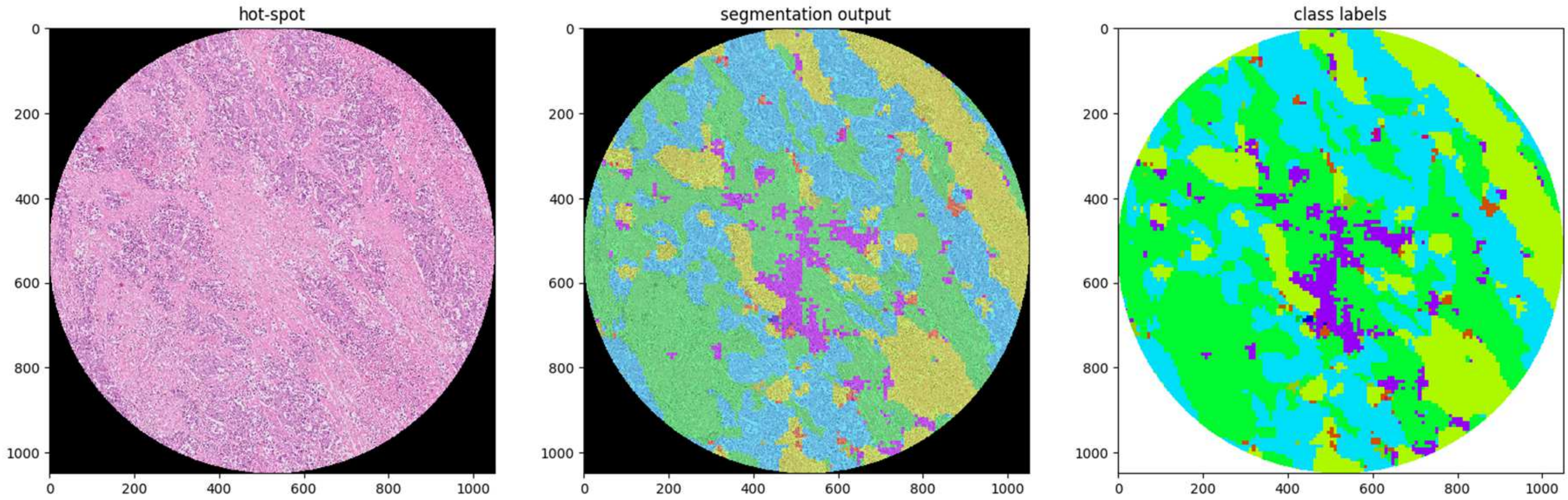

Fully-automated output

Top row; left: the tumor bulk is annotated. Right: heatmap is created. The biggest dot corresponds with the highest stroma-percentage (TSR-1), the second biggest with the second highest (TSR-2), etcetera.

Bottom row; left: the class output of the highest spot (TSR-1), middle: the second highest spot (TSR-2) and right the third highest spot (TSR-3)

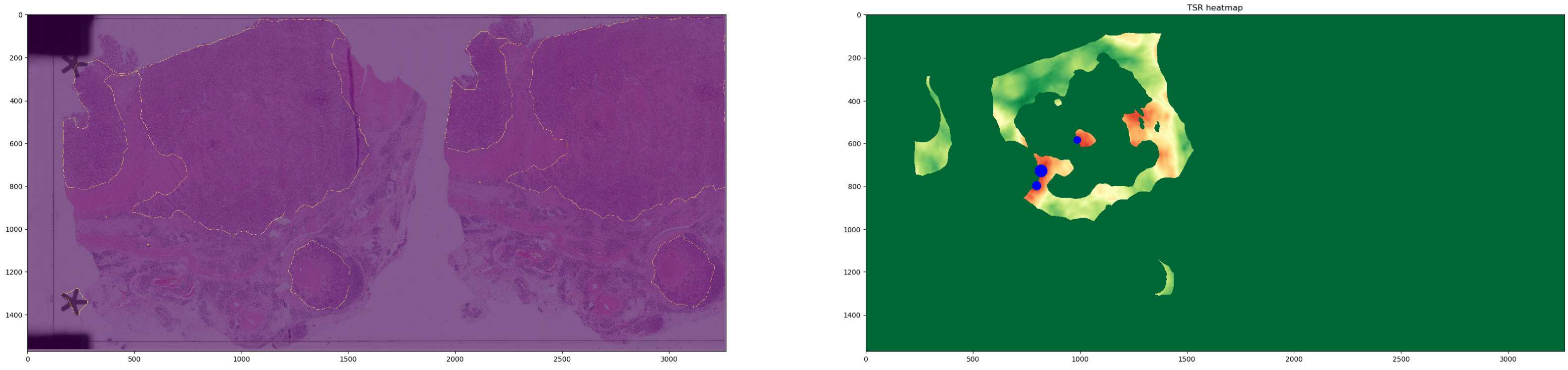

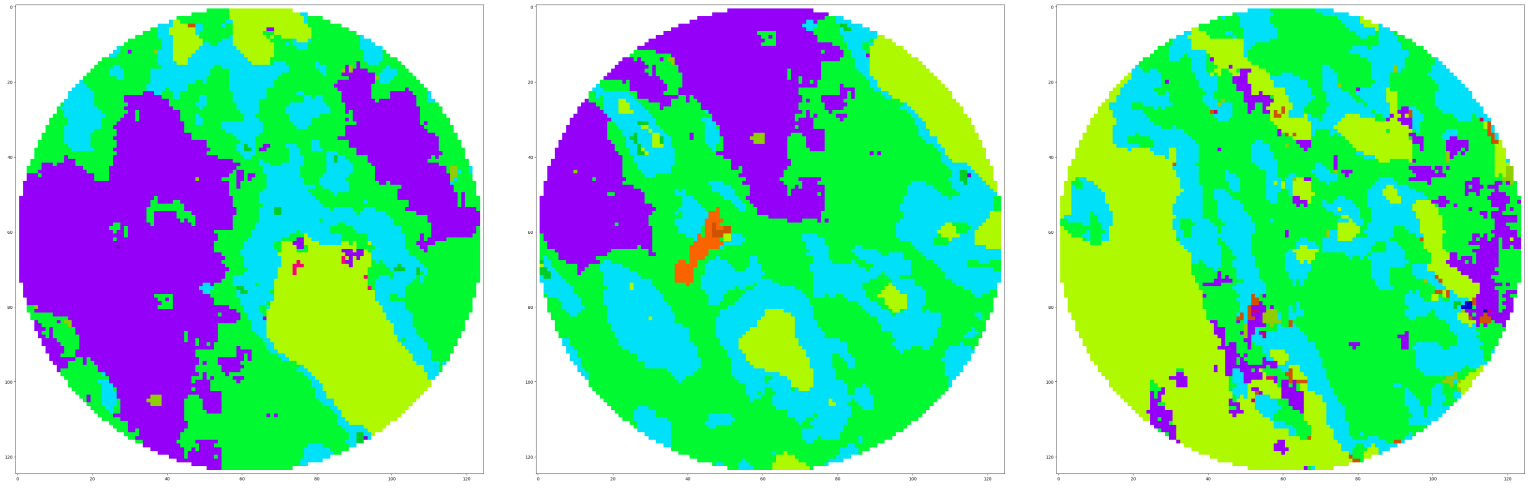

Case 53

Semi-automated output

Left: H&E stained section in the spot chosen by microscopic assessment. Middle: the first step was making an segmentation output. Right the class labels can be displayed

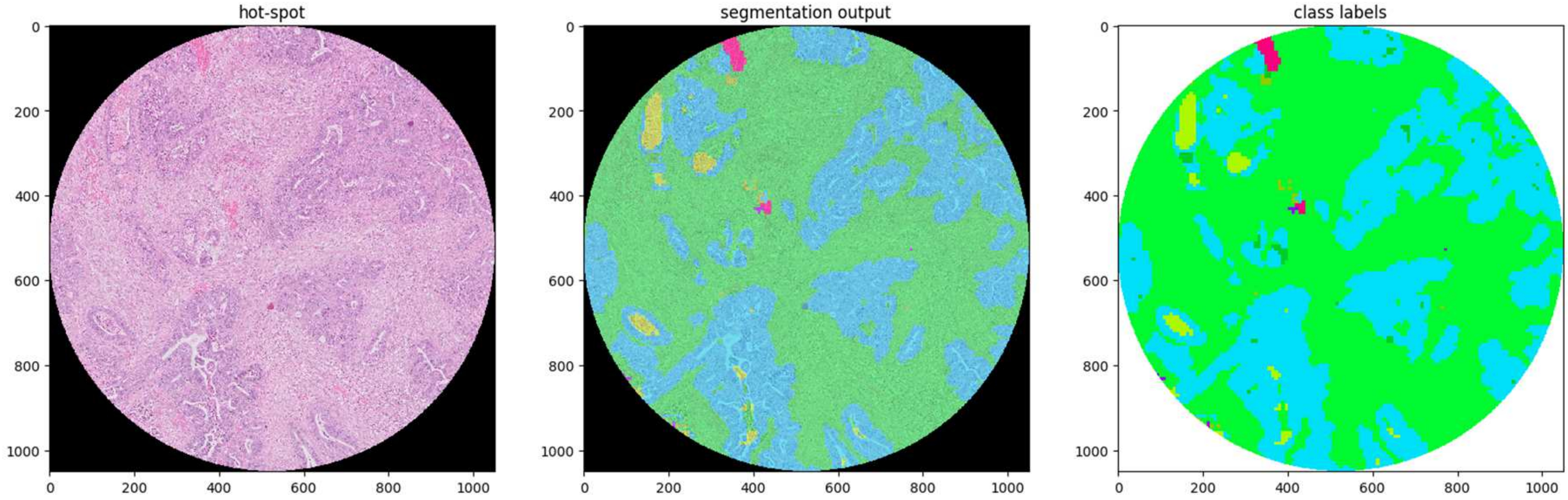

Fully-automated output

Top row; left: the tumor bulk is annotated. Right: heatmap is created. The biggest dot corresponds with the highest stroma-percentage (TSR-1), the second biggest with the second highest (TSR-2), etcetera.

Bottom row; left: the class output of the highest spot (TSR-1), middle: the second highest spot (TSR-2) and right the third highest spot (TSR-3)

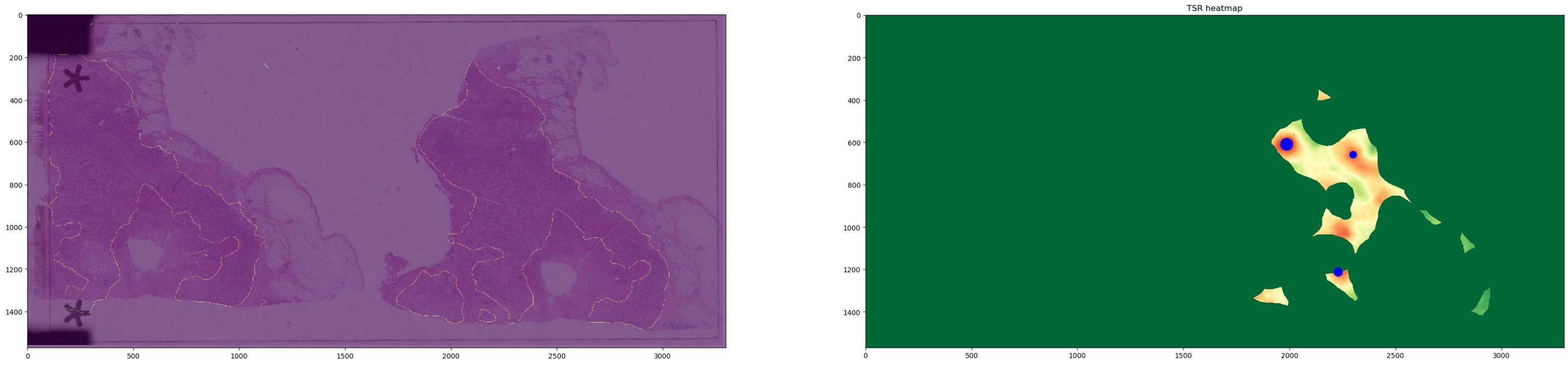

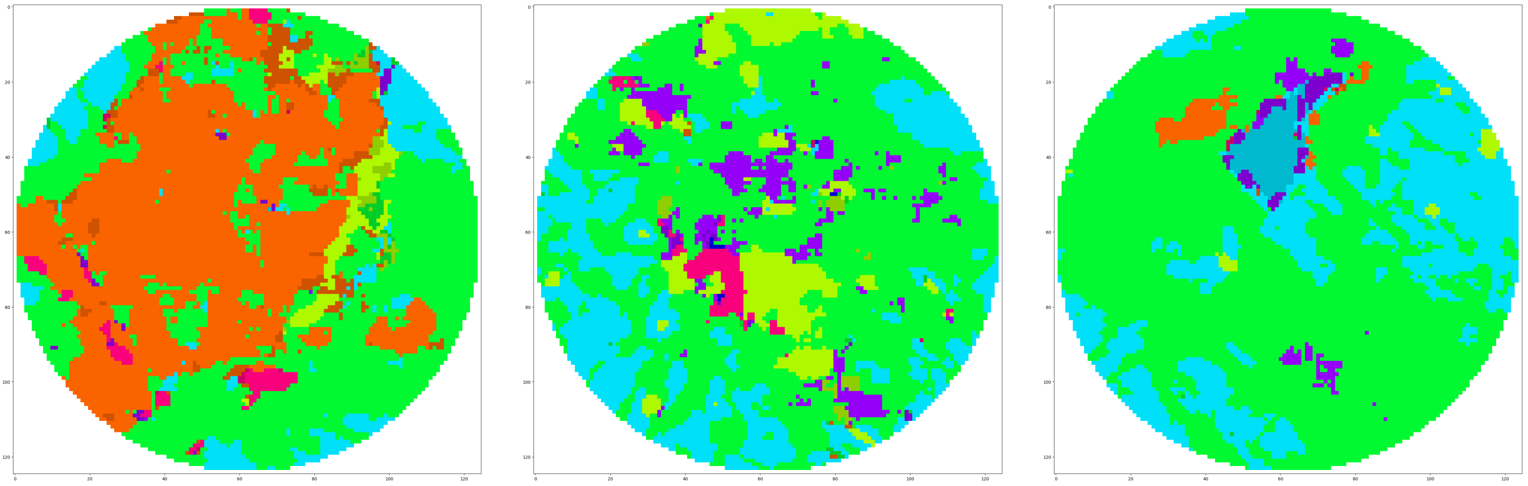

Case 54

Semi-automated output

Left: H&E stained section in the spot chosen by microscopic assessment. Middle: the first step was making an segmentation output. Right the class labels can be displayed

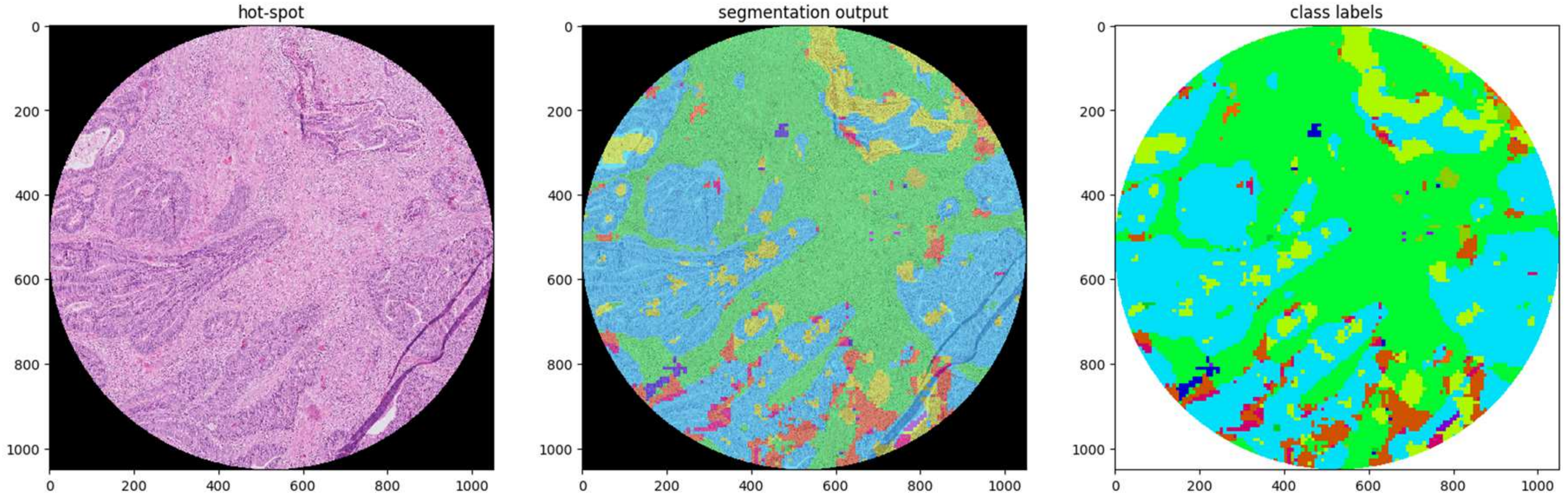

Fully-automated output

Top row; left: the tumor bulk is annotated. Right: heatmap is created. The biggest dot corresponds with the highest stroma-percentage (TSR-1), the second biggest with the second highest (TSR-2), etcetera.

Bottom row; left: the class output of the highest spot (TSR-1), middle: the second highest spot (TSR-2) and right the third highest spot (TSR-3)

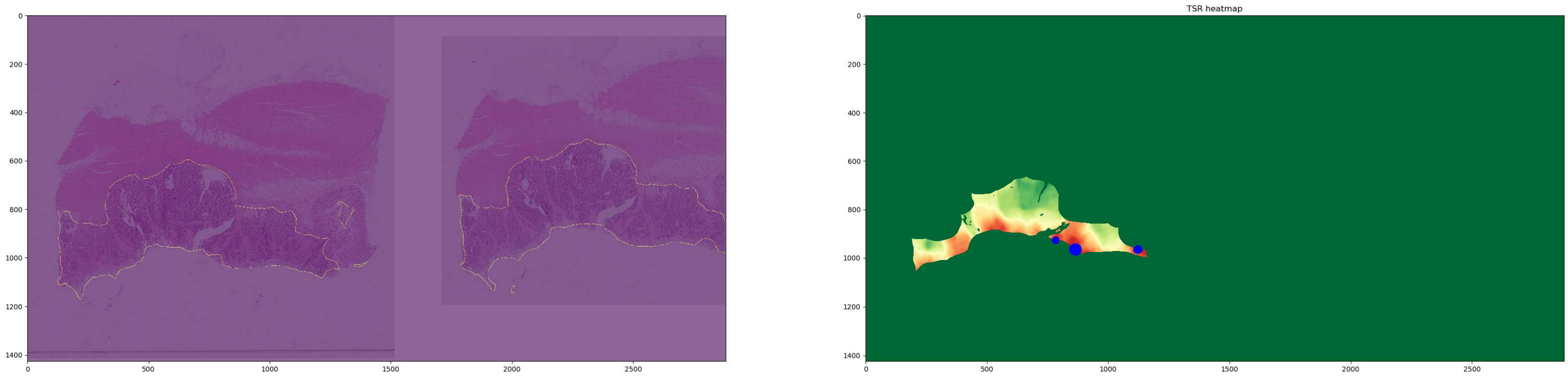

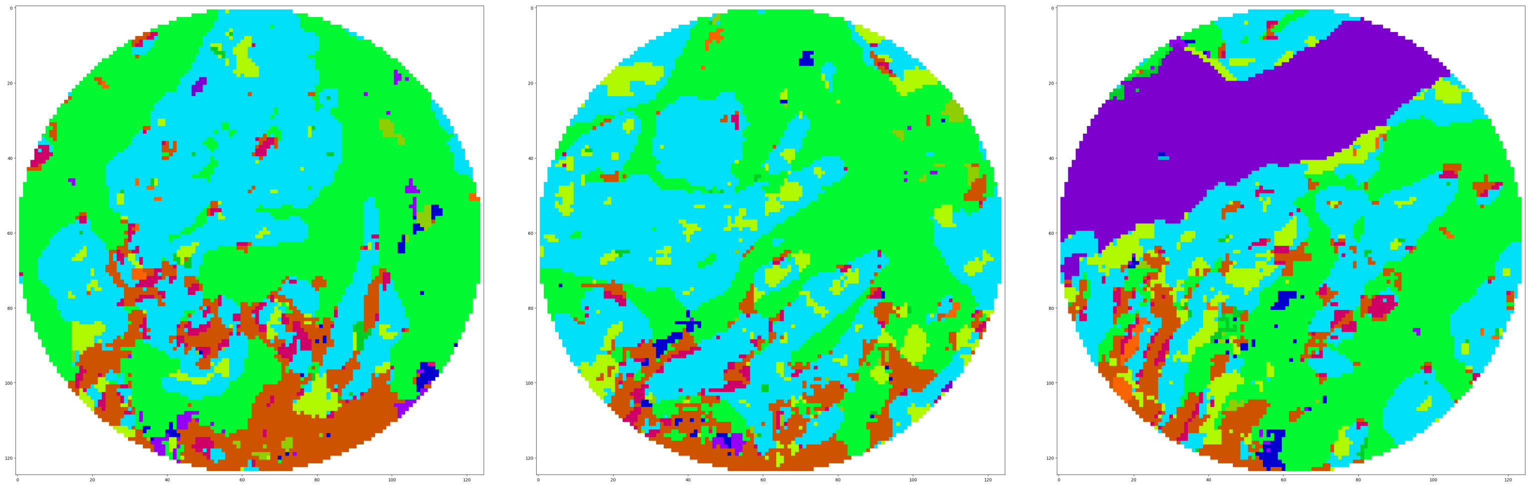

Case 55

Semi-automated output

Left: H&E stained section in the spot chosen by microscopic assessment. Middle: the first step was making an segmentation output. Right the class labels can be displayed

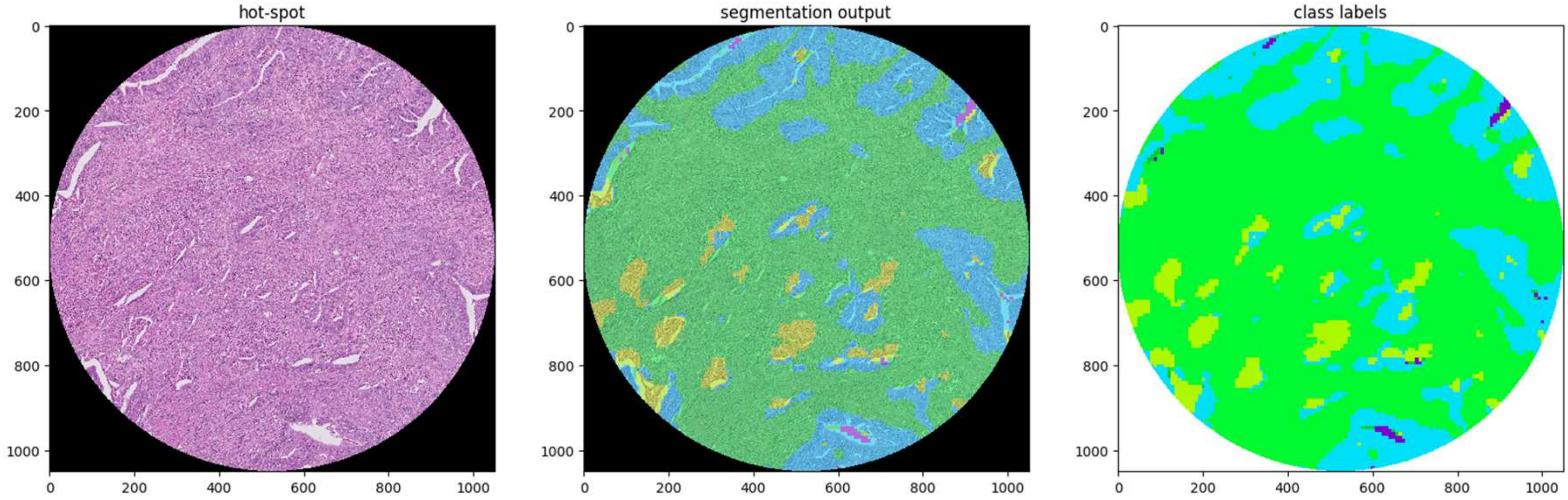

Fully-automated output

Top row; left: the tumor bulk is annotated. Right: heatmap is created. The biggest dot corresponds with the highest stroma-percentage (TSR-1), the second biggest with the second highest (TSR-2), etcetera.

Bottom row; left: the class output of the highest spot (TSR-1), middle: the second highest spot (TSR-2) and right the third highest spot (TSR-3)

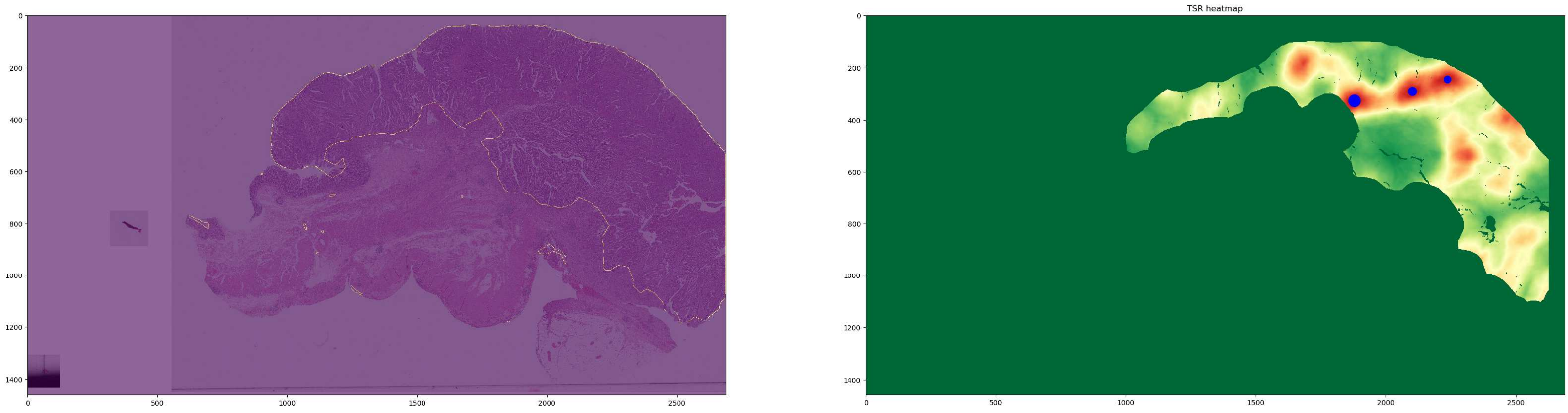

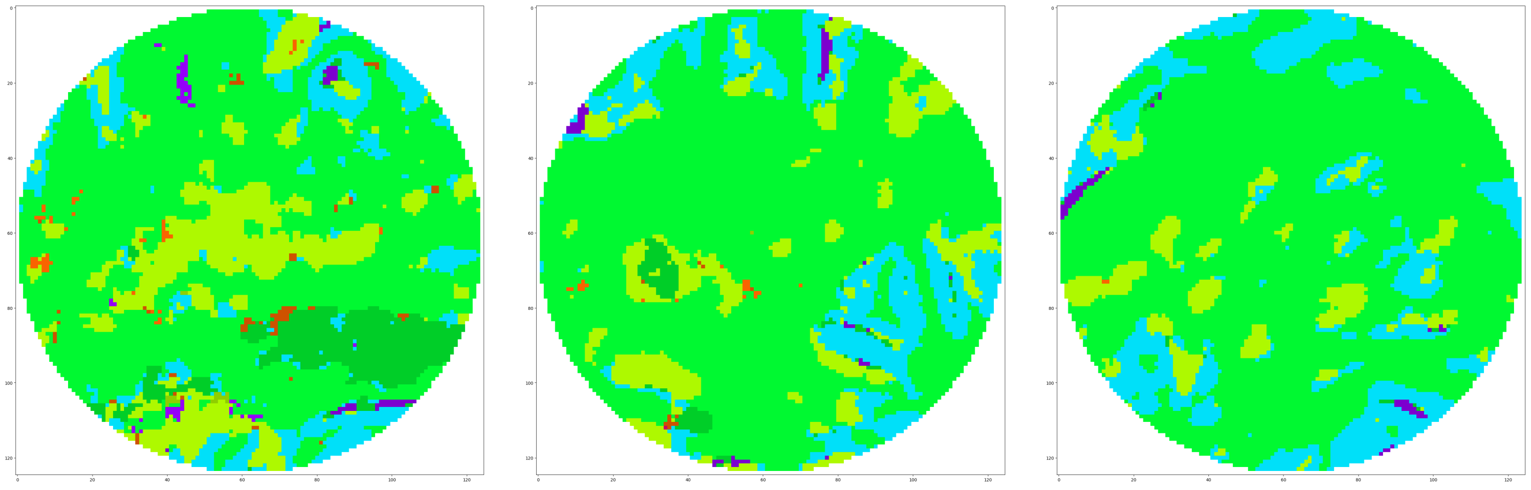

<u>Case 56</u>

Semi-automated output

Left: H&E stained section in the spot chosen by microscopic assessment. Middle: the first step was making an segmentation output. Right the class labels can be displayed

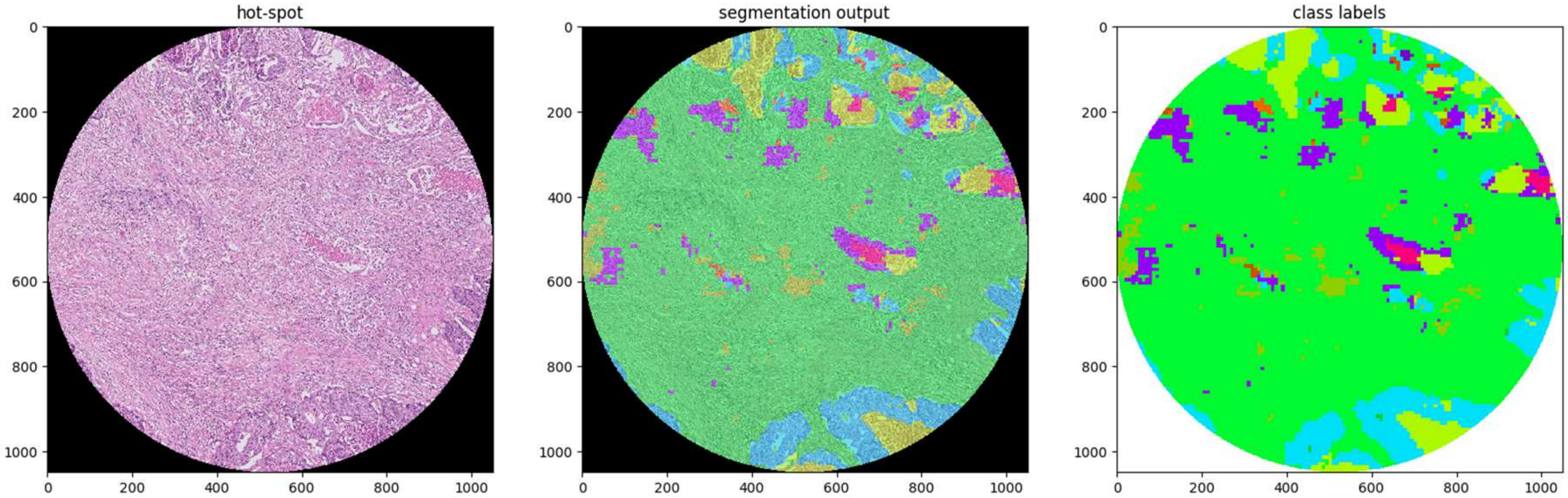

Fully-automated output

Top row; left: the tumor bulk is annotated. Right: heatmap is created. The biggest dot corresponds with the highest stroma-percentage (TSR-1), the second biggest with the second highest (TSR-2), etcetera.

Bottom row; left: the class output of the highest spot (TSR-1), middle: the second highest spot (TSR-2) and right the third highest spot (TSR-3)

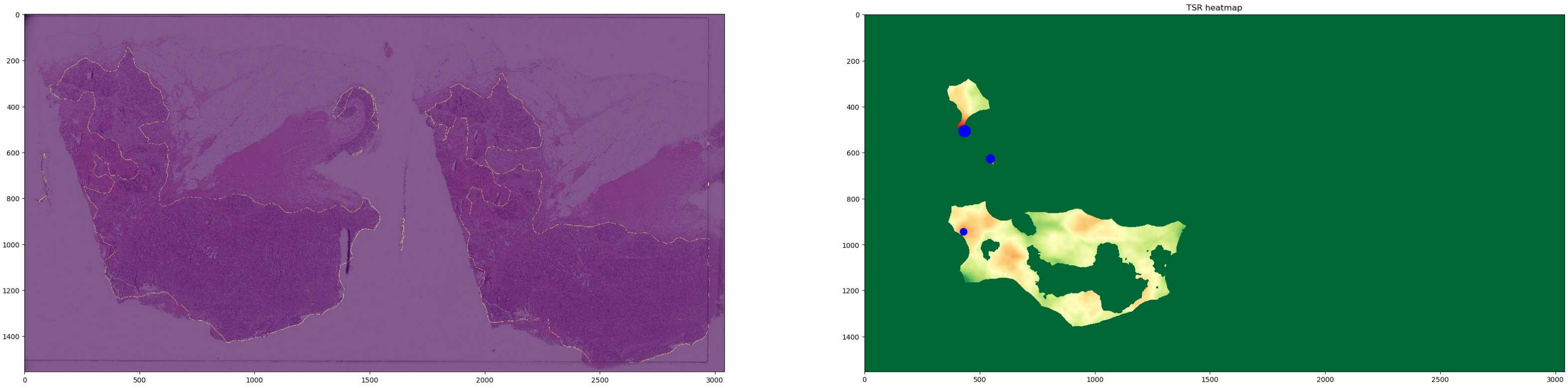

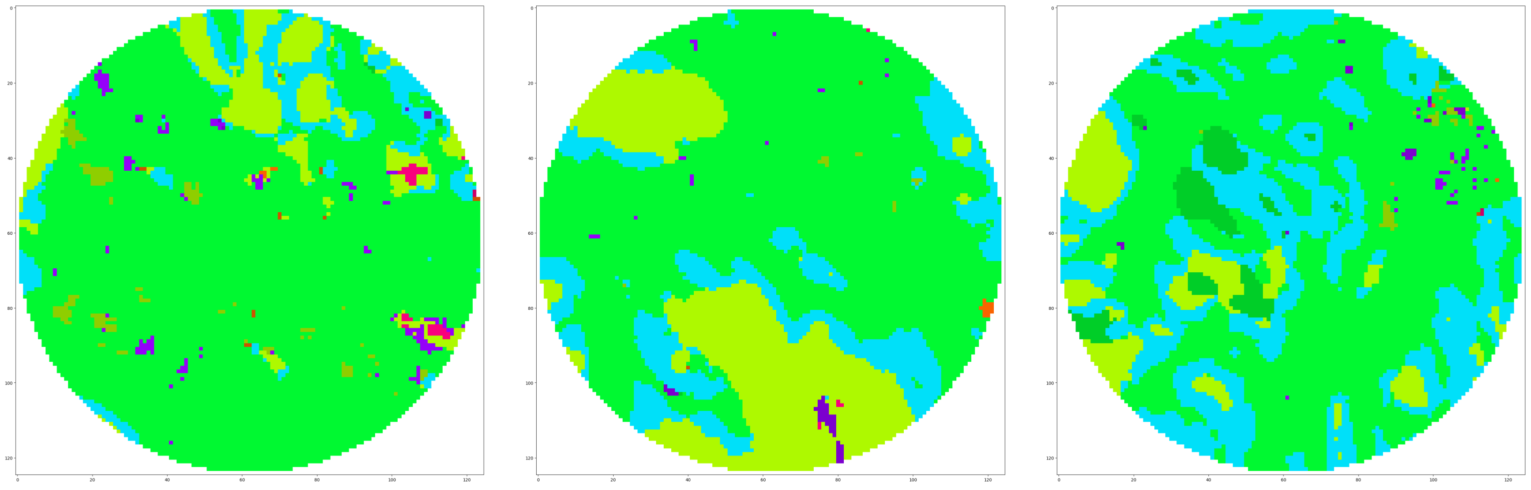

Case 57

Semi-automated output

Left: H&E stained section in the spot chosen by microscopic assessment. Middle: the first step was making an segmentation output. Right the class labels can be displayed

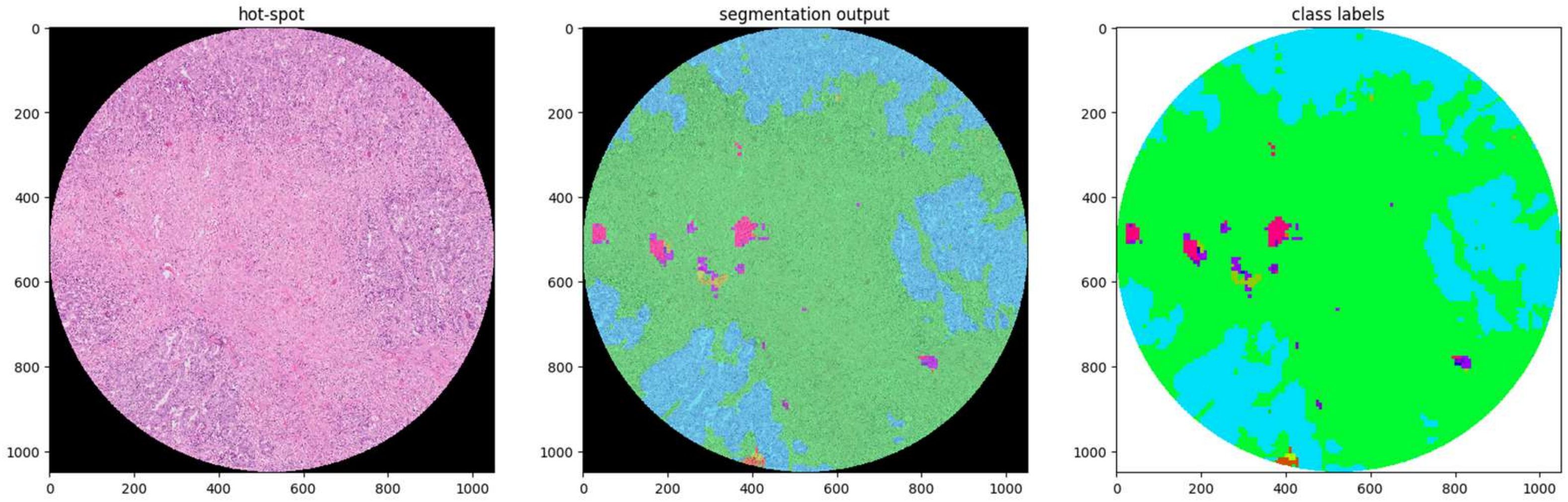

Fully-automated output

Top row; left: the tumor bulk is annotated. Right: heatmap is created. The biggest dot corresponds with the highest stroma-percentage (TSR-1), the second biggest with the second highest (TSR-2), etcetera.

Bottom row; left: the class output of the highest spot (TSR-1), middle: the second highest spot (TSR-2) and right the third highest spot (TSR-3)

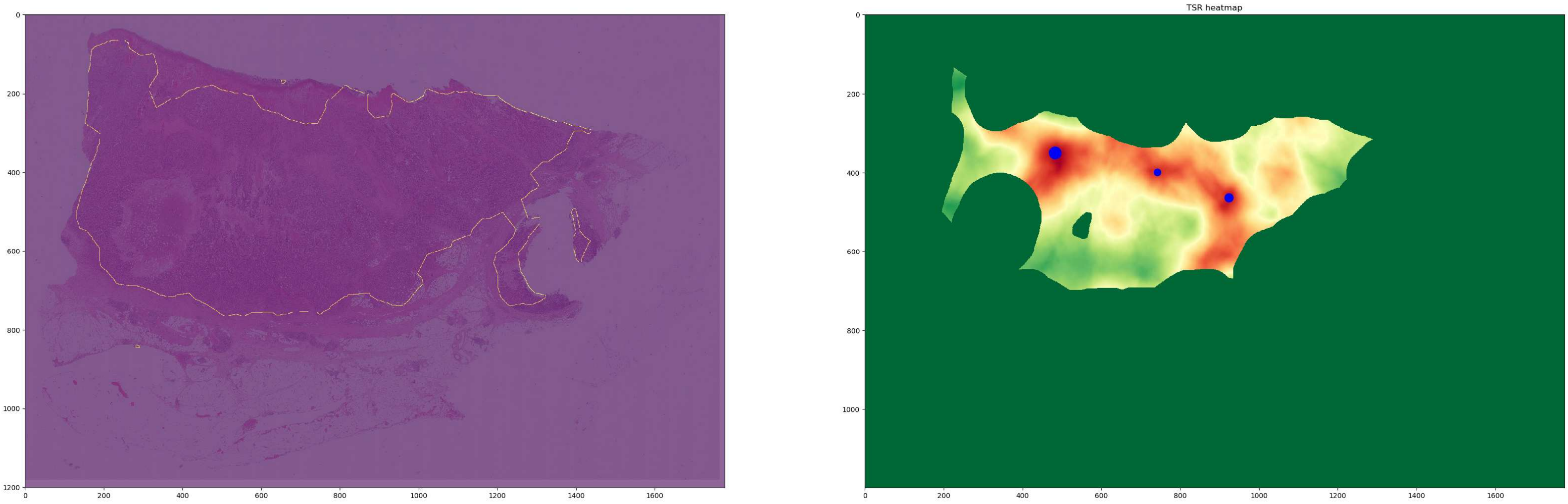

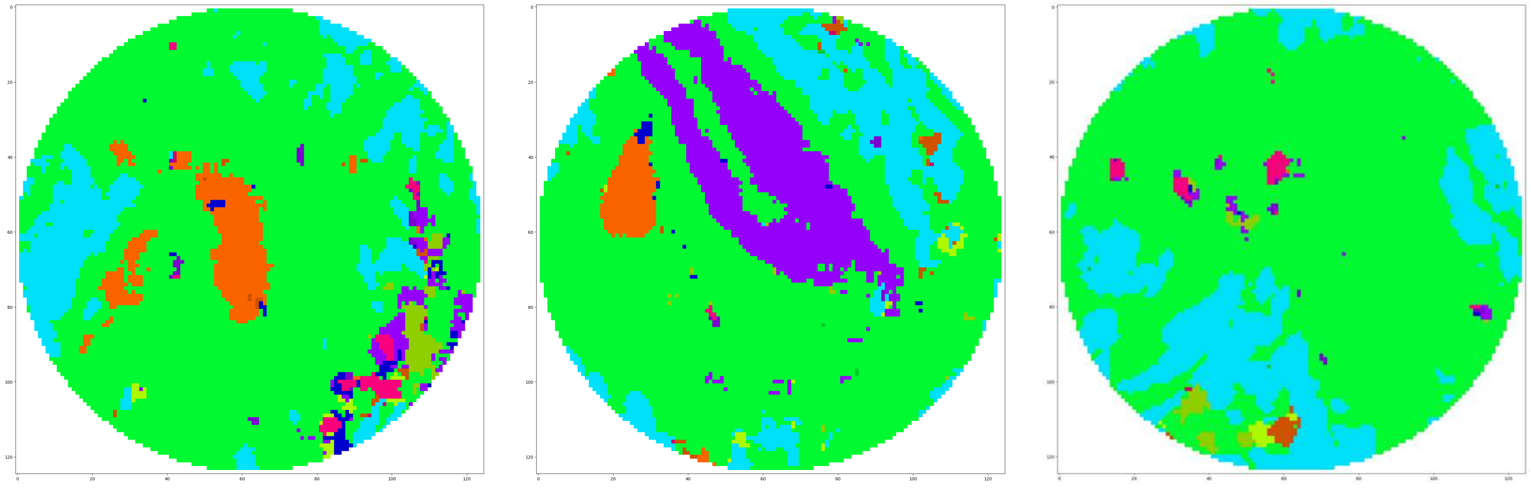

Case 58

Semi-automated output

Left: H&E stained section in the spot chosen by microscopic assessment. Middle: the first step was making an segmentation output. Right the class labels can be displayed

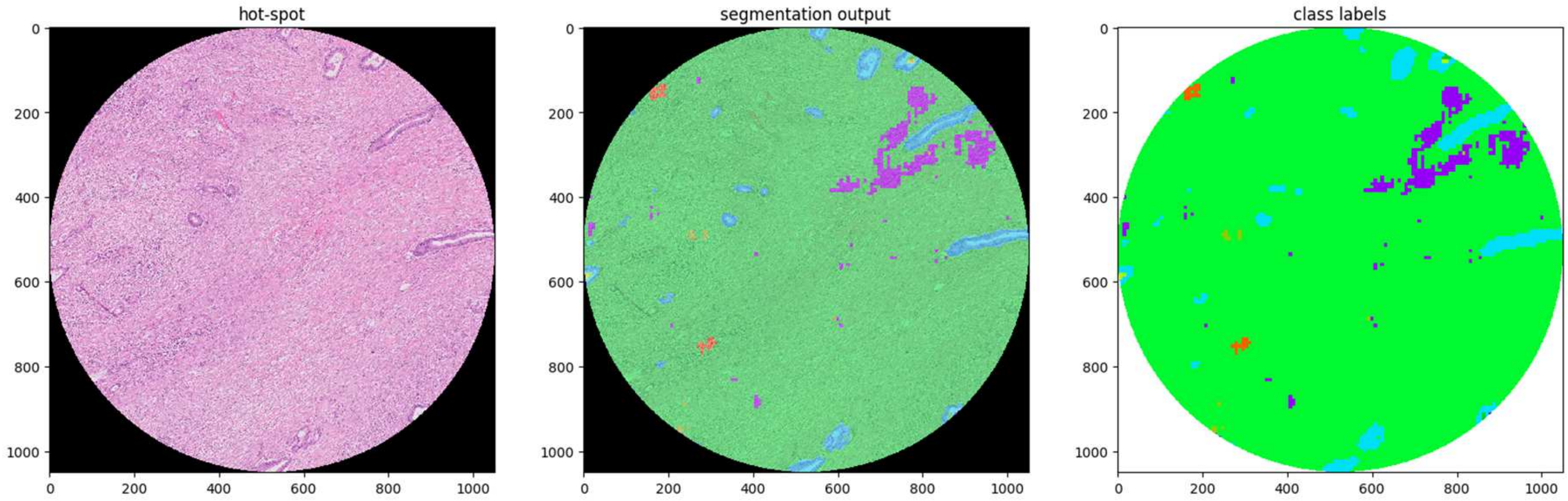

Fully-automated output

Top row; left: the tumor bulk is annotated. Right: heatmap is created. The biggest dot corresponds with the highest stroma-percentage (TSR-1), the second biggest with the second highest (TSR-2), etcetera.

Bottom row; left: the class output of the highest spot (TSR-1), middle: the second highest spot (TSR-2) and right the third highest spot (TSR-3)

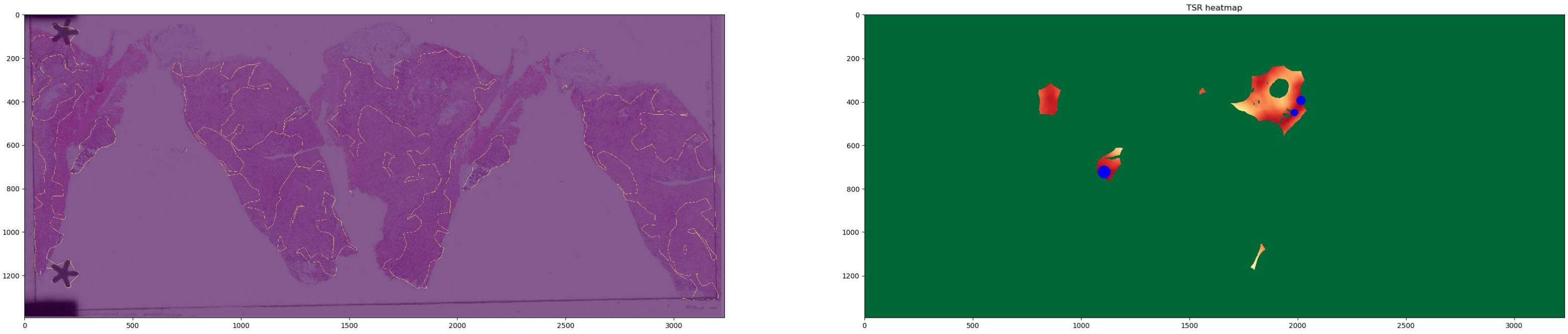

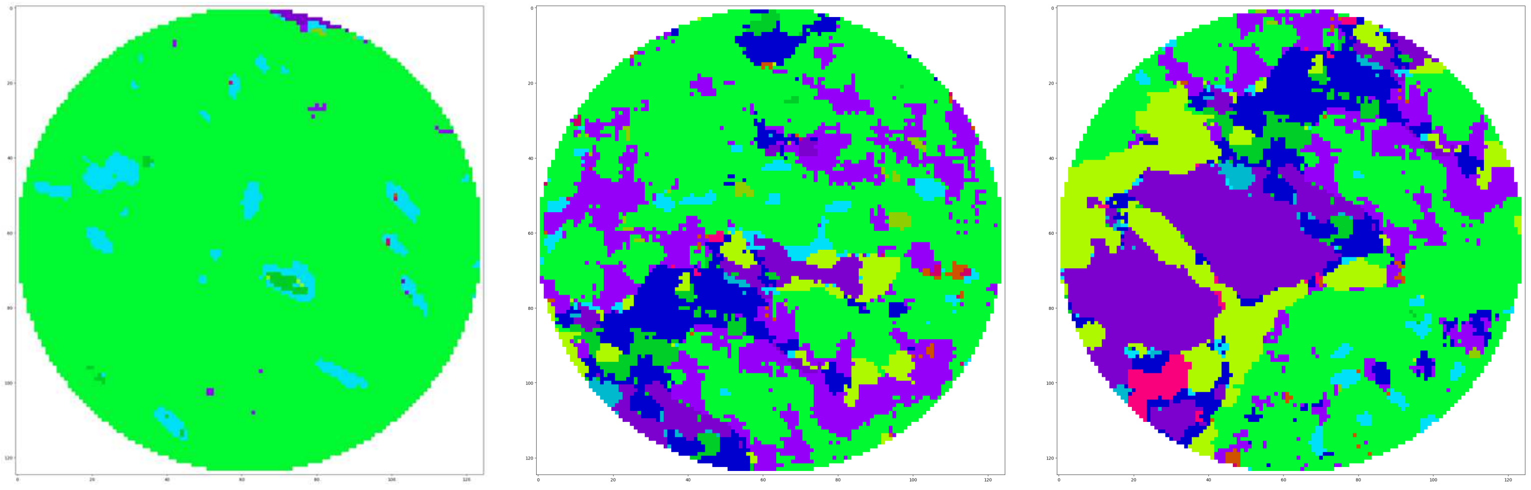

<u>Case 59</u>

Semi-automated output

Left: H&E stained section in the spot chosen by microscopic assessment. Middle: the first step was making an segmentation output. Right the class labels can be displayed

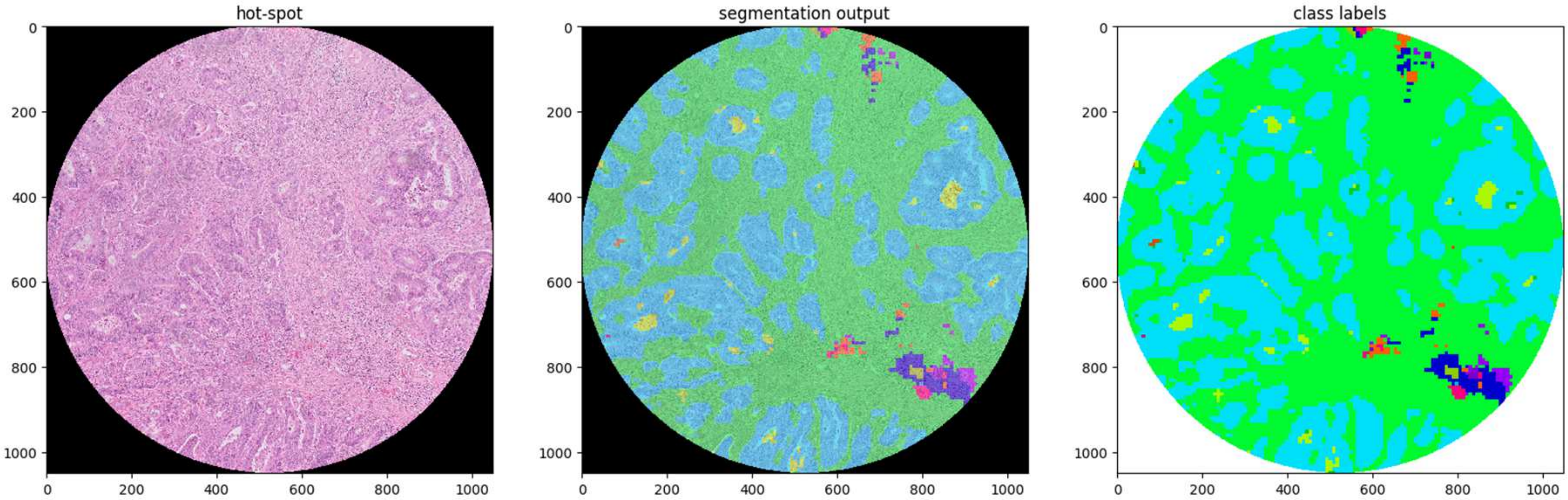

Fully-automated output

Top row; left: the tumor bulk is annotated. Right: heatmap is created. The biggest dot corresponds with the highest stroma-percentage (TSR-1), the second biggest with the second highest (TSR-2), etcetera.

Bottom row; left: the class output of the highest spot (TSR-1), middle: the second highest spot (TSR-2) and right the third highest spot (TSR-3)

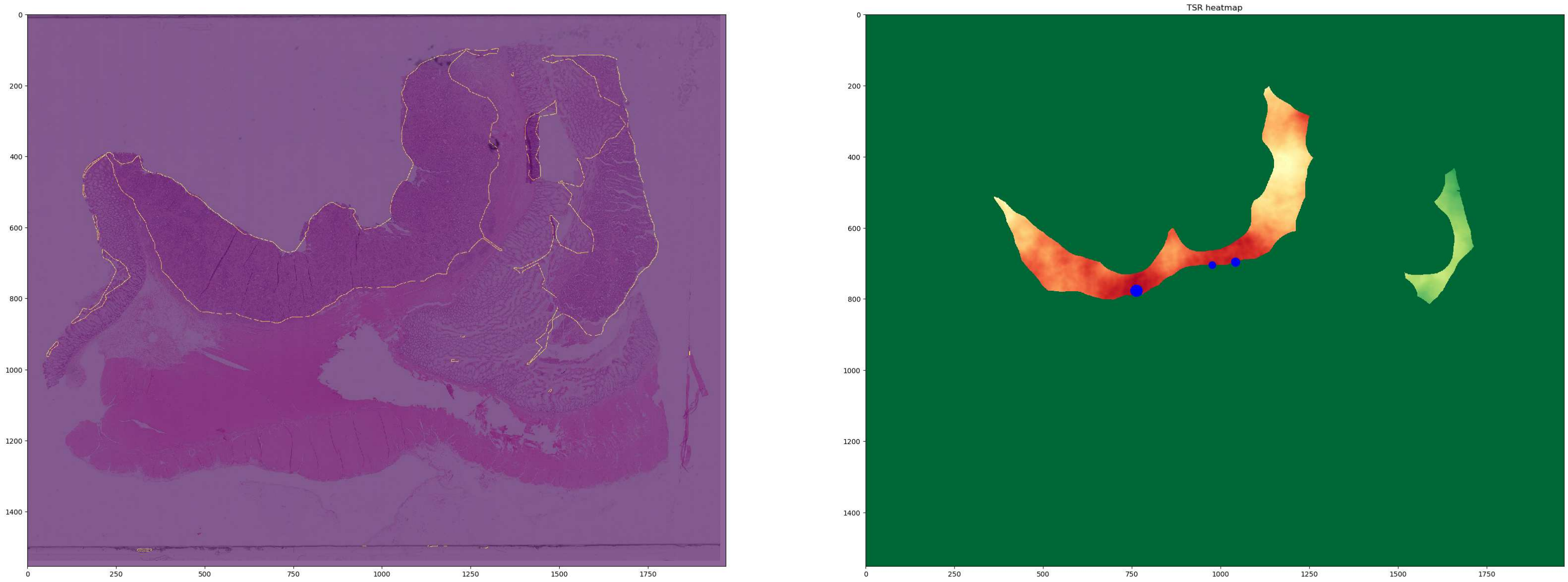

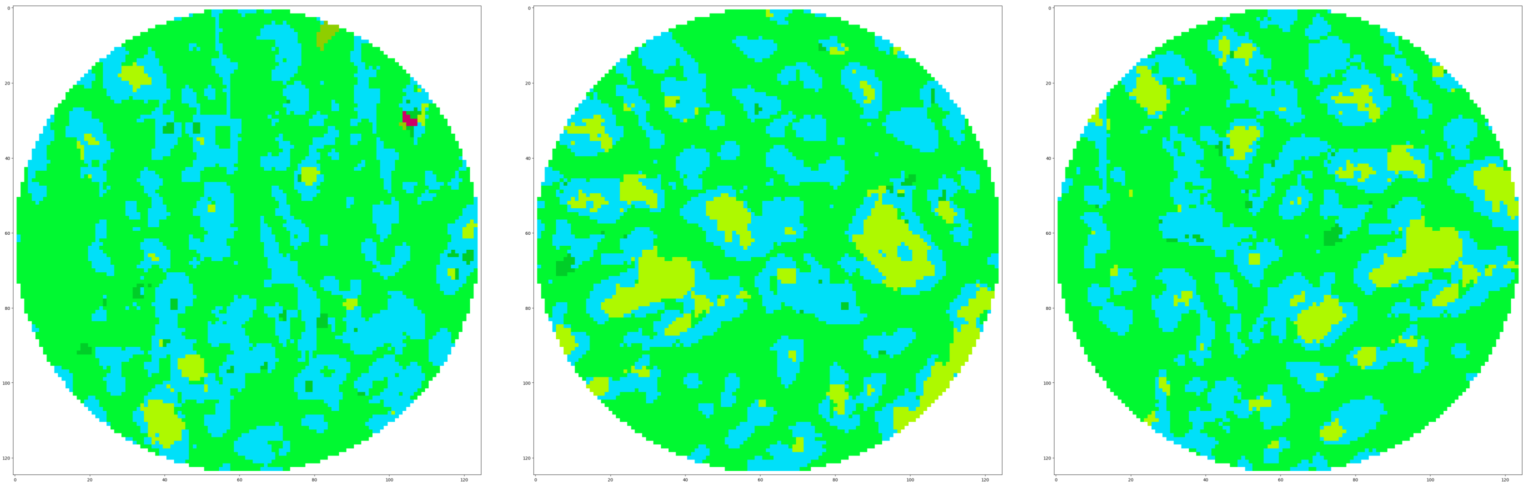

Case 60

Semi-automated output

Left: H&E stained section in the spot chosen by microscopic assessment. Middle: the first step was making an segmentation output. Right the class labels can be displayed

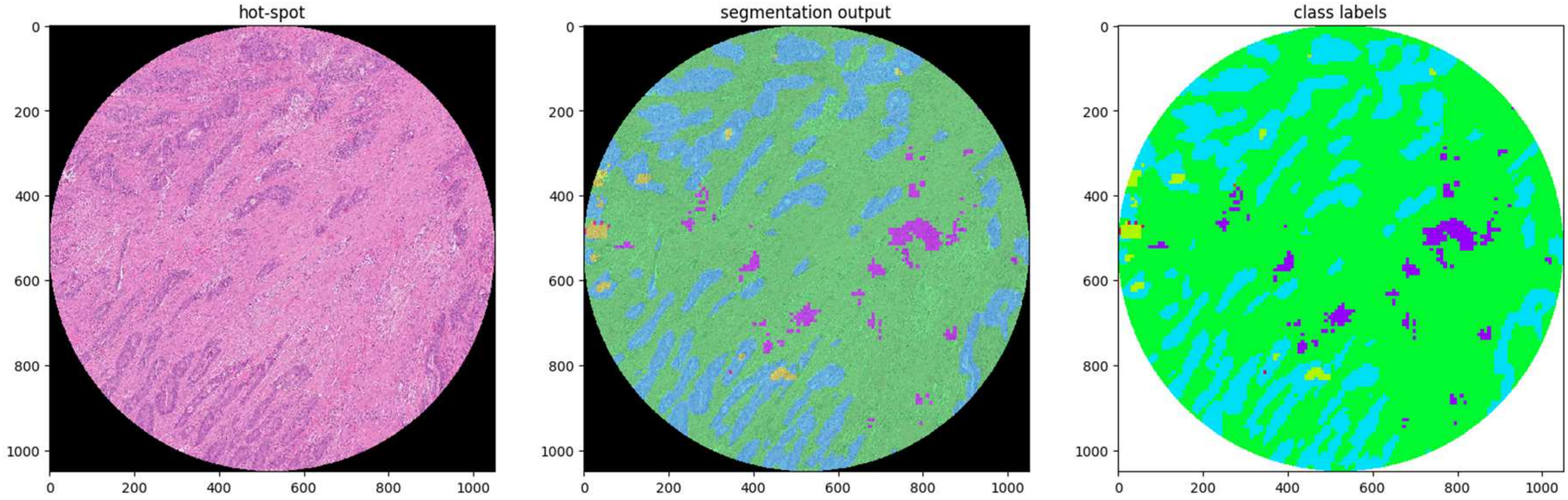

Fully-automated output

Top row; left: the tumor bulk is annotated. Right: heatmap is created. The biggest dot corresponds with the highest stroma-percentage (TSR-1), the second biggest with the second highest (TSR-2), etcetera.

Bottom row; left: the class output of the highest spot (TSR-1), middle: the second highest spot (TSR-2) and right the third highest spot (TSR-3)

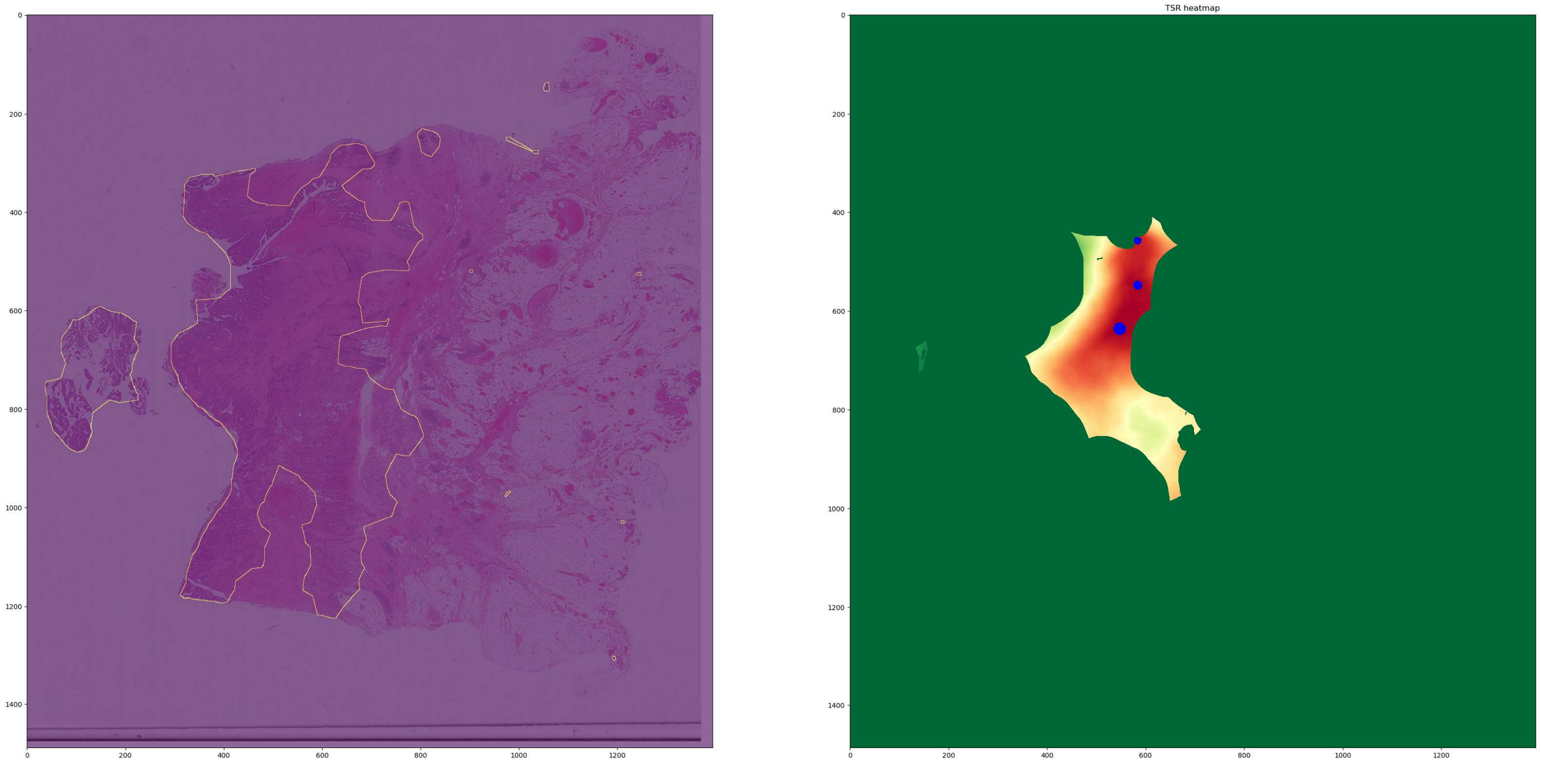

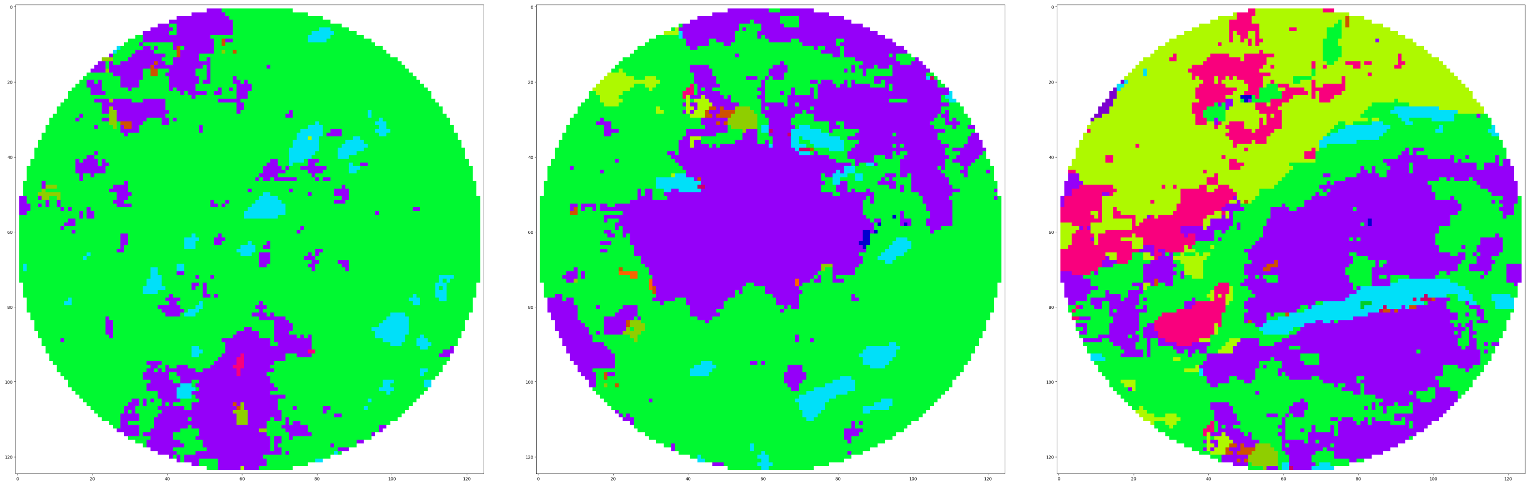

<u>Case 61</u>

Semi-automated output

Left: H&E stained section in the spot chosen by microscopic assessment. Middle: the first step was making an segmentation output. Right the class labels can be displayed

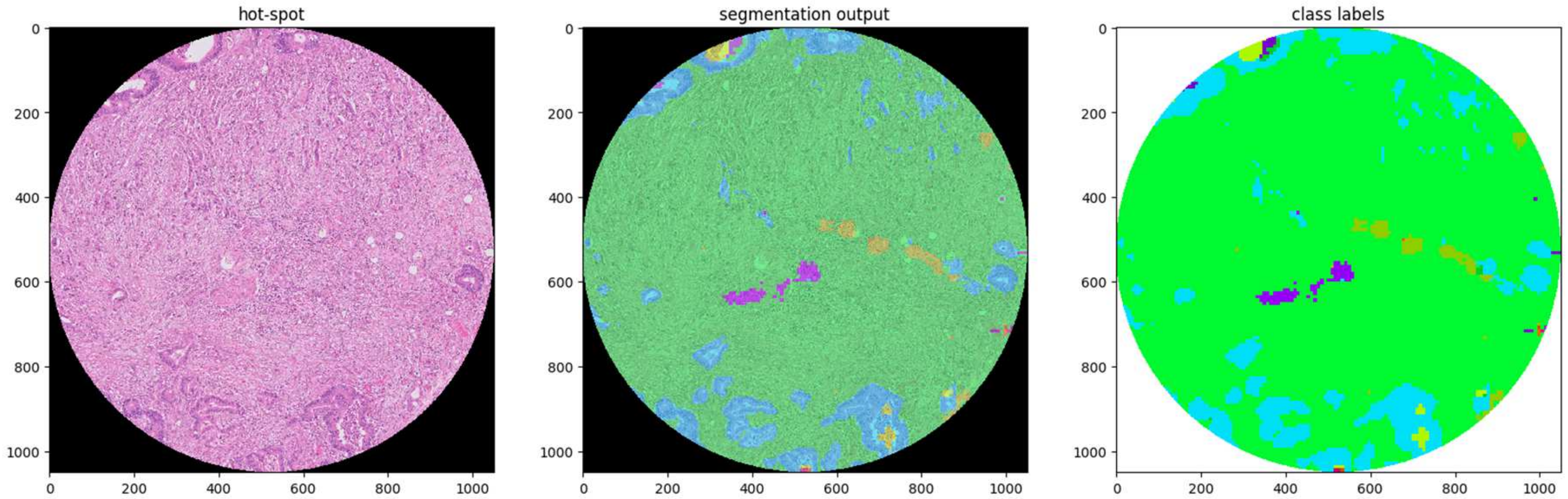

Fully-automated output

Top row; left: the tumor bulk is annotated. Right: heatmap is created. The biggest dot corresponds with the highest stroma-percentage (TSR-1), the second biggest with the second highest (TSR-2), etcetera.

Bottom row; left: the class output of the highest spot (TSR-1), middle: the second highest spot (TSR-2) and right the third highest spot (TSR-3)

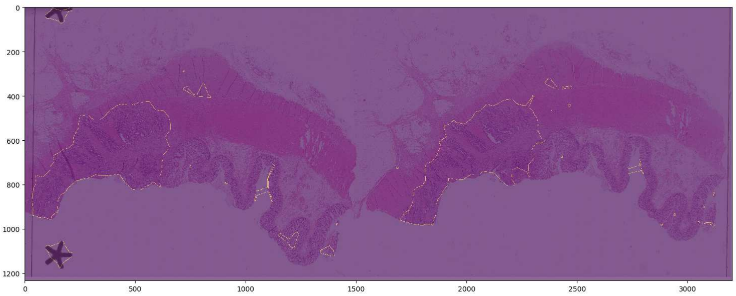

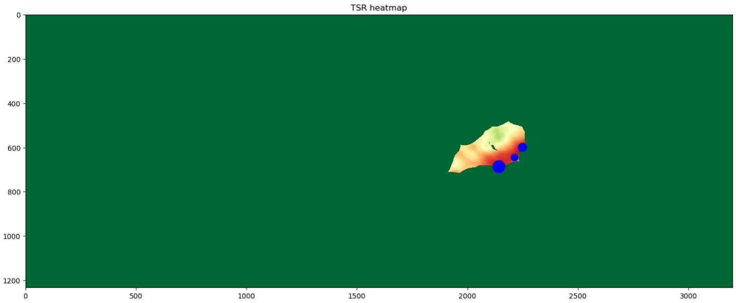

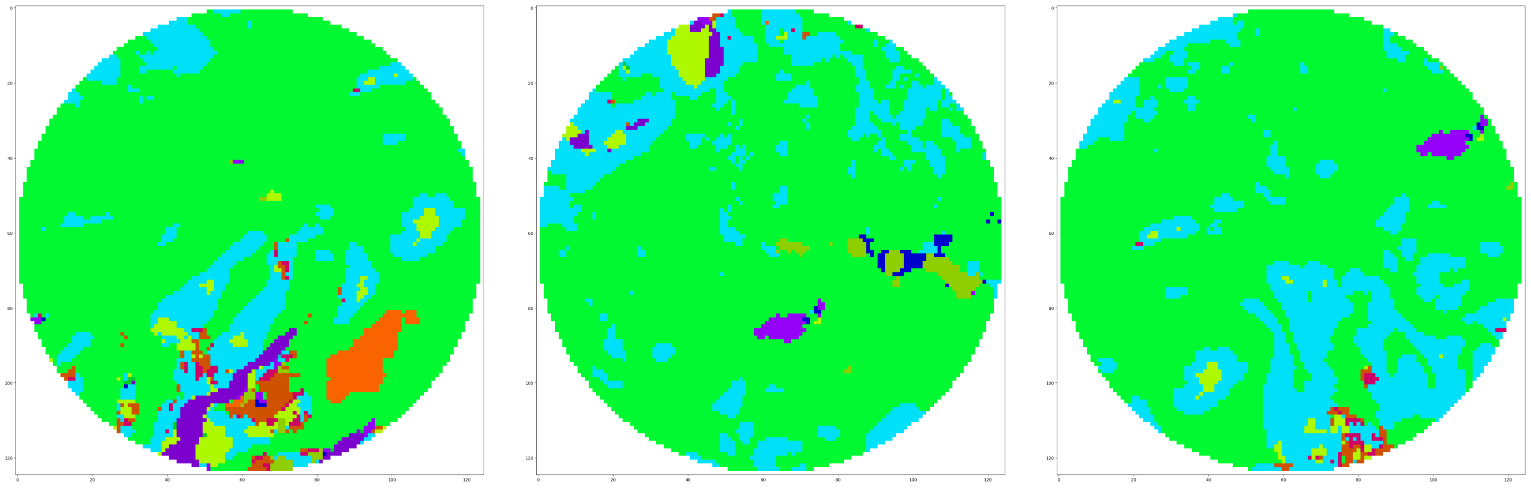

Case 62

Semi-automated output

Left: H&E stained section in the spot chosen by microscopic assessment. Middle: the first step was making an segmentation output. Right the class labels can be displayed

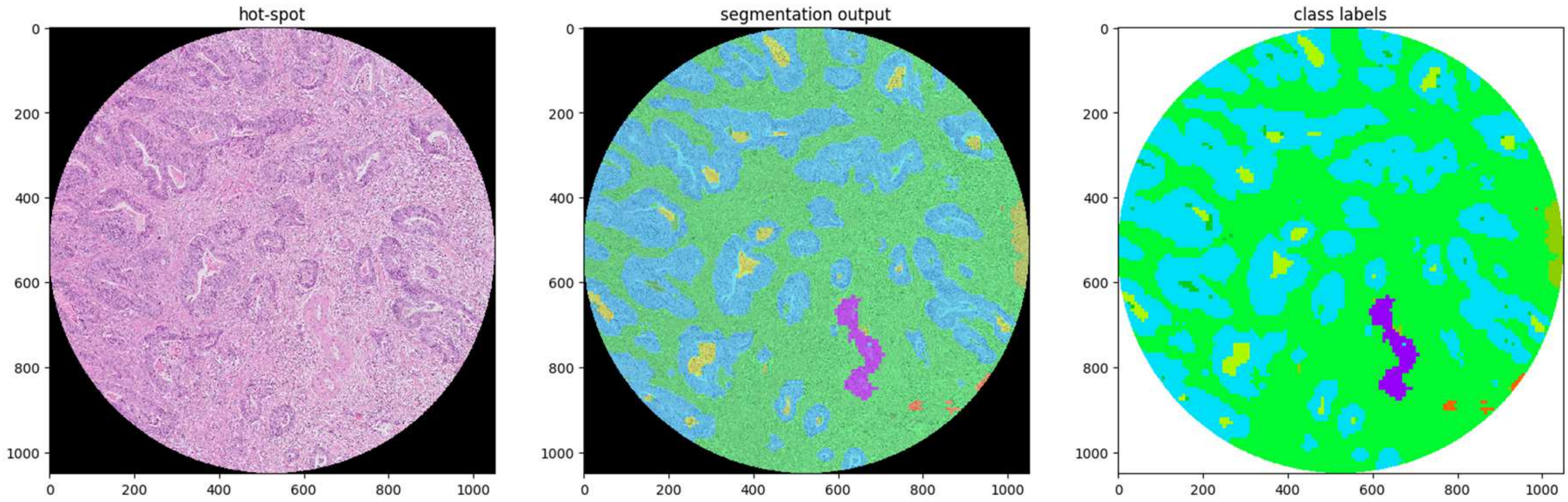

Fully-automated output

Top row; left: the tumor bulk is annotated. Right: heatmap is created. The biggest dot corresponds with the highest stroma-percentage (TSR-1), the second biggest with the second highest (TSR-2), etcetera.

Bottom row; left: the class output of the highest spot (TSR-1), middle: the second highest spot (TSR-2) and right the third highest spot (TSR-3)

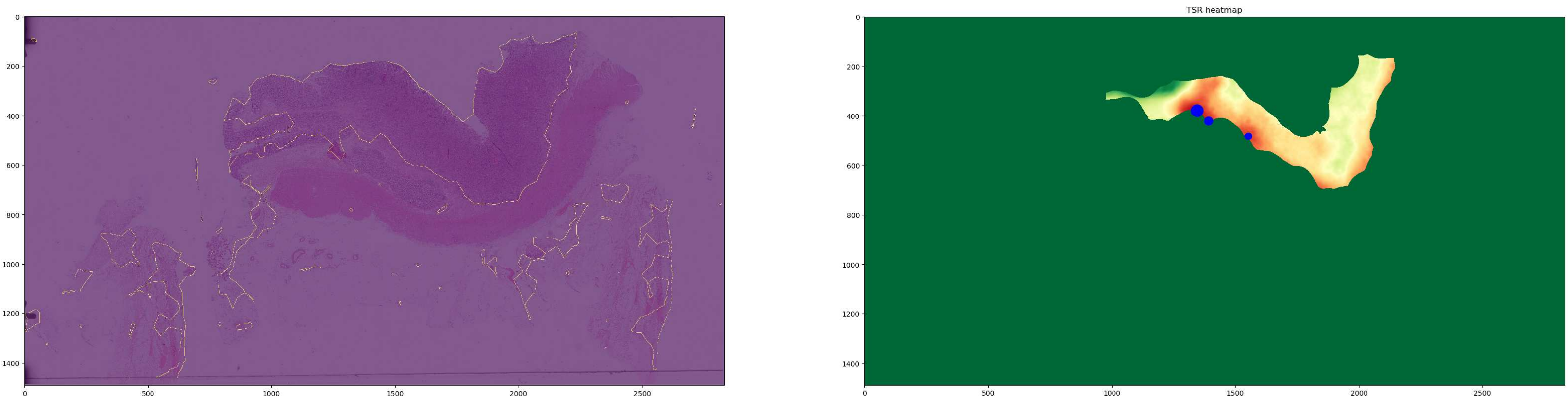

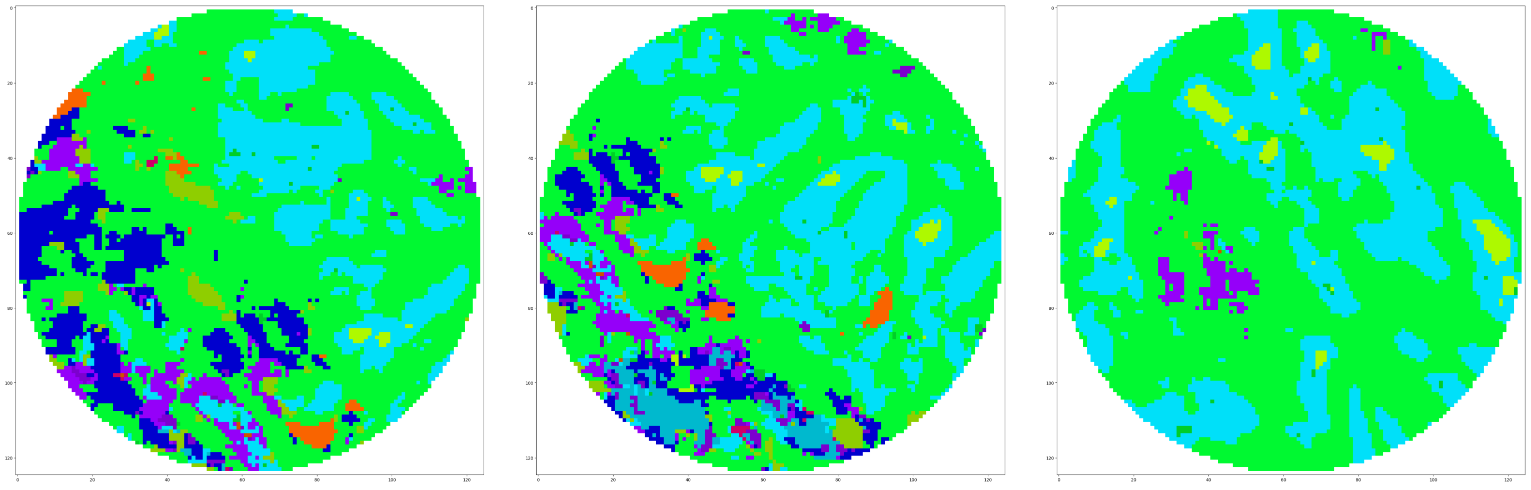

<u>Case 63</u>

Semi-automated output

Left: H&E stained section in the spot chosen by microscopic assessment. Middle: the first step was making an segmentation output. Right the class labels can be displayed

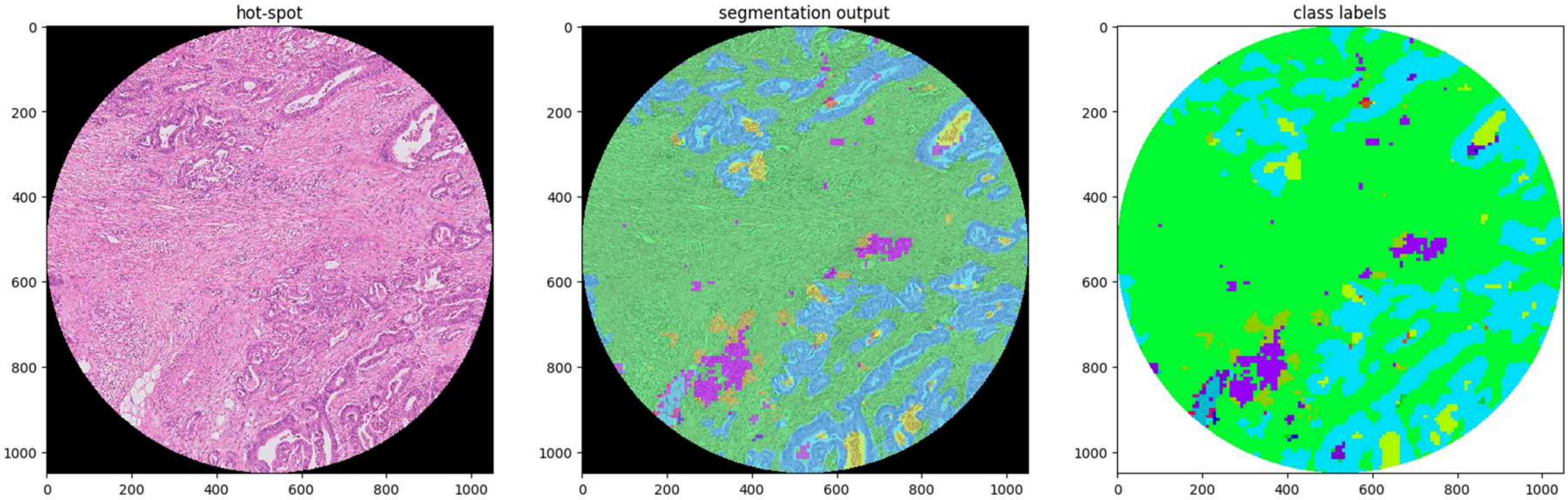

Fully-automated output

Top row; left: the tumor bulk is annotated. Right: heatmap is created. The biggest dot corresponds with the highest stroma-percentage (TSR-1), the second biggest with the second highest (TSR-2), etcetera.

Bottom row; left: the class output of the highest spot (TSR-1), middle: the second highest spot (TSR-2) and right the third highest spot (TSR-3)

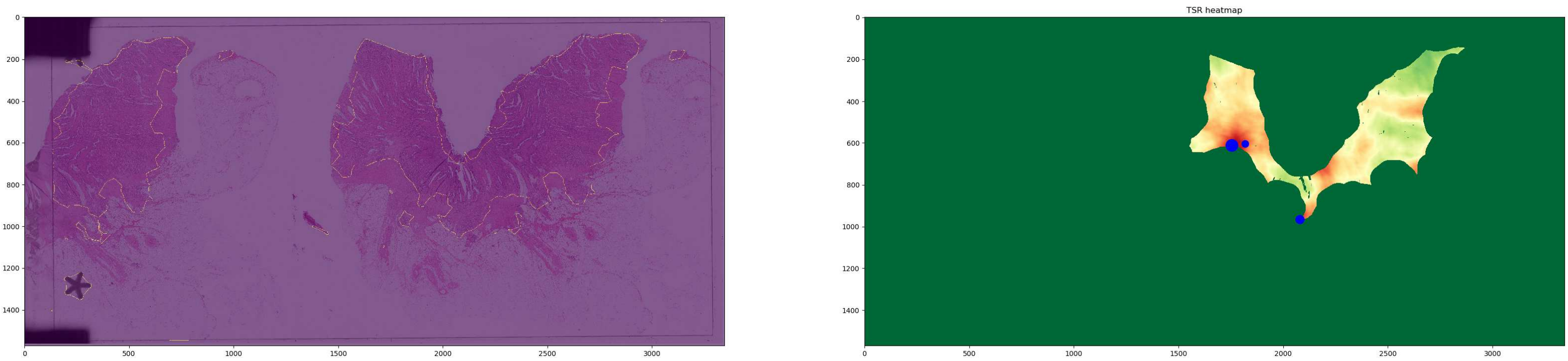

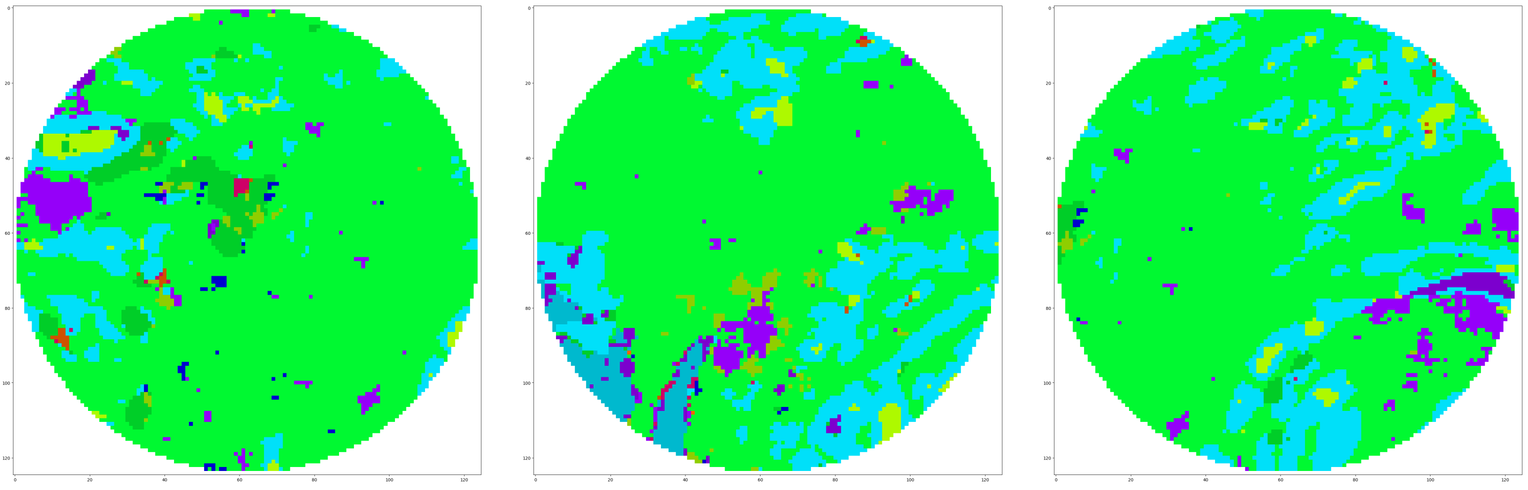

Case 64

Semi-automated output

Left: H&E stained section in the spot chosen by microscopic assessment. Middle: the first step was making an segmentation output. Right the class labels can be displayed

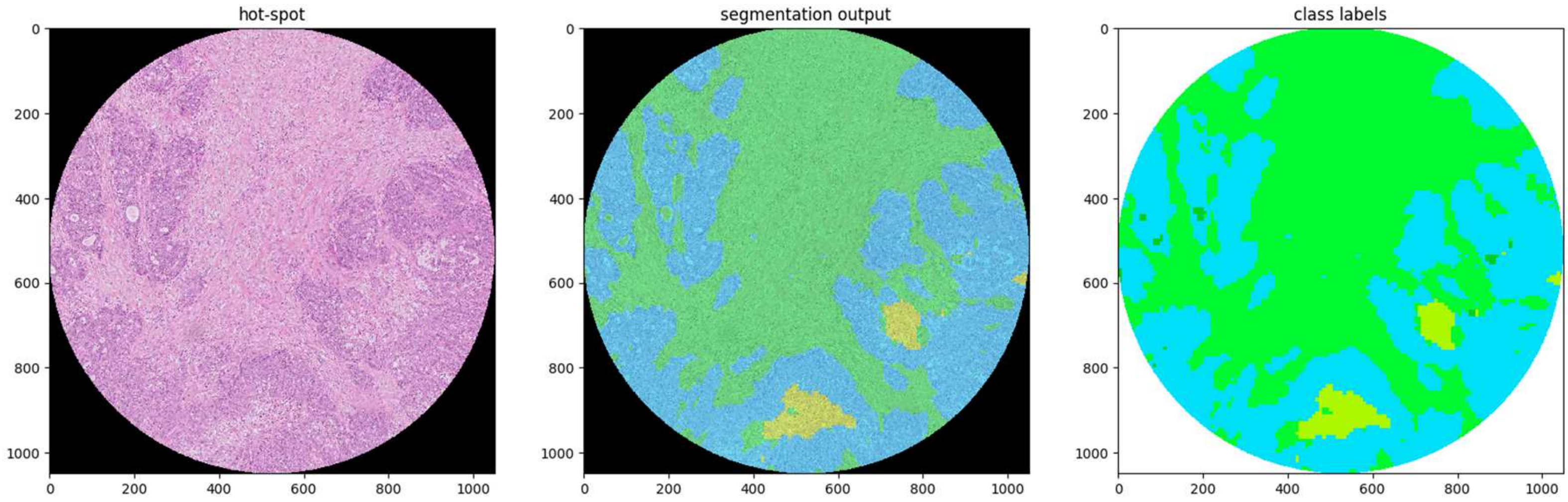

Fully-automated output

Top row; left: the tumor bulk is annotated. Right: heatmap is created. The biggest dot corresponds with the highest stroma-percentage (TSR-1), the second biggest with the second highest (TSR-2), etcetera.

Bottom row; left: the class output of the highest spot (TSR-1), middle: the second highest spot (TSR-2) and right the third highest spot (TSR-3)

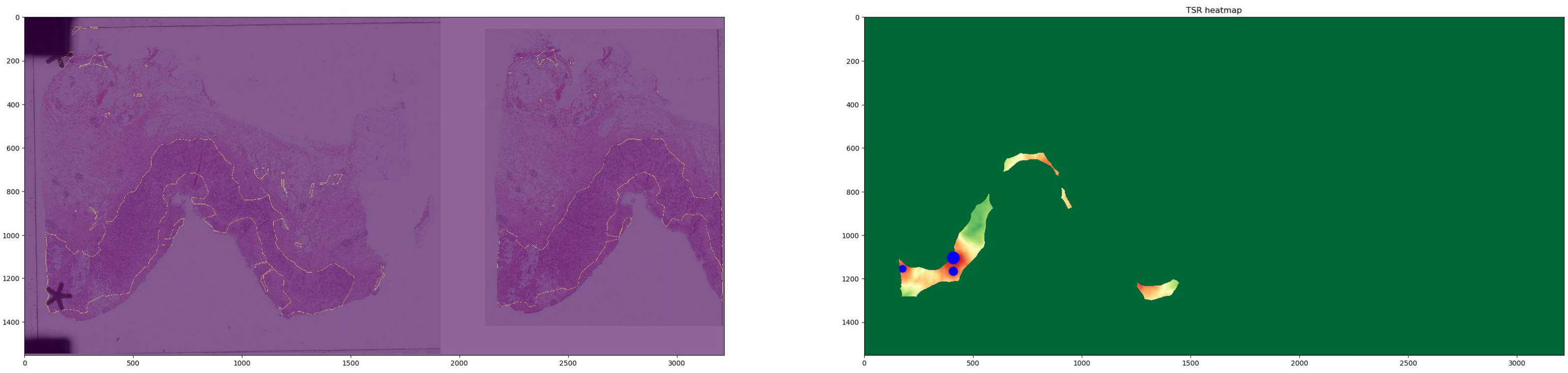

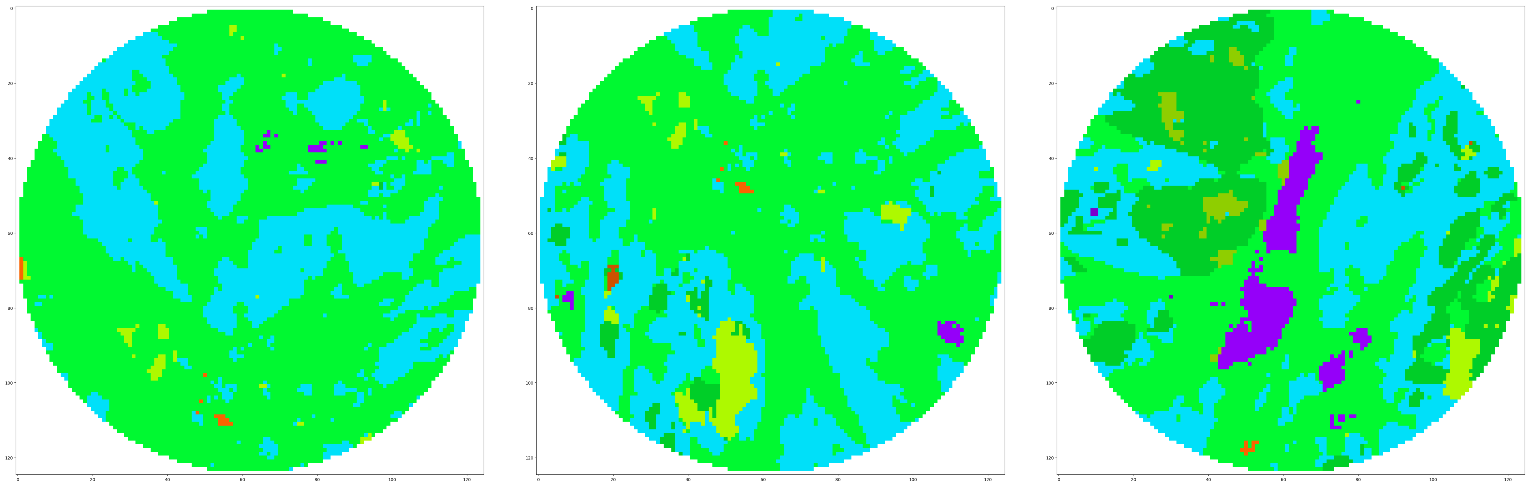

<u>Case 65</u>

Semi-automated output

Left: H&E stained section in the spot chosen by microscopic assessment. Middle: the first step was making an segmentation output. Right the class labels can be displayed

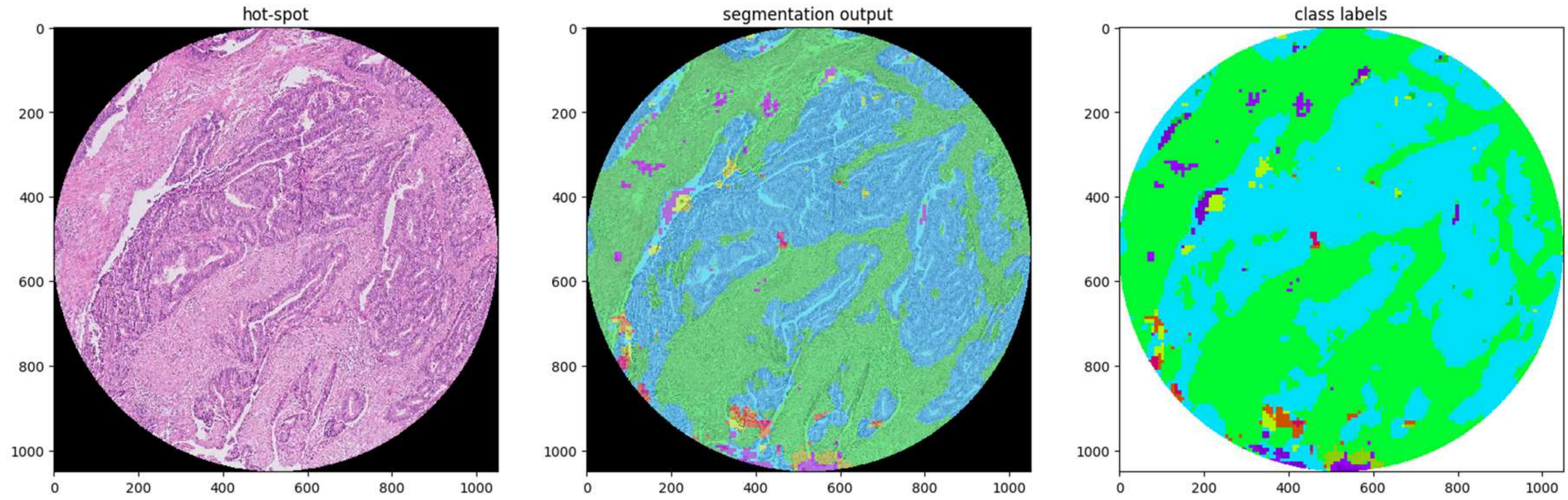

Fully-automated output

Top row; left: the tumor bulk is annotated. Right: heatmap is created. The biggest dot corresponds with the highest stroma-percentage (TSR-1), the second biggest with the second highest (TSR-2), etcetera.

Bottom row; left: the class output of the highest spot (TSR-1), middle: the second highest spot (TSR-2) and right the third highest spot (TSR-3)

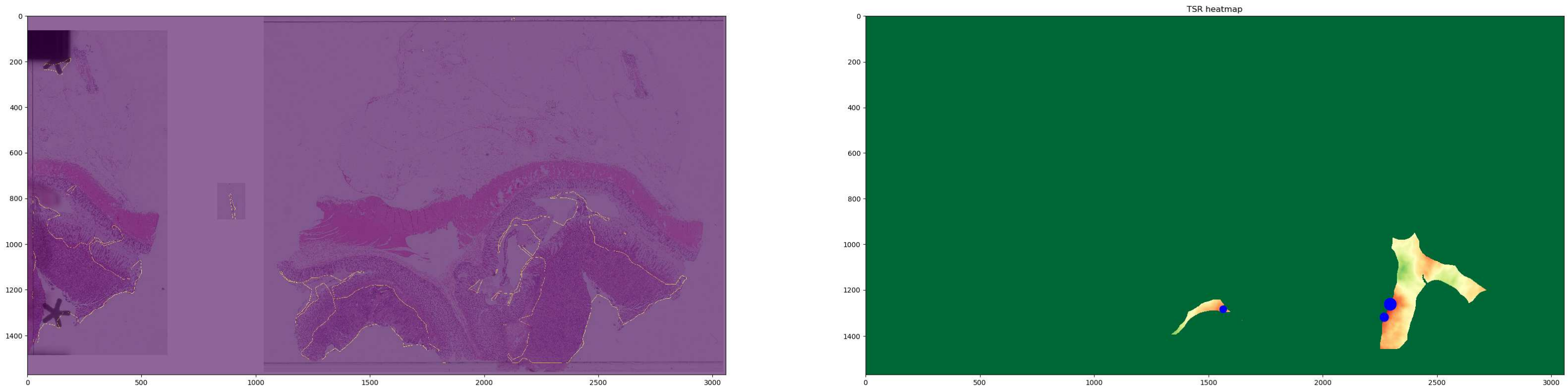

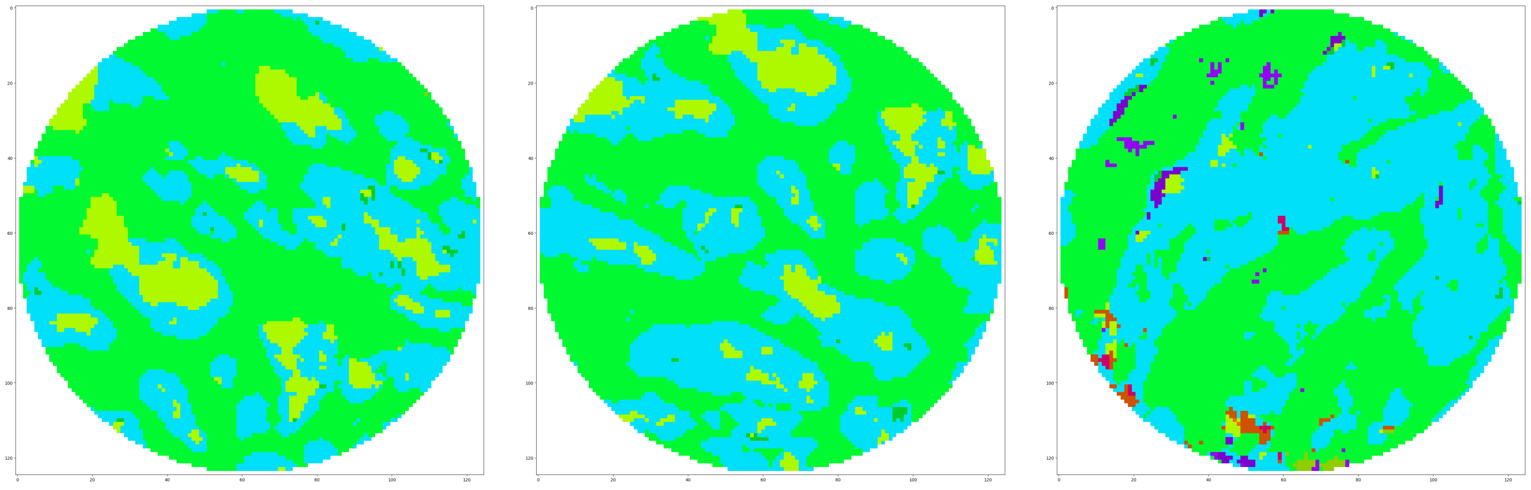

Case 66

Semi-automated output

Left: H&E stained section in the spot chosen by microscopic assessment. Middle: the first step was making an segmentation output. Right the class labels can be displayed

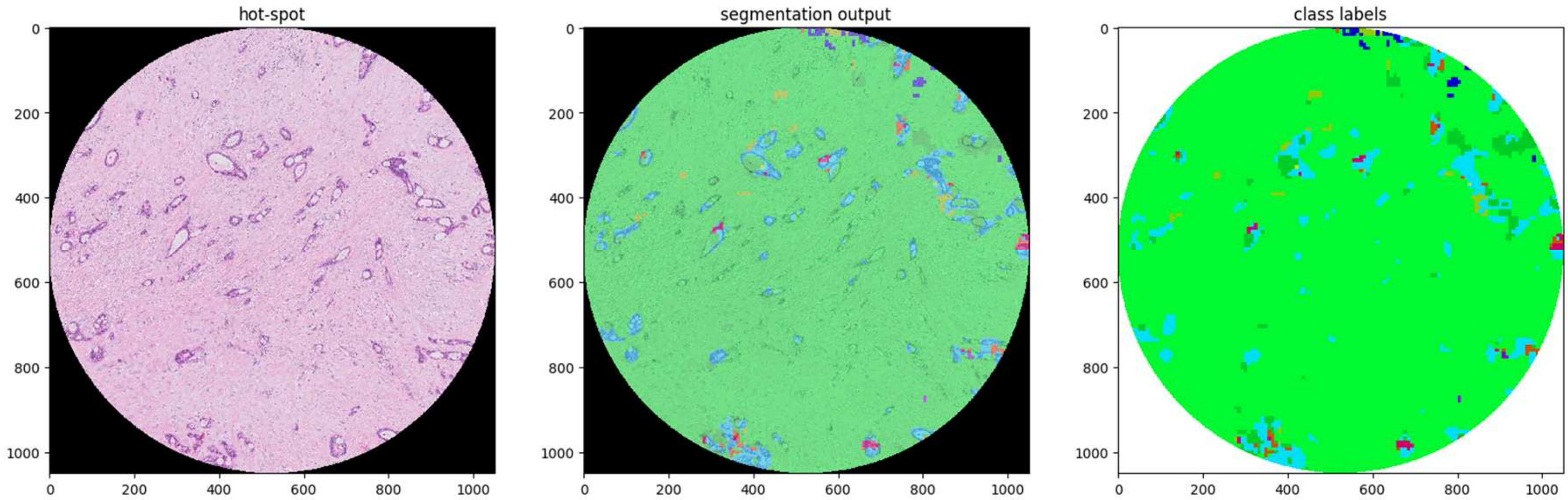

Fully-automated output

Top row; left: the tumor bulk is annotated. Right: heatmap is created. The biggest dot corresponds with the highest stroma-percentage (TSR-1), the second biggest with the second highest (TSR-2), etcetera.

Bottom row; left: the class output of the highest spot (TSR-1), middle: the second highest spot (TSR-2) and right the third highest spot (TSR-3)

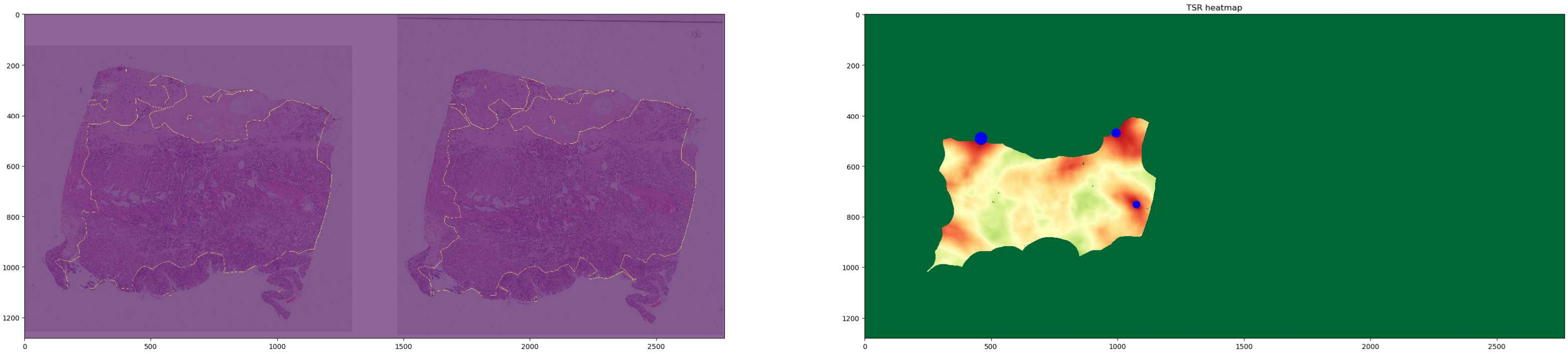

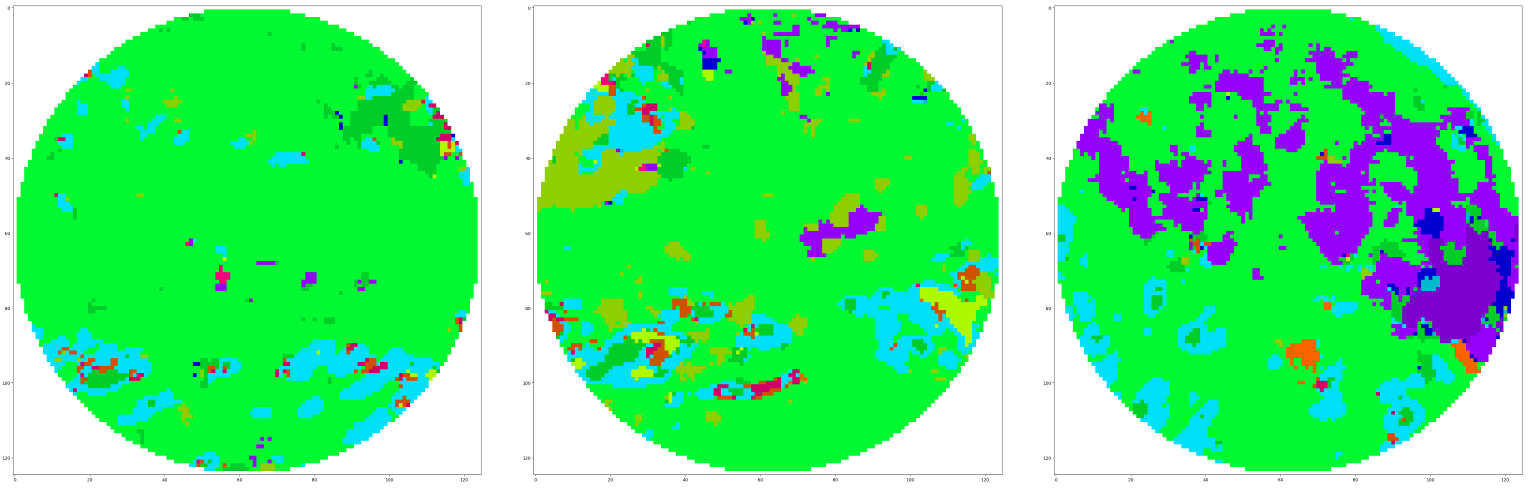

<u>Case 67</u>

Semi-automated output

Left: H&E stained section in the spot chosen by microscopic assessment. Middle: the first step was making an segmentation output. Right the class labels can be displayed

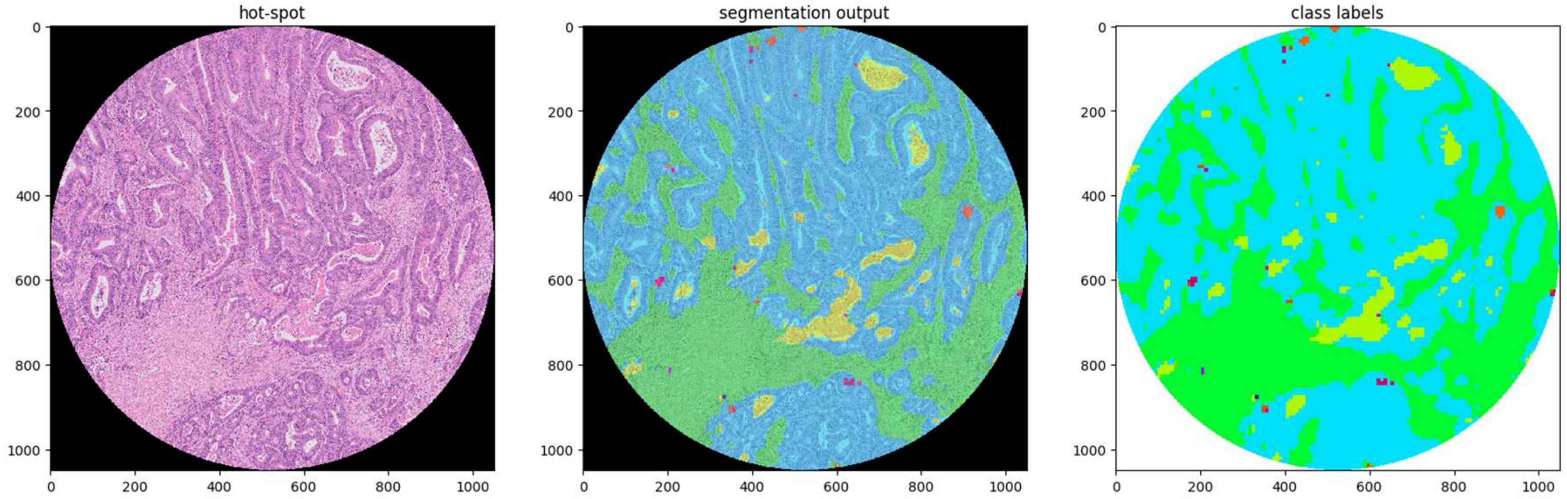

Fully-automated output

Top row; left: the tumor bulk is annotated. Right: heatmap is created. The biggest dot corresponds with the highest stroma-percentage (TSR-1), the second biggest with the second highest (TSR-2), etcetera.

Bottom row; left: the class output of the highest spot (TSR-1), middle: the second highest spot (TSR-2) and right the third highest spot (TSR-3)

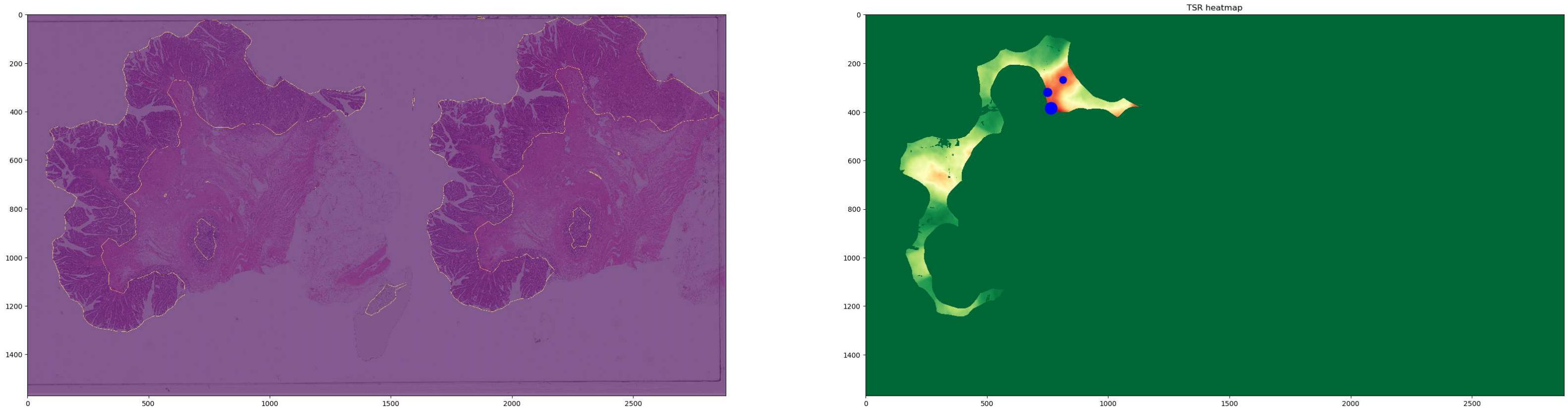

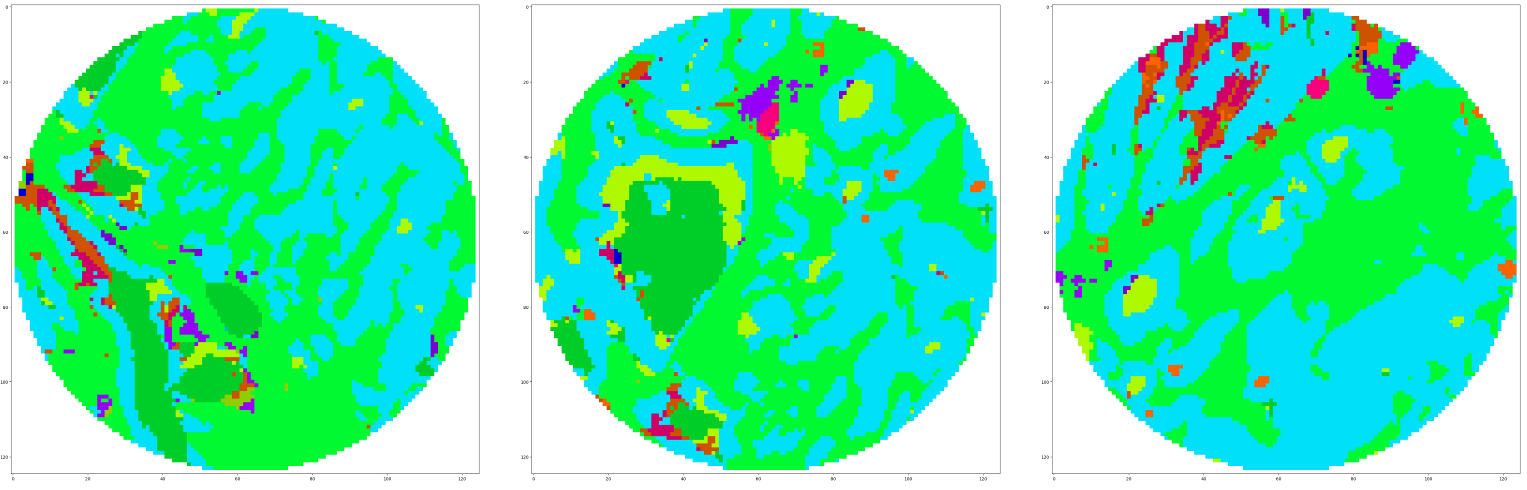

Case 68

Semi-automated output

Left: H&E stained section in the spot chosen by microscopic assessment. Middle: the first step was making an segmentation output. Right the class labels can be displayed

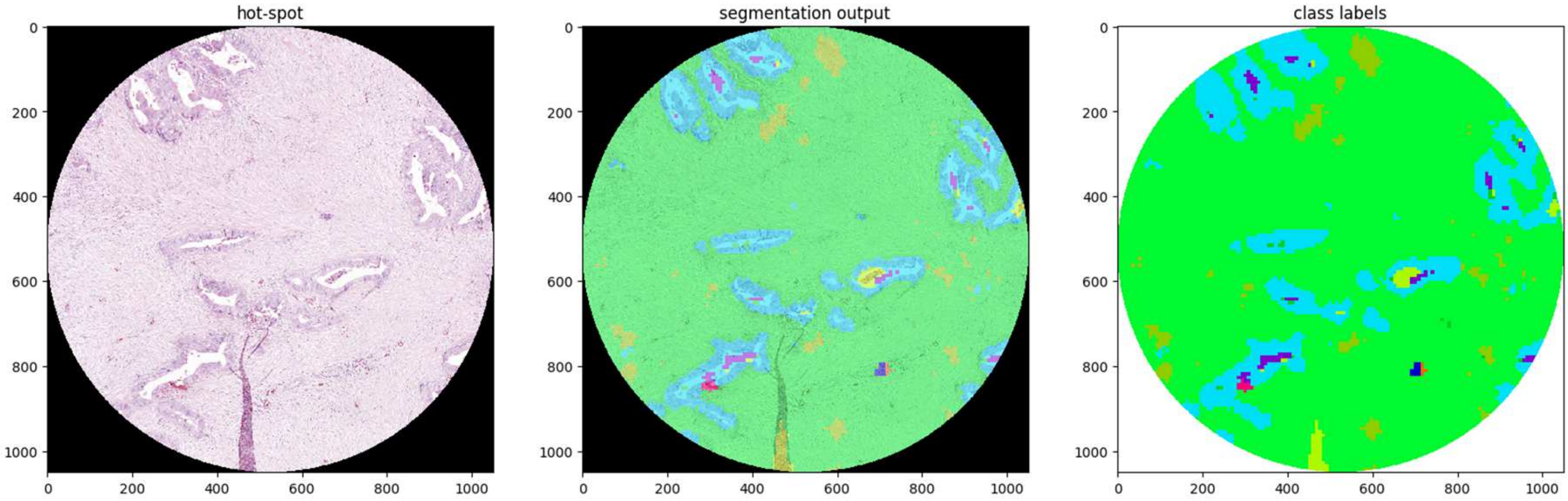

Fully-automated output

Top row; left: the tumor bulk is annotated. Right: heatmap is created. The biggest dot corresponds with the highest stroma-percentage (TSR-1), the second biggest with the second highest (TSR-2), etcetera.

Bottom row; left: the class output of the highest spot (TSR-1), middle: the second highest spot (TSR-2) and right the third highest spot (TSR-3)

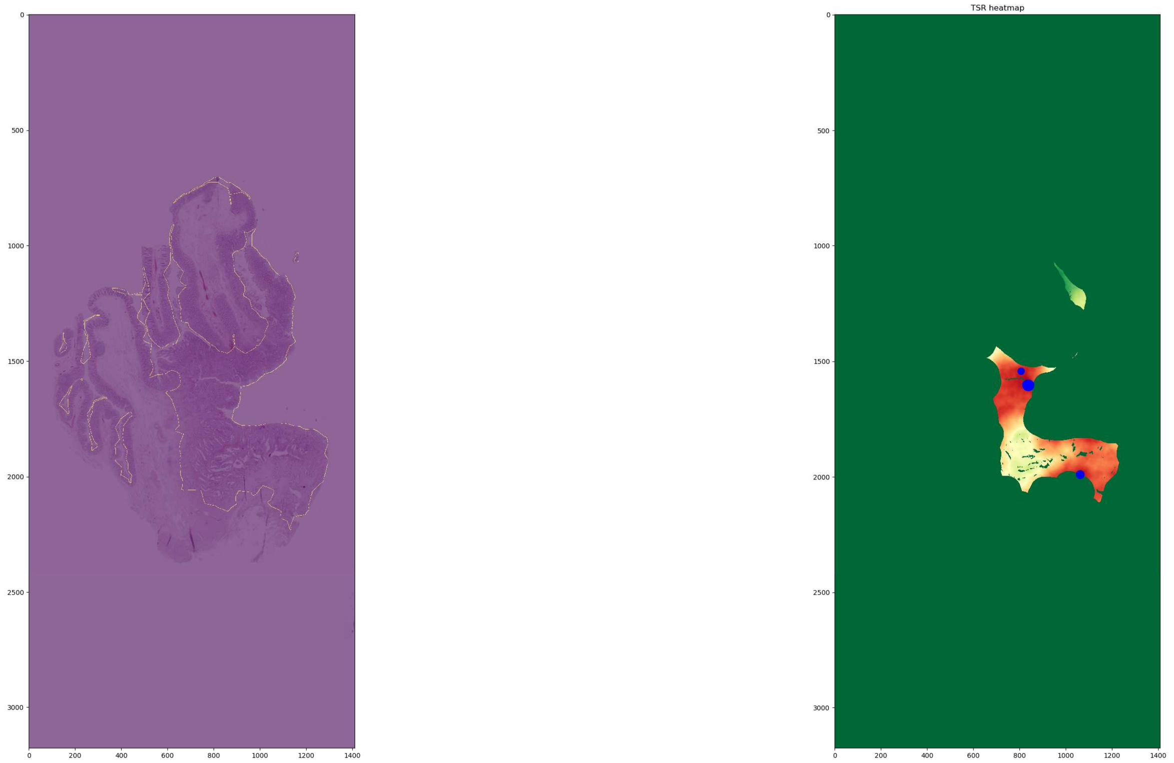

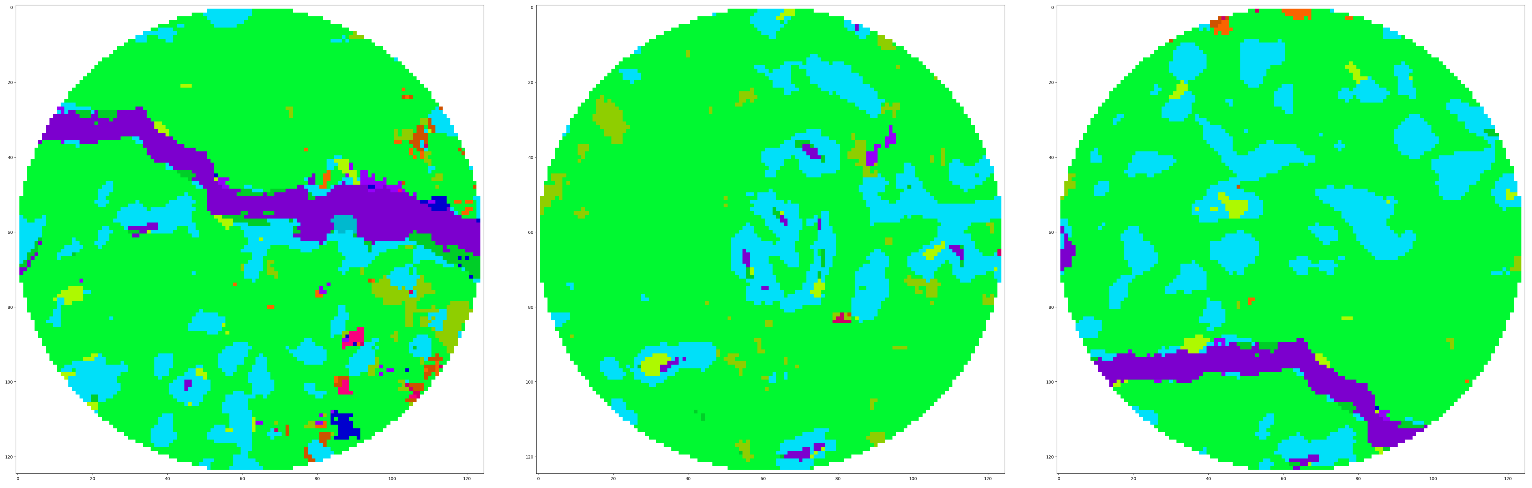

Case 69

Semi-automated output

Left: H&E stained section in the spot chosen by microscopic assessment. Middle: the first step was making an segmentation output. Right the class labels can be displayed

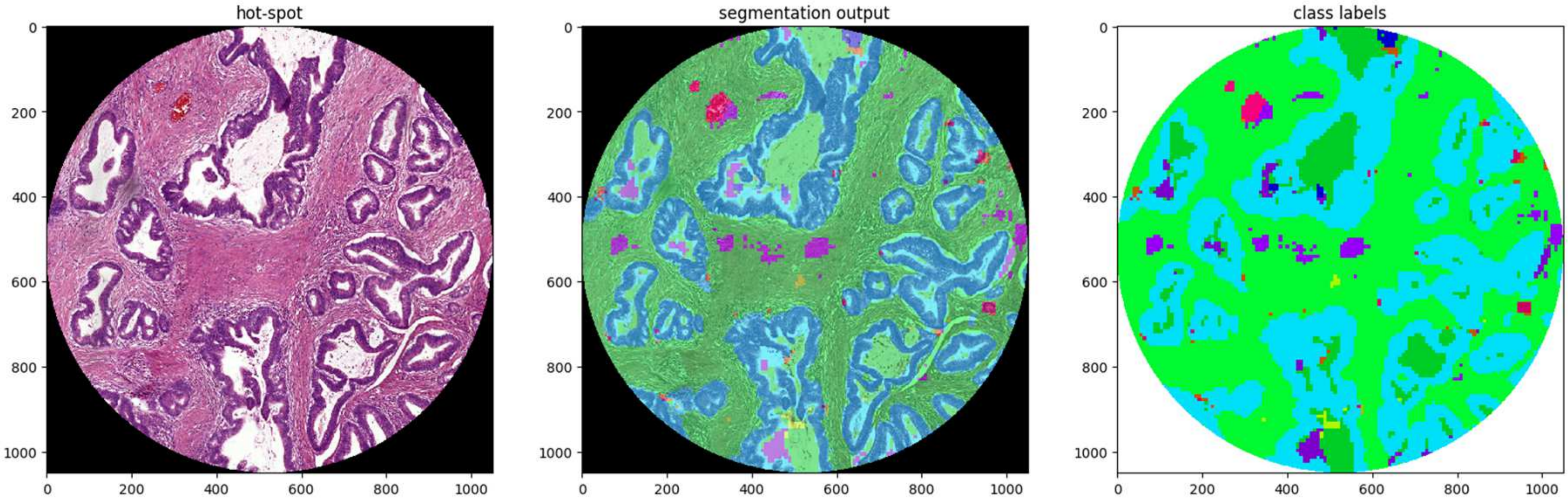

Fully-automated output

Top row; left: the tumor bulk is annotated. Right: heatmap is created. The biggest dot corresponds with the highest stroma-percentage (TSR-1), the second biggest with the second highest (TSR-2), etcetera.

Bottom row; left: the class output of the highest spot (TSR-1), middle: the second highest spot (TSR-2) and right the third highest spot (TSR-3)

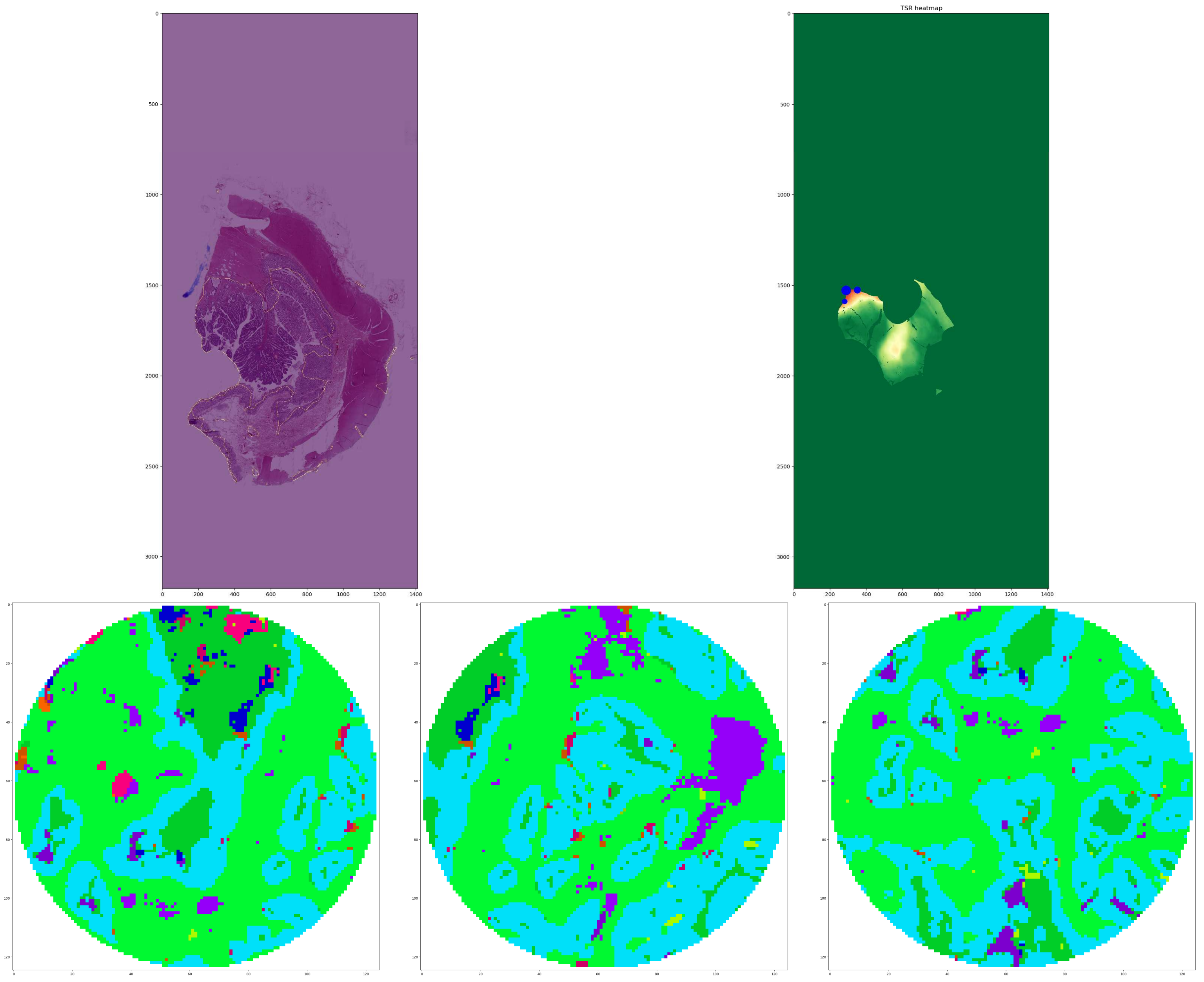

Case 70

Semi-automated output

Left: H&E stained section in the spot chosen by microscopic assessment. Middle: the first step was making an segmentation output. Right the class labels can be displayed

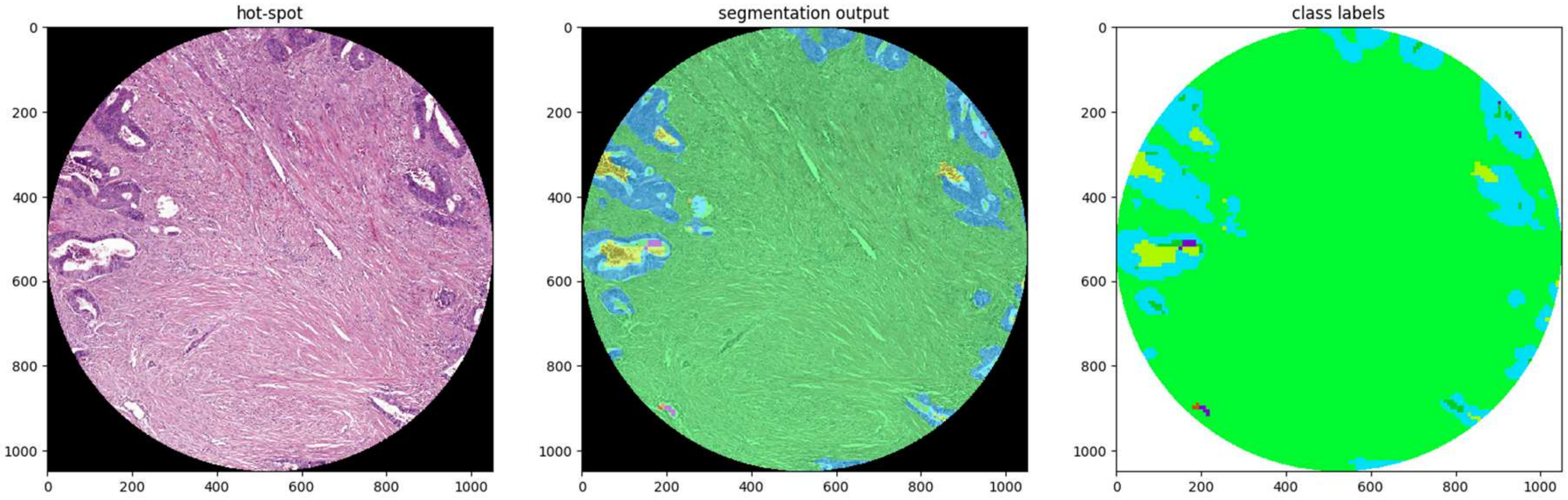

Fully-automated output

Top row; left: the tumor bulk is annotated. Right: heatmap is created. The biggest dot corresponds with the highest stroma-percentage (TSR-1), the second biggest with the second highest (TSR-2), etcetera.

Bottom row; left: the class output of the highest spot (TSR-1), middle: the second highest spot (TSR-2) and right the third highest spot (TSR-3)

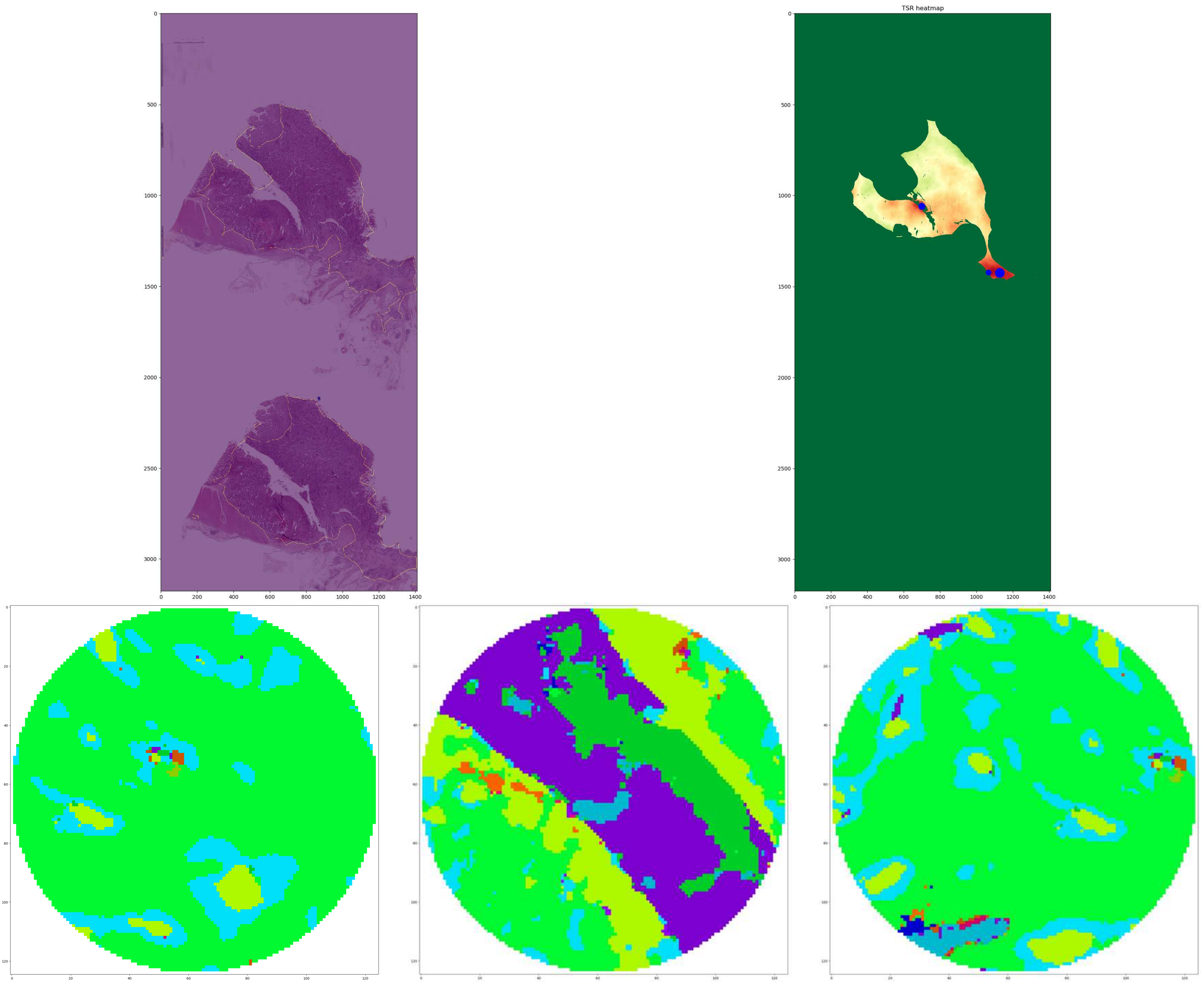

Case 71

Semi-automated output

Left: H&E stained section in the spot chosen by microscopic assessment. Middle: the first step was making an segmentation output. Right the class labels can be displayed

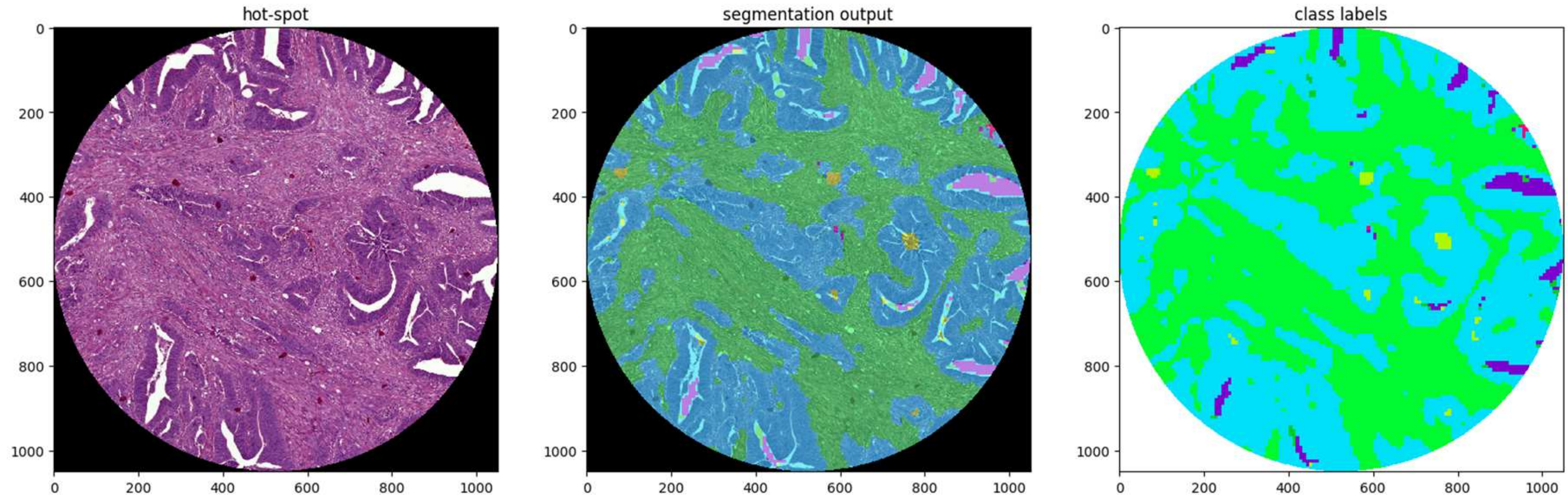

Fully-automated output

Top row; left: the tumor bulk is annotated. Right: heatmap is created. The biggest dot corresponds with the highest stroma-percentage (TSR-1), the second biggest with the second highest (TSR-2), etcetera.

Bottom row; left: the class output of the highest spot (TSR-1), middle: the second highest spot (TSR-2) and right the third highest spot (TSR-3)

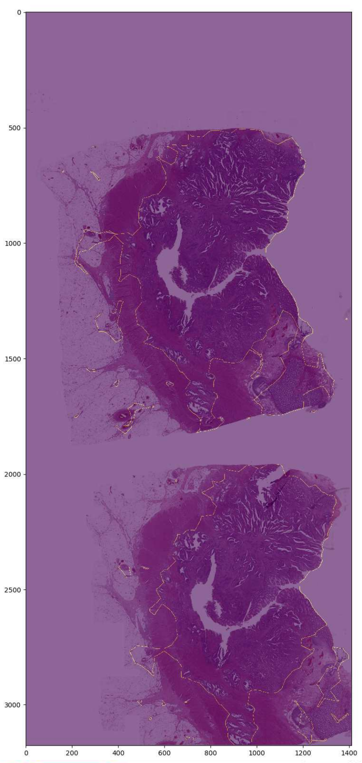

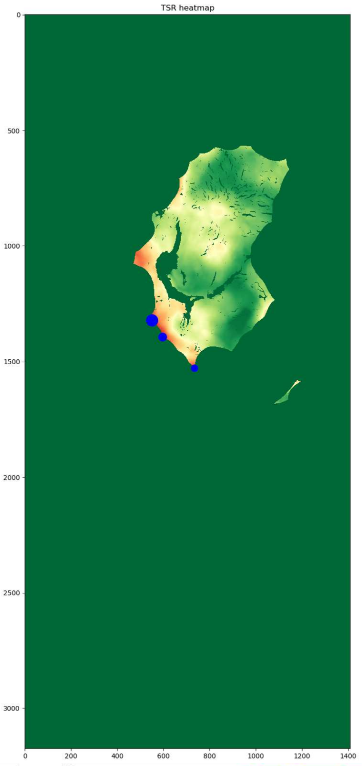

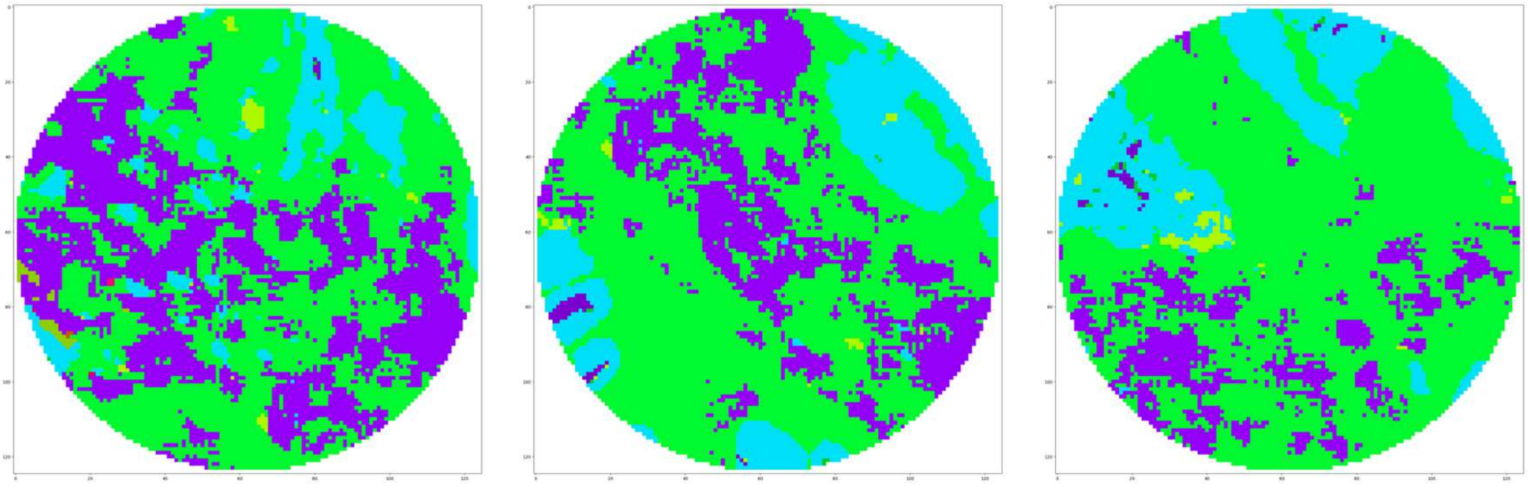

<u>Case 72</u>

Semi-automated output

Left: H&E stained section in the spot chosen by microscopic assessment. Middle: the first step was making an segmentation output. Right the class labels can be displayed

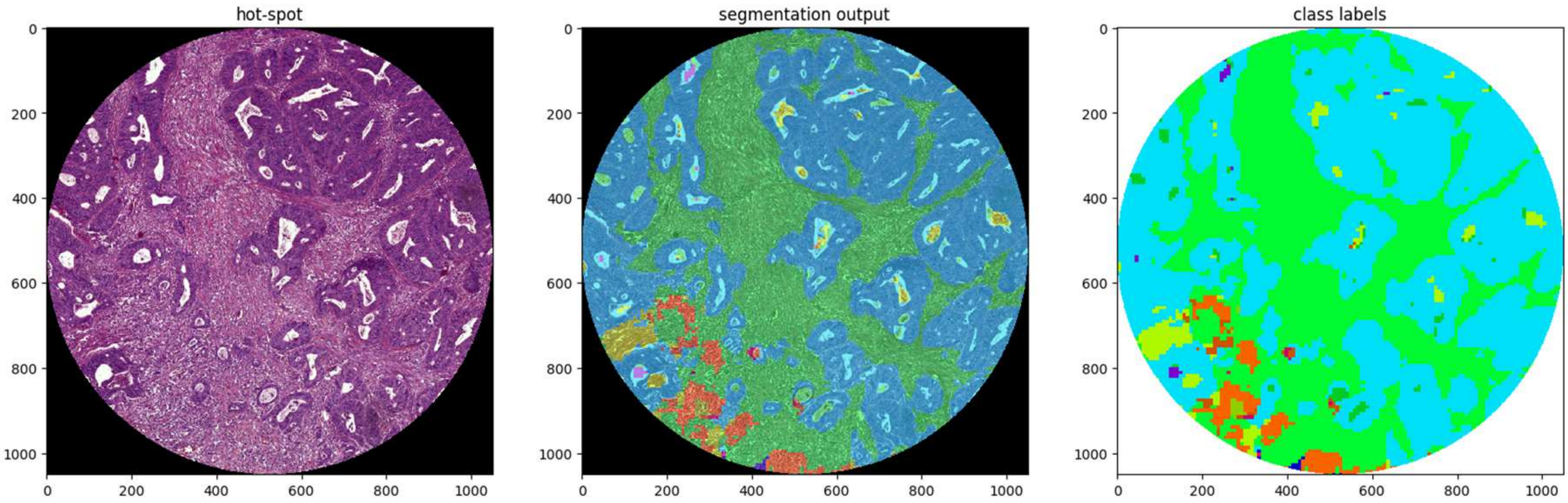

Fully-automated output

Top row; left: the tumor bulk is annotated. Right: heatmap is created. The biggest dot corresponds with the highest stroma-percentage (TSR-1), the second biggest with the second highest (TSR-2), etcetera.

Bottom row; left: the class output of the highest spot (TSR-1), middle: the second highest spot (TSR-2) and right the third highest spot (TSR-3)

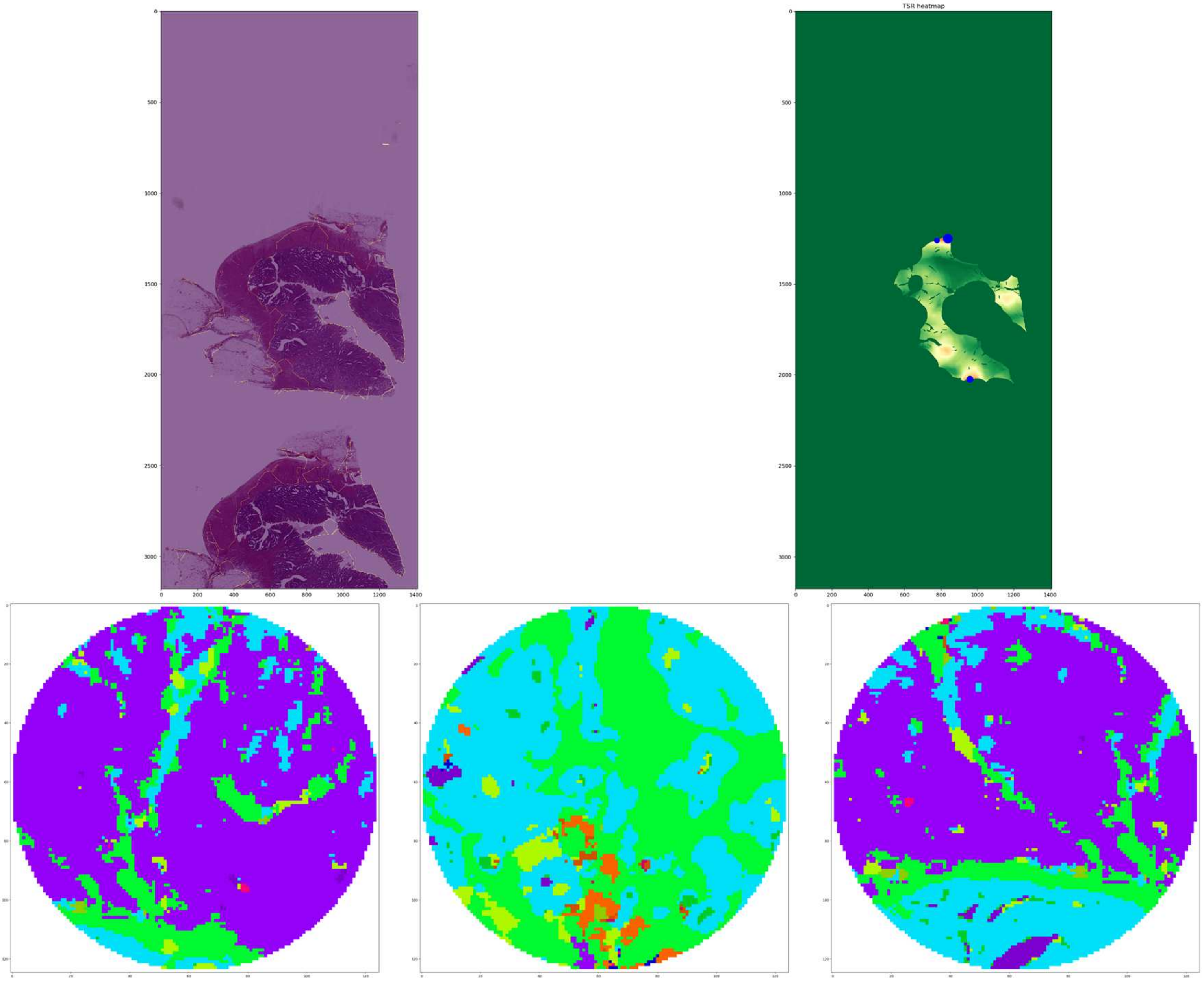

Case 73

Semi-automated output

Left: H&E stained section in the spot chosen by microscopic assessment. Middle: the first step was making an segmentation output. Right the class labels can be displayed

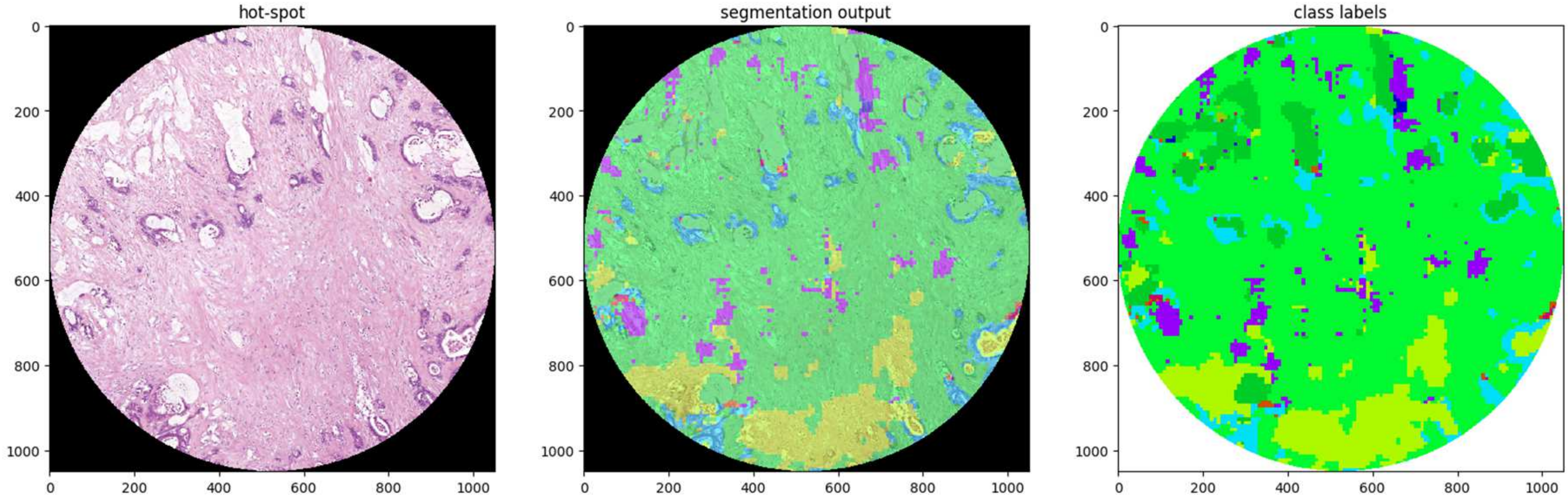

Fully-automated output

Top row; left: the tumor bulk is annotated. Right: heatmap is created. The biggest dot corresponds with the highest stroma-percentage (TSR-1), the second biggest with the second highest (TSR-2), etcetera.

Bottom row; left: the class output of the highest spot (TSR-1), middle: the second highest spot (TSR-2) and right the third highest spot (TSR-3)

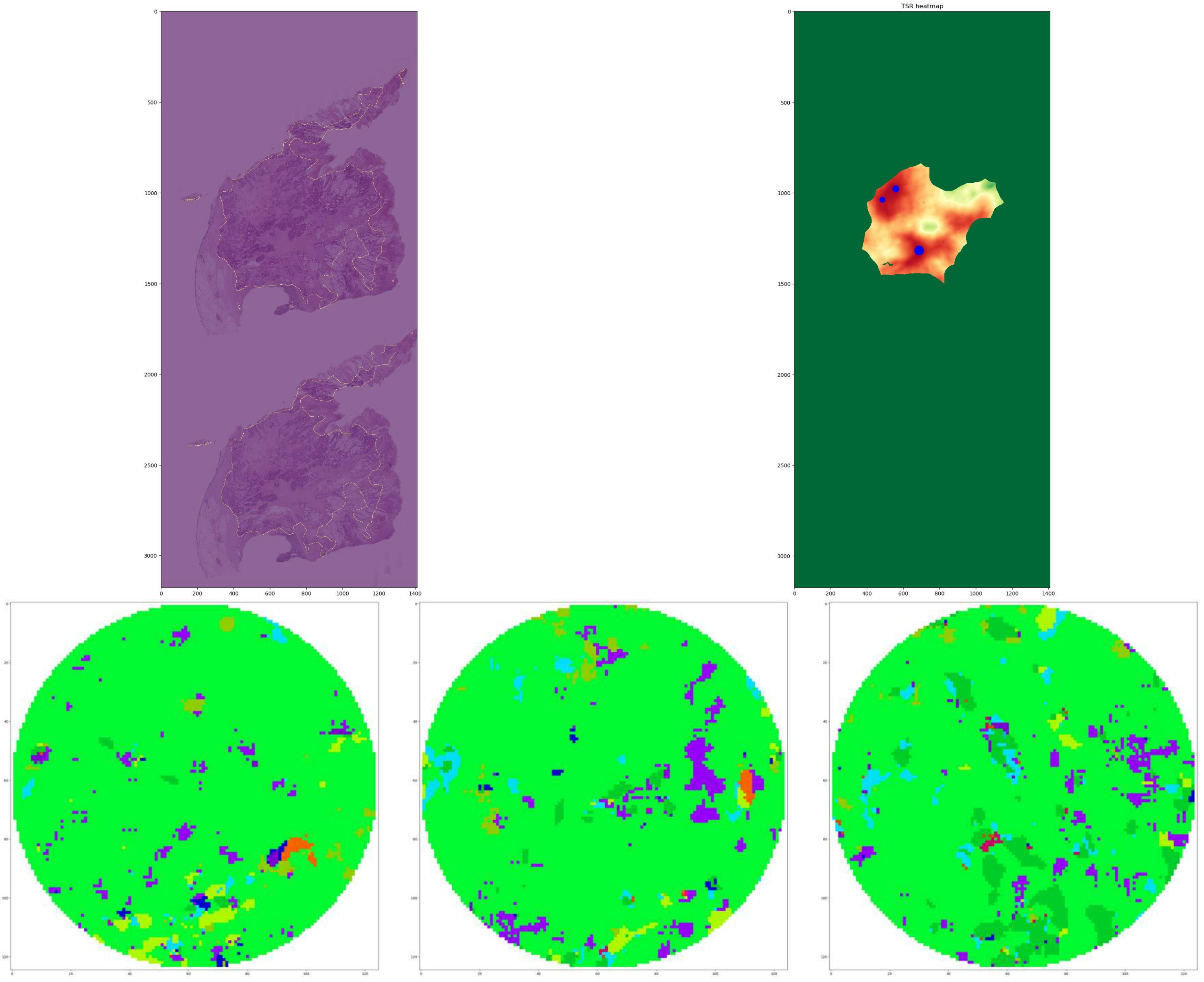

Case 74

Semi-automated output

Left: H&E stained section in the spot chosen by microscopic assessment. Middle: the first step was making an segmentation output. Right the class labels can be displayed

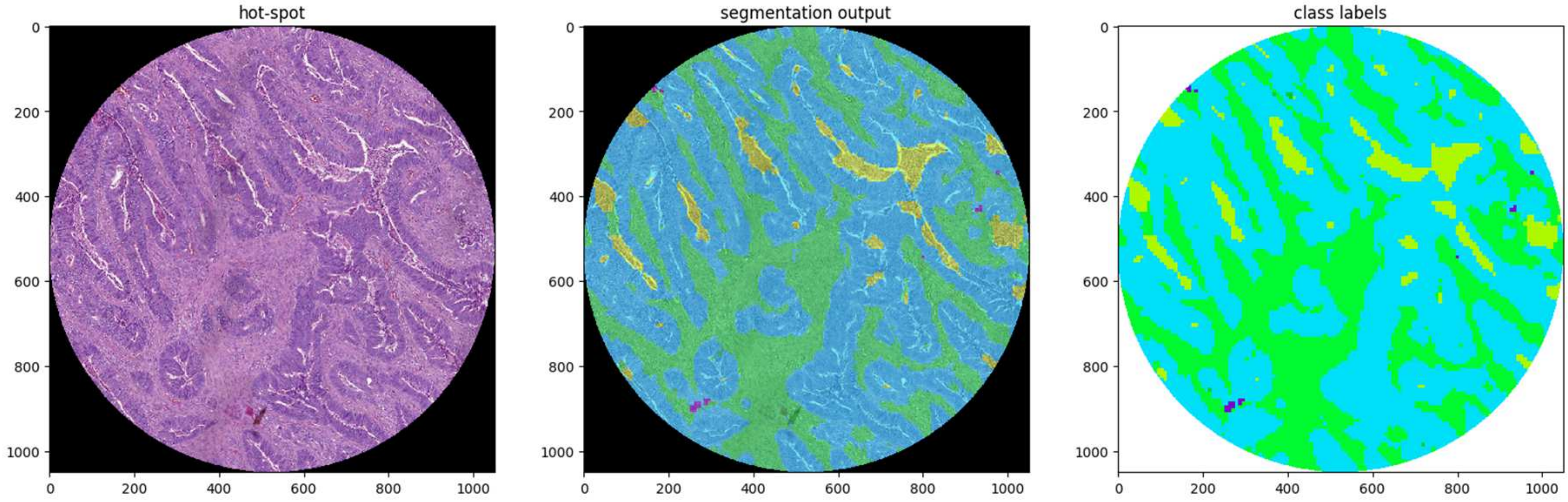

Fully-automated output

Top row; left: the tumor bulk is annotated. Right: heatmap is created. The biggest dot corresponds with the highest stroma-percentage (TSR-1), the second biggest with the second highest (TSR-2), etcetera.

Bottom row; left: the class output of the highest spot (TSR-1), middle: the second highest spot (TSR-2) and right the third highest spot (TSR-3)

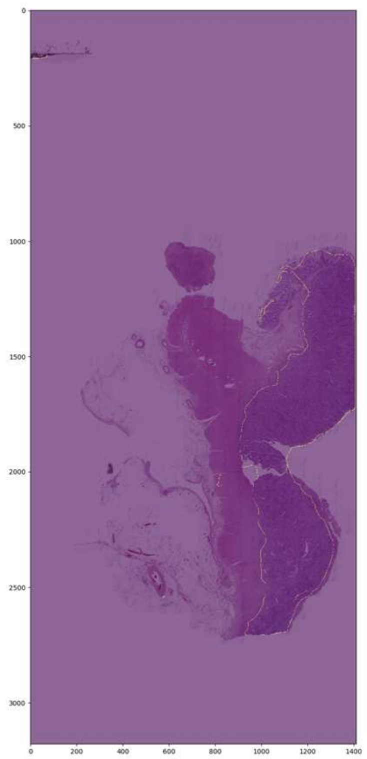

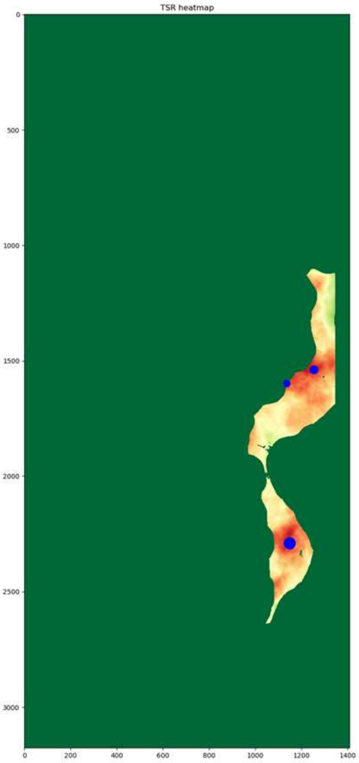

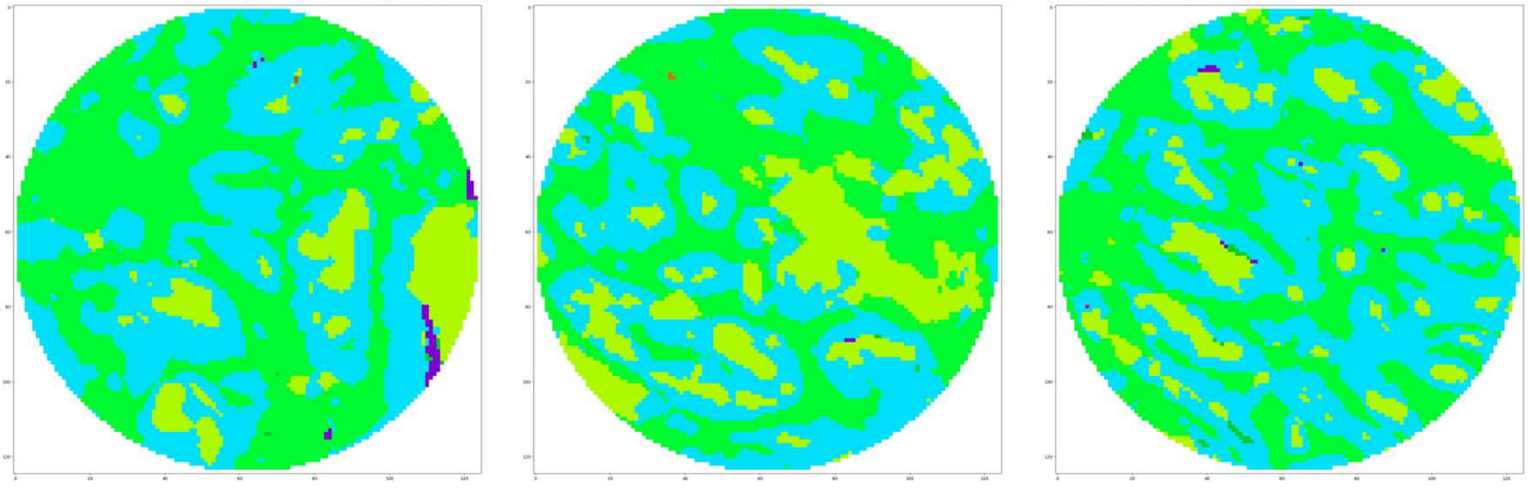

<u>Case 75</u>

Semi-automated output

Left: H&E stained section in the spot chosen by microscopic assessment. Middle: the first step was making an segmentation output. Right the class labels can be displayed

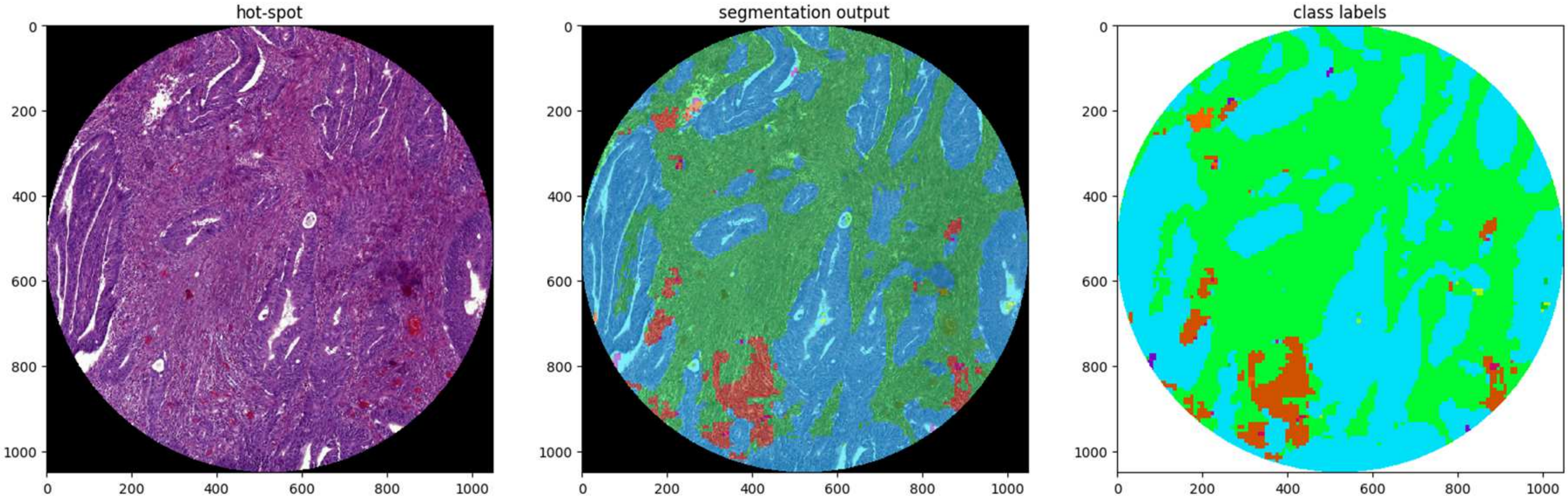

Fully-automated output

Top row; left: the tumor bulk is annotated. Right: heatmap is created. The biggest dot corresponds with the highest stroma-percentage (TSR-1), the second biggest with the second highest (TSR-2), etcetera.

Bottom row; left: the class output of the highest spot (TSR-1), middle: the second highest spot (TSR-2) and right the third highest spot (TSR-3)

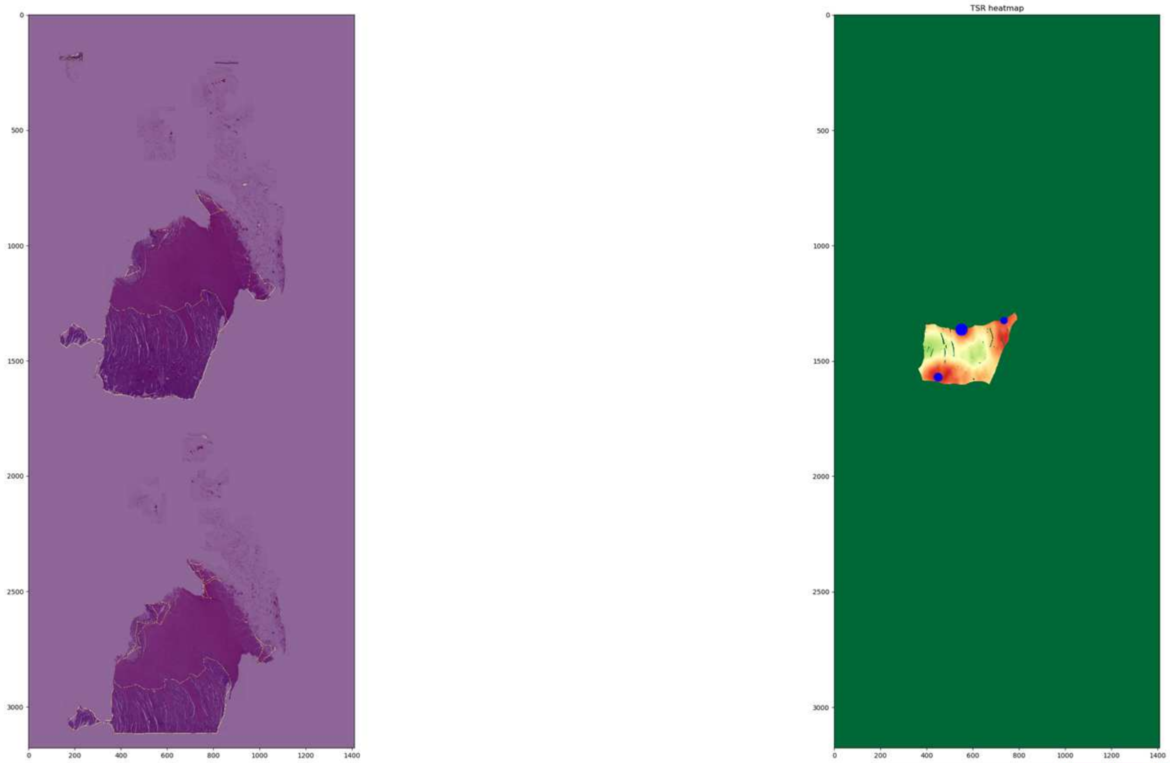

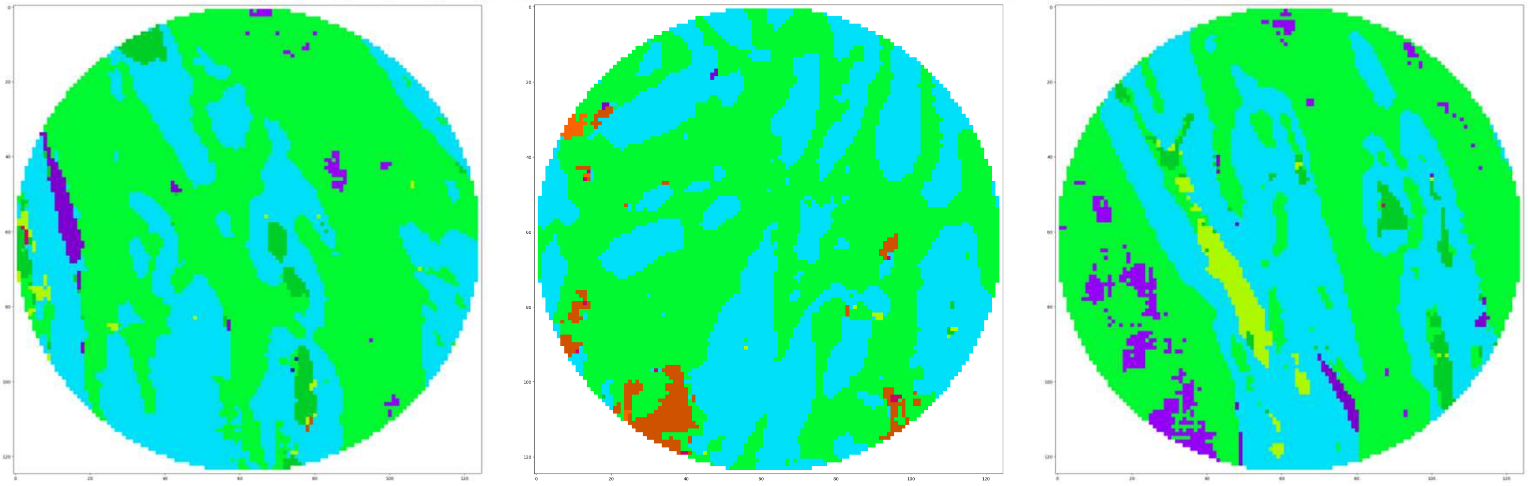

Supplement: Supplementary material 3 — Output for all cases for the semi-automated algorithm and the fully-automated algorithm. In part 1 case 1-24, part 2 case 25-49 and part 3 50-75. [file mmc3.zip › mmc3/Supplementary data - output figures (part 3).pdf]
